# Block Synthesis and Step-Growth Polymerization of C-6-Sulfonatomethyl-Containing Sulfated Malto-Oligosaccharides and Their Biological Profiling

**DOI:** 10.3390/ijms25010677

**Published:** 2024-01-04

**Authors:** Mihály Herczeg, Fruzsina Demeter, Tibor Nagy, Ágnes Rusznyák, Jan Hodek, Éva Sipos, István Lekli, Ferenc Fenyvesi, Jan Weber, Sándor Kéki, Anikó Borbás

**Affiliations:** 1 Department of Pharmaceutical Chemistry, Faculty of Pharmacy, University of Debrecen, Egyetem tér 1, H-4032 Debrecen, Hungary; demeter.fruzsina@science.unideb.hu; 2 Department of Applied Chemistry, Faculty of Science and Technology, Institute of Chemistry, University of Debrecen, Egyetem tér 1, H-4032 Debrecen, Hungary; nagy.tibor@science.unideb.hu (T.N.); keki.sandor@science.unideb.hu (S.K.); 3 Department of Molecular and Nanopharmaceutics, Faculty of Pharmacy, University of Debrecen, Nagyerdei Körút 98, H-4032 Debrecen, Hungary; rusznyak.agnes@pharm.unideb.hu (Á.R.); fenyvesi.ferenc@pharm.unideb.hu (F.F.); 4 Institute of Healthcare Industry, University of Debrecen, Egyetem tér 1, H-4032 Debrecen, Hungary; 5 Institute of Organic Chemistry and Biochemistry, Czech Academy of Sciences, Flemingovo nam. 2, CZ-16000 Prague, Czech Republic; jan.hodek@uochb.cas.cz (J.H.); jan.weber@uochb.cas.cz (J.W.); 6 Department of Pharmacodynamics, Faculty of Pharmacy, University of Debrecen, Nagyerdei Körút 98, H-4032 Debrecen, Hungary; sipos.eva@pharm.unideb.hu (É.S.); lekli.istvan@pharm.unideb.hu (I.L.); 7 HUN-REN-UD Molecular Recognition and Interaction Research Group, University of Debrecen, Egyetem tér 1, H-4032 Debrecen, Hungary

**Keywords:** malto-oligomers, sulfonic acid, polymerization, glycosylation, anti-inflammatory effect

## Abstract

Highly sulfated malto-oligomers, similar to heparin and heparan-sulfate, have good antiviral, antimetastatic, anti-inflammatory and cell growth inhibitory effects. Due to their broad biological activities and simple structure, sulfated malto-oligomer derivatives have a great therapeutic potential, therefore, the development of efficient synthesis methods for their production is of utmost importance. In this work, preparation of α-(1→4)-linked oligoglucosides containing a sulfonatomethyl moiety at position C-6 of each glucose unit was studied by different approaches. Malto-oligomeric sulfonic acid derivatives up to dodecasaccharides were prepared by polymerization using different protecting groups, and the composition of the product mixtures was analyzed by MALDI-MS methods and size-exclusion chromatography. Synthesis of lower oligomers was also accomplished by stepwise and block synthetic methods, and then the oligosaccharide products were persulfated. The antiviral, anti-inflammatory and cell growth inhibitory activity of the fully sulfated malto-oligosaccharide sulfonic acids were determined by in vitro tests. Four tested di- and trisaccharide sulfonic acids effectively inhibited the activation of the TNF-α-mediated inflammatory pathway without showing cytotoxicity.

## 1. Introduction

**Figure 1:**
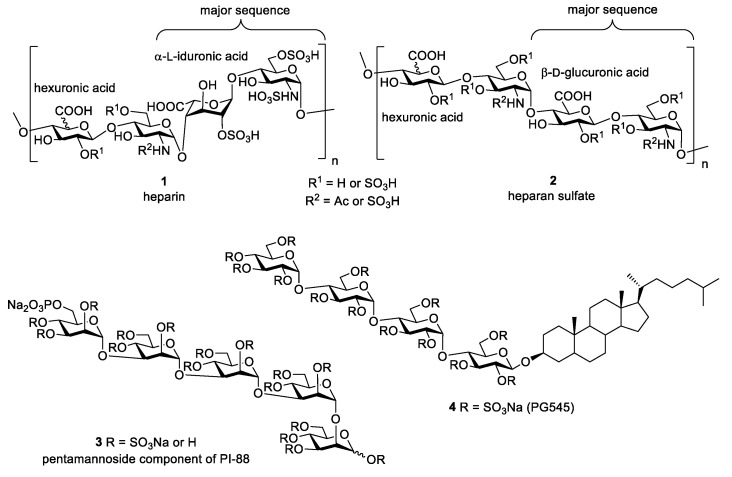
Structure of heparin (**1**), heparan sulfate (**2**), sulfated phosphomannopentaose (**3**) (major component of PI-88) and the fully sulfated maltotetraose derivative PG545 (**4**).

In recent decades, researchers have paid increasing attention to the highly negatively charged polysaccharides and higher oligosaccharides on cell surfaces or in the extracellular matrix, as these carbohydrates play important roles in many vital biological processes. An important class of these structures includes glycosaminoglycans (GAGs), which are linear, high molecular weight, polydisperse heteropolysaccharides consisting of repeating disaccharide units and often containing sulfate ester groups in various patterns [1,2,3,4,5]. The most well-known representatives of GAGs are heparin (**1**, HP) and heparan sulfate (**2**, HS) (Figure 1), which are copolymers built up alternately from d-glucosamine and a hexuronic acid [6]. Heparin has been used as an anticoagulant in the medical field since the late 1930s [7].

Both heparin and heparan sulfate have many other biological effects including anti-inflammatory, cardiovascular and tissue protective, kidney and nerve protective, angiogenic, metastasis and growth factor inhibitory as well as antimalarial, antibacterial and antiviral activity [8,9,10,11,12,13,14,15,16].

The semisynthetic heparan sulfate mimetic PI-88 (muporfostat), a mixture of highly sulfated, monophosphorylated mannose oligosaccharides up to hexasaccharide, with pentasaccharide **3** as the main component (~60%), has received significant attention due to its remarkable antiangiogenic, tumor growth inhibitory and anti-metastatic activity (Figure 1) [17,18]. PI-88, obtained from the exopolysaccharide of a diploid yeast, *Pichia holstii*, by acid-catalysed hydrolysis followed by exhaustive sulfation and its analogues is considered a promising antitumor drug candidate [11].

Another structurally simplified heparan sulfate mimetic is PG545 (**4**, pixatimod), a fully sulfated synthetic maltotetraose derivative linked to a cholestanol aglycone (Figure 1), which has shown strong in vivo efficacy in angiogenesis, solid tumor and metastasis models [19,20].

**Figure 2:**
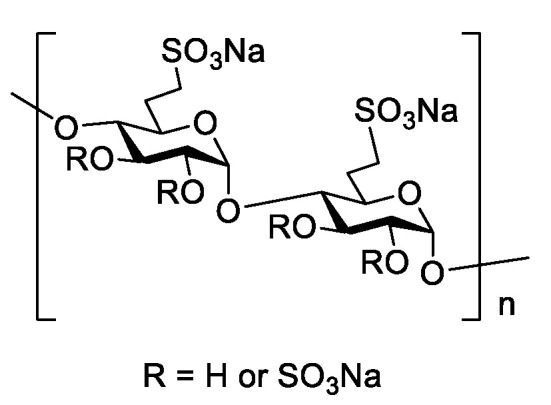
General structure of the planned C-6-sulfonatomethyl-containing malto-oligomers.

As part of our efforts to produce heparin analogues, we have synthesized a number of non-glycosaminoglycan heparinoid oligosaccharides [21,22] and malto-oligomers [23] containing sulfonatomethyl groups at different positions of the glucose units. Sulfonic acids are resistant to cleavage by esterases and are therefore more stable in vivo than their sulfated counterparts and may exhibit stronger binding affinity to target proteins. Continuing this line of research, we aimed to synthesize malto-oligomeric derivatives that contain a sulfonatomethyl group at the C-6 position of each glucose unit and their hydroxyl groups are sulfated, thus capable of forming strong ionic interactions (Figure 2). Here, we describe our oligomerization, block synthetic and step-by-step synthesis approaches for fast and facile preparation of the designed oligomers. While conventional oligosaccharide synthesis routes, such as stepwise and block synthesis, involve multiple reaction steps (glycosylation reactions; work-up procedure and deprotection after each glycosylation step), the oligomerization method offers a faster and more convenient way to produce higher oligosaccharides in a single step, one-pot manner. We also report on the anti-cell growth, anti-inflammatory and antiviral evaluation of the new malto-oligosaccharide sulfonic acids.

## 2. Results and Discussion

### 2.1. Synthesis Using Benzyl Protecting Groups

**Scheme 1:**
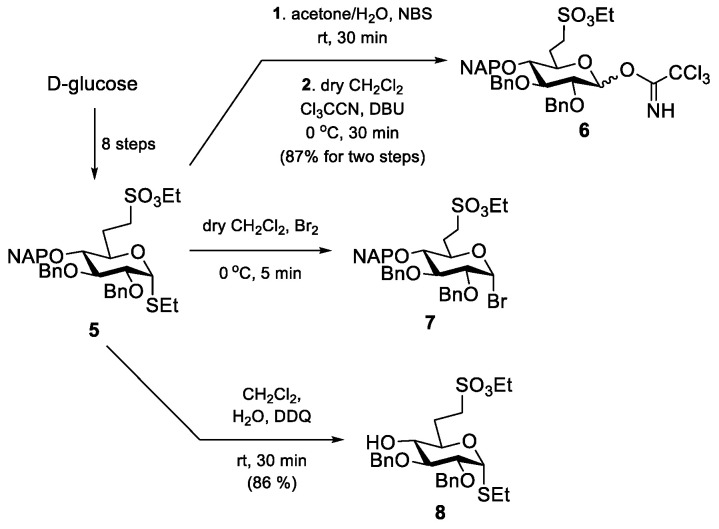
Preparation of the C6-sulfonatomethyl-containing monosaccharide donors (**6**, **7**) and acceptor (**8**).

The preparation of the planned C6-sulfonatomethyl-containing malto-oligomers requires suitable monosaccharide building blocks, which can be used to create the desired α-(1-4)-glycosidic bond with full regio- and stereoselectivity. The key monosaccharide, the 6-deoxy-6-sulfonatomethyl-containing thioglucoside **5** (Scheme 1), was obtained by the reaction of the corresponding primary triflate derivative with lithiated ethyl methanesulfonate according to our previous work [24,25]. The substitution pattern of this compound enables it to be converted into an acceptor through the selective release of 4-OH, and when used as a donor in glycosylation reactions, the presence of the non-participating benzyl group at the C-2 position along with the shielding effect of the bulky sulfonic acid ethyl ester group from the β-side ensures full α-selectivity [25,26]. Furthermore, 2-*O*-benzylated glycosyl donors are very reactive, so-called armed donors in glycosylation reactions, which presumably results in efficient glycosylation steps during the synthesis [27,28].

Thioglycoside **5** was converted to trichloroacetimidate **6** and glycosyl bromide **7**, respectively, to obtain glycosyl donors suitable for the block synthetic approach. Synthesis of imidate **6** was accomplished in two steps, including removal of the ethylthio aglycone by N-bromosuccinimide (NBS) in an in acetone/H_2_O mixture and reaction of the resulting hemiacetal with trichloroacetonitrile under alkaline conditions [29].

Due to its degradability, the bromosugar derivative (**7**) was prepared in situ before the glycosylation reaction using elemental bromine in dry CH_2_Cl_2_ [30].

The (2-naphthyl)methyl ether (NAP) of **5** was selectively removed with 2,3-dichloro-5,6-dicyano-1,4-benzoquinone (DDQ), taking advantage of its sensitivity to oxidation by the single-electron transfer mechanism [31]. The obtained thioglycoside **8** having a free 4-OH group served as a monomeric unit in the polymerization experiments and as an acceptor in the block synthetic approach.

It is important to note that polymerization of compound **8** enables the production of not only linear but also cyclic oligomers in a one-pot reaction, similarly to literature cyclooligomerizations observed in the polyglycosylation reactions of thioglycosides acting as both glycosyl donors and acceptors [32,33,34,35].

#### 2.1.1. Block Synthesis

**Scheme 2:**
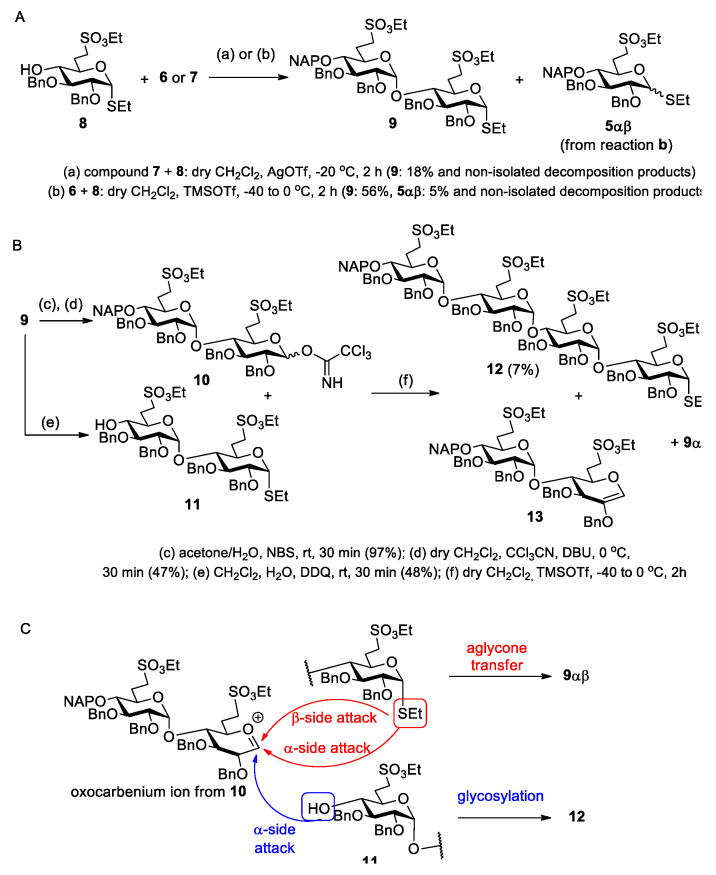
Block synthetic route to tetrasaccharide **12**. Synthesis of the disaccharide building block **9** (**A**); transformation of **9** into donor and acceptor and their condensation to tetrasaccharide **12** (**B**); the mechanism of the formation of side product **9αβ** (**C**).

We first studied the block synthesis for the production of malto-oligomeric C6-sulfonic acids (Scheme 2). When acceptor **8** was reacted with the glycosyl bromide donor **7** in the presence of AgOTf, the desired disaccharide building block **9** was obtained in a very low yield of 18%, and significant degradation was observed. In order to increase the yield, the disaccharide was also prepared by glycosylation of acceptor **8** with the imidate donor **6** using TMSOTf catalyst in dry CH_2_Cl_2_. Although the reaction did not go to completion, disaccharide **9** was isolated with a good yield of 56%. Acceptor **8** was recovered with 13% yield and thioglycoside **5** was also isolated with 5% yield in the form of an anomeric mixture. The unexpected side product **5** could be formed by the reaction of the thio-aglycone of acceptor **8** with the anomeric carbon atom of the oxocarbenium ion from donor **6** in an aglycone transfer reaction [25,36,37].

The fully protected **9** was converted into a trichloroacetimidate derivative by NBS-mediated removal of the anomeric thiol group followed by reaction with trichloroacetonitrile, thus obtaining the donor building block **10** in 47% yield. In parallel, starting from disaccharide **9**, the removal of the NAP protecting group with DDQ in a CH_2_Cl_2_/H_2_O mixture provided the disaccharide acceptor **11** with a free hydroxyl group at the C-4 position.

Glycosylation reaction of the disaccharide acceptor **11** with the disaccharide donor **10** upon TMSOTf activation proceeded with full stereoselectivity but low efficiency. The expected tetrasaccharide was isolated from the reaction mixture with a 7% yield after double purification by column chromatography. The mass spectrometric analysis of the reaction mixture (Figure S1, Supporting information) revealed that in addition to the desired tetrasaccharide, compounds **13** and **9** were also formed in the reaction. The disaccharide glycal **13** was obtained by elimination from the donor, and the fully protected thioglycoside **9** resulted from an aglycone transfer reaction, which took place competitively with the glycosylation reaction. Due to the poor yield of the desired tetrasaccharide, the block synthetic approach was not investigated further.

#### 2.1.2. Polymerization—Synthesis and MS Study

**Scheme 3:**
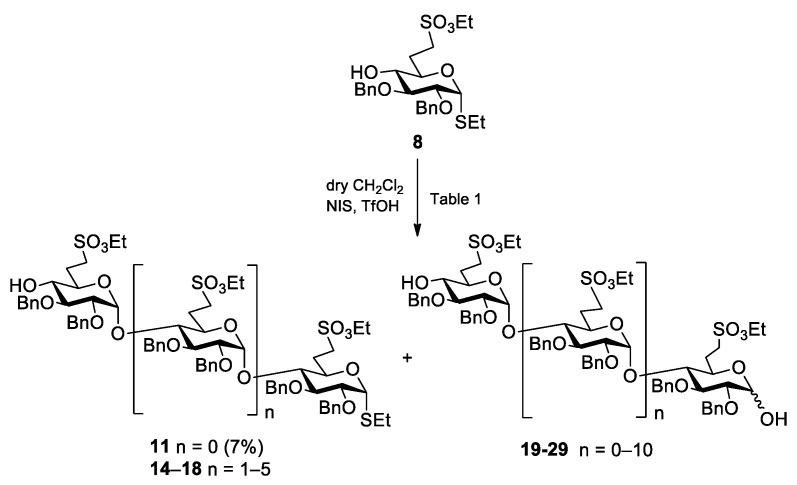
Synthesis of malto-oligomers by polymerization reaction of the C-6-sulfonatomethyl derivative **8**.

Next, we focused on the polymerization reactions of the 4-OH-containing ethyl thioglucoside **8**, which can function both as a glycosyl donor and as a glycosyl acceptor. Monomer **8** was subjected to NIS-TfOH-mediated polyglycosylation, and the scope of the reaction was examined to create linear and cyclic malto-oligosaccharides with different degrees of oligomerization (Scheme 3, Table 1). The reactions were performed using different amounts of promoter, as listed in Table 1, and after work-up, the composition of the crude reaction mixture was analysed by MALDI-TOF mass spectrometry. The standard glycosylation reaction using 1.2 equiv. of NIS resulted in linear oligosaccharides up to decamer (**19**–**27**), in the form of hemiacetals (Table 1, Reaction 1, Figures S2–S4). The corresponding thioglycoside oligomers were also present in the reaction mixture to a small extent, but surprisingly formation of cyclic oligomers was not observed.

In order to shift the composition of the product mixture from the difficult-to-isolate hemiacetal series to the easier-to-isolate thioglycosides, the polyglycosylation was performed with a substoichiometric amount of promoter. Using 0.6 equiv. of NIS, the polymerization proceeded up to hexamers in 2 h, with the predominance of di- and trisaccharides, and the formation of the oligosaccharide hemiacetals was significantly suppressed (Table 1, Reaction 2, Figure S5).

**Figure 3:**
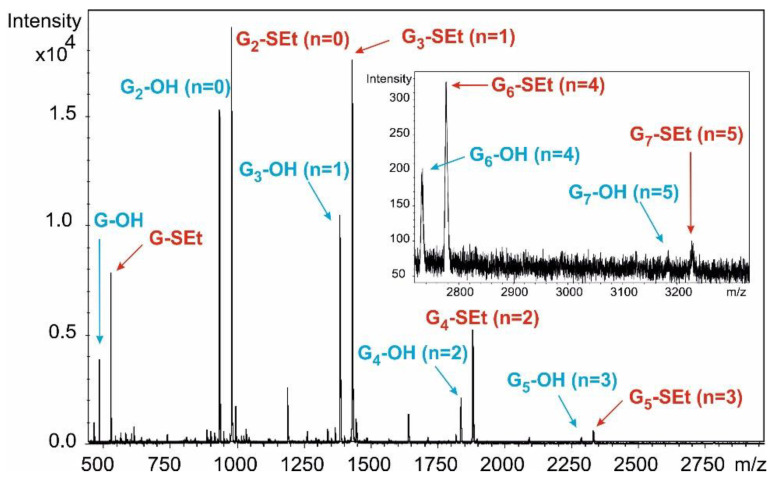
MALDI-TOF MS spectrum of the reaction mixture obtained by oligomerization reaction of **8**. The Figure inset shows the partial MALDI-TOF MS spectrum of the reaction mixture in the *m*/*z* range of 2700–3300. The oligomer series appeared in the MALDI-TOF MS spectrum are cationized by sodium ion ([M + Na]^+^).

To increase the degree of oligomerization, compound **8** was subjected to polyglycosylation in the presence of 0.6 equiv. of NIS for 24 h (Table 1, Reaction 3), and after work-up, the resulting reaction mixture was studied in detail by MALDI-TOF MS. As Figure 3 and Figure 3 inset show, two series of oligomers with decreasing MALDI-TOF MS intensities were formed up to heptamers (n = 5, see Scheme 3).

The difference between the *m*/*z* values of the neighbouring peaks in each oligomer series is 448, which corresponds to the mass of the repeat unit with a composition of C_23_H_28_O_7_S. Furthermore, based on the *m*/*z* values, our previous assumption was confirmed that one of the two oligomer series was formed with –SC_2_H_5_ (–SEt) and the other with the –OH group on the anomeric carbon atom of the last monomeric unit as indicated in Figure 3 (the mass difference between the series is 44). 

The presence of the hemiacetal oligomer series with the –OH end-group can be ascribed to the reaction of the activated –SC_2_H_5_ chain-end with water during the work-up of the reaction mixture (see the Experimental). Interestingly, the appearance of a very low intensity series of peaks with *m*/*z* values lower by 62 compared to those with the –SC_2_H_5_ end-group ([M-C_2_H_5_SH + Na]^+^) can also be recognized in the MALDI-TOF MS spectrum (see Figure S6 in the Supporting Information). This series of peaks may be due to the in-source fragmentation of the chain-end by elimination of a C_2_H_5_SH unit and/or formation of cyclic oligomers. Owing to the relatively large size of the repeat unit, formation of cyclic oligomers can only be expected at higher degrees of polymerization, e.g., when n ≥ 3. However, the [M-C_2_H_5_SH + Na]^+^ series of peaks is also present at low degrees of polymerization even at *m*/*z* 471, i.e., in the case of the monomer **8**. This finding may suggest that the [M-C_2_H_5_SH + Na]^+^ series is due to the in-source fragmentation occurring under MALDI-TOF MS conditions, although the formation of cyclic oligomer at higher degrees of polymerization cannot be ruled out.

**Figure 4:**
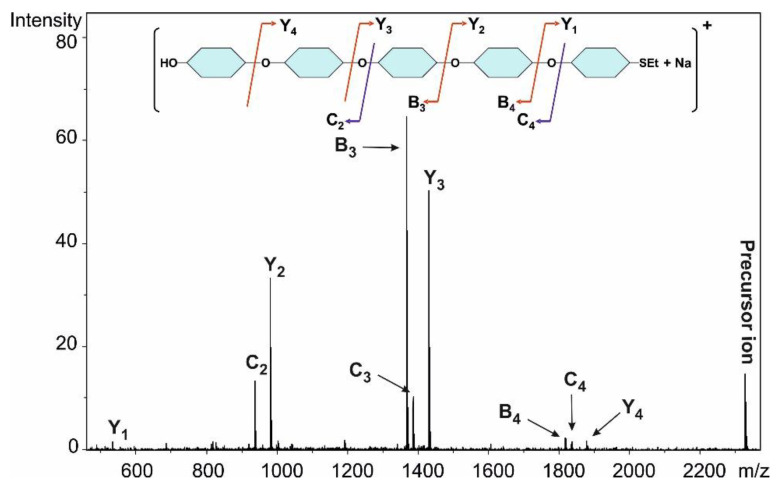
ESI-TOF MS/MS spectrum of the sodiated oligomer of **8** with n = 4 ([M + Na]^+^) recorded at a collision energy of 80 eV. The Figure inset shows the characteristics glycosidic bond cleavages designated according to Domon and Costello [38].

In order to confirm the structure of the main oligomer series formed, ESI-TOF MS/MS experiments were performed. For these experiments, the target precursor ions were selected and subjected to collision-induced dissociation (CID). The ESI-TOF MS/MS spectrum of the sodiated oligomer with the –SC_2_H_5_ end-group and with n = 4 is shown in Figure 4. As seen in the figure, the main fragmentations are due to the cleavages at the glycosidic bonds (Domon and Costello, 1988) resulting in the formation of a series of structurally important Y, B and C-type product ions, i.e., [Y_1_ + Na]^+^ (*m*/*z* 533.2), [Y_2_ + Na]^+^ (*m*/*z* 981.3), [Y_3_ + Na]^+^ (*m*/*z* 1429.5), [Y_4_ + Na]^+^ (*m*/*z* 1877.6), [B_3_ + Na]^+^ (*m*/*z* 1367.5), [B_4_ + Na]^+^ (*m*/*z* 1815.6), [C_2_ + Na]^+^ (*m*/*z* 937.3), [C_3_ + Na]^+^ (*m*/*z* 1385.5) and [C_4_ + Na]^+^ (*m*/*z* 1833.6).

After purification of the reaction mixture of (Reaction 3, Table 1) by column chromatography, disaccharide **11** was obtained with a yield of 7% in a completely pure form.

Compound **8** was also subjected to polymerization using 1.5 equiv. of NIS and 0.3 equiv. of TfOH (Table 1, Reaction 4). The reaction yielded oligomers up to dodecamers (n = 10), predominantly in the form of hemiacetals (see Figures S7 and S8 in the Supporting Information). Unfortunately, the formation of cyclic oligomers was not observed in this case either.

**Scheme 4:**
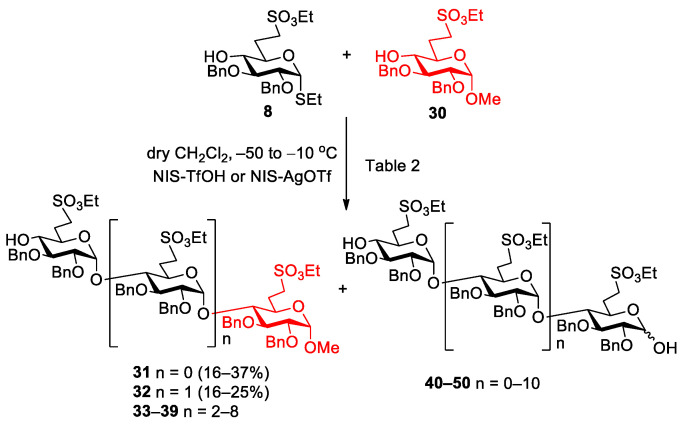
One-pot polyglycosylation reactions of the α-OMe glycoside acceptor **30** with the C-6-sulfonatomethyl monomer **8**. The capping monomer is highlighted in red.

In order to increase the amount of easily isolated oligomers and reduce the amount of difficult-to-isolate hemiacetal oligosaccharides formed as anomeric mixtures, we changed our polymerization strategy and henceforth thioglycoside **8** was subjected to a polyglycosylation reaction in the presence of a glycosyl acceptor molecule (**30**) with a fixed anomeric centre (Scheme 4).

We hypothesized that methyl glycoside **30** [25] can function as a capping unit at the reducing end, ensuring the efficient formation of the oligomer series in the form of methyl glycoside. First, the preactivation method [39,40] was used, that is, thioglycoside **8** was activated with the NIS-TfOH promoter system, then, the mixture was stirred for 2 h and only then was acceptor **30** added to the reaction mixture (Table 2, Reaction 1). Unfortunately, the expected capping did not occur, the methyl glycoside unit **30** was not incorporated into the oligomers and only hemiacetal oligomers **40**–**44** were detected by MALDI-TOF MS (Figure S9). Therefore, we switched from the preactivation method to the standard glycosylation conditions. At a 2.2:1 donor:acceptor ratio, when thioglycoside **8** was added in two portions to the reaction mixture and a NIS-TfOH promoter system was used, the expected methyl-glycoside-capped oligosaccharide series was efficiently formed. (Table 2, Reaction 2, Figure S10).

Disaccharide **31** and trisaccharide **32** were isolated from the reaction mixture with a 37% and 16% yield, respectively, and additional oligosaccharides up to octamers, both in the form of methyl glycosides and hemiacetals, were detected by MALDI-TOF MS. Using a NIS-AgOTf promoter system and a longer reaction time (Table 2, Reaction 3), but still adding the donor in two portions, the product ratio was slightly shifted to higher oligomers. Thus, disaccharide **31** was isolated with a yield of 16%, while trisaccharide **32** was isolated with a yield of 20%, and higher oligomers up to octasaccharides were identified in the MALDI mass spectra (Figure S11). The best result was achieved when polyglycosylation was started with three equivalents of thioglycoside excess (ratio of **8** and **30** was 3:1 from the beginning) using a NIS-TfOH promoter system (Table 2, Reaction 4). Both di- and trisaccharides were isolated with good yields (33% for **31** and 25% for **32**) and further oligosaccharides up to decamers were detected by MALDI MS.

The polymerization was also performed with a 10:1 ratio of **8** and **30** using 1.5 equiv. NIS and 0.3 equiv. of TfOH (Table 2, Reaction 5). In this case, the degree of oligomerization increased slightly up to the dodecamer (n = 10), but the product ratio shifted significantly towards hemiacetals. Oligosaccharides larger than the trimer were predominantly present as hemiacetals in the reaction mixture (Figures S12 and S13).

**Figure 5:**
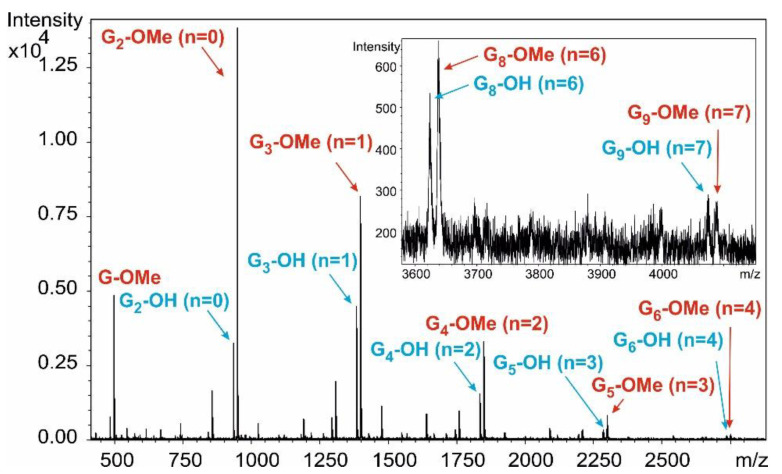
MALDI-TOF MS spectrum of the reaction mixture obtained by oligomerization reaction of **8** in the presence of **30**. The Figure inset shows the partial MALDI-TOF MS spectrum of the reaction mixture in the *m*/*z* range of 3550–4150. The oligomer series appeared in the MALDI-TOF MS spectrum are cationized by sodium ions ([M + Na]^+^).

The composition of the reaction mixture obtained by the oligomerization reaction of **8** in the presence of **30**, with a 3:1 ratio of the monomeric units (Table 2, Reaction 4), was studied by MALDI MS (Figure 5). As seen in Figure 5 and Figure 5 inset, a series of oligomers end-capped with acceptor **30** (-OMe end-group) ranging from n = 0 to n = 8 are formed in addition to an oligomer series with the –OH end-group that was most probably formed by the reaction of the activated –SC_2_H_5_ chain-end with water during the work-up of the reaction mixture as discussed before. Interestingly, according to the MALDI-TOF MS, the presence of the starting acceptor (**30**) could be observed, however, the hydrolyzed product of the starting monomer (**8**) occurred with low intensity.

**Figure 6:**
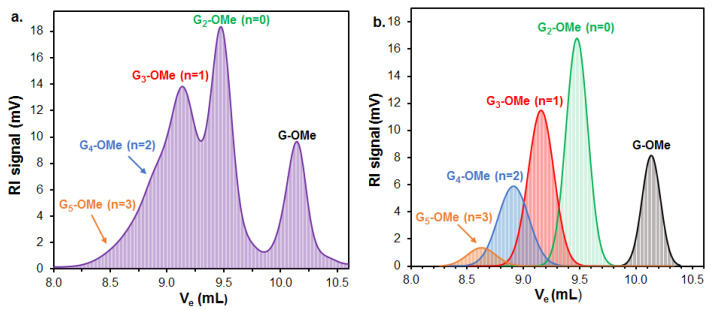
SEC chromatogram of the reaction mixture obtained after oligomerization reaction of **8** and **30** (**a**) and the deconvoluted SEC chromatogram obtained after baseline correction (**b**) Purple indicates the unresolved SEC trace in panel (**a**).

Size-exclusion chromatography (SEC) also supports the formation of oligomers with different degree of polymerization as shown in Figure 6. SEC trace presented in Figure 6a was deconvoluted into five different SEC traces corresponding to those of the starting acceptor (**30**), dimer, trimer, tetramer and pentamer as shown in Figure 6b. Note that due to the very small variation in size, oligomers with the same degree of polymerization but with different end-groups cannot be resolved by SEC.

According to the deconvoluted SEC traces and assuming a similar refractive index increment (dn/dc) for each oligomers, the composition of the reaction mixture can be estimated, for which 15 wt% (donor + hydrolyzed monomer), 35 wt% (dimer), 28 wt% (trimer), 17 wt% (tetramer) and 4 wt% (pentamer) was obtained. Note that oligomers with a higher degree of polymerization are also present in the reaction mixture as evidenced by MALDI-TOF MS, but their quantities are too low to be detected by SEC.

**Scheme 5:**
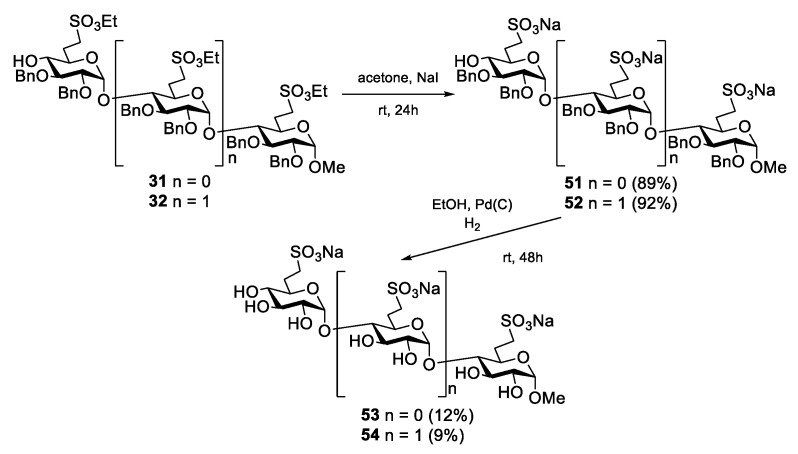
Deprotection of the di- and trisaccharide derivatives **31** and **32**.

The work was continued by removing the protecting groups of **31** and **32** in order to transform the oligosaccharides into the final, sulfated form suitable for biological assays (Scheme 5). First, the sulfonic acid esters were deprotected by nucleophilic substitution reaction using NaI. The sulfonic acid sodium salt derivatives (**51** and **52**) were formed from both di- and trisaccharides **31** and **32** in excellent yields. Next, we attempted to remove the benzyl groups by catalytic hydrogenation. Although the debenzylation went to completion, hard-to-handle gel products were obtained, probably due to complex formation with Pd, from which the expected products **53** and **54** could be isolated with very low yields.

### 2.2. Synthesis Using a Cyclic Acetal Protecting Group

**Scheme 6:**
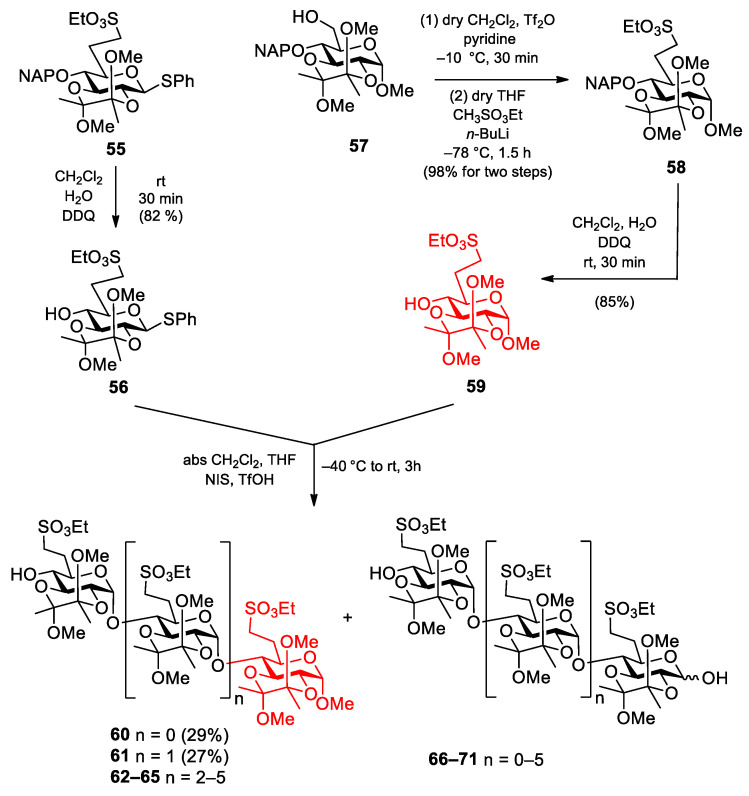
Synthesis of the BDA-protected C6-sulfonatomethyl-containing monosaccharide building blocks (**56** and **59**) and polymerization of **56** using **59** as a capping unit. The capping monomer is highlighted in red.

Due to the difficulties experienced during the removal of benzyl protecting groups, we changed the protecting group strategy in order to efficiently produce the designed free malto-oligomeric sulfonic acids. To protect the monomers, we chose the butane diacetal group (BDA, 2,3-dimethoxybutane-2,3-diyl) [41,42] because (i) it can be easily removed under acidic conditions, (ii) it is a non-participating group in glycosylation reactions and therefore enables the formation of the desired 1,2-cis α-glycosidic bond [43] and (iii) the BDA-protected donors are semi-disarmed donors (more reactive than their acylated congeners), thus, they function as effective glycosylating agents [43,44]. The synthesis of the fully protected 2,3-*O*-BDA-thioglycoside **55** was described earlier [24]. The NAP-deprotection of **55** with DDQ liberated the C4 hydroxyl group to obtain the glycosyl donor **56** suitable for oligomerization (Scheme 6). The methyl glycoside building block **59** was prepared from compound **57** [45]. First, the sulfonic acid ethyl ester moiety was introduced to the primary position in a two-step reaction (triflate formation followed by chain-elongation with lithiated ethyl methanesulfonate). The C-4 position of the resulting **58** was freed under oxidative removal of the NAP group using DDQ to produce the acceptor building block **59**.

Next, polymerization reaction was carried out with a 3:1 ratio of the repeating unit **56** and the capping glycosyl acceptor **59** using the NIS-TfOH promoter system (Scheme 6). After work-up, the series of oligosaccharides in both the methyl glycoside form and the hemiacetal form were detected by MALDI-TOF MS measurement up to the heptamers (Figure S14), and the di- and trisaccharides were isolated with acceptable yields (29% for **60** and 27% for **61**). According to our expectations, the glycosylation took place with full stereoselectivity; diastereoisomers of **60** and **61** with a β-interglycosidic bond were not observed.

**Scheme 7:**
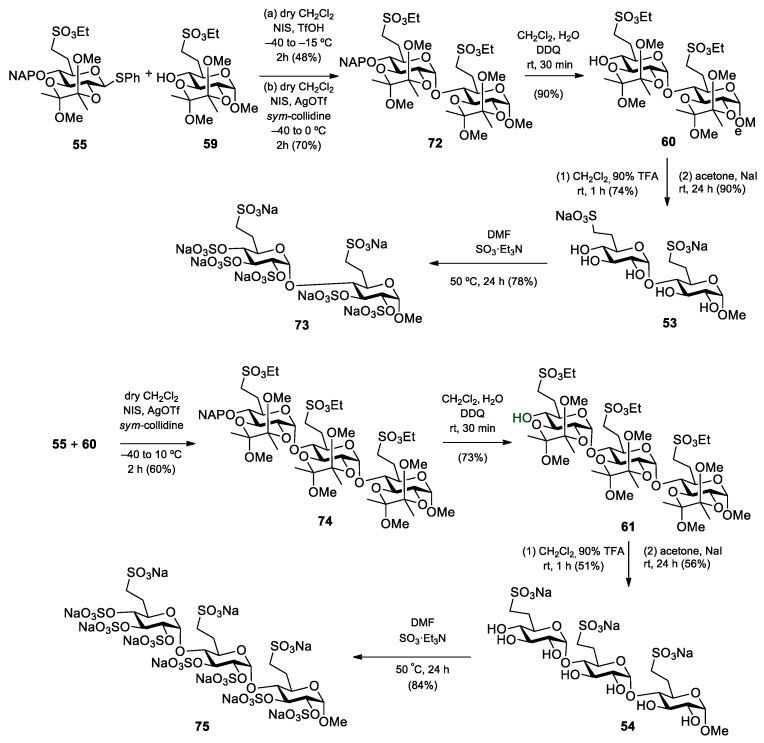
Step-wise preparation of malto-disaccharide disulfonic acids **53** and **73** and malto-trisaccharide trisulfonic acids **54** and **75**.

In parallel to the oligomerization, the di- and trisaccharide derivatives were also produced by step-by-step synthesis (Scheme 7). First, acceptor **59** was glycosylated with the fully protected donor **55** using the NIS/TfOH promoter system in dry CH_2_Cl_2_. The expected protected disaccharide (**72**) was formed with excellent stereoselectivity, but the yield was moderate (48%) due to the partial loss of the acetal-protecting group during glycosylation. To increase the yield, the reaction was repeated in the presence of *sym*-collidine to prevent cleavage of the acid-sensitive BDA. In this case, disaccharide **72** was produced with an excellent yield of 70%.

Removal of the NAP group at position 4 with DDQ afforded derivative **60**, part of which was used as an acceptor for the synthesis of the trisaccharide congener. Further deprotection of **60** included hydrolysis of the BDA groups with trifluoroacetic acid (TFA) and cleavage of the ethyl ester group of the sulfonic acid moieties with NaI. Finally, the free hydroxyl groups of **53** were sulfated with SO_3_·Et_3_N reagent in dry DMF to obtain the fully sulfated disaccharide derivative **73**.

Trisaccharide **74** was prepared analogously to the synthesis of the disaccharide congener by coupling of **55** and **60** upon NIS-AgOTf activation in the presence of *sym*-collidine. The protecting groups were then removed in three steps via NAP-deprotection (**61**) followed by acidic hydrolysis of the cyclic BDA group and conversion of the sulfonic acid esters into sulfonate salts (**54**). Treatment of **54** with SO_3_·Et_3_N resulted in the persulfated trisaccharide sulfonic acid final product **75** with good yield.

## 3. Biological Evaluation

The di- and trisaccharide sulfonic acid derivatives, both the ones with free hydroxyl groups (**53** and **54**) and the persulfated forms (**73** and **75**) were subjected to biological assays to study their cytotoxic, anti-inflammatory and antiviral effects.

It has been shown that heparin and heparinoid derivatives can prevent the binding of SARS-CoV-2 to human cells and inhibit the entry of different SARS-CoV-2 strains into cells [46,47,48]. Therefore, we hypothesized that our heparin-analogue oligosaccharides may be effective against SARS-associated coronaviruses. The anti-SARS-CoV-2 effect of the compounds was tested in Vero E6 cells (Table S1/Figure S15). Unfortunately, neither oligosaccharides showed activity against the tested strain.

The test molecules had a dose-dependent cytotoxicity on hCMEC/D3, Caco-2, HeLa, H9c2 and MCF-7 cells, with IC_50_ values higher than 50 µM for each molecule (Figures S16 and S17).

**Figure 7:**
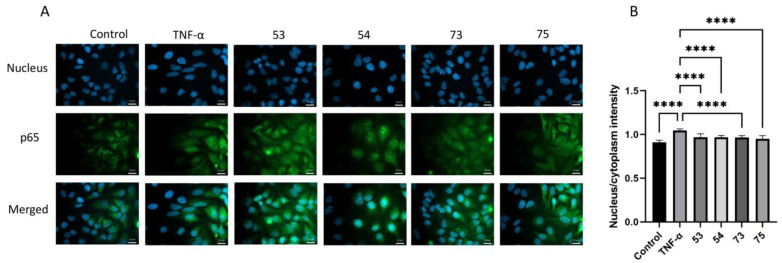
Inhibition of the NF-κB inflammatory pathway in HeLa cells by the 30 min pre-treatment of **53**, **54**, **73** and **75** compounds at a final concentration of 5 µM. (**A**) p65 subunit of NF-κB was labeled with a monoclonal antibody (green). The p65 subunit localizes in the cytoplasm in control cells, however after the inflammatory stimuly of TNF-α it translocates into the cell nucleus (blue). (**B**) Compounds **53**, **54**, **73** and **75** significantly reduced (*p* < 0.0001) the translocation of p65 subunit into the nucleus indicating a potential anti-inflammatory effect compared to the positive control TNF-α-treated cells, but not reached the control, unstimulated level. Mean ± S.D. values are shown, n = 10 cells were analyzed/group. **** *p* < 0.0001.

To examine the possible anti-inflammatory effects of compounds **53**, **54**, **73** and **75**, the inhibitory potential of NF-κB pathway activation was investigated in HeLa cells (Figure 7). Pretreatment of HeLa cells for 30 min with these compounds at a final concentration of 5 µM significantly reduced (*p* < 0.0001) the tumor necrosis factor alpha-induced (TNF-α) translocation of the NF-κB p65 subunit from the cytoplasm into the cell nucleus, inhibiting the activation of this main inflammatory pathway. According to these results, all four oligosaccharides can exert an anti-inflammatory effect on the tested cell line.

It is important to note that only sulfated oligosaccharides can be expected to have significant biological results based on the literature, so it was very surprising that the non-sulfated di- and trisaccharides (**53** and **54**, showed the same anti-inflammatory effect as their sulfated counterparts (**73** and **75**). It can be assumed that the non-sulfated oligosaccharides are able to bind to the target protein with sufficient strength through their two or three sulfonic acid groups, thus creating the carbohydrate–protein interaction necessary for the biological effect. However, further studies are necessary for a deeper understanding of the anti-inflammatory effect and to reveal the structure–effect relationships.

## 4. Conclusions

During our block synthetic approach using thioglycosides as acceptors for the production of malto-oligosaccharides, aglycone transfer side reactions took place which impaired the efficiency of the syntheses. In the polymerization reactions, if the thioglycoside building block **8** was used as both donor and acceptor, the polymerization proceeded efficiently, but difficult-to-isolate hemiacetal oligosaccharides were predominantly formed. Unfortunately, contrary to our expectations, the formed oligomers showed no tendency to cyclize, and we could not detect cyclodextrin-type products in the reaction mixtures.

The most effective methods proved to be the polymerization reaction using a methyl glycoside acceptor (**30** or **59**) as a capping monomer. The polyglycosylation reactions of the methyl glycoside acceptors proceeded efficiently and with full stereoselectivity using both benzyl and butane diacetal-protecting groups, providing oligomeric series up to dodecasaccharides. In this work, only di- and trisaccharides were isolated from the polymerization mixtures, but according to size-exclusion chromatography (SEC) analysis, the method is suitable for the rapid production of higher oligomers, up to pentasaccharides, with good/acceptable yields. The most economical method for the preparation of small oligomers, including di- and trisaccharides, was stepwise synthesis, which provided the desired malto-oligosaccharides in 60–70% yield.

Our results show that, as expected, the polymerization method resulted in oligosaccharides up to dodecamers in a single step, thus significantly shortening the synthesis. However, from a preparative point of view, it was not superior to traditional methods, since higher oligosaccharides could not be isolated in pure form due to the complexity of the polymerization mixtures.

The produced maltooligomeric di- and trisaccharide sulfonic acids proved to be inactive against SARS-CoV-2 viruses; it is assumed that the antiviral effect requires malto-oligosaccharides with a higher degree of oligomerization.

Cytotoxicity tests performed on five cell lines revealed that the di- and trisaccharide sulfonic acids tested are biocompatible, showing no significant toxicity up to a concentration of 50 µM. According to preliminary studies, the malto-oligomeric sulfonic acids deserve further investigation as potential anti-inflammatory agents, as they effectively inhibit the activation of a main inflammatory pathway induced by tumor necrosis factor alpha.

## 5. Materials and Methods

### 5.1. General Information

Optical rotations were measured at room temperature on a Perkin-Elmer 241 automatic polarimeter (PerkinElmer GmbH, Rodgau, Germany). TLC analysis was performed on Kieselgel 60 F_254_ silica-gel plates (Merck KGaA, Darmstadt, Germany) with visualization by immersing in a sulphuric-acid solution (5% in EtOH) followed by heating. Column chromatography was performed on silica gel 60 (Merck 0.063–0.200 mm). Organic solutions were dried over MgSO_4_ and concentrated under vacuum. ^1^H and ^13^C NMR spectroscopy (^1^H: 360, 400 and 500 MHz; ^13^C: 90.54, 100.28 and 125.76 MHz) were performed on Bruker DRX-360, DRX-400 and Bruker Avance II 500 spectrometers (Bruker, Billerica, MA, USA) at 25 °C. Chemical shifts are referenced to SiMe_4_ or sodium 3-(trimethylsilyl)-1-propanesulfonate (DSS, d = 0.00 ppm for ^1^H nuclei) and to residual solvent signals (CDCl_3_: *δ* = 77.16 ppm, CD_3_OD: *δ* = 49.15 ppm for ^13^C nuclei). In D_2_O, the H_2_O and CD_3_OD signals were used as a reference. MALDI-TOF MS measurements were carried out with a Bruker Autoflex Speed mass spectrometer (Bruker, Bremen, Germany) equipped with a time-of-flight (TOF) mass analyser. In all cases, 19 kV (ion source voltage 1) and 16.65 kV (ion source voltage 2) were used. For reflectron mode, 21 kV and 9.55 kV were applied as reflector voltage 1 and reflector voltage 2, respectively. A solid phase laser (355 nm, ≥100 μJ/pulse) operating at 500 Hz was applied to produce laser desorption and 3000 shots were summed. A 2,5-Dihydroxybenzoic acid (DHB) was used as the matrix and F_3_CCOONa as a cationising agent in DMF. HRMS measurements were carried out on a maXis II UHR ESI-QTOF MS instrument (Bruker, Bremen, Germany) in positive ionization mode. The following parameters were applied for the electrospray ion source: capillary voltage: 3.6 kV; end plate offset: 500 V; nebulizer pressure: 0.5 bar; dry gas temperature: 200 °C and dry gas flow rate: 4.0 L/min. Constant background correction was applied for each spectrum; the background was recorded before each sample by injecting the blank sample matrix (solvent). Na-formate calibrant was injected after each sample, which enabled internal calibration during data evaluation. Mass spectra were recorded by otofControl version 4.1 (build: 3.5, Bruker, Bremen, Germany) and processed by Compass DataAnalysis version 4.4 (build: 200.55.2969, Bruker Daltonics GmbH, Bremen, Germany).

SEC chromatograms were recorded at a flow rate of 0.5 mL/min of tetrahydrofuran (THF) using a Waters Alliance e2695 HPLC separation module (Waters Corp., Milford, MA, USA) equipped with four gel columns (4.6 × 300 mm, 5 μm Styragel columns: HR 0.5, 1, 2 and 4) and with a Waters 2414 refractive index detector (Waters Corp., Milford, MA, USA). SEC was calibrated with polystyrene standards. The concentration of the samples was 1 mg/mL.

Anti-SARS-CoV-2 activity was measured by determining the extent the compounds inhibited virus-induced cytopathic effect (CPE) and viral replication assessed by immunofluorescence assay (IFA) in Vero E6 cells (ATCC no. CRL-1586, LGC standards, Lomianki, Poland). For CPE-based assay, two-fold serial dilutions of compounds were added in triplicate to Vero E6 cells that were seeded one day before in the amount of 20,000 cells in 96-well plate in DMEM medium with 10% FBS, 100 U of penicillin/mL and 100 µg of streptomycin/mL (all Merck). After 1 h incubation, SARS-CoV-2 (strain hCoV-19/Czech Republic/NRL_6632_2/2020) was added in the multiplicity of infection (MOI) 0.01 IU/cell and the cells were incubated for 72 h at 37 °C, 5% CO_2_. After incubation, the cell viability was analyzed by formazan-based (XTT) cell proliferation assay (Thermo Fisher Scientific, Waltham, MA, USA). Briefly, mixture of XTT labelling reagent and PMS electron-coupling reagent (both Sigma-Aldrich, Budapest, Hungary) was added to the cells and incubated for 4 h at 37 °C, 5% CO_2_. The absorbance of newly formed orange formazan dye was measured in EnVision (Perkin Elmer GmbH, Rodgau, Germany) at 450 nm. The compound concentrations resulting in 50% reduction in CPE (EC_50_) were calculated from plots of percentage of absorbance versus log_10_ drug concentration using nonlinear regression using GraphPad Prism v.9.5.1 (GraphPad Software, GraphPad Software Inc, Boston, MA, USA). For immunofluorescence-based assay, Vero E6 cells and compounds were prepared same as above but SARS-CoV-2 was added at MOI 0.005 IU/cells. After three days incubation at 37 °C, 5% CO_2_, IFA was performed. Briefly, medium was removed and cells were fixed using 4% paraformaldehyde, permeabilized with 0.2% Triton-X100 (both Sigma-Aldrich, Budapest, Hungary), incubated with the first mouse anti-SARS-CoV-2 antibody (mouse monoclonal nucleoprotein IgG, ProSci Inc, Poway, CA, USA) for 2 h at room temperature followed by incubation with the second anti-mouse antibody conjugated with Cy-3 fluorophore (Jackson ImmunoResearch Europe LTD, Ely, UK) for 1.5 h. Signal was detected using fluorescent microscope with camera (Olympus, Olympus Corporation, Tokyo, Japan). Images were processed in ImageJ program (NIH, Bethesda, Maryland, USA) and compound concentrations required to reduce fluorescence signal by 50% (EC_50_) were calculated from plots of percentage of fluorescent cells versus log_10_ drug concentration as above. For cytotoxicity determination, the same setup as for CPE-based assay without the addition of virus was used. Vero E6 cytotoxicity was determined after 72 h incubation at 37 °C, 5% CO_2_ using XTT proliferation assay performed same as above. The compound concentrations resulting in a 50% reduction in cell viability (CC_50_) were calculated using nonlinear regression as above for EC_50_ determination. Remdesivir was used as a control in all experiments. The immortalized hCMEC/D3 human endothelial cell line (Merck KGaA, Darmstadt, Germany, Cat. # SCC066) was cultured in Endothelial Cell Growth Medium MV (supplemented with the components of the Supplement Pack) (Sigma-Aldrich Ltd., Budapest, Hungary).

The human Caco-2 intestinal epithelial cell line and HeLa cell line were obtained from European Collection of Cell Cultures (ECACC, Salisbury, UK) and grown in Dulbecco’s Minimum Essential Medium (DMEM), supplemented with 10% fetal bovine serum (FBS, (iBiotech, Szigetszentmiklós, Hungary), 1% non-essential amino acid and 1% penicillin-streptomycin solution, and kept in an incubator with 5% CO_2_ atmosphere. H9c2 embryonic rat heart-derived (ventricular) cells (myoblasts) from ATCC were cultured in Dulbecco’s modified Eagle’s medium (DMEM, iBiotech, Szigetszentmiklós, Hungary) supplemented with 10% fetal bovine serum (FBS) under 95% air/5% CO_2_ and subcultured at 50–60% confluence. MCF-7 human breast cancer cell lines from ATCC were cultured in Dulbecco’s modified Eagle’s medium (DMEM) supplemented with L-glutamine, 10% FBS and 1% penicillin/streptomycin in a humidified chamber at 37 °C with 5% CO_2_. Cells were subcultured every 3 days using a standard trypsinization procedure.

The anti-inflammatory study was performed in HeLa cells (ECACC, Salisbury, UK). In this experiment, 40,000 cells/well were seeded on round glass cover-slips placed into 24 well plates. Four days later, when the cells reached the appropriate confluence, cells were washed twice with HBSS and pre-incubated for 30 min at 37 °C with 5 µM solutions of **53**, **54**, **73** and **75** compounds in HBSS. After this, cells were washed twice with HBSS and incubated with TNF-α (50 ng/mL) to activate the NF-κB inflammatory pathway. After the incubation time cells were washed twice with HBSS and fixed with methanol: acetone 1:1 for 5 min at −20 °C. After this incubation time cells were washed three times with HBSS and the nonspecific binding sites were blocked with fetal bovine serum (FBS) for 15 min at room temperature. Cells were then incubated with 2 µg/mL anti-p65 antibody (RELA/NF-κB p65 Antibody (F-6) Alexa Fluor^®^ 488) (Santa Cruz Biotechnology, Inc., Heidelberg, Germany) for 1 h at 37 °C. After this incubation, cells were washed four times with HBSS and cell nuclei were stained with DAPI (283 nM) for 5 min at 37 °C. After this, cells were washed once with HBSS and the round glass coverslips were glued to the slides. Fluorescence microscopy measurements and analyses were carried out by a Zeiss Axioscope A1 (Zeiss, Jena, Germany) fluorescent microscope. The following filters were used to examine the samples: DAPI: excitation G 365 nm, emission BP 445/50 nm; fluorescein: e xcitation BP 470/40 nm, emission BP525/50 nm. Fluorescence intensities of the cytoplasms and cell nuclei were evaluated by ZEN 2011 software (Zeiss, Jena, Germany) and the nucleus/cytoplasm fluorescence intensity ratio was calculated. ANOVA staistical analysis with Tukey’s multiple comparison test was done by GraphPad Prism 9 software (GraphPad Software Inc., San Diego, CA, USA).

#### 5.1.1. General Methods

##### General Method A for Introduction of the Trichloroacetimidate Group (**6**, **10**)

To the solution of the thioglycoside (**5**, **9**) (0.482 mmol) in acetone/H_2_O (9:1, 21 mL), NBS (0.819 mmol, 1.7 equiv.) was added. The reaction mixture was stirred at room temperature for 30 min. The mixture was neutralized with a saturated aqueous solution of NaHCO_3_ (5 mL) and concentrated in vacuo. The residue was dissolved in EtOAc (100 mL), washed successively with saturated aqueous solution of NaHCO_3_ (2 × 15 mL) and H_2_O (2 × 15 mL). The organic phase was dried over MgSO_4_ and concentrated under reduced pressure. The crude product was purified by column chromatography (6:4 *n*-hexane/EtOAc) to give an αβ-mixture of the appropriate hemiacetal as a colourless syrup. The solution of the hemiacetal (0.469 mmol) in dry CH_2_Cl_2_ (7.0 mL) was cooled to 0 °C and trichloroacetonitrile (8.77 mmol, 18.7 equiv.) and DBU (0.120 mmol, 0.25 equiv.) were added. The reaction mixture was stirred for 30 min, then concentrated in vacuo at 30 °C. The residue was purified by silica gel column chromatography.

##### General Method B for Removal of the (2-Naphthyl)methyl Ether Group (**8**, **11**, **56**, **59**, **60**, **61**)

To a vigorously stirred solution of the NAP ether protected derivatives (**5**, **9**, **55**, **58**, **72**, **74**) (1 mmol) in CH_2_Cl_2_/H_2_O (9:1, 10 mL) DDQ (1.5 mmol, 1.5 equiv.) was added. The reaction mixture was stirred at room temperature for 30 min, diluted with CH_2_Cl_2_ (30 mL), washed successively with saturated aqueous solution of NaHCO_3_ (15 mL) and H_2_O (15 mL). The organic phase was dried over MgSO_4_ and concentrated under reduced pressure. The crude product was purified by silica gel column chromatography.

##### General Method C for Removal of the Ethyl ester Groups (**51**, **52**, **53**, **54**)

To a solution of the ethyl esters (**31**, **32**, **60**, **61**) (0.426 mmol) in acetone (25 mL) NaI (1.278 mmol, 1.5 equiv./ester) was added at room temperature. After 24 h, the mixture was concentrated under reduced pressure and the residue was purified by silica gel column chromatography.

##### General Method D for Persulfation (**73**, **75**)

Persulfation reactions were performed in dry DMF using SO_3_·Et_3_N complex according to literature procedures [22,26].

### 5.2. Synthesis Using Benzyl Protecting Groups

#### 5.2.1. Preparation of the Monosaccharide Building Blocks and Block Synthesis

2,3-Di-O-benzyl-6-deoxy-6-C-(ethylsulfonatomethyl)-4-O-(2-naphthyl)methyl-α,β-d-glucopyranosyl-trichloroacetimidate (**6**). Compound **5** [24] (102 mg, 0.158 mmol) was converted to **6** according to general method A. The residue was purified by silica gel column chromatography (6:4 *n*-hexane/EtOAc + 1% Et_3_N) to give **6** (89 mg, 87%) as a colourless syrup. *R*_f_ 0.67 (6:4 *n*-hexane/EtOAc + 1% Et_3_N).

Ethyl 2,3-di-O-benzyl-6-deoxy-6-C-(ethylsulfonatomethyl)-1-thio-α-d-glucopyranoside (**8**), Compound **5** [24] (1.08 g, 1.660 mmol) was converted to **8** according to general method B. The crude product was purified by silica gel column chromatography (6:4 *n*-hexane/EtOAc) to give **8** (729 mg, 86%) as a colourless syrup. *R*_f_ = 0.50 (*n*-hexane/EtOAc 6:4); [α]_D_^24^ = +77.3 (*c* = 0.22 in CHCl_3_); ^1^H NMR (400 MHz, CDCl_3_): *δ* = 7.39–7.27 (m, 10H, Ar-H), 5.32 (d, *J*_1,2_ = 5.4 Hz, 1H, H-1), 4.97 (d, *J*_gem_ = 11.4 Hz, 1H, PhC*H*_2_a), 4.72 (d, *J*_gem_ = 11.6 Hz, 1H, PhC*H*_2_a), 4.65 (d, *J*_gem_ = 11.4 Hz, 1H, PhC*H*_2_b), 4.60 (d, *J*_gem_ = 11.6 Hz, 1H, PhC*H*_2_b), 4.26 (q, *J* = 7.1 Hz, 2H, SO_3_C*H*_2_CH_3_), 4.00 (td, *J* = 3.2 Hz, *J* = 9.0 Hz, 1H, H-5), 3.75 (dd, *J* = 5.4 Hz, *J* = 9.5 Hz, 1H, H-2), 3.59 (t, *J*_2,3=3,4_ = 9.1 Hz, 1H, H-3), 3.27–3.19 (m, 2H, H-4, H-7a), 3.09 (ddd, *J* = 5.0 Hz, *J* = 11.2 Hz, *J* = 14.2 Hz, 1H, H-7b), 2.51 (qd, *J* = 4.1 Hz, *J* = 7.4 Hz, 2H, SC*H*_2_CH_3_), 2.40–2.39 (s, 1H, H-4-O*H*), 2.38–2.33 (m, 1H, H-6a), 2.01–1.92 (m, 1H, H-6b), 1.37 (t, *J* = 7.1 Hz, 3H, SO_3_CH_2_C*H*_3_), 1.28 (t, *J* = 7.4 Hz, 3H, SCH_2_C*H*_3_) ppm; ^13^C NMR (100 MHz, CDCl_3_): *δ* = 138.5, 137.7 (2C, C_q_ Ar), 128.7–128.0 (10C, Ar), 83.0 (1C, C-1), 81.4 (1C, C-3), 79.3 (1C, C-2), 75.4 (1C, Ph*C*H_2_), 73.3 (1C, C-4), 72.1 (1C, Ph*C*H_2_), 69.1 (1C, C-5), 66.2 (1C, SO_3_*C*H_2_CH_3_), 46.9 (1C, C-7), 26.1 (1C, C-6), 24.0 (1C, S*C*H_2_CH_3_), 15.2 (1C, SO_3_CH_2_*C*H_3_), 14.8 (1C, SCH_2_*C*H_3_) ppm; UHR ESI-QTOF (positive ion): *m*/*z* calcd for [C_25_H_34_O_7_S_2_ + Na]^+^: 533.1638; found: 533.1638.

Ethyl [2,3-di-O-benzyl-6-deoxy-6-C-(ethylsulfonatomethyl)-4-O-(2-naphthyl)methyl-α-d-glucopyranosyl]-(1→4)-2,3-di-O-benzyl-6-deoxy-6-C-(ethylsulfonatomethyl)-1-thio-α-d-glucopyranoside (**9**).

Method I.: A solution of **5** [24] (500 mg, 0.769 mmol) in dry CH_2_Cl_2_ (4.4 mL) was cooled to 0 °C under argon and Br_2_ (45 µL) was added. The mixture was stirred for 30 min. The reaction mixture was concentrated at 30 °C and co-evaporated with toluene (2 × 5 mL). The obtained glycosyl bromide (**7**) and acceptor **8** (450 mg, 0.880 mmol, 1.15 equiv.) were dissolved in dry CH_2_Cl_2_ (10 mL) and 4 Å molecular sieves (2.5 g) were added. The solution was stirred for 15 min at room temperature then for a further 15 min at −20 °C. AgOTf (395 mg, 1.538 mmol) dissolved in toluene (2.0 mL) was added and the mixture was allowed to warm up to room temperature in 1.5 h. The reaction mixture was diluted with CH_2_Cl_2_ (80 mL) and filtered through a pad of Celite. The filtrate was washed successively with satd. aq. solution of NaHCO_3_ (2 × 15 mL) and H_2_O (2 × 15 mL). The organic phase was dried over MgSO_4_ and concentrated. The crude product was purified by column chromatography (7:3 *n*-hexane/EtOAc) to give **9** (148 mg, 18%) as a colourless syrup.

Method II.: To the solution of acceptor **8** (46 mg, 0.090 mmol) and donor **6** (89 mg, 0.137 mmol, 1.5 equiv.) in dry CH_2_Cl_2_ (2.9 mL), 4 Å molecular sieves (0.5 g) were added. The stirred mixture was cooled to −40 °C under argon. After 30 min at this temperature, TMSOTf (2.5 µL, 0.013 mmol, 0.1 equiv.) in dry CH_2_Cl_2_ (98 µL) was added and the reaction mixture was allowed to warm up to 0 °C in 1.5 h. The mixture was diluted with CH_2_Cl_2_ (30 mL) washed successively with satd. aq. solution of NaHCO_3_ (2 × 5 mL) and H_2_O (2 × 5 mL), dried over MgSO_4_ and concentrated. The crude product was purified by column chromatography (7:3 *n*-hexane/EtOAc) to give **9** (56 mg, 56%) as a colourless syrup and **5**αβ (5 mg, 5%) as a colourless syrup. Data of **9**: *R*_f_ = 0.32 (*n*-hexane/EtOAc 7:3); [α]_D_^24^ = +78.2 (*c* = 0.11 in CHCl_3_); ^1^H NMR (360 MHz, CDCl_3_): *δ* = 7.80–7.13 (m, 27H, Ar-H), 5.58 (d, *J*_1′,2′_ = 3.8 Hz, 1H, H-1′), 5.27 (d, *J*_1,2_ = 5.3 Hz, 1H, H-1), 5.05–4.49 (m, 10H, 4 × PhC*H*_2_, NpC*H*_2_), 4.20–4.14 (m, 5H, 2 × SO_3_C*H*_2_CH_3_, H-5), 3.96 (t, *J* = 9.3 Hz, 1H, H-3′), 3.88 (t, *J* = 8.9 Hz, 1H, H-3), 3.79 (dd, *J* = 5.3 Hz, *J* = 9.1 Hz, 1H, H-2), 3.77 (dd, *J* = 2.3 Hz, *J* = 9.3 Hz, 1H, H-5′), 3.50 (dd, *J* = 8.1 Hz, *J* = 9.4 Hz, 1H, H-4), 3.47 (dd, *J* = 3.7 Hz, *J* = 9.7 Hz, 1H, H-2′), 3.30–3.18 (m, 4H, H-7′a,b, H-7a, H-4′), 3.09–3.00 (m, 1H, H-7b), 2.54–2.45 (m, 3H, SC*H*_2_CH_3_, H-6a), 2.40–2.34 (m, 1H, H-6a’), 1.99–1.89 (m, 2H, H-6b, H-6b’), 1.32–1.18 (m, 9H, 2 × SO_3_CH_2_C*H*_3_, SCH_2_C*H*_3_) ppm; ^13^C NMR (90 MHz, CDCl_3_): *δ* = 138.6, 138. 8, 138.5, 137.9, 137.5, 135.4, 133.3, 133.2 (7C, C_q_ Ar), 128.5–126.0 (27C, Ar), 96.9 (C-1′), 82.9 (C-1), 81.6 (1C, C-3), 81.5 (2C, C-3′, C-4′), 79.6 (1C, C-2), 79.5 (1C, C-2′), 77.5 (1C, C-4), 75.7, 75.3, 74.1, 73.7, 72.5 (5C, 4 × Ph*C*H_2_, Np*C*H_2_), 69.8 (1C, C-5′), 68.3 (1C, C-5), 66.7, 66.3 (2C, 2 × SO_3_*C*H_2_CH_3_), 47.3 (1C, C-7), 46.5 (1C, C-7′), 27.6 (1C, C-6), 26.5 (1C, C-6′), 24.1 (1C, S*C*H_2_CH_3_), 15.2 (2C, 2 × SO_3_CH_2_*C*H_3_), 14.9 (1C, SCH_2_CH_3_) ppm; UHR ESI-QTOF (positive ion): *m*/*z* calcd for [C_59_H_70_O_14_S_3_ + Na]^+^: 1121.3820; found: 1121.3822.

[2,3-Di-O-benzyl-6-deoxy-6-C-(ethylsulfonatomethyl)-4-O-(2-naphthyl)methyl-α-d-glucopyranosyl]-(1→4)-2,3-di-O-benzyl-6-deoxy-6-C-(ethylsulfonatomethyl)-α,β-d-glucopyranosyl-trichloroacetimidate (**10**). Compound **9** (530 mg, 0.482 mmol) was converted to **10** according to general method A. The residue was purified by column chromatography (6:4 *n*-hexane/EtOAc + 1% Et_3_N) to give **10** (263 mg, 47%) as a colourless syrup. *R*_f_ 0.72 (6:4 *n*-hexane/EtOAc + 1% Et_3_N).

Ethyl [2,3-di-O-benzyl-6-deoxy-6-C-(ethylsulfonatomethyl)-α-d-glucopyranosyl]-(1→4)-2,3-di-O-benzyl-6-deoxy-6-C-(ethylsulfonatomethyl)-1-thio-α-d-glucopyranoside (**11**) Compound **9** (377 mg, 0.343 mmol) was converted to **11** according to general method **B**. The crude product was purified by column chromatography (7:3 *n*-hexane/EtOAc) to give **11** (158 mg, 48%) as a colourless syrup. *R*_f_ = 0.22 (n-hexane/EtOAc 7:3); [α]_D_^24^ = +79.3 (*c* = 0.15 in CHCl_3_); ^1^H NMR (400 MHz, CDCl_3_): *δ* = 7.37–7.14 (m, 20H, Ar-H), 5.58 (d, *J*_1′,2′_ = 3.6 Hz, 1H, H-1′), 5.28 (d, *J*_1,2_ = 5.3 Hz, 1H, H-1), 4.97–4.46 (m, 8H, 4 × PhC*H*_2_), 4.27 (q, *J* = 7.1 Hz, 4H, 2 × SO_3_C*H*_2_CH_3_), 4.18 (td, *J* = 2.1 Hz, *J* = 9.6 Hz, 1H, H-5), 3.88 (t, *J* = 8.5 Hz, 1H, H-3), 3.81 (dd, *J* = 5.3 Hz, *J* = 9.0 Hz, 1H, H-2), 3.73–3.66 (m, 2H, H-3′, H-5′), 3.54 (t, *J* = 9.1 Hz, 1H, H-4), 3.40 (dd, *J* = 3.6 Hz, *J* = 9.7 Hz, 1H, H-2′), 3.33–3.21 (m, 4H, H-4′, H-7a, H-7′a,b), 3.06 (td, *J* = 4.6 Hz, *J* = 12.9 Hz, *J* = 14.4 Hz, 1H, H-7b), 2.52 (q, *J* = 7.1 Hz, 2H, SC*H*_2_CH_3_), 2.48–2.43 (m, 1H, H-6a), 2.38–2.31 (m, 2H, H-6a’, H-4-OH), 2.03–1.91 (m, 2H, H-6b, H-6b’), 1.39–1.35 (m, 6H, 2 × SO_3_CH_2_C*H*_3_), 1.30 (t, *J* = 7.4 Hz, 3H, SCH_2_C*H*_3_) ppm; ^13^C NMR (100 MHz, CDCl_3_): *δ* = 138.8, 138.4, 137.8, 137.4 (4C, C_q_ Ar), 128.7–126.6 (20C, Ar), 96.9 (C-1′), 82.9 (C-1), 81.4 (1C, C-3), 81.0 (C-3′), 79.6 (1C, C-2), 79.2 (1C, C-2′), 77.1 (1C, C-4), 75.4, 74.0 (2C, 2 × Ph*C*H_2_), 73.7 (1C, C-4′), 73.4, 72.5 (2C, 2 × Ph*C*H_2_),70.3 (1C, C-5′), 68.3 (1C, C-5), 66.7, 66.4 (2C, 2 × SO_3_*C*H_2_CH_3_), 47.2 (1C, C-7), 46.4 (1C, C-7′), 27.3 (1C, C-6), 26.5 (1C, C-6′), 24.1 (1C, S*C*H_2_CH_3_), 15.2 (2C, 2 × SO_3_CH_2_*C*H_3_), 14.8 (SCH_2_*C*H_3_) ppm; UHR ESI-QTOF (positive ion): *m*/*z* calcd for [C_48_H_62_O_14_S_3_ + Na]^+^: 981.3194; found: 981.3198.

Ethyl [2,3-di-O-benzyl-6-deoxy-6-C-(ethylsulfonatomethyl)-4-O-(2′-naphthyl)methyl-α-d-glucopyranosyl]-(1→4)-[2,3-di-O-benzyl-6-deoxy-6-C-(ethylsulfonatomethyl)-α-d-glucopyranosyl]-(1→4)-[2,3-di-O-benzyl-6-deoxy-6-C-(ethylsulfonatomethyl)-α-d-glucopyranosyl]-(1→4)-2,3-di-O-benzyl-6-deoxy-6-C-(ethylsulfonatomethyl)-1-thio-α-d-glucopyranoside (**12**). To the solution of acceptor **11** (140 mg, 0.146 mmol) and donor **10** (263 mg, 0.219 mmol, 1.5 equiv.) in dry CH_2_Cl_2_ (5.0 mL), 4 Å molecular sieves (1.0 g) were added. The stirred mixture was cooled to −40 °C under argon. After 30 min at this temperature, TMSOTf (4.0 µL, 0.022 mmol) in dry CH_2_Cl_2_ (100 µL) was added and the mixture was allowed to warm up to 0 °C in 1.5 h. The mixture was diluted with CH_2_Cl_2_ (40 mL), washed successively with satd. aq. solution of NaHCO_3_ (2 × 10 mL) and H_2_O (2 × 10 mL), dried over MgSO_4_ and concentrated. The crude product was purified by column chromatography (98:2 CH_2_Cl_2_/acetone) to give **12** (21 mg, 7%) as a colourless syrup. *R*_f_ = 0.39 (CH_2_Cl_2_/acetone 98:2); [α]_D_^24^ = +35.0 (*c* = 0.08 in CHCl_3_); ^1^H NMR (400 MHz, CDCl_3_): *δ* = 7.81–7.02 (m, 47H, Ar-H), 5.47 (d, *J* = 3.7 Hz, 1H, H-1), 5.42 (d, *J* = 3.7 Hz, 1H, H-1), 5.28 (d, *J* = 5.3 Hz, 1H, H-1), 5.23 (d, *J* = 3.6 Hz, 1H, H-1), 5.04–4.33 (m, 18H, 8 × PhC*H*_2_, NpC*H*_2_), 4.30–4.14 (m, 9H, H-5, 4 × SO_3_C*H*_2_CH_3_), 3.98–3.79 (m, 8H, H-2, H-3, H-3′, H-3″, H-3‴, H-5′, H-5″, H-5‴), 3.51–3.39 (m, 6H, H-2′, H-2″, H-2‴, H-4, H-4′, H-4″), 3.34–3.03 (m, 9H, H-4‴, 4 × H-7a,b), 2.56–2.35 (m, 6H, SC*H*_2_CH_3_, 4 × H-6a), 1.96–1.88 (m, 4H, 4 × H-6b), 1.39–1.22 (m, 15H, 4 × SO_3_CH_2_*C*H_3_, SCH_2_*C*H_3_) ppm; ^13^C NMR (100 MHz, CDCl_3_): *δ* = 138.8, 138.7, 138.6, 138.1, 137.9, 137.8, 137.6, 137.6, 135.5, 133.4, 133.1 (11C, C_q_ Ar), 128.6–126.0 (47C, Ar), 97.9, 97.8, 97.1 (3C, C-1′, C-1″, C-1‴), 82.9 (1C, C-1), 81.9, 81.5, 81.2, 80.4, 80.3, 80.2, 80.1, 79.5, 79.4, 79.2, 79.1, 78.8, 78.6 (12C, skeleton carbons), 75.6, 75.3, 74.3, 74.2, 73.9, 73.7, 73.6, 72.5 (9C, 8 × Ph*C*H_2_, Np*C*H_2_), 70.1, 69.6, 69.5 (3C, C-5′, C-5″, C-5‴), 68.4 (1C, C-5), 67.2, 67.1, 67.0, 66.5 (4C, 4 × SO_3_*C*H_2_CH_3_), 47.1, 46.5, 46.4, 46.3 (4C, 4 × C-7), 29.9, 27.4, 26.8, (4C, 4 × C-6), 24.2 (1C, S*C*H_2_CH_3_), 15.4, 15.2, 14.9 (5C, 4 × SO_3_CH_2_*C*H_3_, SCH_2_*C*H_3_) ppm; UHR ESI-QTOF (positive ion): *m*/*z* calcd for [C_105_H_126_O_28_S_5_ + 2Na]^2+^: 1020.8428; found: 1020.8439.

#### 5.2.2. Polymerization Reactions of Compound **8** (Table 1, Reactions 1–4)

Reaction 1. To a solution of compound **8** (100 mg, 0.196 mmol) in dry CH_2_Cl_2_ (2.0 mL), 4 Å molecular sieves (0.2 g) were added. After 30 min, the stirred mixture was cooled to −50 °C under argon and a mixture of NIS (53 mg, 0.235 mmol, 1.2 equiv.) and TfOH (5.0 µL, 0.059 mmol, 0.3 equiv.) dissolved in THF (100 µL) was added and the reaction mixture was allowed to warm up to 10 °C in 24 h. The reaction mixture was diluted with CH_2_Cl_2_ (50 mL), washed successively with 10% aq. solution of Na_2_S_2_O_3_ (2 × 10 mL), satd. aq. solution of NaHCO_3_ (2 × 10 mL) and H_2_O (2 × 10 mL), dried over MgSO_4_ and concentrated. The crude product was purified by column chromatography (7:3 *n*-hexane/EtOAc) to give a mixture of oligomers as a colourless syrup. (MALDI-TOF MS spectra of the mixture: Figures S2–S4).

Reaction 2. To a solution of compound **8** (509 mg, 1.00 mmol) in dry CH_2_Cl_2_ (15 mL), 4 Å molecular sieves (0.5 g) were added. After 30 min, the stirred mixture was cooled to −50 °C under argon and a mixture of NIS (135 mg, 0.60 mmol, 0.6 equiv.) and TfOH (16 µL, 0.18 mmol, 0.18 equiv.) dissolved in THF (500 µL) was added and the reaction mixture was allowed to warm up to −10 °C in 2 h. The reaction mixture was diluted with CH_2_Cl_2_ (150 mL), washed successively with 10% aq. Na_2_S_2_O_3_ solution (2 × 15 mL), satd. aq. solution of NaHCO_3_ (2 × 15 mL) and H_2_O (2 × 15 mL), dried over MgSO_4_ and concentrated. The crude product was purified by column chromatography (7:3 *n*-hexane/EtOAc) to give a mixture of oligomers as a colourless syrup. (MALDI-TOF MS spectra of the mixture: Figure S5).

Reaction 3. To a solution of compound **8** (509 mg, 1.00 mmol) in dry CH_2_Cl_2_ (15 mL), 4 Å molecular sieves (0.5 g) were added. After 30 min, the stirred mixture was cooled to −50 °C under argon and a mixture of NIS (135 mg, 0.60 mmol, 0.6 equiv.) and TfOH (16 µL, 0.18 mmol, 0.18 equiv.) dissolved in THF (500 µL) was added and the reaction mixture was allowed to warm up to 10 °C in 24 h. The reaction mixture was diluted with CH_2_Cl_2_ (150 mL), washed successively with 10% aq. solution of Na_2_S_2_O_3_ (2 × 15 mL), satd. aq. solution of NaHCO_3_ (2 × 15 mL) and H_2_O (2 × 15 mL), dried over MgSO_4_ and concentrated. The crude product was purified by column chromatography (7:3 *n*-hexane/EtOAc) to give **11** (63 mg, 7%) as a colourless syrup. Higher oligomers were detected by MALDI-TOF MS (Figure 3).

Reaction 4. To a solution of compound **8** (100 mg, 0.196 mmol) in dry CH_2_Cl_2_ (2.0 mL), 4 Å molecular sieves (0.2 g) were added. After 30 min, the stirred mixture was cooled to −50 °C under argon and a mixture of NIS (66 mg, 0.294 mmol, 1.5 equiv.) and TfOH (7.0 µL, 0.088 mmol, 0.3 equiv.) dissolved in THF (100 µL) was added and the reaction mixture was allowed to warm up to 10 °C in 48 h. The reaction mixture was diluted with CH_2_Cl_2_ (50 mL), washed successively with 10% aq. solution of Na_2_S_2_O_3_ (2 × 10 mL), satd. aq. solution of NaHCO_3_ (2 × 10 mL) and H_2_O (2 × 10 mL), dried over MgSO_4_ and concentrated. The crude product was purified by column chromatography (7:3 *n*-hexane/EtOAc) to give a mixture of oligomers as a colourless syrup. (MALDI-TOF MS spectra of the mixture: Figures S7 and S8).

#### 5.2.3. Polymerization Reactions of Compound **8** in the Presence of Acceptor 30 (Table 2, Reactions 1–5)

Methyl [2,3-di-O-benzyl-6-deoxy-6-C-(ethylsulfonatomethyl)-α-d-glucopyranosyl]-(1→4)-2,3-di-O-benzyl-6-deoxy-6-C-(ethylsulfonatomethyl)-α-d-glucopyranoside (**31**) and methyl [2,3-di-O-benzyl-6-deoxy-6-C-(ethylsulfonatomethyl)-α-d-glucopyranosyl]-(1→4)-[2,3-di-O-benzyl-6-deoxy-6-C-(ethylsulfonatomethyl)-α-d-glucopyranosyl]-(1→4)-2,3-di-O-benzyl-6-deoxy-6-C-(ethylsulfonatomethyl)-α-d-glucopyranoside (**32**).

Reaction 1. To a solution of compound **8** (255 mg, 0.500 mmol) in dry CH_2_Cl_2_ (18 mL), 4 Å molecular sieves (1.0 g) was added. After 30 min, the stirred mixture was cooled to −50 °C under argon and a mixture of NIS (135 mg, 0.600 mmol, 1.2 equiv.) and TfOH (6 µL, 0.072 mmol, 0.12 equiv.) dissolved in THF (250 µL) was added and the reaction mixture was allowed to warm up to −10 °C. After 2 h, compound **30** (144 mg, 0.300 mmol, 0.6 equiv.) in dry CH_2_Cl_2_ (2.0 mL) was added and the reaction mixture was stirred for 1 h, at −10 °C. After this, the reaction mixture was diluted with CH_2_Cl_2_ (100 mL), washed successively with 10% aq. solution of Na_2_S_2_O_3_ (2 × 15 mL), saturated aqueous solution of NaHCO_3_ (2 × 15 mL) and H_2_O (2 × 15 mL), dried over MgSO_4_ and concentrated. The crude product was purified by column chromatography (7:3 *n*-hexane/acetone) to give a mixture of hemiacetals as a colourless syrup. (MALDI-TOF MS spectra of the mixture: Figure S9).

Reaction 2. To a solution of compound **8** (306 mg, 0.600 mmol) and compound **30** (240 mg, 0.500 mmol, 0.8 equiv.) in dry CH_2_Cl_2_ (18 mL), 4 Å molecular sieves (1.0 g) was added. After 30 min, the stirred mixture was cooled to −50 °C under argon and a mixture of NIS (162 mg, 0.720 mmol, 1.2 equiv.) and TfOH (8.0 µL, 0.086 mmol, 0.12 equiv.) dissolved in THF (300 µL) was added and the reaction mixture was allowed to warm up to −10 °C. After 2.5 h, compound **8** (240 mg, 0.500 mmol, 0.8 equiv.) was added in dry CH_2_Cl_2_ (1.0 mL). Then, NIS (162 mg, 0.720 mmol, 1.2 equiv.) and TfOH (8.0 µL, 0.086 mmol, 0.12 equiv.) dissolved in THF (300 µL) was added and the reaction mixture was allowed to warm up to 10 °C in 5 h. The reaction mixture was diluted with CH_2_Cl_2_ (125 mL), washed successively with 10% aq. solution of Na_2_S_2_O_3_ (2 × 20 mL), saturated aqueous solution of NaHCO_3_ (2 × 20 mL) and H_2_O (2 × 20 mL), dried and concentrated. The crude product was purified by column chromatography (7:3 *n*-hexane/acetone) to give **31** (171 mg, 37%) as a colourless syrup and **32** (73 mg, 16%) as a colourless syrup. Higher oligomers were detected by MALDI-TOF MS (Figure S10).

Reaction 3. To a solution of compound **8** (177 mg, 0.346 mmol) and compound **30** (112 mg, 0.231 mmol, 0.6 equiv.) in dry CH_2_Cl_2_ (9 mL), 4 Å molecular sieves (1.0 g) was added. After 30 min, the stirred mixture was cooled to −50 °C under argon and a mixture of NIS (95 mg, 0.420 mmol, 1.2 equiv.) in dry THF (100 µL) and AgOTf (22 µL, 0.084 mmol, 0.24 equiv.) dissolved in toluene (100 µL) was added and the reaction mixture was allowed to warm up to −10 °C. After 7.5 h, compound **8** (177 mg, 0.346 mmol, 1.0 equiv.) was added in dry CH_2_Cl_2_ (1.0 mL). Then, NIS (95 mg, 0.420 mmol, 1.2 equiv.) in dry THF (100 µL) and AgOTf (22 µL, 0.084 mmol, 0.24 equiv.) dissolved in toluene (100 µL) was added and the reaction mixture was allowed to warm up to 10 °C in 24 h. The reaction mixture was diluted with CH_2_Cl_2_ (125 mL), washed successively with 10% aq. solution of Na_2_S_2_O_3_ (2 × 20 mL), saturated aqueous solution of NaHCO_3_ (2 × 20 mL) and H_2_O (2 × 20 mL), dried over MgSO_4_ and concentrated. The crude product was purified by column chromatography (7:3 *n*-hexane/acetone) to give **31** (35 mg, 16%) as a colourless syrup and **32** (44 mg, 20%) as a colourless syrup. Higher oligomers were detected by MALDI-TOF MS (Figure S11).

Reaction 4. To a solution of acceptor **30** (240 mg, 0.500 mmol) and donor **8** (765 mg, 1.50 mmol, 3.0 equiv.) in dry CH_2_Cl_2_ (18 mL), 4 Å molecular sieves (1.0 g) were added. The stirred mixture was cooled to −50 °C under argon. After 30 min at this temperature, a mixture of NIS (405 mg, 1.800 mmol, 1.2 equiv.) and TfOH (19 µL, 0.216 mmol, 0.12 equiv.) dissolved in THF (750 µL) was added and the reaction mixture was allowed to warm up to −10 °C in 12 h. The reaction mixture was diluted with CH_2_Cl_2_ (200 mL), washed successively with 10% aq. solution of Na_2_S_2_O_3_ (2 × 20 mL), satd. aq. solution of NaHCO_3_ (2 × 20 mL) and H_2_O (2 × 20 mL), dried over MgSO_4_ and concentrated. The crude product was purified by column chromatography (7:3 *n*-hexane/acetone) to give **31** (153 mg, 33%) as a colourless syrup and **32** (118 mg, 25%) as a colourless syrup. Higher oligomers were detected by MALDI-TOF MS (Figure 5).

Reaction 5. To a solution of compound **8** (100 mg, 0.196 mmol, 10 equiv.) and compound **30** (9 mg, 0.019 mmol) in dry CH_2_Cl_2_ (2.0 mL), 4 Å molecular sieves (200 mg) was added. After 30 min, the stirred mixture was cooled to −50 °C under argon and a mixture of NIS (66 mg, 0.294 mmol, 1.5 equiv.) and TfOH (7.0 µL, 0.088 mmol, 0.3 equiv.) dissolved in THF (100 µL) was added and the reaction mixture was allowed to warm up to 10 °C. After 48 h, the reaction mixture was diluted with CH_2_Cl_2_ (50 mL), washed successively with 10% aq. solution of Na_2_S_2_O_3_ (2 × 10 mL), saturated aqueous solution of NaHCO_3_ (2 × 10 mL) and H_2_O (2 × 10 mL), dried and concentrated. Higher oligomers were detected by MALDI-TOF MS (Figures S12 and S13).

Data of **31**: *R*_f_ = 0.33 (*n*-hexane/acetone 7:3); [α]_D_^24^ = +48.5 (*c* = 0.13 in CHCl_3_); ^1^H NMR (360 MHz, CDCl_3_): *δ* = 7.34–7.15 (m, 20H, Ar-H), 5.66 (d, *J*_1′,2′_ = 3.7 Hz, 1H, H-1′), 5.03–4.46 (m, 9H, 4 × PhC*H*_2_, H-1), 4.29–4.23 (m, 4H, 2 × SO_3_C*H*_2_CH_3_), 4.04 (t, *J* = 9.0 Hz, 1H), 3.81–3.66 (m, 3H) 3.56–3.37 (m, 4H), 3.34 (s, 3H, C-1-OC*H*_3_), 3.32–3.06 (m, 4H, H-7a,b, H-7‘a,b), 2.64 (s, 1H, H-4′-O*H*), 2.46–1.87 (m, 4H, H-6a,b, H-6′a,b), 1.38–1.33 (m, 6H, 2 × SO_3_CH_2_C*H*_3_) ppm; ^13^C NMR (90 MHz, CDCl_3_): *δ* = 138.8, 138.4, 137.7 (4C, C_q_ Ar), 128.6–126.5 (20C, Ar), 97.7 (C-1′), 96.8 (C-1), 81.3, 81.0, 80.3, 79.1, 76.7, 73.6, 70.2, 67.5 (8C, skeleton carbons), 75.4, 74.2, 73.4 (4C, 4 × Ph*C*H_2_), 66.7, 66.4 (2C, 2 × SO_3_*C*H_2_CH_3_), 55.5 (1C, C-1-O*C*H_3_), 46.9, 46.3 (2C, C-7, C-7′), 27.2, 26.5 (2C, C-6, C-6′), 15.2 (2C, 2 × SO_3_CH_2_*C*H_3_) ppm; MALDI-TOF (positive ion): *m*/*z* calcd for C_47_H_60_NaO_15_S_2_ [M + Na]^+^: 951.33; found: 951.35.

Data of **32**: *R*_f_ = 0.24 (*n*-hexane/acetone 7:3); [α]_D_^24^ = +51.8 (*c* = 0.11 in CHCl_3_); ^1^H NMR (360 MHz, CDCl_3_): *δ* = 7.35–7.08 (m, 30H, Ar-H), 5.56 (d, *J*_1′,2′_ = 3.4 Hz, 1H, H-1′), 5.34 (d, *J*_1″,2″_ = 3.4 Hz, 1H, H-1″), 5.01–4.39 (m, 13H, 6 × PhC*H*_2_, H-1), 4.30–4.19 (m, 6H, 3 × SO_3_C*H*_2_CH_3_), 4.09–3.44 (m, 12H), 3.36 (s, 3H, C-1-OC*H*_3_), 3.34–3.04 (m, 6H, H-7a,b, H-7‘a,b, H-7″a,b), 2.81–2.73 (m, 1H, H-4″-O*H*), 2.52–1.86 (m, 6H, H-6a,b, H-6′a,b, H-6″a,b), 1.39–1.25 (m, 9H, 3 × SO_3_CH_2_C*H*_3_) ppm; ^13^C NMR (90 MHz, CDCl_3_): *δ* = 138. 9, 138.7, 138.6, 137.9, 137.7, 137.5 (6C, C_q_ Ar), 128.7–126.6 (30C, Ar), 98.0, 97.8 (2C, C-1′, C1″), 96.6 (C-1), 81.3, 80.8, 80.4, 80.3, 79.3, 79.2, 79.0, 74.0, 70.6, 69.4, 67.8 (12C, skeleton carbons), 75.4, 75.3, 74.4, 73.5, 73.4 (6C, 6 × Ph*C*H_2_), 67.0, 66.8, 66.6 (3C, 3 × SO_3_*C*H_2_CH_3_), 55.6 (1C, C-1-O*C*H_3_), 47.0, 46.6, 46.3 (3C, C-7, C-7′, C-7″), 27.5, 27.2, 26.7 (3C, C-6, C-6′, C-6″), 15.3, 15.2 (3C, 3 × SO_3_CH_2_*C*H_3_) ppm; UHR ESI-QTOF (positive ion): *m*/*z* calcd for [C_70_H_88_O_22_S_3_ + 2Na]^2+^: 711.2357; found: 711.2357.

Methyl (6-deoxy-6-C-(sulfonatomethyl)-α-d-glucopyranosyl)-(1→4)-(6-deoxy-6-C-(sulfonatomethyl)-α-d-glucopyranoside disodium salt (**53**).

Method I: Compound **31** (396 mg, 0.158 mmol) was converted to disodium salt according to general method **C**. The residue was purified by column chromatography on Sephadex LH-20 (MeOH) to give **51** (347 mg, 89%) as a white solid. *R*_f_ = 0.43 (CH_2_Cl_2_/MeOH 8:2); [α]_D_^24^ = +56.2 (*c* = 0.13 in CHCl_3_); ^1^H NMR (360 MHz, CDCl_3_ + CD_3_OD): *δ* = 7.34–7.10 (m, 20H, Ar-H), 5.66 (s, 1H, H-1′), 4.93–4.41 (m, 9H, 4 × PhC*H*_2_, H-1), 4.01–3.55 (m, 8H), 3.36 (s, 3H, C-1-O-C*H*_3_), 3.33–3.15 (m, 4H, H-7a,b, H-7‘a,b), 2.95 (s, 1H, H-4′-O*H*), 2.49–1.95 (m, 4H, H-6a,b, H-6′a,b) ppm; ^13^C NMR (90 MHz, CDCl_3_ + CD_3_OD): *δ* = 138.5, 138.2, 137.6, 137.3 (4C, C_q_ Ar), 128.4–125.5 (20C, Ar), 97.0 (C-1′), 96.7 (C-1), 81.2, 80.9, 79.9, 78.7, 74.1, 71.1, 67.8 (8C, skeleton carbons), 74.9, 73.4, 72.8, 72.4 (4C, 4 × Ph*C*H_2_), 54.3 (1C, C-1-O*C*H_3_), 46.7, 46.1 (2C, C-7, C-7′), 27.4, 26.7 (2C, C-6, C-6′) ppm; MALDI-TOF (positive ion): *m*/*z* calcd for C_43_H_50_Na_3_O_15_S_2_ [M + Na]^+^: 939.23; found: 939.31. The disodium salt (**51**) (300 mg, 0.327 mmol) was dissolved in EtOH/AcOH (96%, 29:1, 15 mL), Pd/C (10%, 235 mg) was added and the mixture was stirred in an autoclave under a H_2_ atmosphere (10 bar) for 48 h. The catalyst was filtered through a pad of Celite and the filtrate was concentrated. The crude product was treated with Dowex ion-exchange resin (Na^+^ form) and then purified by column chromatography on Sephadex LH-20 (MeOH) to give compound **53** (21 mg, 12%) as a white solid.

Method II: To a solution of compound **60** (55 mg, 0.069 mmol) in CH_2_Cl_2_ (1.0 mL), 90% F_3_CCOOH (500 µL) was added. After 1 h, the mixture was diluted with toluene and concentrated. The residue was purified by column chromatography on Sephadex LH-20 (MeOH) to give the pentaol (25 mg, 74%) as a white solid. *R*_f_ = 0.53 (CH_2_Cl_2_/MeOH 8:2); [α]_D_^24^ = +30.0 (*c* = 0.11 in MeOH); MALDI-TOF (positive ion): *m*/*z* calcd for C_19_H_36_NaO_15_S_2_ [M + Na]^+^: 591.19; found: 591.30. The pentaol (20 mg, 0.035 mmol) was converted to **53** according to general method C in acetone (1.0 mL) and H_2_O (100 µL). The residue was purified by column chromatography on Sephadex LH-20 (MeOH) to give the disodium salt **53** (17 mg, 90%) as a white solid. *R*_f_ = 0.41 (CH_2_Cl_2_/MeOH/H_2_O 7:6:1); [α]_D_^24^ = +60.4 (*c* = 0.24 in MeOH); ^1^H NMR (400 MHz, D_2_O): *δ* = 5.58 (s, 1H, H-1′), 4.67 (d, 1H, H-1), 4.35–4.24 (m, 5H, 2 × SO_3_C*H*_2_CH_3_, H-3), 3.86–3.80 (m, 2H, H-3′, H-5), 3.74–3.56 (m, 4H, H-2, H-2′, H-4, H-5′), 3.43–3.40 (m, 1H, H-4′), 3.38, 3.36, 3.29, 3.26, 3.21 (5 × s, 15H, C-1-OC*H*_3_, 4 × BDA-OC*H*_3_), 3.23–3.19 (m, 3H, 2 × H-7a, H-7b), 3.12–3.07 (m, 1H, H-7b), 2.66–2.61 (m, 1H, H-4′-O*H*), 2.42–2.36 (m, 2H, 2 × H-6a), 2.05–1.87 (m, 2H, 2 × H-6b), 1.44–1.19 (m, 18H, 2 × SCH_2_C*H*_3_, 4 × BDA-C*H*_3_) ppm; ^13^C NMR (100 MHz, D_2_O): *δ* = 100.0 (C-1′), 99.9 (C-1), 80.8 (C-4), 74.7 (C-3), 73.9 (C-4′), 73.4 (C-3′), 72.7 (C-2′), 72.1 (C-2), 71.3 (C-5′), 68.9 (C-5), 56.1 (1C, C-1-O*C*H_3_), 47.9 (2C, C-7, C-7′), 27.8, 27.3 (2C, C-6, C-6′) ppm; UHR ESI-QTOF (positive ion): *m*/*z* calcd for C_15_H_26_Na_3_O_15_S_2_ [M + Na]^+^: 579.0401; found: 579.0402.

Methyl (6-deoxy-6-C-(sulfonatomethyl)-α-d-glucopyranosyl)-(1→4)-(6-deoxy-6-C-(sulfonatomethyl)-α-d-glucopyranosyl)]-(1→4)-(6-deoxy-6-C-(sulfonatomethyl)-α-d-glucopyranoside) trisodium salt (**54**).

Method I: Compound **32** (220 mg, 0.159 mmol) was converted to trisodium salt according to general method C. The residue was purified by column chromatography on Sephadex LH-20 (MeOH) to give the trisodium salt **52** (201 mg, 92%) as a white solid. *R*_f_ = 0.51 (CH_2_Cl_2_/MeOH 8:2); MALDI-TOF (positive ion): *m*/*z* calcd for C_64_H_73_Na_4_O_22_S_3_ [M + Na]^+^: 1381.33; found: 1381.22. The trisodium salt **52** (201 mg, 0.148 mmol) was dissolved in EtOH/AcOH (96%, 29:1, 5 mL), Pd/C (10%, 50 mg) was added and the mixture was stirred in an autoclave under a H_2_ atmosphere (10 bar) for 48 h. The catalyst was filtered through a pad of Celite and the filtrate was concentrated. The crude product was treated with Dowex ion-exchange resin (Na^+^ form) and then purified by column chromatography on Sephadex LH-20 (MeOH) to give compound **54** (11 mg, 9%) as a white solid.

Method II: To a solution of compound **61** (120 mg, 0.101 mmol) in CH_2_Cl_2_ (1.5 mL), 90% F_3_CCOOH (731 µL) was added. After 1 h, the mixture was diluted with toluene and concentrated. The residue was purified by column chromatography (85:15 CH_2_Cl_2_/MeOH) to give the heptaol (46 mg, 51%) as a white solid. *R*_f_ = 0.13 (CH_2_Cl_2_/MeOH 9:1); [α]_D_^24^ = +96.4 (*c* = 0.11 in MeOH); ^1^H NMR (400 MHz, CD_3_OD): *δ* = 5.16 (d, *J*_1′,2′_ =3.6 Hz, 1H, H-1′), 5.13 (d, *J*_1″,2″_ = 3.6 Hz, 1H, H-1″), 4.65 (d, *J*_1,2_ = 3.7 Hz, 1H, H-1), 4.36–4.27 (m, 6H, 3 × SO_3_C*H*_2_CH_3_), 3.87–3.44 (m, 12H, H-2, H-2′, H-2″, H-3, H-3′, H-3″, H-4, H-4′, H-4″, H-5, H-5′, H-5″), 3.40 (s, 3H, C-1-OC*H*_3_), 3.38–3.24 (m, 6H, 3 × H-7a,b), 2.43–2.36 (m, 3H, 3 × H-6a), 1.99–1.89 (m, 3H, 3 × H-6b), 1.42–1.36 (m, 9H, 3 × SCH_2_C*H*_3_) ppm; ^13^C NMR (100 MHz, CD_3_OD): *δ* = 103.3 (C-1), 101.1 (C-1′), 101.0 (C-1″), 85.9, 75.1, 74.7, 74.5, 74.4, 74.2, 73.6, 73.0, 72.2, 70.4, 68.9 (12C, skeleton carbons), 68.5, 68.4, 68.2 (3C, 3 × SO_3_*C*H_2_CH_3_), 56.0 (1C, C-1-O*C*H_3_), 47.2, 47.0 (3C, 3 × C-7), 27.6, 27.4, 27.3 (3C, 3 × C-6), 15.6, 15.5, 15.4 (3C, 3 × SO_3_CH_2_*C*H_3_) ppm; UHR ESI-QTOF (positive ion): *m*/*z* calcd for C_28_H_52_NaO_22_S_3_ [M + Na]^+^: 859.2005; found: 859.2002. The heptaol (40 mg, 0.048 mmol) was converted to **54** according to general method C in acetone (1.5 mL) and H_2_O (250 µL). The residue was purified by column chromatography on Sephadex LH-20 (MeOH) to give the trisodium salt **54** (22 mg, 56%) as a white solid. *R*_f_ = 0.45 (CH_2_Cl_2_/MeOH/H_2_O 7:6:1); [α]_D_^24^ = +123.0 (*c* = 0.10 in H_2_O); ^1^H NMR (400 MHz, D_2_O): *δ* = 5.53 (d, *J*_1″,2″_ = 3.7 Hz, 1H, H-1″), 5.48 (d, *J*_1′,2′_ = 3.7 Hz, 1H, H-1′), 4.80 (d, 1H, H-1), 3.98–3.87 (m, 4H, H-3, H-3′, H-5, H-5′), 3.80 (t, *J* = 7.5 Hz, 1H, H-5″), 3.71 (t, *J* = 9.5 Hz, 1H, H-3″), 3.63–3.60 (m, 2H, H-2, H-2′), 3.56 (dd, *J* = 10.0 Hz, *J* = 3.8 Hz, 1H, H-2″), 3.50–3.45 (m, 2H, H-4, H-4′), 3.43 (s, 3H, C-1-O-C*H*_3_), 3.24 (t, *J* = 9.4 Hz, 1H, H-4″), 3.16–3.01 (m, 6H, H-7a,b, H-7‘a,b, H-7″a,b), 2.39–2.28 (m, 3H, H-6a, H-6′a, H-6″a), 1.97–1.90 (m, 3H, H-6b, H-6′b, H-6″b) ppm; ^13^C NMR (100 MHz, D_2_O): *δ* = 99.9 (C-1), 99.6 (C-1″), 99.5 (C-1′), 80.6 (C-4), 79.9 (C-4′), 74.6 (C-3), 74.2 (C-3′), 73.9 (C-4″), 73.3 (C-3″), 72.7 (C-2″), 72.6, 72.2 (2C, C-2, C-2′), 71.7 (C-5″), 70.2 (C-5′), 68.9 (C-5), 56.1 (1C, C-1-O*C*H_3_), 47.9, 47.8 (3C, C-7, C-7′, C-7″), 28.1, 27.8 (2C, C-6, C-6′), 27.3 (1C, C-6″) ppm; UHR ESI-QTOF (positive ion): *m*/*z* calcd for [C_22_H_37_Na_3_O_22_S_3_ + Na]^+^: 841.0524; found: 841.0526.

Phenyl 2,3-O-(2′,3′-dimethoxybutane-2′,3′-diyl)-6-deoxy-6-C-(ethylsulfonatomethyl)-1-thio-β-d-glucopyranoside (**56**). Compound **55** (900 mg, 1.400 mmol) was converted to **56** according to general method B. The crude product was purified by column chromatography (1:1 *n*-hexane/EtOAc) to give **56** (574 mg, 82%) as a colourless syrup. *R*_f_ = 0.57 (*n*-hexane/EtOAc 1:1); [α]_D_^24^ = −122.1 (*c* = 0.05 in CHCl_3_); ^1^H NMR (500 MHz, CDCl_3_): *δ* = 7.50–7.26 (m, 5H, Ar-H), 4.76 (d, *J*_1,2_ = 9.7 Hz, 1H, H-1), 4.21 (p, *J* = 7.2 Hz, 2H, SO_3_C*H*_2_CH_3_), 3.69 (t, *J* = 9.2 Hz, 1H, H-3), 3.60 (t, *J* = 9.6 Hz, 1H, H-2), 3.47 (t, *J* = 9.0 Hz, 1H, H-4), 3.40 (t, *J* = 9.0 Hz, 1H, H-5), 3.28, 3.23 (2 × s, 6H, 2 × BDA-OC*H*_3_), 3.22–3.18 (m, 1H, H-7a), 3.14–3.08 (m, 1H, H-7b), 2.64 (s, 1H, H-4-OH), 2.49–2.42 (m, 1H, H-6a), 2.02–1.96 (m, 1H, H-6b), 1.38–1.33 (m, 9H, SCH_2_C*H*_3_, 2 × BDA-C*H*_3_) ppm; ^13^C NMR (125 MHz, CDCl_3_): *δ* = 132.7 (1C, C_q_ Ar), 132.3, 129.0, 127.8 (5C, Ar), 100.2, 99.8 (2C, 2 × C_q_ BDA), 84.9 (1C, C-1), 77.6 (1C, C-5), 74.3 (1C, C-3), 71.0 (1C, C-4), 68.3 (1C, C-2), 66.3 (1C, SO_3_*C*H_2_CH_3_), 48.3, 48.1 (2C, 2 × BDA-O*C*H_3_), 46.4 (1C, C-7), 26.3 (1C, C-6), 17.7 (2C, 2 × BDA-*C*H_3_), 15.2 (1C, SO_3_CH_2_*C*H_3_) ppm; UHR ESI-QTOF (positive ion): *m*/*z* calcd for [C_21_H_32_O_9_S_2_ + Na]^+^: 515.1380; found: 515.1379.

Methyl 2,3-O-(2′,3′-dimethoxybutane-2′,3′-diyl)-6-deoxy-6-C-(ethylsulfonatomethyl)-4-O-(2-naphthyl)methyl-α-d-glucopyranoside (**58**). To a solution of **57** [45] (2.59 g, 5.780 mmol) in dry CH_2_Cl_2_ (16 mL), dry pyridine (1.6 mL) was added. The stirred mixture was cooled to −10 °C under argon and trifluoromethanesulfonic anhydride (1.36 mL, 8.09 mmol, 1.4 equiv.) dissolved in CH_2_Cl_2_ (3.5 mL) was added. After the complete disappearance of the starting material (30 min), the reaction mixture was diluted with CH_2_Cl_2_ (150 mL) and washed successively with H_2_O (2 × 25 mL), 1 M HCl (2 × 15 mL), H_2_O (2 × 25 mL), satd. aq. solution of NaHCO_3_ (2 × 15 mL) and H_2_O (15 mL). The organic phase was dried over MgSO_4_ and concentrated at 30 °C. The crude product was used for further reaction without purification. A solution of methanesulfonic acid ethyl ester (1.19 mL, 11.56 mmol, 2.0 equiv.) in dry THF (36 mL) was cooled to −80 °C under argon and 2.5 M *n*-BuLi (4.63 mL, 11.56 mmol, 2.0 equiv.) in *n*-hexane was added. After stirring at −78 °C for 30 min, a solution of the triflate (3.35 g, 5.78 mmol) in dry THF (18 mL) was added dropwise. The reaction mixture was stirred for 1.5 h while its temperature was raised to −20 °C. The stirred mixture was quenched by the addition of satd. aq. solution of NH_4_Cl (40 mL) and diluted with EtOAc (200 mL). The organic phase was washed successively with satd. aq. solution of NH_4_Cl (2 × 15 mL), H_2_O (2 × 20 mL), satd. aq. solution of NaCl (2 × 15 mL), dried over MgSO_4_ and concentrated. The crude product was purified by column chromatography (1:1 *n*-hexane/EtOAc) to give **58** (3.15 g, 98% for two steps) as a colourless syrup. *R*_f_ = 0.54 (*n*-hexane/EtOAc 1:1); [α]_D_^24^ = +32.8 (*c* = 0.25 in CHCl_3_); ^1^H NMR (400 MHz, CDCl_3_): *δ* = 7.84–7.46 (m, 7H, Ar-H), 5.15 (d, *J*_gem_ = 11.3 Hz, 1H, NpC*H*_2_a), 4.84 (d, *J*_gem_ = 11.3 Hz, 1H, NpC*H*_2_b), 4.68 (d, *J*_1,2_ = 3.5 Hz, 1H, H-1), 4.31–4.28 (m, 3H, SO_3_C*H*_2_CH_3_, H-3), 3.77 (dd, *J* = 10.3 Hz, *J* = 3.6 Hz, 1H, H-2), 3.71 (dt, *J* = 9.1 Hz, *J* = 2.9 Hz, 1H, H-5), 3.38 (t, *J* = 9.2 Hz, 1H, H-4), 3.37, 3.32, 3.28 (3 × s, 9H, C-1-OC*H*_3_, 2 × BDA-OC*H*_3_), 3.25–3.05 (m, 2H, H-7a,b), 2.42–2.40 (m, 1H, H-6a), 2.03–1.89 (m, 1H, H-6b), 1.42–1.28 (m, 9H, SCH_2_C*H*_3_, 2 × BDA-C*H*_3_) ppm; ^13^C NMR (100 MHz, CDCl_3_): *δ* = 135.5, 133.2, 133.0 (3C, C_q_ Ar), 128.3–126.0 (7C, Ar), 99.9, 99.4 (2C, 2 × C_q_ BDA), 97.7 (C-1), 78.4 (1C, C-4), 70.5 (1C, C-3), 68.7 (1C, C-5), 68.3 (1C, C-2), 74.9 (1C, Np*C*H_2_), 66.2 (1C, SO_3_*C*H_2_CH_3_), 55.1 (1C, C-1-O*C*H_3_), 48.0, 47.9 (2C, 2 × BDA-O*C*H_3_), 46.7 (1C, C-7), 26.0 (1C, C-6), 18.0, 17.7 (2C, 2 × BDA-*C*H_3_), 15.0 (1C, SO_3_CH_2_*C*H_3_) ppm; UHR ESI-QTOF (positive ion): *m*/*z* calcd for [C_27_H_38_O_10_S + Na]^+^: 577.2078; found: 577.2079.

Methyl 2,3-O-(2′,3′-dimethoxybutane-2′,3′-diyl)-6-deoxy-6-C-(ethylsulfonatomethyl)-α-d-glucopyranoside (**59**). Compound **58** (3.15 g, 5.680 mmol) was converted to **59** according to general method **B**. The crude product was purified by column chromatography (7:3 *n*-hexane/acetone) to give **59** (1.99 g, 85%) as a colourless syrup. *R*_f_ = 0.31 (*n*-hexane/acetone 7:3); [α]_D_^24^ = +92.4 (*c* = 0.45 in CHCl_3_); ^1^H NMR (400 MHz, CDCl_3_): *δ* = 4.70 (d, *J*_1,2_ = 3.5 Hz, 1H, H-1), 4.31 (q, *J* = 7.1 Hz, 2H, SO_3_C*H*_2_CH_3_), 3.97 (dd, *J* = 10.0 Hz, *J* = 9.5 Hz, 1H, H-3), 3.69 (dd, *J* = 10.4 Hz, *J* = 3.6 Hz, 1H, H-2), 3.66 (dt, *J* = 9.2 Hz, *J* = 3.0 Hz, 1H, H-5), 3.44 (t, *J* = 9.1 Hz, 1H, H-4), 3.40, 3.29, 3.26 (3 × s, 9H, C-1-OC*H*_3_, 2 × BDA-OC*H*_3_), 3.37–3.31 (m, 1H, H-7a), 3.21–3.14 (m, 1H, H-7a), 2.88 (d, *J* = 2.1 Hz, 1H, H-4-O*H*), 2.47–2.39 (m, 1H, H-6a), 2.05–1.96 (m, 1H, H-6b), 1.42 (t, *J* = 7.1 Hz, 3H, SCH_2_C*H*_3_), 1.34, 1.31 (2 × s, 6H, 3 × BDA-C*H*_3_) ppm; ^13^C NMR (100 MHz, CDCl_3_): *δ* = 99.9, 99.5 (2C, 2 × C_q_ BDA), 98.0 (1C, C-1), 71.5 (1C, C-4), 69.5 (1C, C-5), 69.2 (1C, C-3), 68.2 (1C, C-2), 66.2 (1C, SO_3_*C*H_2_CH_3_), 55.2 (1C, C-1-O*C*H_3_), 48.0, 47.9 (2C, 2 × BDA-O*C*H_3_), 46.7 (1C, C-7), 25.8 (1C, C-6), 17.7, 17.6 (2C, 2 × BDA-*C*H_3_), 15.1 (1C, SO_3_CH_2_*C*H_3_) ppm; UHR ESI-QTOF (positive ion): *m*/*z* calcd for [C_16_H_30_O_10_S + Na]^+^: 437.1452; found: 437.1453.

Methyl [2,3-O-(2′,3′-dimethoxybutane-2′,3′-diyl)-6-deoxy-6-C-(ethylsulfonatomethyl)-α-d-glucopyranosyl]-(1→4)-2,3-O-(2′,3′-dimethoxybutane-2′,3′-diyl)-6-deoxy-6-C-(ethylsulfonatomethyl)-α-d-glucopyranoside (**60**) and methyl [2,3-O-(2′,3′-dimethoxybutane-2′,3′-diyl)-6-deoxy-6-C-(ethylsulfonatomethyl)-α-d-glucopyranosyl]-(1→4)-[2,3-O-(2′,3′-dimethoxybutane-2′,3′-diyl)-6-deoxy-6-C-(ethylsulfonatomethyl)-α-d-glucopyranosyl]-(1→4)-2,3-O-(2′,3′-dimethoxybutane-2′,3′-diyl)-6-deoxy-6-C-(ethylsulfonatomethyl)-α-d-glucopyranoside (**61**).

To a solution of acceptor **59** (140 mg, 0.338 mmol) and donor **56** (500 mg, 1.015 mmol, 3.0 equiv.) in dry CH_2_Cl_2_ (18 mL), 4 Å molecular sieves (1.0 g) were added. The stirred mixture was cooled to −40 °C under argon. After 30 min at this temperature, a mixture of NIS (320 mg, 1.421 mmol, 1.4 equiv.) and TfOH (31 µL, 0.355 mmol) dissolved in THF (750 µL) was added and the reaction mixture was allowed to warm up to rt in 2.5 h. The reaction mixture was diluted with CH_2_Cl_2_ (200 mL), washed successively with 10% aq. solution of Na_2_S_2_O_3_ (2 × 25 mL), satd. aq. solution of NaHCO_3_ (2 × 25 mL) and H_2_O (2 × 25 mL), dried over MgSO_4_ and concentrated. The crude product was purified by column chromatography (1:1 *n*-hexane/EtOAc) to give **60** (78 mg, 29%) as a colourless syrup and **61** (107 mg, 27%) as a colourless syrup. (Higher oligomers were detected by MALDI-TOF MS, see Figure S14).

Data of disaccharide 60: *R*_f_ = 0.19 (*n*-hexane/EtOAc 1:1); [α]_D_^24^ = −14.6 (*c* = 0.11 in CHCl_3_); ^1^H NMR (400 MHz, CDCl_3_): *δ* = 5.58 (s, 1H, H-1′), 4.67 (s, 1H, H-1), 4.35–4.24 (m, 5H, 2 × SO_3_C*H*_2_CH_3_, H-3), 3.86–3.80 (m, 2H, H-3′, H-5), 3.74–3.56 (m, 4H, H-2, H-2′, H-4, H-5′), 3.43–3.40 (m, 1H, H-4′), 3.38, 3.36, 3.29, 3.26, 3.21 (5 × s, 15H, C-1-OC*H*_3_, 4 × BDA-OC*H*_3_), 3.23–3.19 (m, 3H, 2 × H-7a, H-7b), 3.13–3.06 (m, 1H, H-7b), 2.72–2.67 (m, 1H, H-4′-O*H*), 2.42–2.36 (m, 2H, 2 × H-6a), 2.05–1.87 (m, 2H, 2 × H-6b), 1.44–1.19 (m, 18H, 2 × SCH_2_C*H*_3_, 4 × BDA-C*H*_3_) ppm; ^13^C NMR (100 MHz, CDCl_3_): *δ* = 99.8, 99.7, 99.4, 99.1 (4C, 4 × C_q_ BDA), 97.8 (1C, C-1), 96.9 (1C, C-1′), 75.3 (1C, C-4), 71.4 (1C, C-4′), 70.5 (1C, C-5′), 69.8 (1C, C-3), 68.9 (1C, C-5), 68.3 (1C, C-2), 67.8 (1C, C-3′), 67.6 (1C, C-2′), 66.7, 66.3 (2C, 2 × SO_3_*C*H_2_CH_3_), 55.2 (1C, C-1-O*C*H_3_), 48.0, 47.9, 47.6 (4C, 4 × BDA-O*C*H_3_), 46.7, 46.3 (2C, 2 × C-7), 27.1, 26.2 (2C, 2 × C-6), 17.8, 17.7, 17.3 (4C, 4 × BDA-*C*H_3_), 15.1, 15.0 (2C, 2 × SO_3_CH_2_*C*H_3_) ppm; UHR ESI-QTOF (positive ion): *m*/*z* calcd for C_31_H_56_NaO_19_S_2_ [M + Na]^+^: 819.2749; found: 819.2749.

Compound **60** was also obtained from the fully protected disaccharide **72** (200 mg, 0.213 mmol) by removal of the NAP-protecting group according to general method B. The crude product was purified by column chromatography (1:1 *n*-hexane/EtOAc) to give **60** (153 mg, 90%) as a colourless syrup.

Data of trisaccharide 61: *R*_f_ = 0.39 (*n*-hexane/EtOAc 4:6); [α]_D_^24^ = −20.0 (*c* = 0.12 in CHCl_3_); ^1^H NMR (400 MHz, CDCl_3_): *δ* = 5.50–5.48 (m, 2H, H-1′, H-1″), 4.67 (d, *J*_1,2_ = 3.3 Hz, 1H, H-1), 4.36–4.29 (m, 6H, 3 × SO_3_C*H*_2_CH_3_), 4.26 (t, *J* = 9.8 Hz, 1H, H-3), 4.09 (t, *J* = 9.7 Hz, 1H, H-3′), 3.83–3.64 (m, 7H, H-2, H-2′, H-2″, H-3″, H-5, H-5′, H-5″), 3.58–3.52 (m, 2H, H-4, H-4′), 3.43–3.41 (m, 1H, H-4), 3.40, 3.37, 3.36, 3.27, 3.24, 3.21, 3.20 (7 × s, 21H, C-1-OC*H*_3_, 6 × BDA-OC*H*_3_), 3.33–3.29 (m, 3H, 3 × H-7a), 3.18–3.11 (m, 3H, 3 × H-7b), 2.49 (s, 1H, H-4″-O*H*), 2.42–2.37 (m, 3H, 3 × H-6a), 2.06–1.86 (m, 3H, 3 × H-6b), 1.47–1.17 (m, 27H, 3 × SCH_2_C*H*_3_, 6 × BDA-C*H*_3_) ppm; ^13^C NMR (100 MHz, CDCl_3_): *δ* = 99.8, 99.7, 99.3, 99.2, 99.1 (6C, 6 × C_q_ BDA), 97.8 (1C, C-1), 97.6 (1C, C-1′), 97.1 (1C, C-1″), 76.5 (1C, C-4′), 76.2 (1C, C-4), 71.8 (1C, C-4″), 70.7 (1C, C-5″), 69.5, 69.3 (2C, C-3, C-3′), 68.8, 68.7, 68.4, 67.9, 66.6 (6C, C-2, C-2′, C-2″, C-3″, C-5, C-5′), 67.1, 67.0, 66.6 (3C, 3 × SO_3_*C*H_2_CH_3_), 55.3 (1C, C-1-O*C*H_3_), 48.2, 48.1, 48.0, 47.8, 47.6 (6C, 6 × BDA-O*C*H_3_), 46.7, 46.2 (3C, 3 × C-7), 27.4, 26.4 (3C, 3 × C-6), 17.9, 17.8, 17.4, 17.3 (6C, 6 × BDA-*C*H_3_), 15.3, 15.2, 15.1 (3C, 3 × SO_3_CH_2_*C*H_3_) ppm; UHR ESI-QTOF (positive ion): *m*/*z* calcd for [C_46_H_82_O_28_S_3_ + Na]^+^: 1201.4047; found: 1201.4045.

Compound **61** was also obtained from the fully protected trisaccharide **74** (210 mg, 0.159 mmol) by removal of the NAP-protecting group according to general method B. The crude product was purified by column chromatography (4:6 *n*-hexane/EtOAc) to give **61** (137 mg, 73%) as a colourless syrup.

Methyl [2,3-O-(2′,3′-dimethoxybutane-2′,3′-diyl)-6-deoxy-6-C-(ethylsulfonatomethyl)-4-O-(2′-naphthyl)methyl-α-d-glucopyranosyl]-(1→4)-2,3-O-(2′,3′-dimethoxybutane-2′,3′-diyl)-6-deoxy-6-C-(ethylsulfonatomethyl)-α-d-glucopyranoside (**72**).

Method I: To a solution of acceptor **59** (200 mg, 0.483 mmol) and donor **55** (457 mg, 0.724 mmol, 1.5 equiv.) in dry CH_2_Cl_2_ (10 mL), 4 Å molecular sieves (1.0 g) were added. The stirred mixture was cooled to −40 °C under argon. After 30 min at this temperature, a mixture of NIS (244 mg, 1.086 mmol, 1.5 equiv.) and TfOH (19 µL, 0.216 mmol) dissolved in THF (400 µL) was added and the reaction mixture was allowed to warm up to −15 °C in 1.5 h. The reaction mixture was diluted with CH_2_Cl_2_ (150 mL), washed successively with 10% aq. solution of Na_2_S_2_O_3_ (2 × 20 mL), satd. aq. solution of NaHCO_3_ (2 × 20 mL) and H_2_O (2 × 20 mL), dried over MgSO_4_ and concentrated. The crude product was purified by column chromatography (9:1 CH_2_Cl_2_/EtOAc) to give **72** (215 mg, 48%) as a colourless syrup.

Method II: To a solution of acceptor **59** (100 mg, 0.241 mmol) and donor **55** (213 mg, 0.338 mmol, 1.4 equiv.) in dry CH_2_Cl_2_ (5.0 mL), 4 Å molecular sieves (0.5 g) and *sym*-collidine (5.0 µL, 0.041 mmol, 0.12 equiv.) were added. The stirred mixture was cooled to −40 °C under argon. After 30 min at this temperature, a mixture of NIS (114 mg, 0.507 mmol, 1.5 equiv.) dissolved in dry THF (200 µL) and AgOTf (20 mg, 0.081 mmol) dissolved in dry toluene (250 µL) were added and the reaction mixture was allowed to warm up to 0 °C in 1.5 h. The reaction mixture was diluted with CH_2_Cl_2_ (100 mL), washed successively with 10% aq. solution of Na_2_S_2_O_3_ (2 × 15 mL), satd. aq. solution of NaHCO_3_ (2 × 15 mL) and H_2_O (2 × 15 mL), dried over MgSO_4_ and concentrated. The crude product was purified by column chromatography (9:1 CH_2_Cl_2_/EtOAc) to give **72** (158 mg, 70%) as a colourless syrup. *R*_f_ = 0.55 (CH_2_Cl_2_/EtOAc 9:1); [α]_D_^24^ = −33.3 (*c* = 0.09 in CHCl_3_); ^1^H NMR (400 MHz, CDCl_3_): *δ* = 7.83–7.44 (m, 7H, Ar-H), 5.57 (d, *J*_1′,2′_ = 4.2 Hz, 1H, H-1′), 5.10 (d, *J*_gem_ = 11.3 Hz, 1H, NpC*H*_2_a), 4.83 (d, *J*_gem_ = 11.3 Hz, 1H, NpC*H*_2_b), 4.66 (d, *J*_1,2_ = 3.4 Hz, 1H, H-1), 4.27 (t, *J* = 9.9 Hz, 1H, H-3), 4.22–4.16 (m, 4H, 2 × SO_3_C*H*_2_CH_3_), 4.08 (t, *J* = 9.1 Hz, 1H, H-3′), 3.82 (td, *J* = 2.2 Hz, *J* = 9.7 Hz, 1H, H-5), 3.77–3.67 (m, 3H, H-2, H-2′, H-5′), 3.57 (t, *J* = 9.3 Hz, 1H, H-4), 3.37, 3.36, 3.32, 3.25, 3.23 (5 × s, 15H, C-1-OC*H*_3_, 4 × BDA-OC*H*_3_), 3.35–3.34 (m, 1H, H-4′), 3.21–3.16 (m, 2H, H-7a, H-7a’), 3.10–3.04 (m, 2H, H-7b, H-7b’), 2.42–2.35 (m, 2H, H-6a, H-6a’), 2.03–2.00 (m, 1H, H-6b’), 1.88–1.85 (m, 1H, H-6b), 1.36–1.17 (m, 18H, 2 × SCH_2_C*H*_3_, 4 × BDA-C*H*_3_) ppm; ^13^C NMR (100 MHz, CDCl_3_): *δ* = 135.5, 133.2, 133.0 (3C, C_q_ Ar), 128.1–125.9 (7C, Ar), 99.8, 99.6, 99.3, 99.1 (4C, 4 × C_q_ BDA), 97.7 (1C, C-1), 96.6 (1C, C-1′), 78.1 (1C, C-4′), 75.3 (1C, C-4), 74.8 (1C, Np*C*H_2_), 70.2 (1C, C-3′), 69.7 (2C, C-3, C-5′), 68.3 (C-2′), 67.7 (2C, C-2, C-5), 66.7, 66.1 (2C, 2 × SO_3_*C*H_2_CH_3_), 55.1 (1C, C-1-O*C*H_3_), 48.0, 47.6 (4C, 4 × BDA-O*C*H_3_), 46.7 (1C, C-7), 46.4 (1C, C-7′), 27.2 (1C, C-6), 26.2 (1C, C-6′), 18.0, 17.8, 17.6, 17.3 (4C, 4 × BDA-*C*H_3_), 15.0, 14.9 (2C, 2 × SO_3_CH_2_*C*H_3_) ppm; UHR ESI-QTOF (positive ion): *m*/*z* calcd for [C_42_H_64_O_19_S_2_ + Na]^+^: 959.3375; found: 959.3372.

Methyl (2,3,4-tri-O-sulfate-6-deoxy-6-C-sulfonatomethyl-α-d-glucopyranosyl-(1→4)-2,3-di-O-sulfate-6-deoxy-6-C-sulfonatomethyl-α-d-glucopyranoside heptasodium salt (**73**). To the solution of compound **53** (45 mg, 0.081 mmol) in dry DMF (2.0 mL) SO_3_·Et_3_N complex (367 mg, 2.025 mmol, 5 equiv./OH) was added and the reaction mixture was stirred at 50 °C for 24 h. The reaction was quenched with saturated aqueous solution of NaHCO_3_ (850 mg, 0.01 mmol), then, the solution was concentrated. The crude product was treated with Dowex ion-exchange resin (Na^+^ form) and purified by Sephadex G-25 column chromatography eluting with H_2_O to give **73** (68 mg, 78%) as a white solid. *R*_f_ = 0.35 (CH_2_Cl_2_/MeOH/H_2_O 6:7:1); [α]_D_^24^ = +8.7 (*c* = 0.15 in H_2_O); ^1^H NMR (400 MHz, D_2_O): *δ* = 5.44 (d, *J*_1′,2′_ = 4.7 Hz, 1H, H-1′), 5.04 (d, *J*_1,2_ = 5.7 Hz, 1H, H-1), 4.98 (t, *J* = 4.8 Hz, 1H, H-3′), 4.67–4.63 (m, 2H, H-2′, H-3), 4.36–4.33 (m, 2H, H-2, H-4′), 4.15–4.12 (m, 1H, H-5′), 3.86–3.82 (m, 1H, H-5), 3.76 (t, *J* = 9.9 Hz, 1H, H-4), 3.37 (s, 3H, C-1-O-C*H*_3_), 3.10–2.92 (m, 4H, H-7a,b, H-7‘a,b), 2.36–1.84 (m, 4H, H-6a,b, H-6′a,b) ppm; ^13^C NMR (100 MHz, D_2_O): *δ* = 96.7 (1C, C-1), 92.8 (1C, C-1′), 77.9 (1C, C-3), 76.1 (1C, C-4), 75.1 (2C, C-2, C-4′), 74.1 (1C, C-3′), 72.6 (1C, C-5′), 72.3 (1C, C-2′), 68.4 (1C, C-5), 55.1 (1C, C-1-O*C*H_3_), 47.2 (2C, C-7, C-7′), 26.6 (2C, C-6, C-6′) ppm; UHR ESI-QTOF (positive ion): *m*/*z* calcd for [C_15_H_21_Na_7_O_30_S_7_ + 2Na]^2+^: 555.8615; found: 555.8614.

Methyl [2,3-O-(2′,3′-dimethoxybutane-2′,3′-diyl)-6-deoxy-6-C-(ethylsulfonatomethyl)-4-O-(2′-naphthyl)methyl-α-d-glucopyranosyl]-(1→4)-[2,3-O-(2′,3′-dimethoxybutane-2′,3′-diyl)-6-deoxy-6-C-(ethylsulfonatomethyl)-α-d-glucopyranosyl]-(1→4)-2,3-O-(2′,3′-dimethoxybutane-2′,3′-diyl)-6-deoxy-6-C-(ethylsulfonatomethyl)-α-d-glucopyranoside (**74**). To a solution of acceptor **60** (230 mg, 0.289 mmol) and donor **55** [24] (274 mg, 0.433 mmol, 1.5 equiv.) in dry CH_2_Cl_2_ (5.0 mL), 4 Å molecular sieves (1.0 g) and *sym*-collidine (7.0 µL, 0.052 mmol, 0.12 equiv.) were added. The stirred mixture was cooled to −40 °C under argon. After 30 min at this temperature, a mixture of NIS (146 mg, 0.649 mmol, 1.5 equiv.) dissolved in dry THF (200 µL) and AgOTf (27 mg, 0.104 mmol, 0.24 equiv.) dissolved in dry toluene (200 µL) was added and the reaction mixture was allowed to warm up to 10 °C in 1.5 h. The reaction mixture was diluted with CH_2_Cl_2_ (150 mL), washed successively with 10% aq. solution of Na_2_S_2_O_3_ (2 × 20 mL), satd. aq. solution of NaHCO_3_ (2 × 20 mL) and H_2_O (2 × 20 mL), dried over MgSO_4_ and concentrated. The crude product was purified by column chromatography (1:1 *n*-hexane/EtOAc) to give **74** (226 mg, 60%) as a colourless syrup. *R*_f_ = 0.25 (*n*-hexane/EtOAc 1:1); [α]_D_^24^ = −14.0 (*c* = 0.10 in CHCl_3_); ^1^H NMR (400 MHz, CDCl_3_): *δ* = 7.83–7.43 (m, 7H, Ar-H), 5.50–5.48 (m, 2H, H-1′, H-1″), 5.10 (d, *J*_gem_ = 11.3 Hz, 1H, NpC*H*_2_a), 4.82 (d, *J*_gem_ = 11.3 Hz, 1H, NpC*H*_2_b), 4.66 (d, *J*_1,2_ = 3.3 Hz, 1H, H-1), 4.28–4.02 (m, 8H, 3 × SO_3_C*H*_2_CH_3_, H-3, H-3′), 3.80–3.70 (m, 6H, H-2, H-2′, H-2″, H-5, H-5′, H-5″), 3.57–3.51 (m, 3H, H-3″, H-4, H-4′), 3.38, 3.37, 3.27, 3.26, 3.23, 3.21 (6 × s, 21H, C-1-OC*H*_3_, 6 × BDA-OC*H*_3_), 3.36–3.34 (m, 1H, H-4), 3.33–3.31 (m, 3H, 3 × H-7a), 3.17–3.13 (m, 3H, 3 × H-7b), 2.41–2.38 (m, 3H, 3 × H-6a), 2.03–1.85 (m, 3H, 3 × H-6b), 1.44–1.17 (m, 27H, 3 × SCH_2_C*H*_3_, 6 × BDA-C*H*_3_) ppm; ^13^C NMR (100 MHz, CDCl_3_): *δ* = 135.6, 133.2, 133.0 (3C, C_q_ Ar), 128.1–125.9 (7C, Ar), 99.8, 99.7, 99.2, 99.1, 99.0 (6C, 6 × C_q_ BDA), 97.7 (1C, C-1), 97.2 (1C, C-1′), 97.0 (1C, C-1″), 78.5 (1C, C-4″), 76.5 (1C, C-4′), 76.1 (1C, C-4), 74.9 (1C, Np*C*H_2_), 69.9, 69.5, 69.3, 68.7, 68.3, 67.8 (9C, C-2, C-2′, C-2″, C-3, C-3′, C-3″, C-5, C-5′, C-5″), 67.9, 66.6 (3C, 3 × SO_3_*C*H_2_CH_3_), 55.2 (1C, C-1-O*C*H_3_), 48.2, 48.1, 48.0, 47.8, 47.6 (6C, 6 × BDA-O*C*H_3_), 46.6, 46.3, 46.0 (3C, 3 × C-7), 27.6, 27.4, 26.4 (3C, 3 × C-6), 17.9, 17.8, 17.7, 17.3, 17.2 (6C, 6 × BDA-*C*H_3_), 15.2, 15.0, 14.9 (3C, 3 × SO_3_CH_2_*C*H_3_) ppm; UHR ESI-QTOF (positive ion): *m*/*z* calcd for [C_57_H_90_O_28_S_3_ + 2Na]^2+^: 682.2283; found: 682.2292.

Methyl 2,3,4-tri-O-sulfate-6-deoxy-6-C-sulfonatomethyl-α-d-glucopyranosyl-(1→4)-2,3-di-O-sulfate-6-deoxy-6-C-sulfonatomethyl-α-d-glucopyranosyl-(1→4)-2,3-di-O-sulfate-6-deoxy-6-C-sulfonatomethyl-α-d-glucopyranoside decasodium salt (**75**). To the solution of compound **54** (20 mg, 0.024 mmol) in dry DMF (600 µL), SO_3_·Et_3_N complex (152 mg, 0.840 mmol, 5 equiv./OH) was added and the reaction mixture was stirred at 50 °C for 24 h. The reaction was quenched with saturated aqueous solution of NaHCO_3_ (850 mg, 0.01 mmol), then, the solution was concentrated. The crude product was treated with Dowex ion-exchange resin (Na^+^ form) and purified by Sephadex G-25 column chromatography eluting with H_2_O to give **75** (31 mg, 84%) as a white solid. *R*_f_ = 0.07 (CH_2_Cl_2_/MeOH/H_2_O 6:7:1); [α]_D_^24^ = +63.8 (*c* = 0.13 in H_2_O); ^1^H NMR (400 MHz, D_2_O): *δ* = 5.55 (s, 2H, H-1′, H-1″), 5.13 (d, *J*_1,2_ = 3.6 Hz, 1H, H-1), 4.99–4.97 (m, 1H, H-3′), 4.84–4.71 (m, 3H, H-2′, H-3, H-3″), 4.52 (dd, *J* = 8.6 Hz, *J* = 2.4 Hz, 1H, H-2″), 4.41 (dd, *J* = 9.7 Hz, *J* = 3.6 Hz, 1H, H-2), 4.31–4.24 (m, 2H, H-4″, H-5′), 3.99–3.91 (m, 3H, H-4′, H-5, H-5″), 3.84 (t, *J* = 9.0 Hz, 1H, H-4), 3.45 (s, 3H, C-1-O-C*H*_3_), 3.24–2.98 (m, 6H, H-7a,b, H-7‘a,b, H-7″a,b), 2.52–2.41 (m, 2H, H-6a, H-6″a), 2.28–2.18 (m, 2H, H-6′a,b), 2.11–1.90 (m, 2H, H-6b, H-6″b) ppm; ^13^C NMR (100 MHz, D_2_O): *δ* = 97.9 (1C, C-1), 94.9, 93.7 (2C, C-1′, C-1″), 79.2 (1C, C-3), 77.9 (1C, C-4′), 76.8 (2C, C-2, C-4), 76.3 (2C, C-3″, C-5″), 75.6 (1C, C-3′), 75.2 (1C, C-2′), 73.4 (1C, C-2″), 73.2 (1C, C-5′), 71.5 (1C, C-4″), 69.5 (1C, C-5), 56.2 (1C, C-1-O*C*H_3_), 48.3, 48.2, 48.0 (3C, C-7, C-7′, C-7″), 28.1, 27.7, 27.3 (3C, C-6, C-6′, C-6″) ppm; UHR ESI-QTOF (positive ion): *m*/*z* calcd for [C_22_H_30_Na_10_O_43_S_10_ + 2Na]^2+^: 788.8065; found: 788.8063.

## Figures and Tables

**Table 1:** Polymerization reaction of compound **8**.

Reaction	Promoter (Equiv.)	Concentration of 8 and Promoter	Time (h)	Temperature (°C)	Products [a]
1	NIS/TfOH (1.2/0.3)	**8**: 0.1 mM NIS: 0.11 mM	24	−50 to 10	**19**–**27**
2	NIS/TfOH (0.6/0.18)	**8**: 0.07 mM NIS: 0.04 mM	1.5	−50 to −10	**11**, **14**–**17** **19**–**22**
3	NIS/TfOH (0.6/0.18)	**8**: 0.07 mM NIS: 0.04 mM	24	−50 to 10	**11** (7%) [b] **14**–**18** **19**–**24**
4	NIS/TfOH (1.5/0.3)	**8**: 0.1 mM NIS: 0.15 mM	48	−50 to 10	**19**–**29**

[a] Detected by MALDI-TOF measurements. [b] Isolated yield.

**Table 2:** Polymerization of compound **8** in the presence of **30**.

Reaction	8:30 Ratio	Promoter (Equiv.)	Time (h)	Temperature (°C)	Products [a]
1	1.7:1	NIS/TfOH (1.2/0.12)	3.5	−50 to −10	**40**–**44**
2	2.2:1	2 x NIS/TfOH (1.2/0.12)	8	−50 to 10	**31** (37%) [b] **32** (16%) [b] **33**–**37**, **40**–**46**
3	3.3:1	2 x NIS/AgOTf (1.2/0.24)	32	−50 to 10	**31** (16%) [b] **32** (20%) [b] **33**–**37**, **40**–**46**
4	3:1	NIS/TfOH (1.2/0.12)	12.5	−50 to −10	**31** (33%) [b] **32** (25%) [b] **33**–**38**, **40**–**47**
5	10:1	NIS/TfOH (1.5/0.3)	48	−50 to 10	**33**–**39**, **40**–**50**

[a] Detected by MALDI-TOF measurements. [b] Isolated yield.

## Data Availability

Data are contained within the article and Supplementary Materials.

## Supplementary Materials

The following supporting information can be downloaded at: https://www.mdpi.com/article/10.3390/ijms25010677/s1.
