# Supplementary material for: Block Synthesis and Step-Growth Polymerization of C-6-Sulfonatomethyl-Containing Sulfated Malto-Oligosaccharides and Their Biological Profiling
Source: Int J Mol Sci. 2024 Jan 4;25(1):677. doi: 10.3390/ijms25010677 (PMC10779578; doi:10.3390/ijms25010677)
Supplement: Supplementary file 1 [file ijms-25-00677-s001.zip › ijms-2786353-supplementary.pdf]

Supporting Information

Block synthesis and step-growth polymerization of C-6-sulfonatomethyl-containing sulfated malto-oligosaccharides and their biological profiling

Mihály Herczeg,*[a] Fruzsina Demeter,[a] Tibor Nagy[b], Ágnes Rusznyák,[c],[d] Jan Hodek,[e] Éva Sipos,[c] István Lekli,[f] Ferenc Fenyvesi,[c] Jan Weber,[e] Sándor Kéki[b] and Anikó Borbás*[a],[g]

[a]Department of Pharmaceutical Chemistry, Faculty of Pharmacy, University of Debrecen, H-4032, Debrecen Egyetem tér 1, Hungary; [b]Department of Applied Chemistry, Faculty of Science and Technology, Institute of Chemistry, University of Debrecen, H-4032, Debrecen Egyetem tér 1, Hungary; [c]Department of Pharmaceutical Technology, Faculty of Pharmacy, University of Debrecen, H-4032, Debrecen Nagyerdei Körút 98, Hungary; [d]Institute of Healthcare Industry, University of Debrecen, Egyetem tér 1. H-4032 Debrecen, Hungary; [e]Institute of Organic Chemistry and Biochemistry, Czech Academy of Sciences, CZ-16000, Prague 6, Flemingovo nam. 2, Czech Republic; [f]Department of Pharmacodynamics, Faculty of Pharmacy, University of Debrecen, H-4032, Debrecen Nagyerdei Körút 98, Hungary; [g]HUN-REN-UD Molecular Recognition and Interaction Research Group, University of Debrecen, H-4032 Debrecen, Egyetem tér 1, Hungary

herczeg.mihaly@pharm.unideb.hu; borbas.aniko@pharm.unideb.hu

Table of Contents

Identification of the eliminated by-product **13** by MALDI-TOF MS 2

Analysis of polymerization reactions using MALDI-TOF MS methods 2

Biological evaluation 11

^{1}H and ^{13}C NMR spectra of the synthesized compounds 15

Identification of the eliminated by-product 13 by MALDI-TOF MS

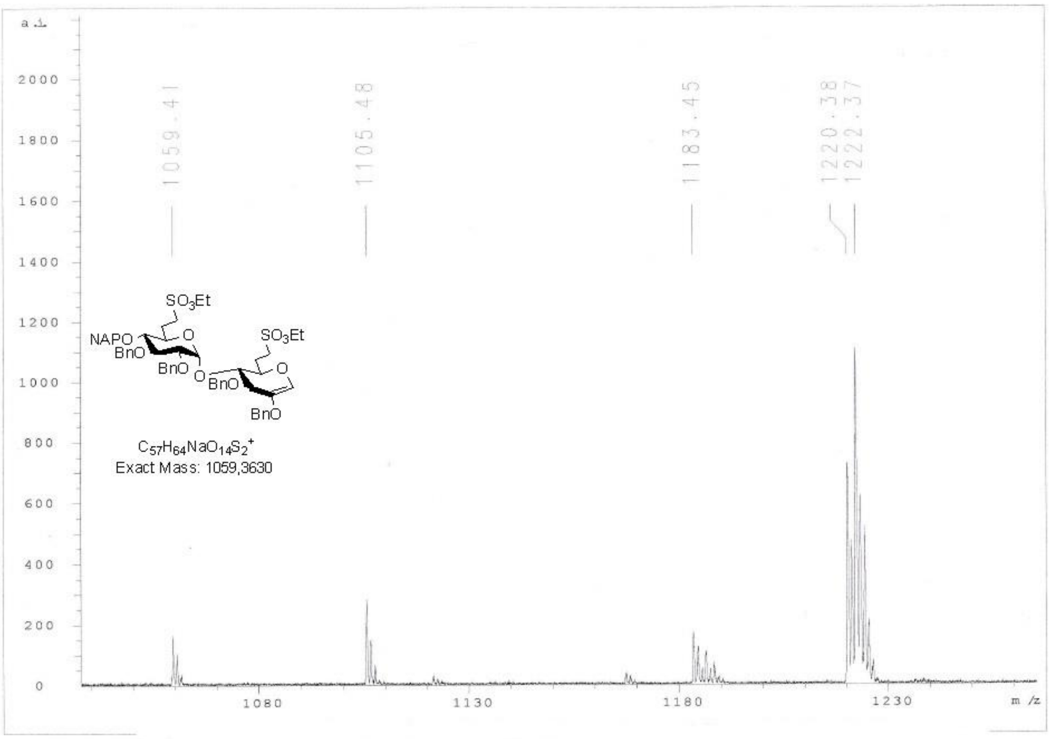

Figure S1. Partial MALDI-TOF MS spectrum of the reaction mixture obtained by glycosylation reaction of **11** and **10.** (MS spectrum of one of the fractions obtained after the first column chromatography purification).

Analysis of polymerization reactions using MALDI-TOF MS methods

Chemicals

Tetrahydrofuran (THF) (HiPerSolc Chromanorm, HPLC purity) and methanol (MeOH) (HiPerSolc Chromanorm, HPLC purity) were purchased from VWR International (Leuven, Belgium). Sodium trifluoroacetate and 2,5-dihydroxybenzoic acid (DHB) were received from Sigma-Aldrich (Taufkirchen, Germany).

Instrumental

Matrix-Assisted Laser Desorption/Ionization Time-of-Flight Mass Spectrometry (MALDI-TOF MS)

The MALDI-TOF MS measurements were performed with a Bruker BIFLEX III^{TM} mass spectrometer equipped with a time-of-flight (TOF) mass analyzer. In all cases 19 kV acceleration voltage was used with pulsed ion extraction (PIE^{TM}). The positive ions were detected in the reflectron mode (20 kV). A nitrogen laser (337 nm, 3 ns pulse width, 10^6-10^7 W/cm^2) operating at 4 Hz was used to produce laser desorption and 200 shots were summed. The MALDI-TOF MS spectra were externally calibrated using poly(ethylene glycol) with M_n=1450 g/mol. In order to observe the oligomers with higher masses, ions below m/z 1000 were deflected.

Samples for MALDI-TOF MS were prepared with 2,5-dihydroxy benzoic acid (DHB) matrix dissolved in tetrahydrofuran (THF) at a concentration of 20 mg/mL, analyte solutions at a concentration of 10 mg/mL in THF. Sodium trifluoroacetate dissolved in tetrahydrofuran at a concentration of 5 mg/mL (used as the cationization agent to promote ionization). The solutions were mixed in a 10:2:1 (v/v) ratio (matrix/analyte/cationization agent). A volume of 0.5 μL of the solution was deposited onto a metal sample plate and allowed to air-dry.

Electrospray Quadrupole Time-of-Flight MS (ESI-QTOF)

A MicroTOF-Q type Qq-TOF MS instrument (Bruker Daltonik, Bremen, Germany) was used for the MS and MS/MS measurements. The instrument was equipped with an electrospray ion source where the spray voltage was 4 kV and N_2 was used as drying gas. The drying temperature was 180 °C and the flow rate was 4.0 L/min. For the MS/MS experiments, nitrogen was used as the collision gas. The pressure in the collision cell was determined to be 1.2×10^{-2} mbar. The precursor ions for MS/MS were selected with an isolation width of 5 *m/z* units and collision energies in the range of 10-100 eV were applied for the collision-induced dissociation (CID) studies. The mass spectra were recorded by means of a digitizer at a sampling rate of 2 GHz. The mass spectra were calibrated externally using the exact masses of clusters $[(NaTFA)_n+TFA]^-$ generated from the electrosprayed solution of sodium trifluoroacetate (NaTFA). The spectra were evaluated with the DataAnalysis 3.4 software from Bruker. The sample solutions at a concentration of 0.01 mg/mL in methanol were introduced directly into the ESI source with a syringe pump (Cole-Parmer Ins. Co., Vernon Hills, IL, USA) at a flow rate of 3 µL/min.

Size-Exclusion Chromatography (SEC)

Size-Exclusion Chromatograms were recorded in THF at a flow rate of 0.5 mL/min at 35 °C with a Waters chromatograph equipped with four gel columns (4.6 x 300 mm, 4.6 x µm Styragel columns: HR 0.5, 1, 2 and 4), a Waters Alliance 2695 HPLC pump and with a Waters 2414 refractive index detector. The SEC was calibrated using low molecular weight polystyrene standards and narrow polydispersities (M_n = 500, 950, 1700, 2200 and 5050 g/mol).

MS spectra of polymerization reactions of monomer **8**

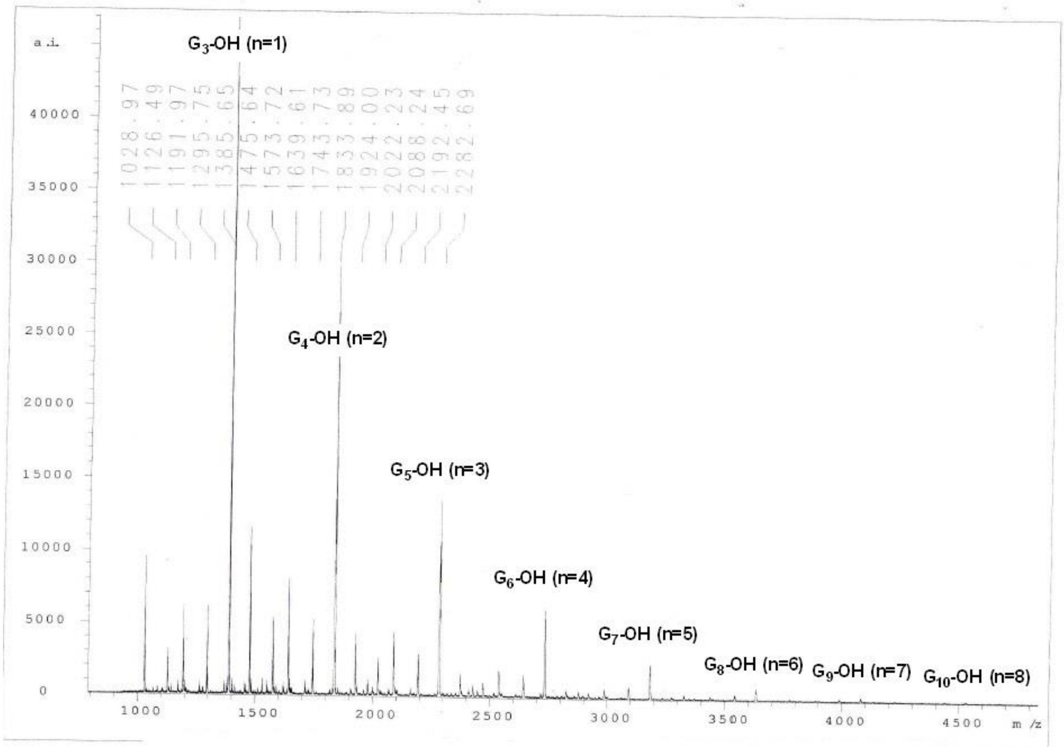

Figure S2. Partial MALDI-TOF MS spectrum of the reaction mixture obtained by oligomerization of **8** (Table 1, Reaction 1: NIS: 1.2 equiv, TfOH: 0.3 equiv.) in the m/z range of 1000-4500. The oligomer series appeared in the MALDI-TOF MS spectrum are cationized by sodium ions ($[M+Na]^+$).

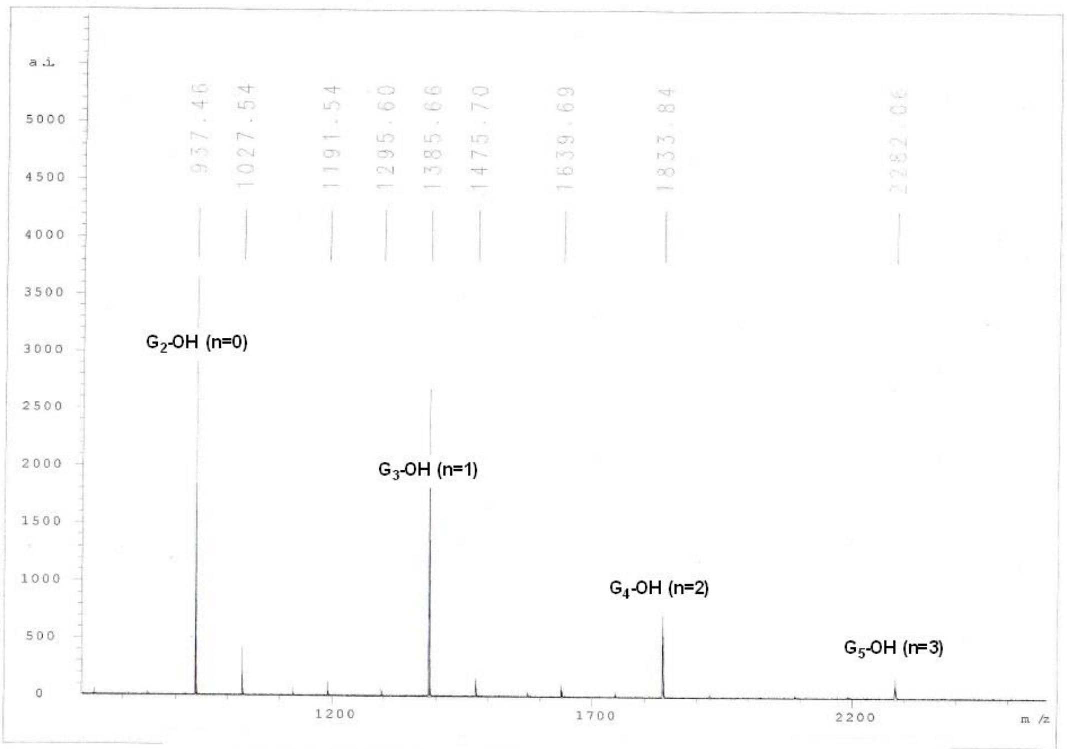

Figure S3. Partial MALDI-TOF MS spectrum of the reaction mixture obtained by oligomerization of **8** (Table 1, Reaction 1: NIS: 1.2 equiv, TfOH: 0.3 equiv.) in the m/z range of 1000-2200. The oligomer series appeared in the MALDI-TOF MS spectrum are cationized by sodium ions ($[M+Na]^+$).

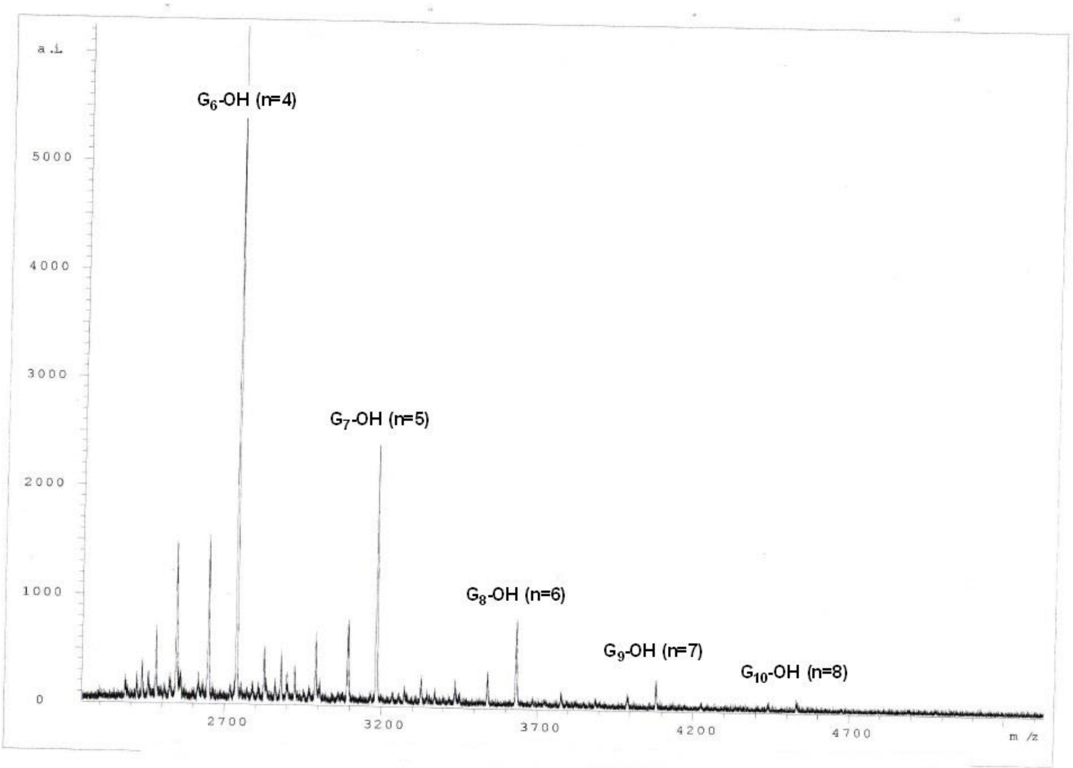

Figure S4. Partial MALDI-TOF MS spectrum of the reaction mixture obtained by oligomerization of **8** (Table 1, Reaction 1: NIS: 1.2 equiv, TfOH: 0.3 equiv.) in the m/z range of 2700-4700. The oligomer series appeared in the MALDI-TOF MS spectrum are cationized by sodium ions ($[M+Na]^+$).

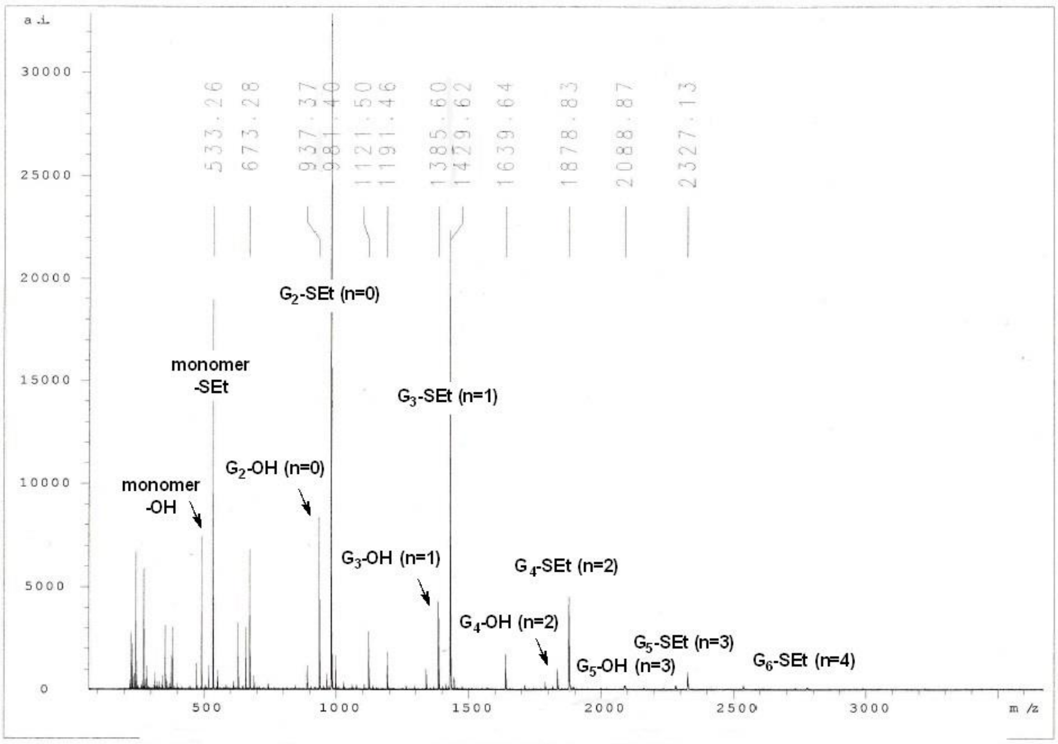

Figure S5. MALDI-TOF MS spectrum of the reaction mixture obtained by oligomerization of **8** (Table 1, Reaction 2: NIS: 0.6 equiv, TfOH: 0.3 equiv., 2 h reaction). The oligomer series appeared in the MALDI-TOF MS spectrum are cationized by sodium ions ($[M+Na]^+$).

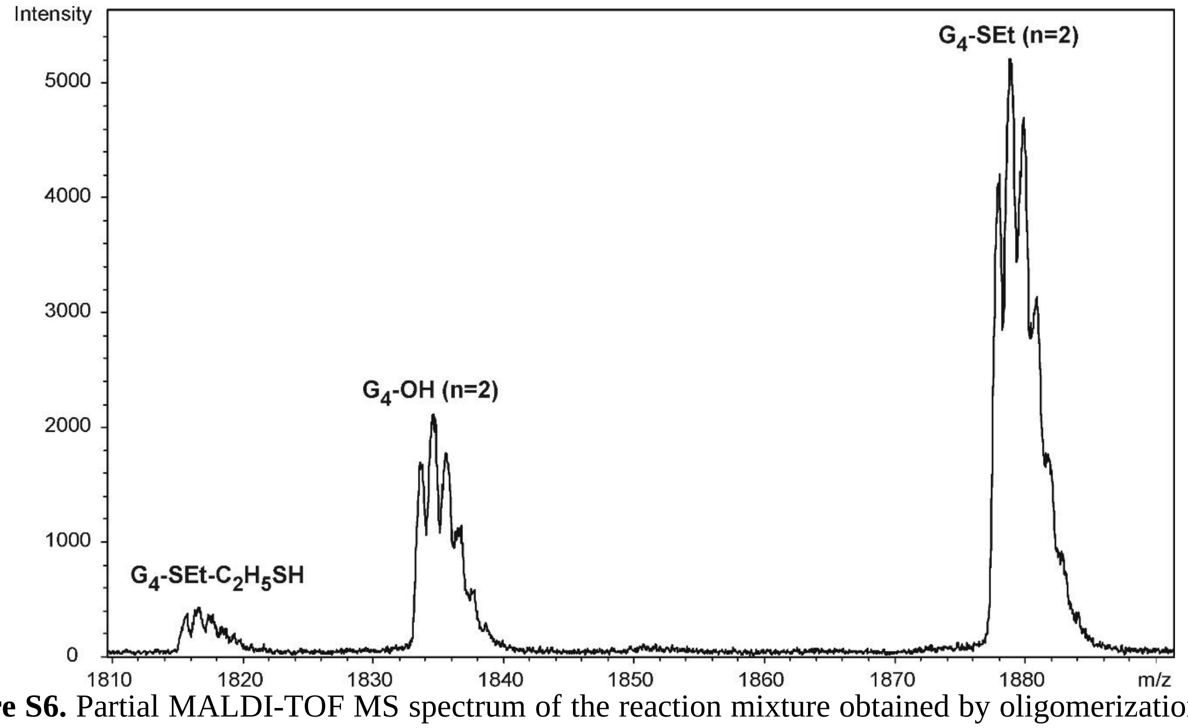

Figure S6. Partial MALDI-TOF MS spectrum of the reaction mixture obtained by oligomerization of **8** (Table 1, Reaction 3: NIS: 0.6 equiv, TfOH: 0.18 equiv., overnight reaction) in the m/z range of 1810-1860. The oligomer series appeared in the MALDI-TOF MS spectrum are cationized by sodium ions ($[M+Na]^+$).

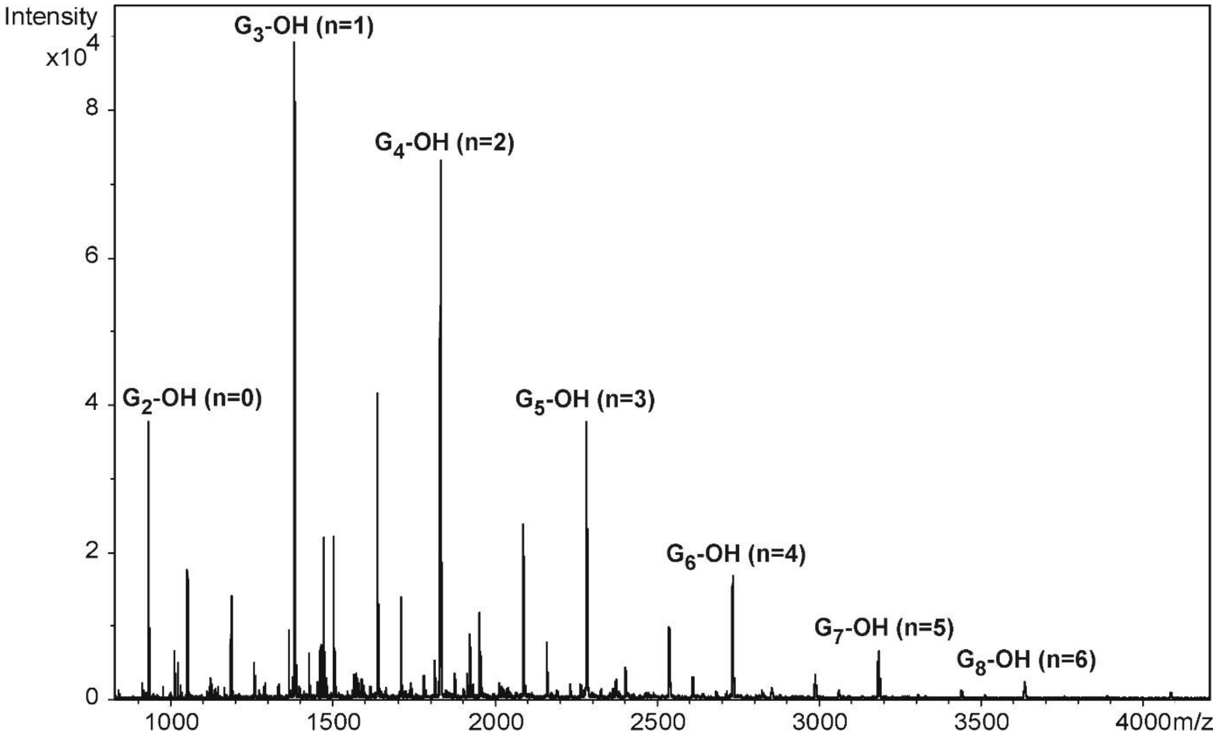

Figure S7. MALDI-TOF MS spectrum of the reaction mixture obtained by oligomerization reaction of **8** (Table 1, Reaction 4: NIS: 1.5 equiv, TfOH: 0.3 equiv.) in the m/z range of 1000-4000. The oligomer series appeared in the MALDI-TOF MS spectrum are cationized by sodium ion ($[M+Na]^+$).

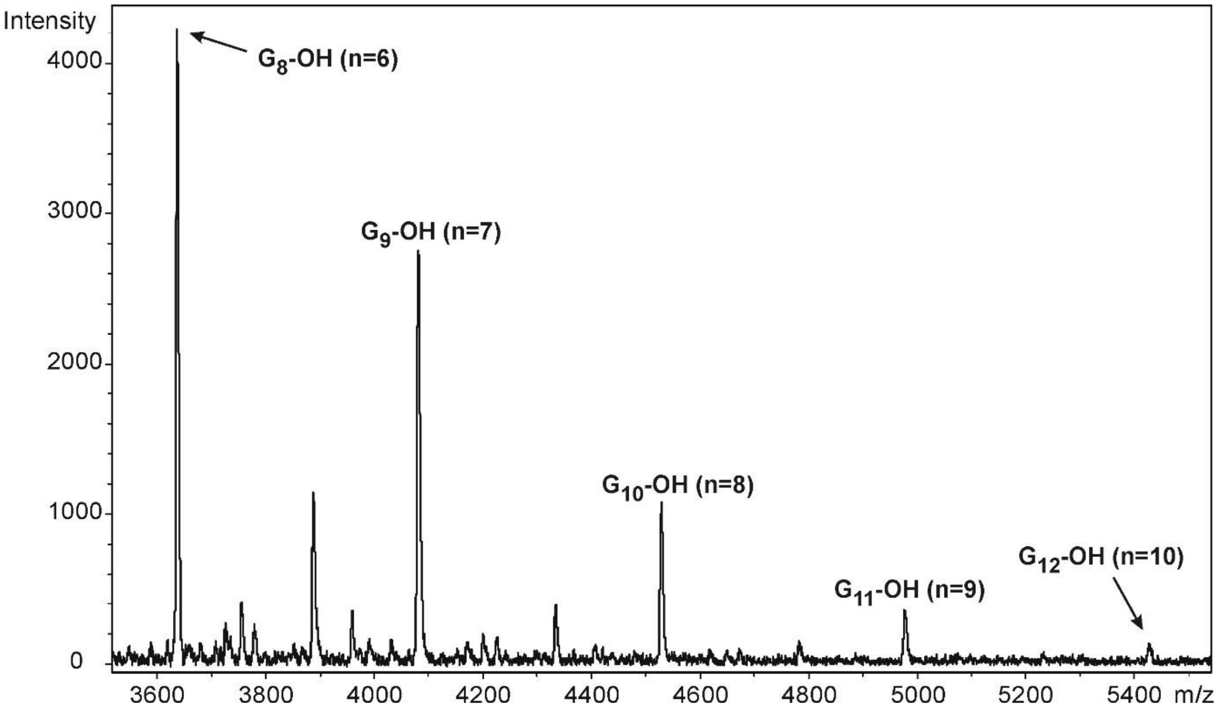

Figure S8. Partial MALDI-TOF MS spectrum of the reaction mixture obtained by oligomerization reaction of **8** (Table 1, Reaction 4: NIS: 1.5 equiv, TfOH: 0.3 equiv.) in the m/z range of 3600-5400. The oligomer series appeared in the MALDI-TOF MS spectrum are cationized by sodium ion ($[M+Na]^+$).

MS spectra of polymerization reactions of thioglycoside **8** in the presence of the capping unit **30**

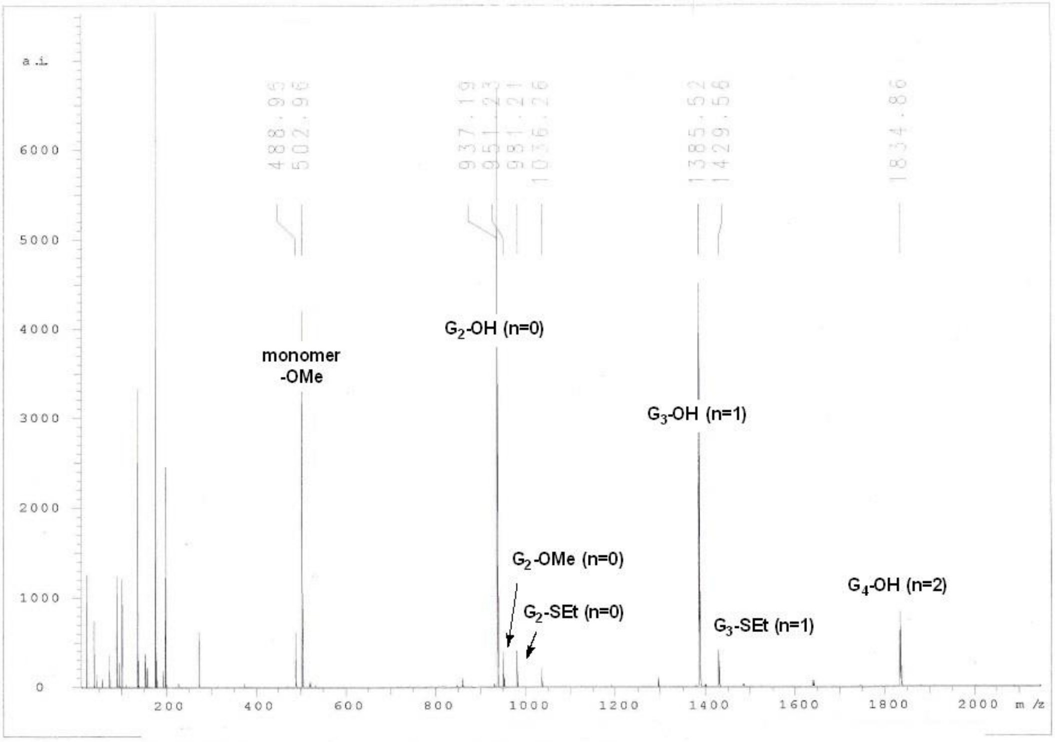

Figure S9. Partial MALDI-TOF MS spectrum of the reaction mixture obtained by oligomerization reaction of **8** in the presence of **30** (**8** and **30** in a1.7:1 ratio; preactivation method, NIS: 1.2 equiv, TfOH: 0.12 equiv.) in the m/z range of 200-2000. The oligomer series appeared in the MALDI-TOF MS spectrum are cationized by sodium ions ($[M+Na]^+$).

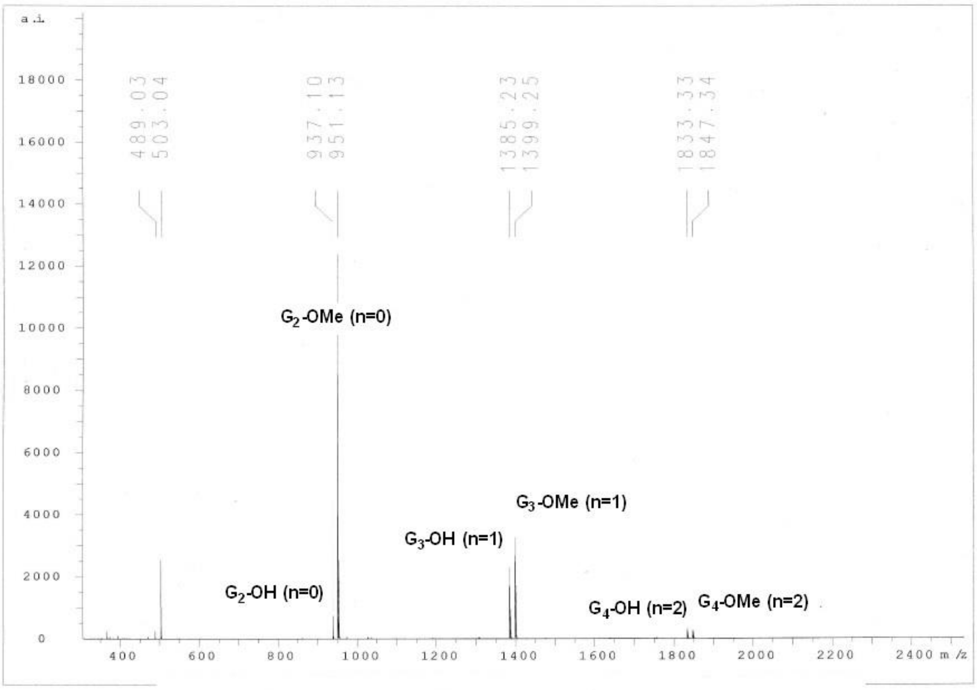

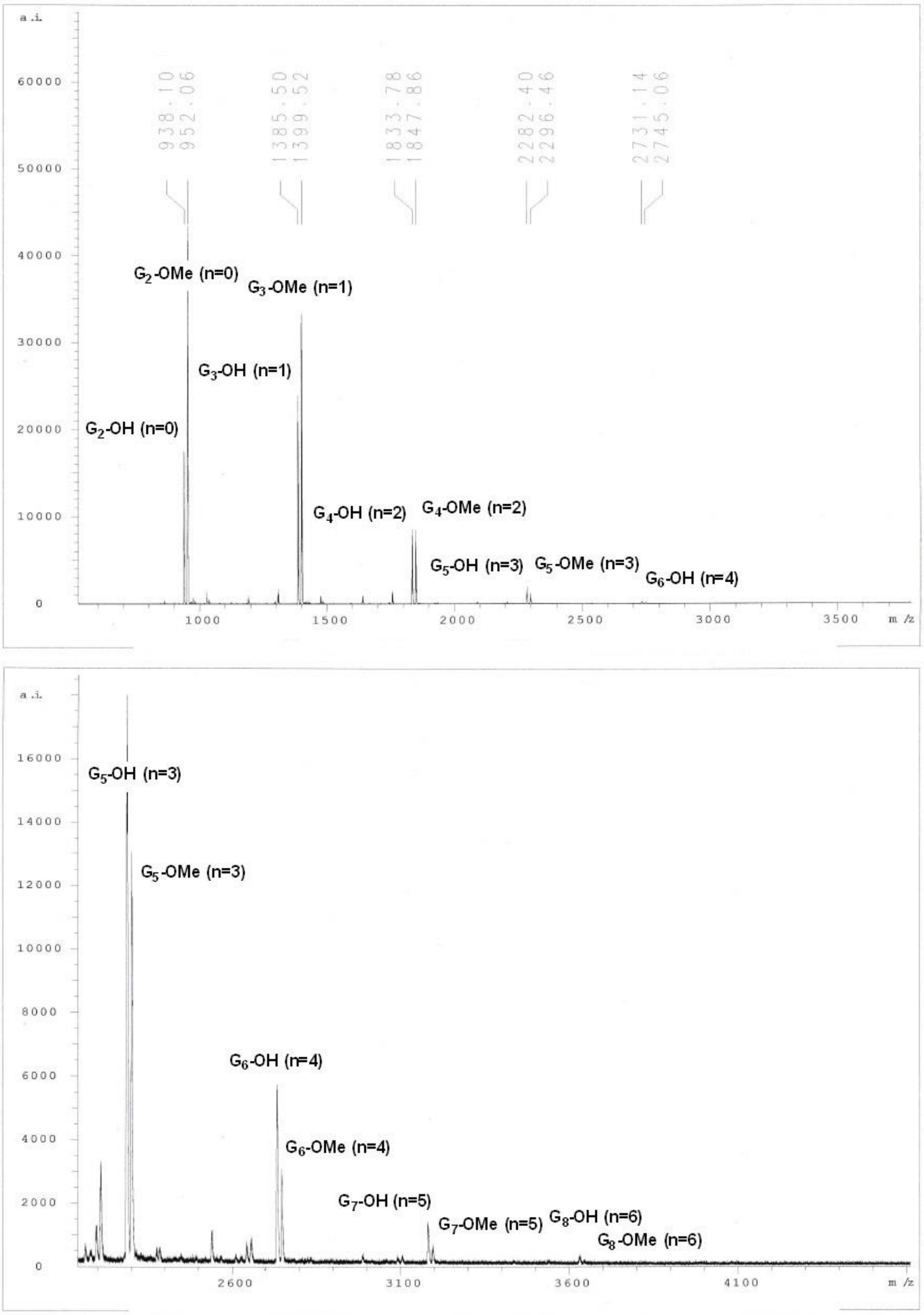

Figure S10. Partial MALDI-TOF MS spectra of the reaction mixture obtained by oligomerization reaction of **8** in the presence of **30** (**8** and **30** in a 2.2:1 ratio; NIS: 1.2 equiv, TfOH: 0.12 equiv.). The oligomer series appeared in the MALDI-TOF MS spectrum are cationized by sodium ions ($[M+Na]^+$).

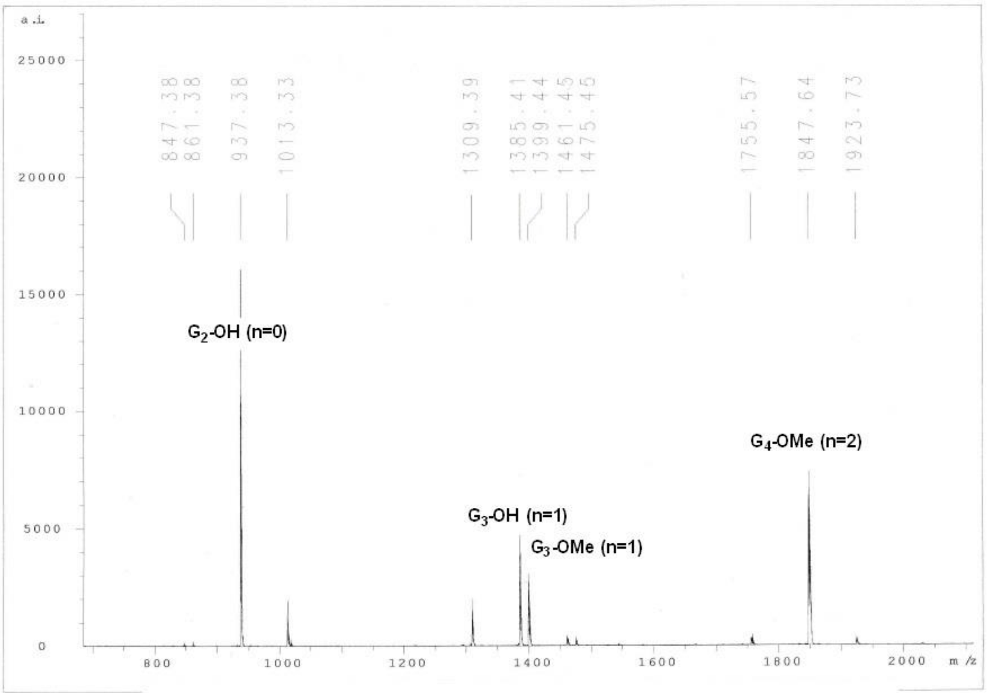

Figure S11. Partial MALDI-TOF MS spectrum of the reaction mixture obtained by oligomerization reaction of **8** in the presence of **30** (**8** and **30** in a 3.3:1 ratio, NIS: 1.2 equiv, AgOTf: 0.24 equiv.) in the m/z range of 800-2000. The oligomer series appeared in the MALDI-TOF MS spectrum are cationized by sodium ions ($[M+Na]^+$).

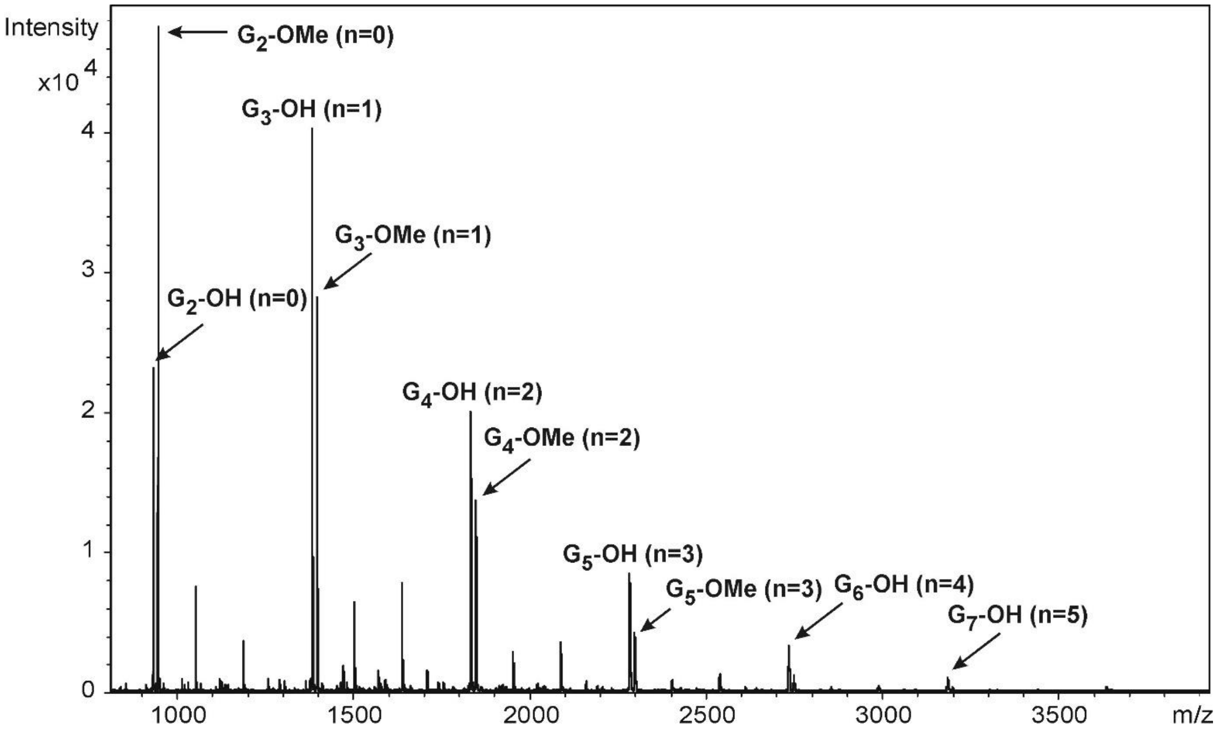

Figure S12. MALDI-TOF MS spectrum of the reaction mixture obtained by oligomerization reaction of **8** in the presence of **30** (**8** and **30** in a 10:1 ratio, NIS: 1.5 equiv, TfOH: 0.3 equiv.) in the m/z range of 1000-3500. The oligomer series appeared in the MALDI-TOF MS spectrum are cationized by sodium ions ($[M+Na]^+$).

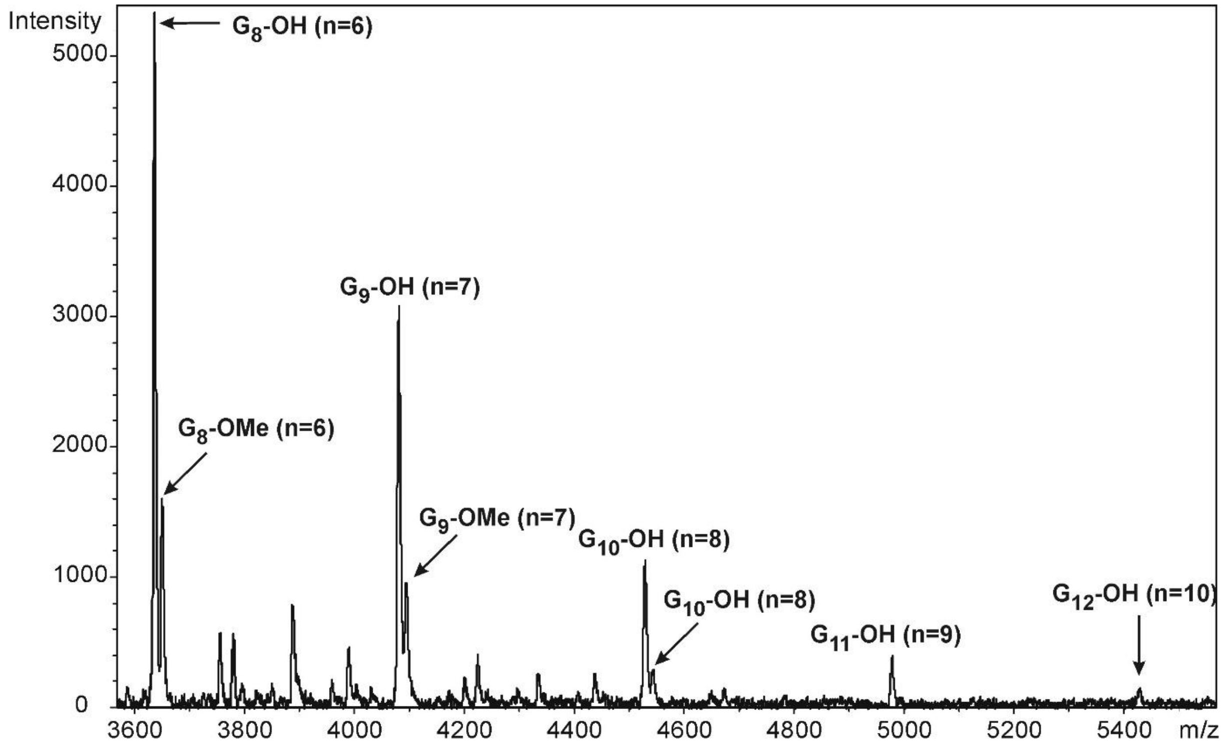

Figure S13. Partial MALDI-TOF MS spectrum of the reaction mixture obtained by oligomerization reaction of **8** in the presence of **30** (**8**:**30** in a 10:1 ratio, NIS: 1.5 equiv, TfOH: 0.3 equiv.) in the m/z range of 3600-5400. The oligomer series appeared in the MALDI-TOF MS spectrum are cationized by sodium ions ($[M+Na]^+$).

Oligomerization reaction of thioglycoside **56** in the presence of the capping unit **59**

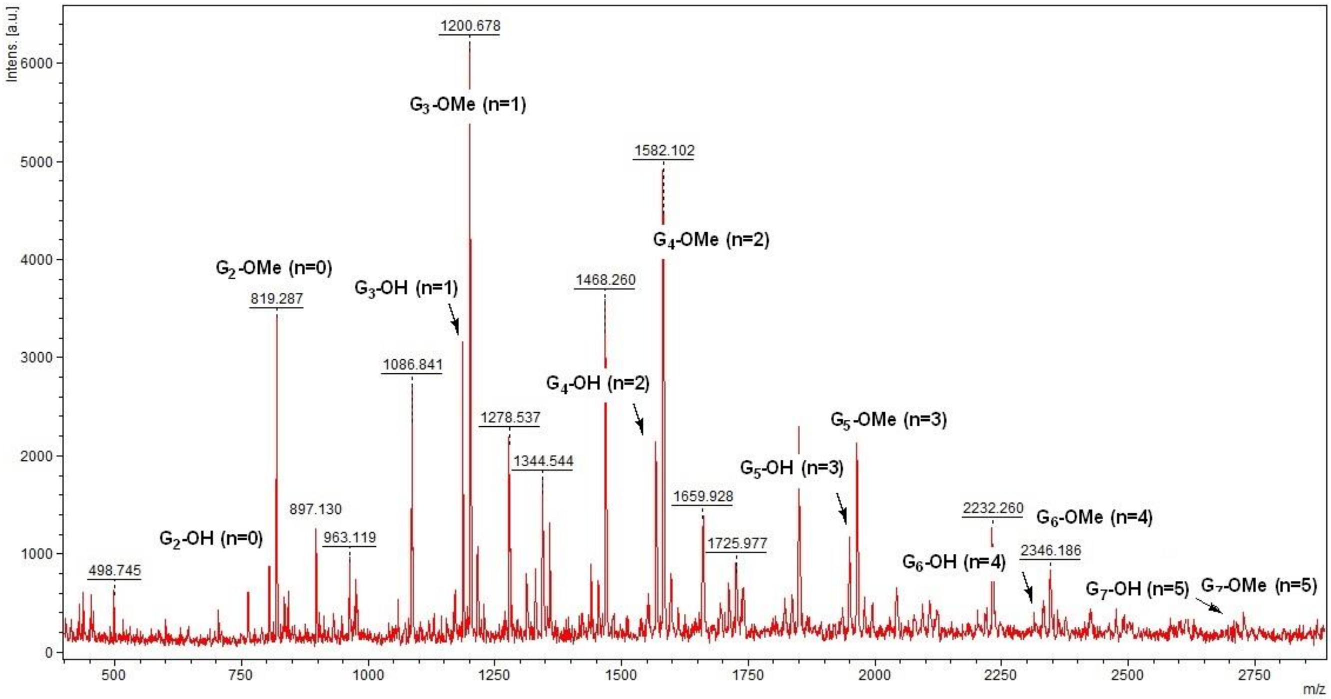

Figure S14. Partial MALDI-TOF MS spectrum of the reaction mixture obtained by oligomerization reaction of **56** in the presence of **59**. The oligomer series appeared in the MALDI-TOF MS spectrum are cationized by sodium ions ($[M+Na]^+$).

Biological evaluation

1. Anti-SARS-CoV-2 and cytotoxicity determination in Vero E6 cells

Anti-SARS-CoV-2 activity was measured by determining the extent the compounds inhibited virus-induced cytopathic effect (CPE) and viral replication assessed by immunofluorescence assay (IFA) in Vero E6 cells (ATCC). For CPE-based assay, two-fold serial dilutions of compounds were added in triplicate to Vero E6 cells that were seeded one day before in the amount of 20,000 cells in 96-well plate in DMEM medium with 10% FBS, 100 U of penicillin/mL and 100 µg of streptomycin/ml (all Merck). After 1 h incubation, SARS-CoV-2 (strain hCoV-19/Czech Republic/NRL_6632_2/2020) was added in the multiplicity of infection (MOI) 0.01 IU/cell and the cells were incubated for 72 hours at 37 ºC, 5% CO_2. After incubation, the cell viability was analyzed by formazan-based (XTT) cell proliferation assay. Briefly, mixture of XTT labelling reagent and PMS electron-coupling reagent was added to the cells and incubated for 4 h at 37 ºC, 5% CO_2. The absorbance of newly formed orange formazan dye was measured in EnVision (Perkin Elmer) at 450 nm. The compound concentrations resulting in 50% reduction of CPE (EC_{50}) were calculated from plots of percentage of absorbance versus $\log_{10}$ drug concentration using nonlinear regression using GraphPad Prism v.9.5.1 (GraphPad Software). For immunofluorescence based assay, Vero E6 cells and compounds were prepared same as above but SARS-CoV-2 was added at MOI 0.005 IU/cells. After three days incubation at 37 ºC, 5% CO_2, IFA was performed. Briefly, medium was removed and cells were fixed using 4% paraformaldehyde, permeabilized with 0.2% Triton-X100 (both Sigma-Aldrich), incubated with 1st mouse anti-SARS-CoV-2 antibody (mouse monoclonal nucleoprotein IgG, ProSci) for 2 h at room temperature followed by incubation with 2nd anti-mouse antibody conjugated with Cy-3 fluorophore (Jackson ImmunoResearch Europe) for 1.5 h. Signal was detected using fluorescent microscope with camera (Olympus). Images were processed in ImageJ program (NIH) and compound concentrations required to reduce fluorescence signal by 50 % (EC_{50}) were calculated from plots of percentage of fluorescent cells versus $\log_{10}$ drug concentration as above. For cytotoxicity determination the same setup as for CPE-based assay without the addition of virus. Vero E6 cytotoxicity was determined after 72 h incubation at 37 ºC, 5% CO_2 using XTT proliferation assay performed same as above. The compound concentrations resulting in 50% reduction in cell viability (CC_{50}) were calculated using nonlinear regression as above for EC_{50} determination. Remdesivir was used as a control in all experiments.

Table S1.: Antiviral activity of the synthesized compounds

**Compound**	EC_{50} CPE [µM]	95% CI of EC_{50} CPE	EC_{50} IFA [µM]	95% CI of EC_{50} IFA	CC_{50} [µM]	95% CI of CC_{50}
**53**	>50	n.a.	>50	n.a.	>50	n.a.
**54**	>50	n.a.	>50	n.a.	>50	n.a.
**73**	>50	n.a.	>50	n.a.	>50	n.a.
**75**	>50	n.a.	>50	n.a.	>50	n.a.
Remdesivir	4.1	3.6 – 4.7	1.6	1.4 – 1.7	>50	n.a.

CI confidence interval, CPE cytopathic effect-based assay, IFA immunofluorescence assay

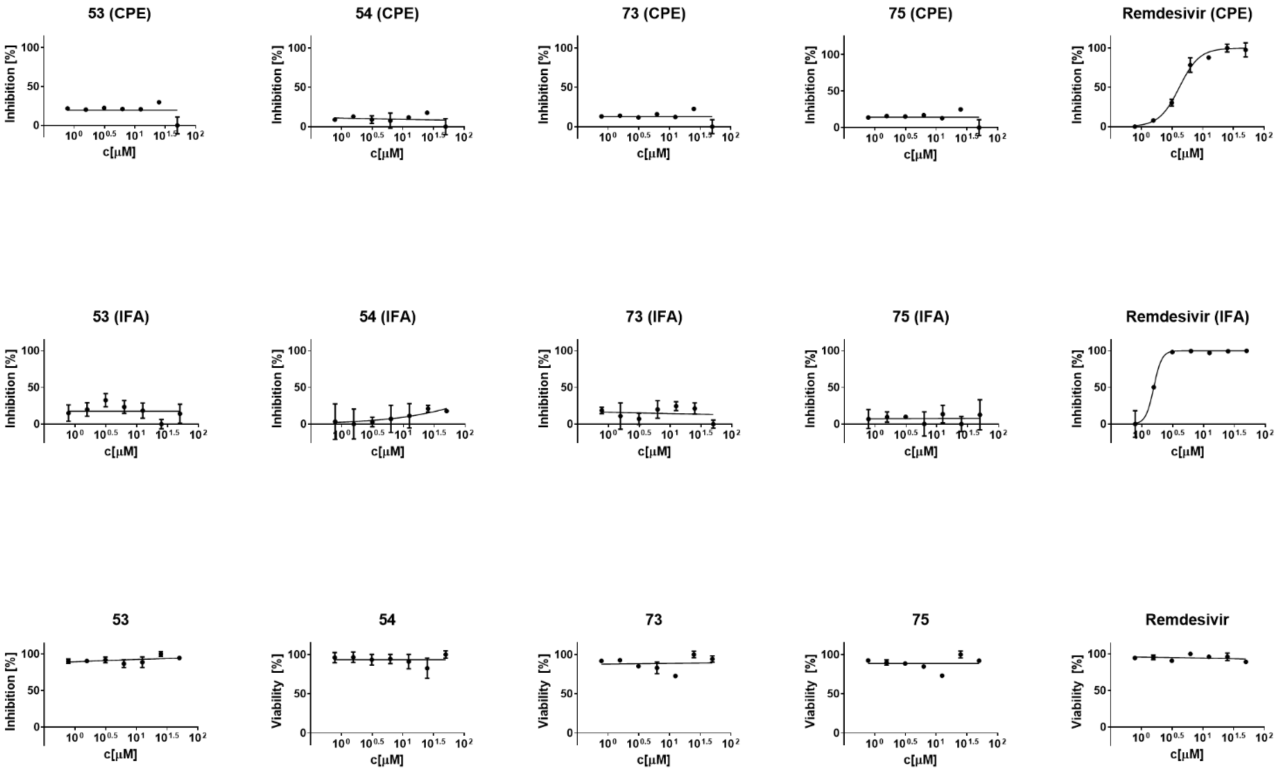

Figure S15. Anti-SARS-CoV-2 effect and cytotoxicity of compounds **53**, **54**, **73** and **75**. The antiviral activity of compounds was determined by cytopathic effect-based assay (upper panel) and by immunofluorescence assay (middle panel). The cytotoxicity of compounds in Vero E6 was determined by formazan-based cell viability assay (bottom panel). Remdesivir was used as a control.

2. Cytotoxicity studies

The immortalized hCMEC/D3 human endothelial cell line (Merck KGaA, Darmstadt, Germany, Cat. # SCC066) was cultured in Endothelial Cell Growth Medium MV (supplemented with the components of the Supplement Pack) (Sigma-Aldrich Ltd., Budapest, Hungary).

Human Caco-2 intestinal epithelial cell line and HeLa cell line were obtained from European Collection of Cell Cultures (ECACC, UK) and grown in Dulbecco's Minimum Essential Medium (DMEM), supplemented with 10% fetal bovine serum (FBS), 1% non-essential amino acid and 1% penicillin-streptomycin solution, and kept in an incubator with 5% CO_2 atmosphere.

H9c2 embryonic rat heart-derived (ventricular) cells (myoblasts) from ATCC were cultured in Dulbecco's modified Eagle's medium (DMEM) supplemented with 10% fetal bovine serum (FBS) under 95% air/5% CO_2 and subcultured at 50-60% confluence.

MCF-7 human breast cancer cell lines from ATCC were cultured in Dulbecco's modified Eagle's medium (DMEM) supplemented with L-glutamine, 10% FBS, and 1% penicillin/streptomycin in a humidified chamber at 37 ºC with 5% CO_2. Cells were subcultured every 3 days using a standard trypsinization procedure.

In cell viability experiments 1×10^4 hCMEC/D3, Caco-2, HeLa cells/well, or $0{,}5 \times 10^4$ H9c2 and MCF-7 were seeded on 96-well plates. Cells were incubated for 24 hours, and treated with the solutions of test compounds **53**, **54**, **73** and **75** in different concentrations ranging from 0.125 to 200 µM (hCMEC/D3, Caco-2, HeLa) or from 1 to 300 µM (H9c2, MCF-7). The control group received cell culture medium. Cells were incubated with the test solutions for 72 hours (hCMEC/D3, Caco-2, HeLa) or 24 hours (H9c2, MCF-7) at 37˚C in an incubator with 5% CO_2, and test solutions were replaced with 0.05 mg/ml 3-(4,5-dimethylthiazol-2-yl)-2,5-diphenyltetrazolium bromide (MTT) solutions prepared in PBS. Cells were incubated with MTT solutions for 4 hours at 37˚C and the formed dark blue formazan crystals were dissolved in acidic isopropanol (isopropanol : 1.0 N hydrochloric acid = 25 : 1). The absorbance was

measured at 570 nm using a 690 nm reference wavelength with a Thermo Fisher Multiskan Go microplate reader (Thermo Fisher, Waltham, MA, USA). Cell viability was expressed as the percentage of the untreated control and IC50 values were calculated by GraphPad Prism 9 software (GraphPad Software Inc., San Diego, CA, USA).

The test compounds had a dose-dependent cytotoxicity on hCMEC/D3, Caco-2 and HeLa cells, and the IC_{50} values were higher, than 50 µM of each compound (**Figure S16**).

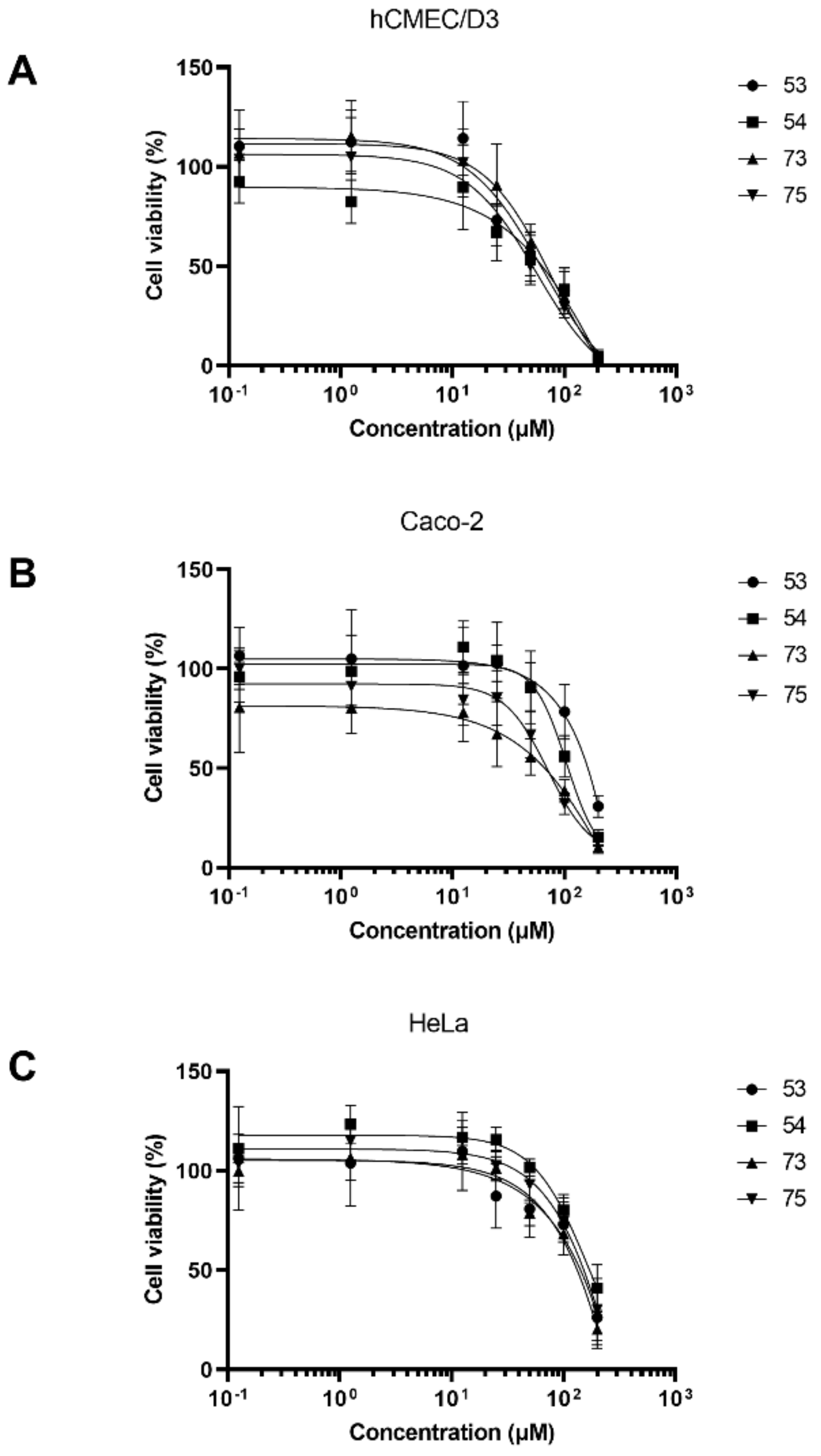

Figure S16. Cyotoxicity of test compounds **53**, **54**, **73** and **75** on hCMEC/D3 (**A**), Caco-2 (**B**) and HeLa (**C**) cells. Cells were incubated for 72 hours with the test solutions, the cell viability was determined by MTT-test and compared to the untreated control cells (100 %).

Short term cytotoxicity test indicated a dose-dependent decrement in cell viability, which were more pronounced in H9c2 cell line. However, after 24 h treatment even in the highest applied concentration (300 µM) the cell viability was around 50 % in each treated group (**Figure S17**).

A

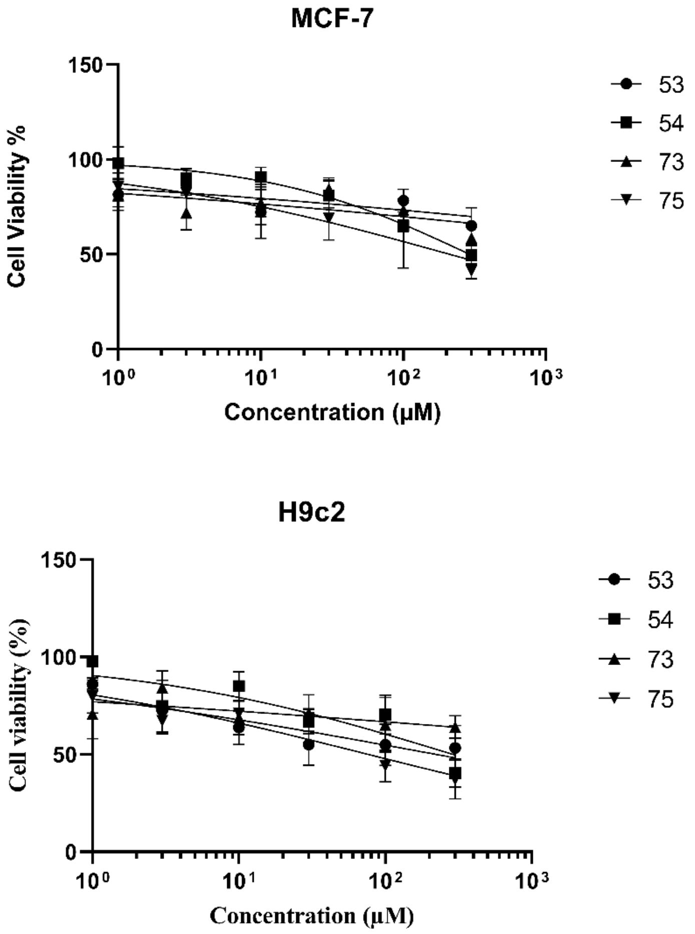

Figure S17. Effect of compounds **53, 54, 73** and **75** on the viability of cancerous and non-cancerous cell lines. A) H9c2 cardiomyocytes and B) MCF-7 human breast cancer cell lines. Viability was determined by MTT assay after 24 h of treatment with the compounds applied in the indicated concentrations and compared to the untreated control cells (100 %).

^{1}H and ^{13}C NMR spectra of the synthesized compounds

^{1}H spectrum of compound 5αβ

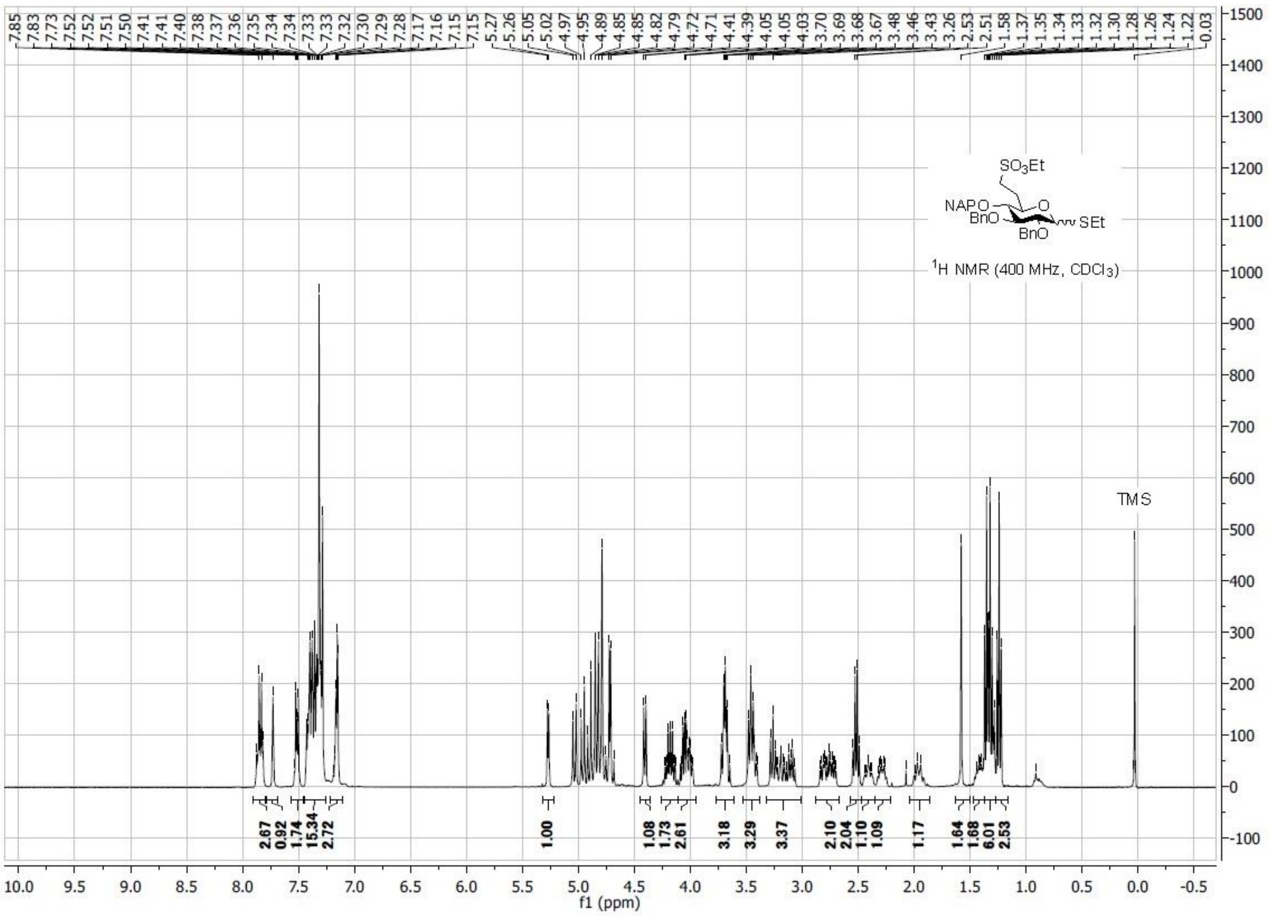

^{1}H and ^{13}C NMR spectra of compound 8

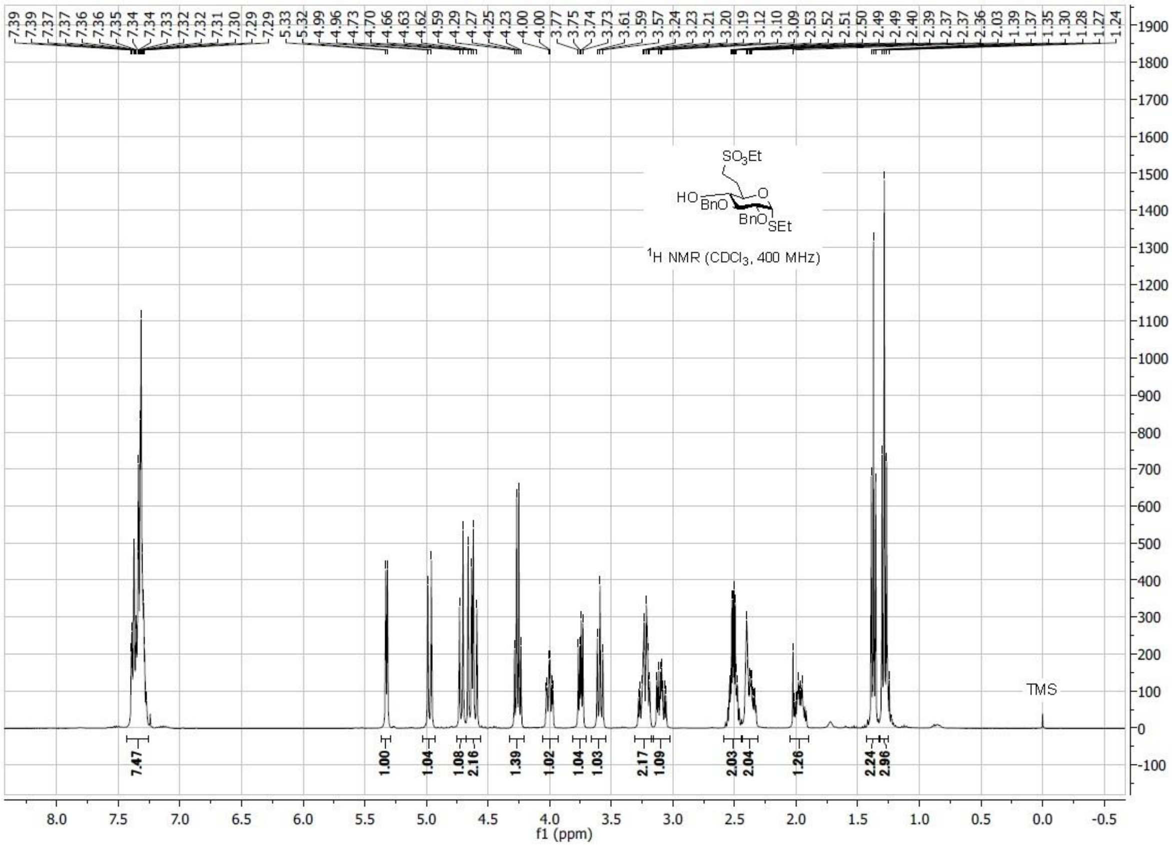

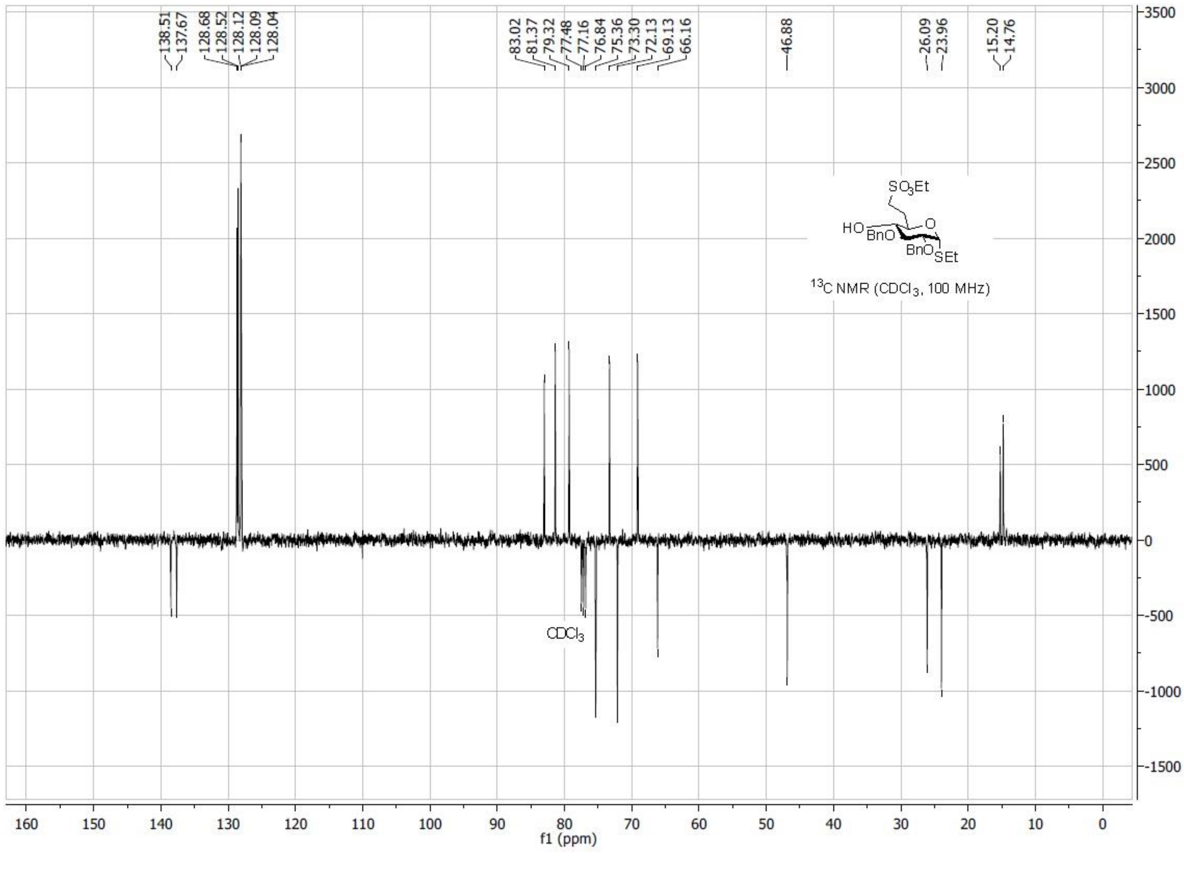

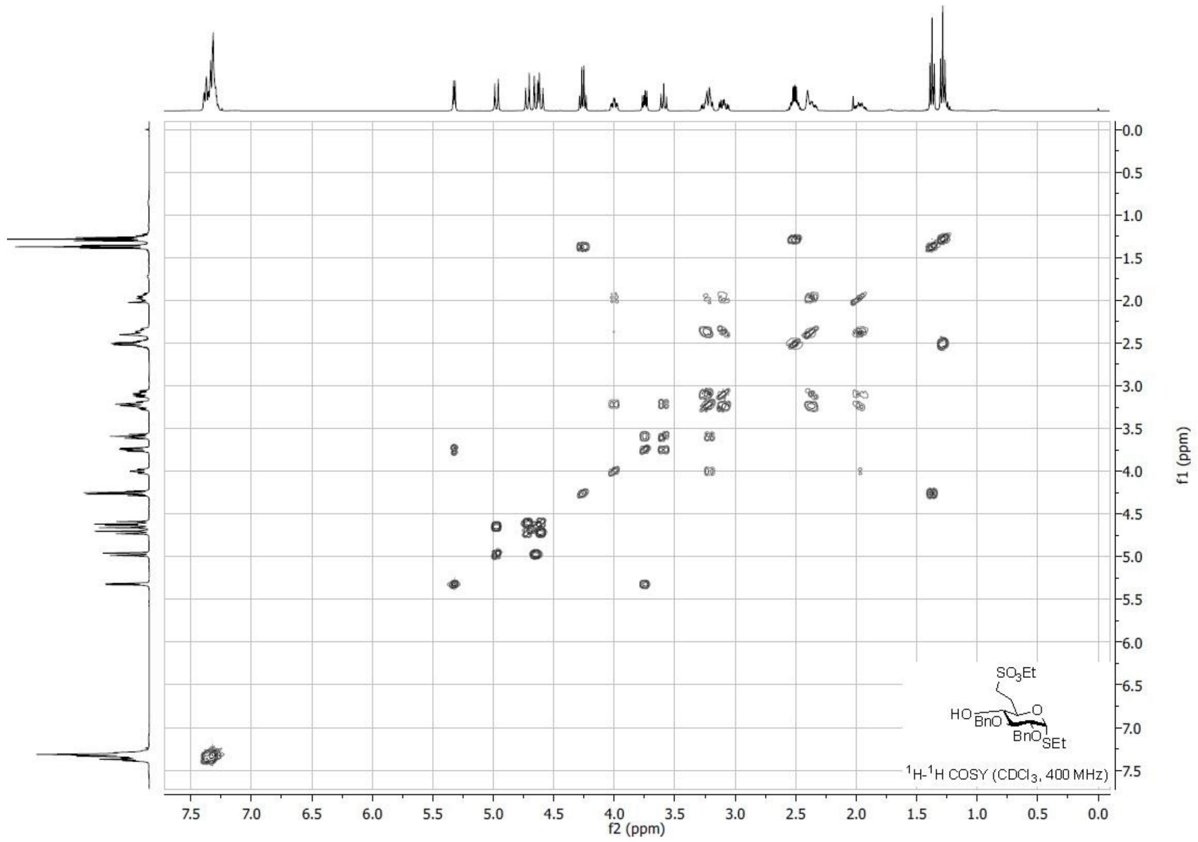
SO3Et
HO
BnO
O
BnO
SEt
1H-1H COSY (CDCl3, 400 MHz)
f2 (ppm)
f1 (ppm)

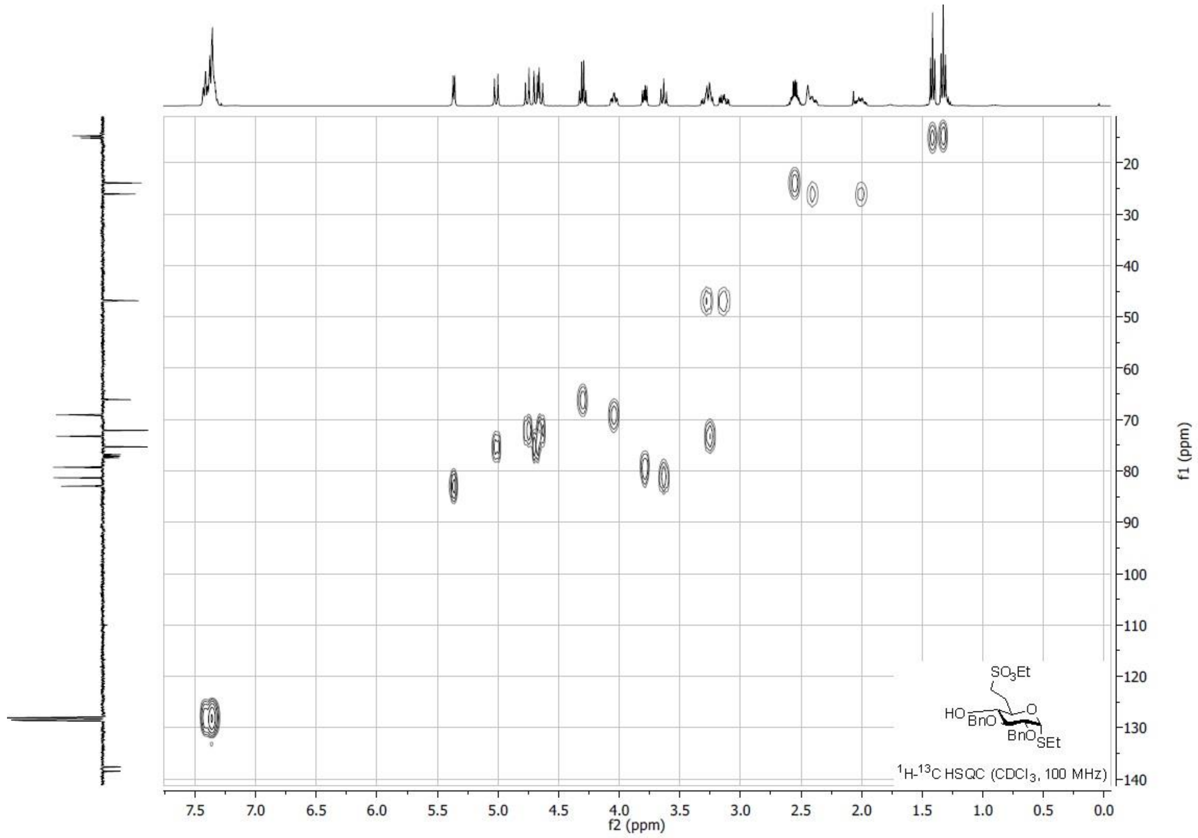
SO3Et
HO
BnO
O
BnO
SEt
1H-13C HSQC (CDCl3, 100 MHz)
f2 (ppm)
f1 (ppm)

^{1}H and ^{13}C NMR spectra of compound 9

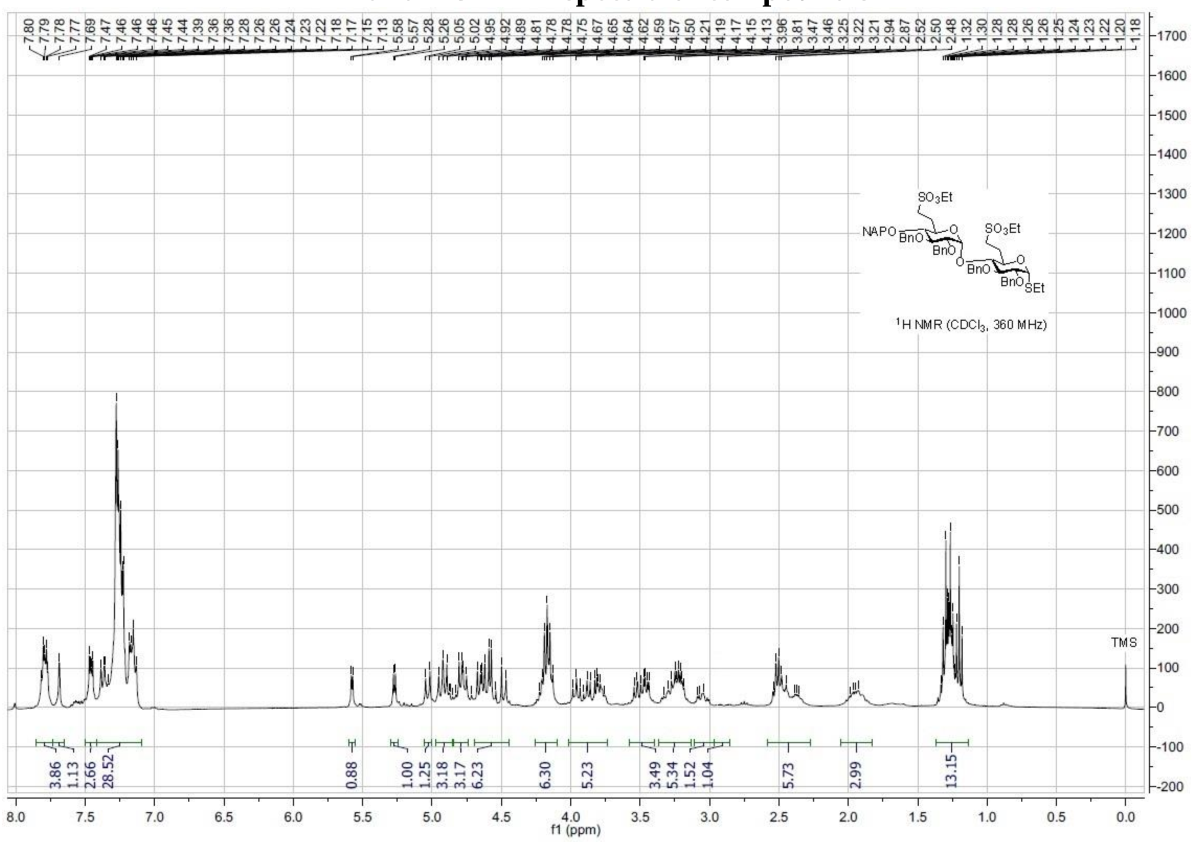

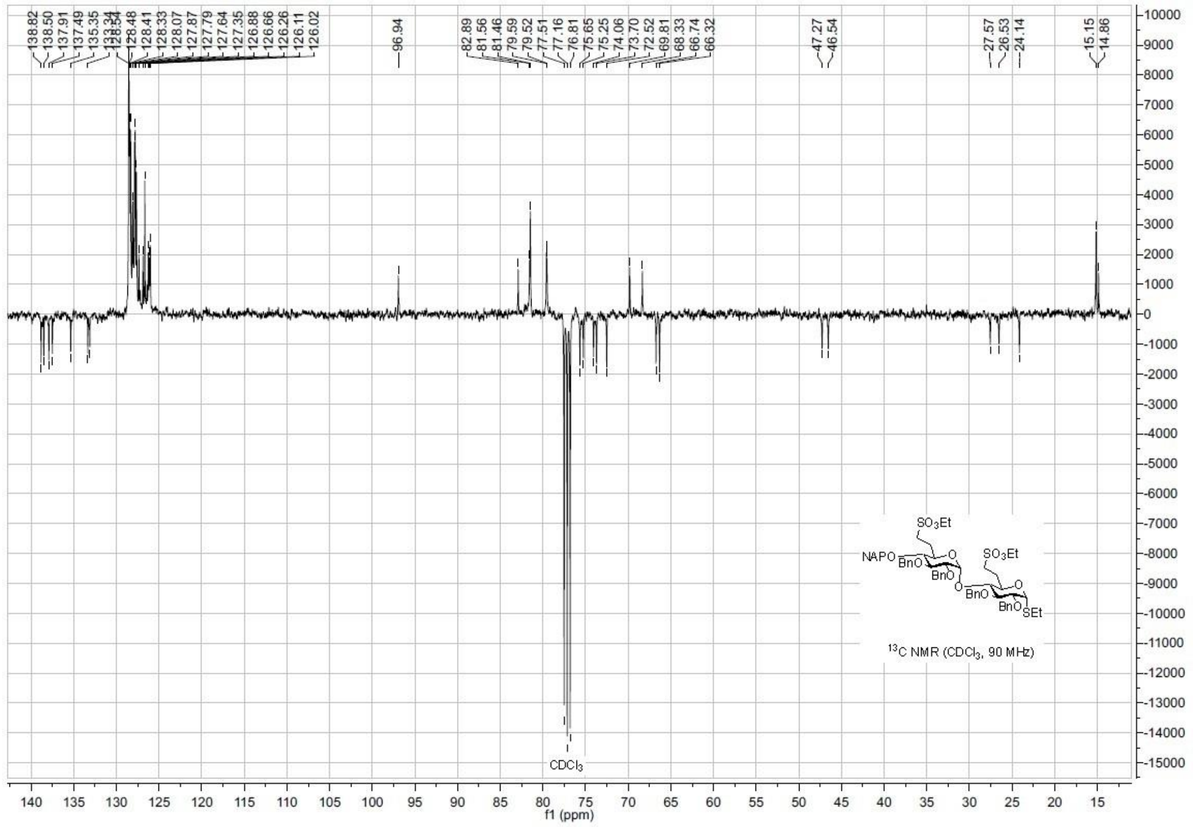

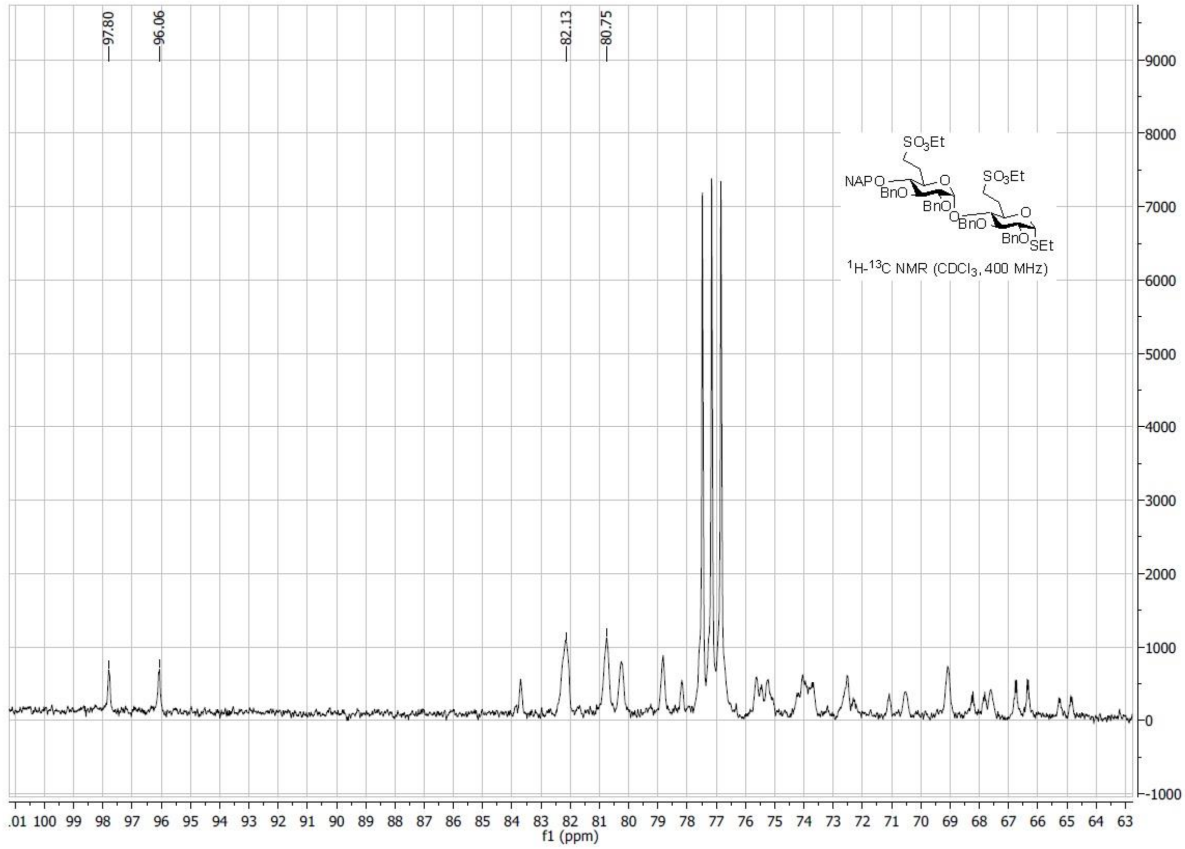

97.80
96.06
82.13
80.75
SO_3Et
NAPO
BnO
BnO
BnO
BnO
SEt
1H-13C NMR (CDCl3, 400 MHz)
f1 (ppm)

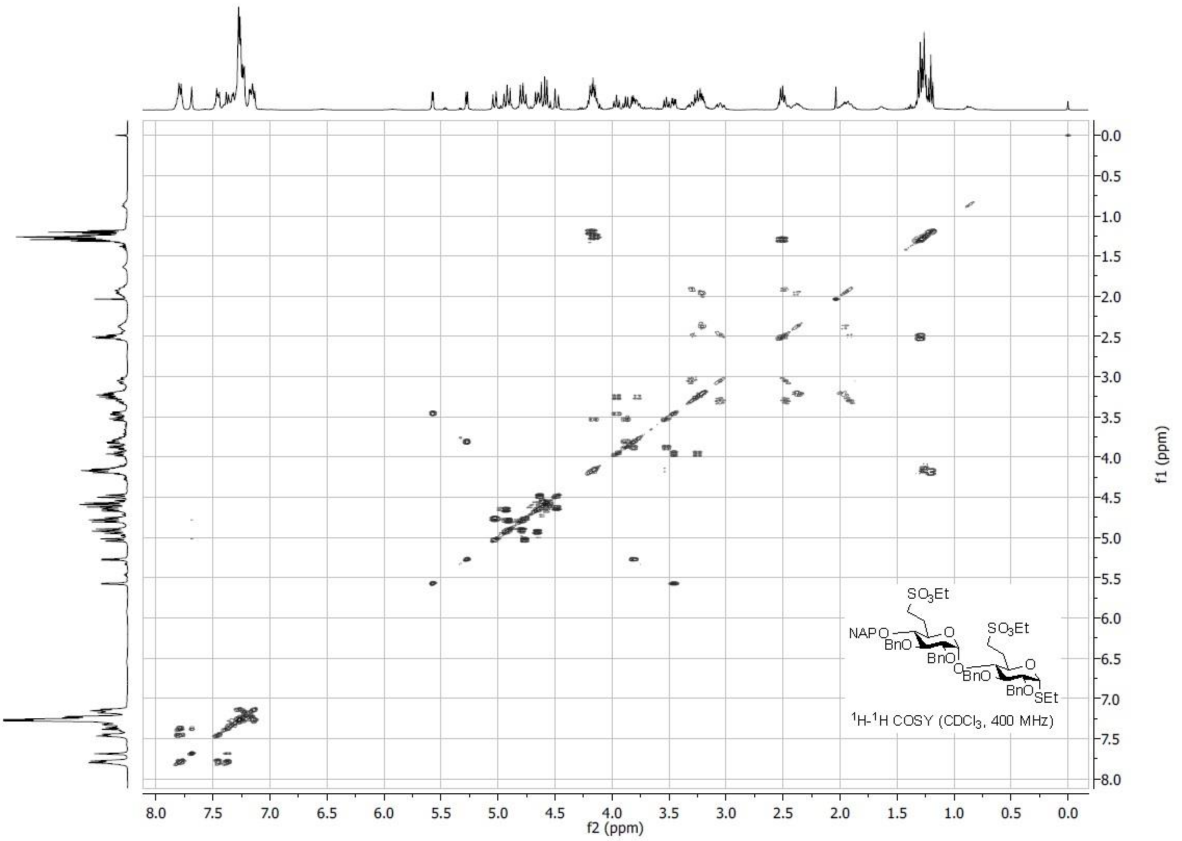

SO_3Et
NAPO
BnO
BnO
BnO
BnO
SEt
1H-1H COSY (CDCl3, 400 MHz)
f2 (ppm)
f1 (ppm)

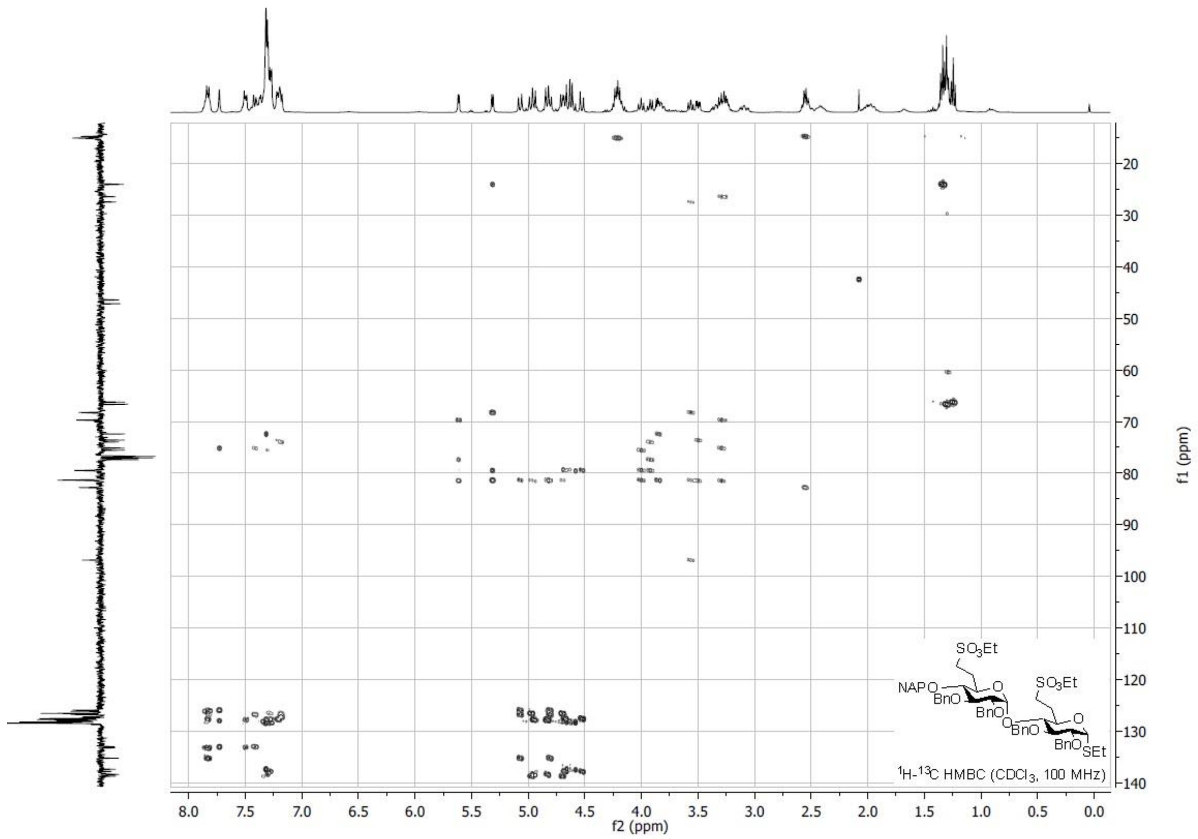
SO3Et
NAPO
BnO
BnO
O
SO3Et
BnO
BnO
SEt
1H-13C HMBC (CDCl3, 100 MHz)
f1 (ppm)
f2 (ppm)

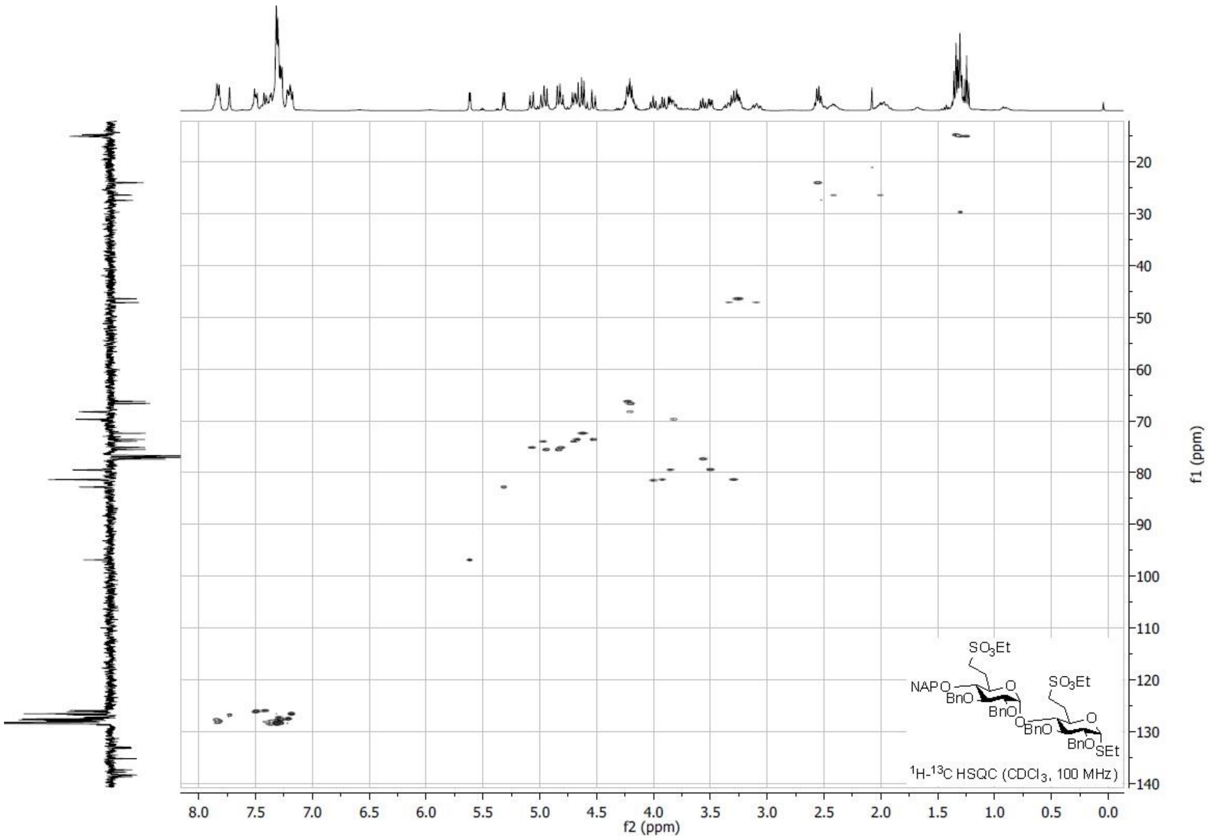
SO3Et
NAPO
BnO
BnO
O
SO3Et
BnO
BnO
SEt
1H-13C HSQC (CDCl3, 100 MHz)
f1 (ppm)
f2 (ppm)

^{1}H and ^{13}C NMR spectra of compound 11

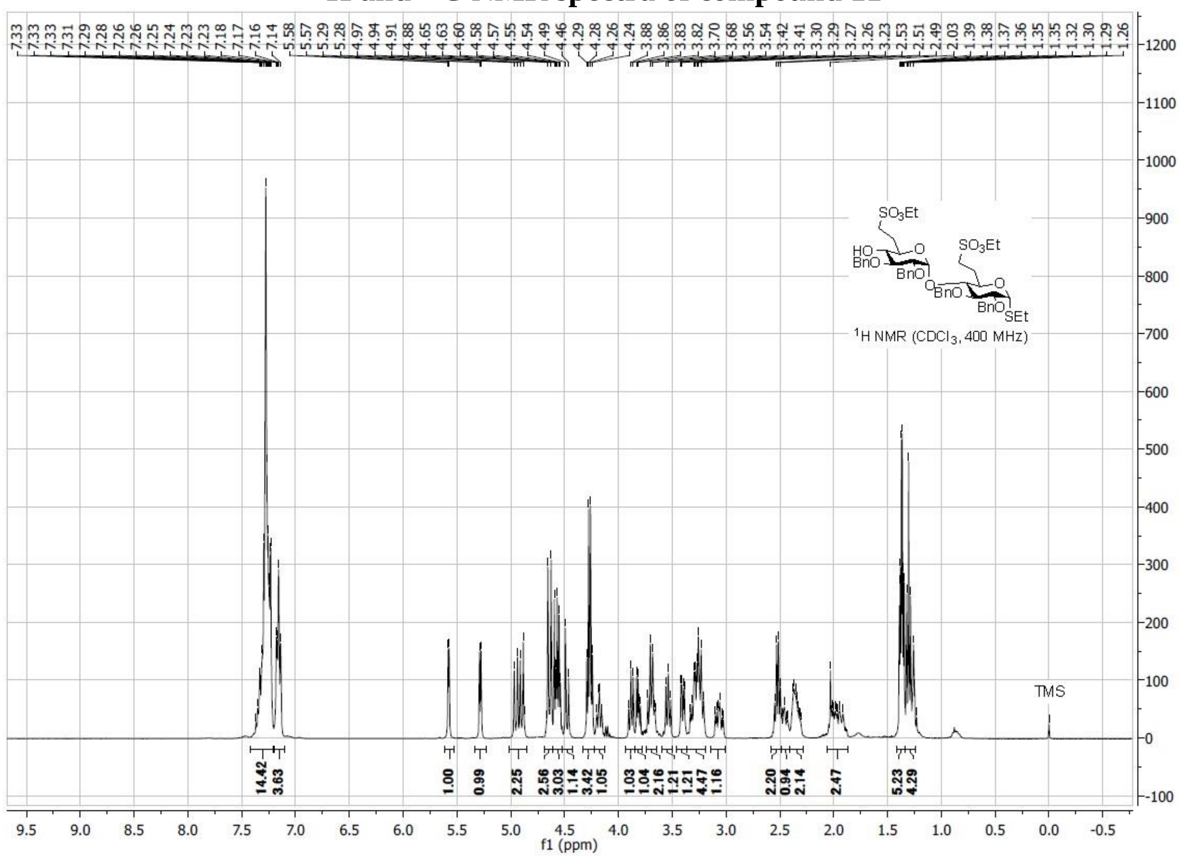

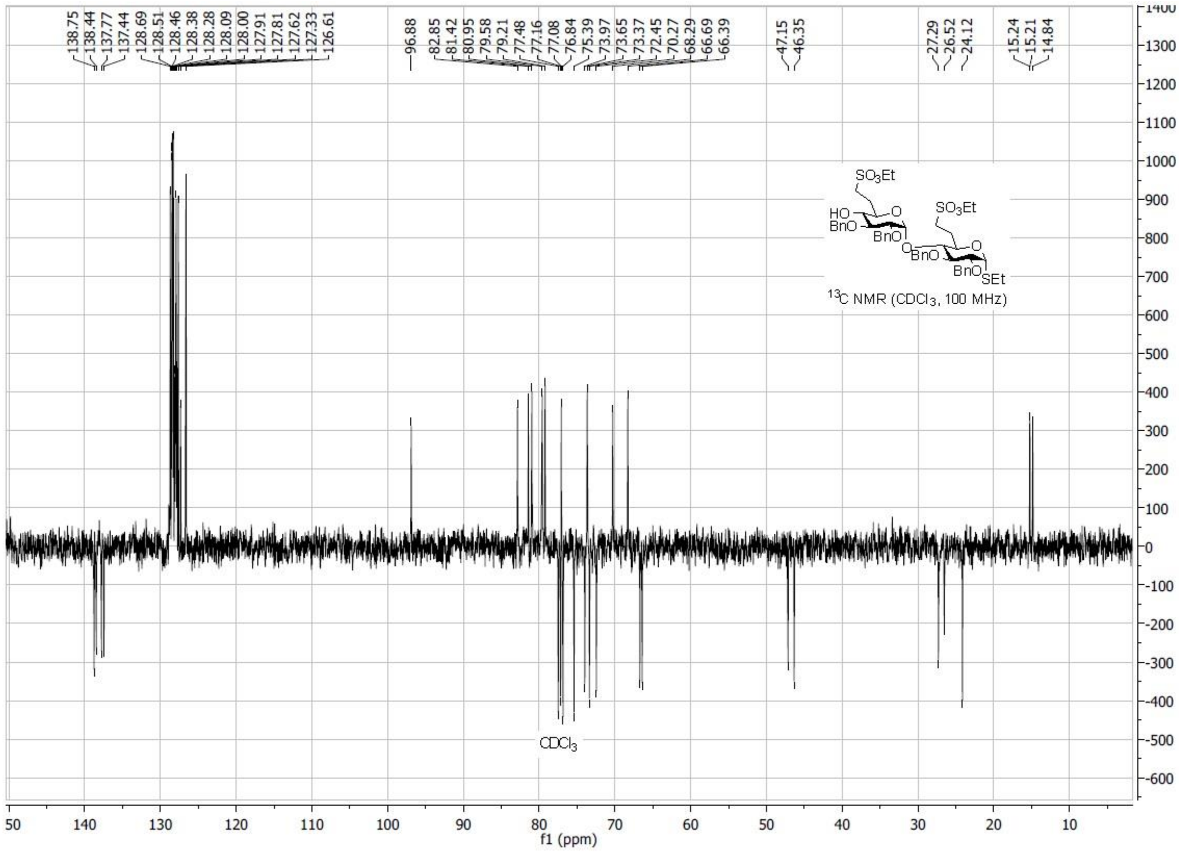

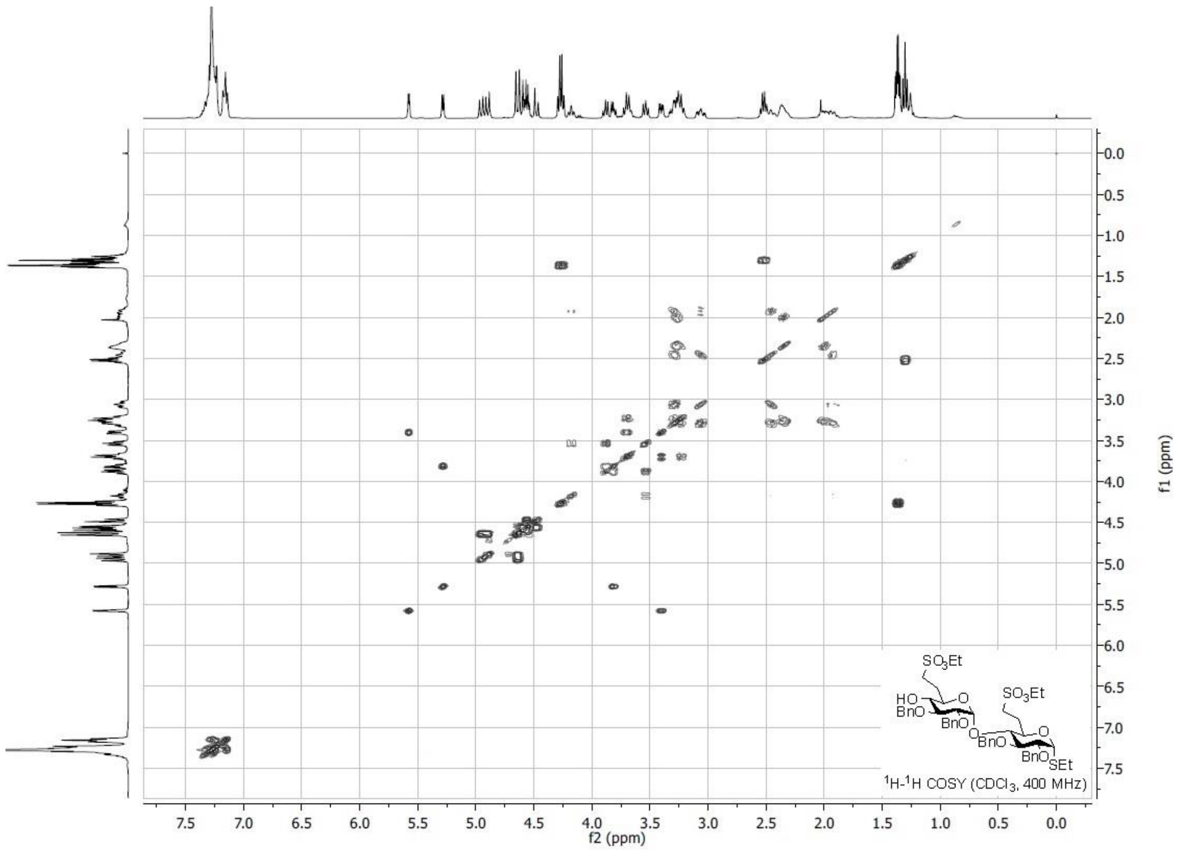
SO3Et
HO
BnO
BnO
O
SO3Et
BnO
BnO
SEt
1H-1H COSY (CDCl3, 400 MHz)
f2 (ppm)
f1 (ppm)

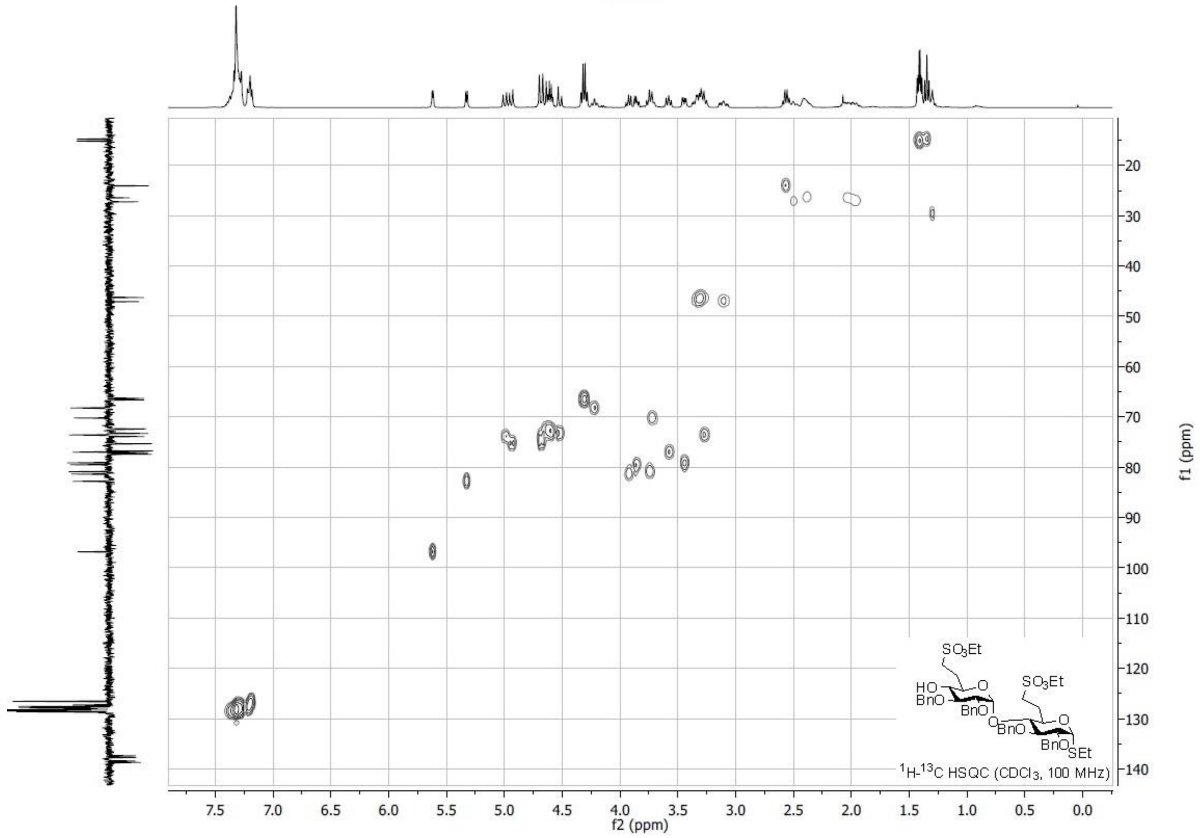
SO3Et
HO
BnO
BnO
O
SO3Et
BnO
BnO
SEt
1H-13C HSQC (CDCl3, 100 MHz)
f2 (ppm)
f1 (ppm)

^{1}H and ^{13}C NMR spectra of compound 12

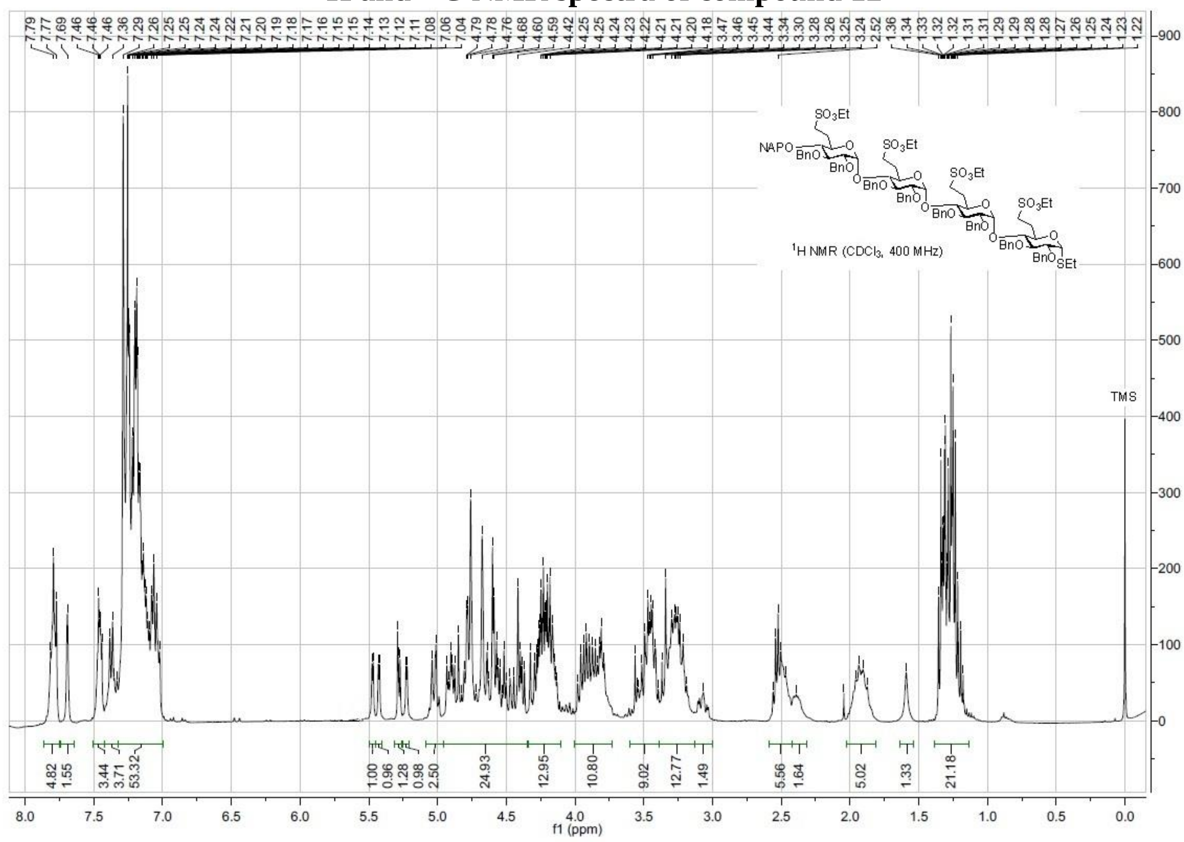

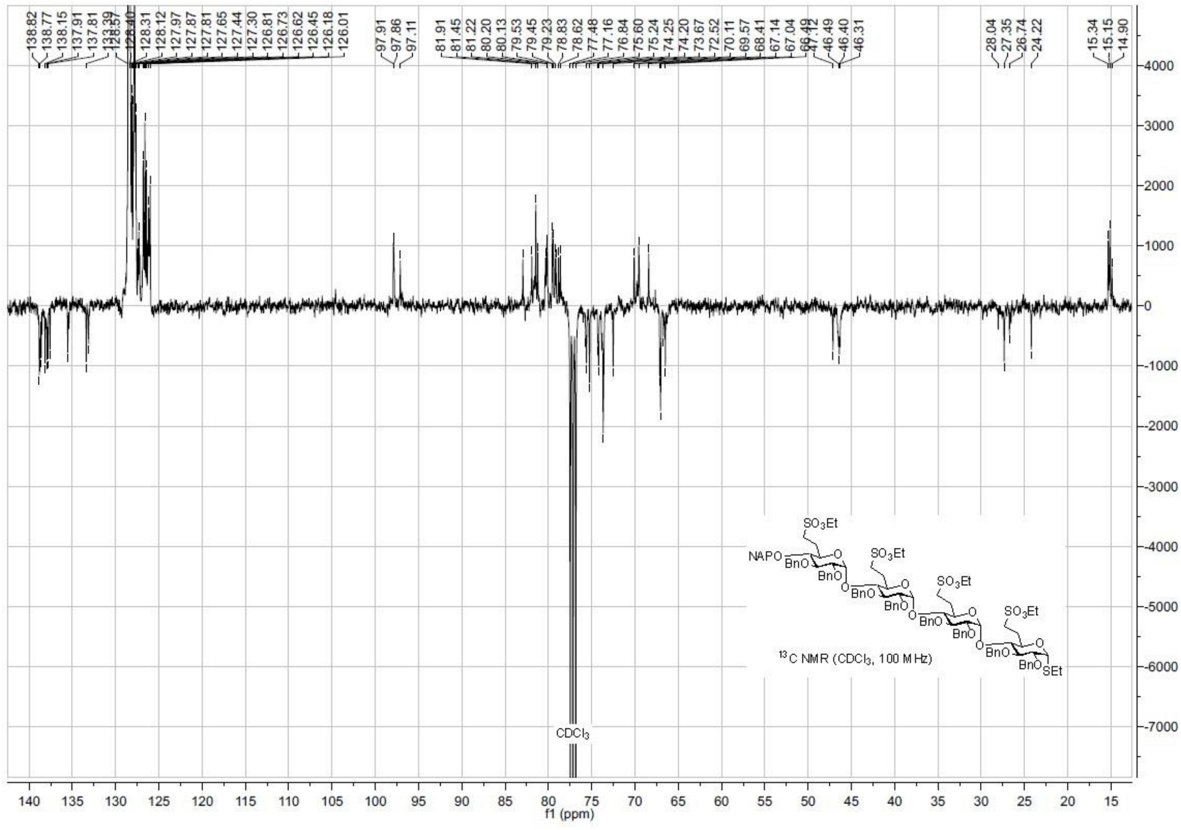

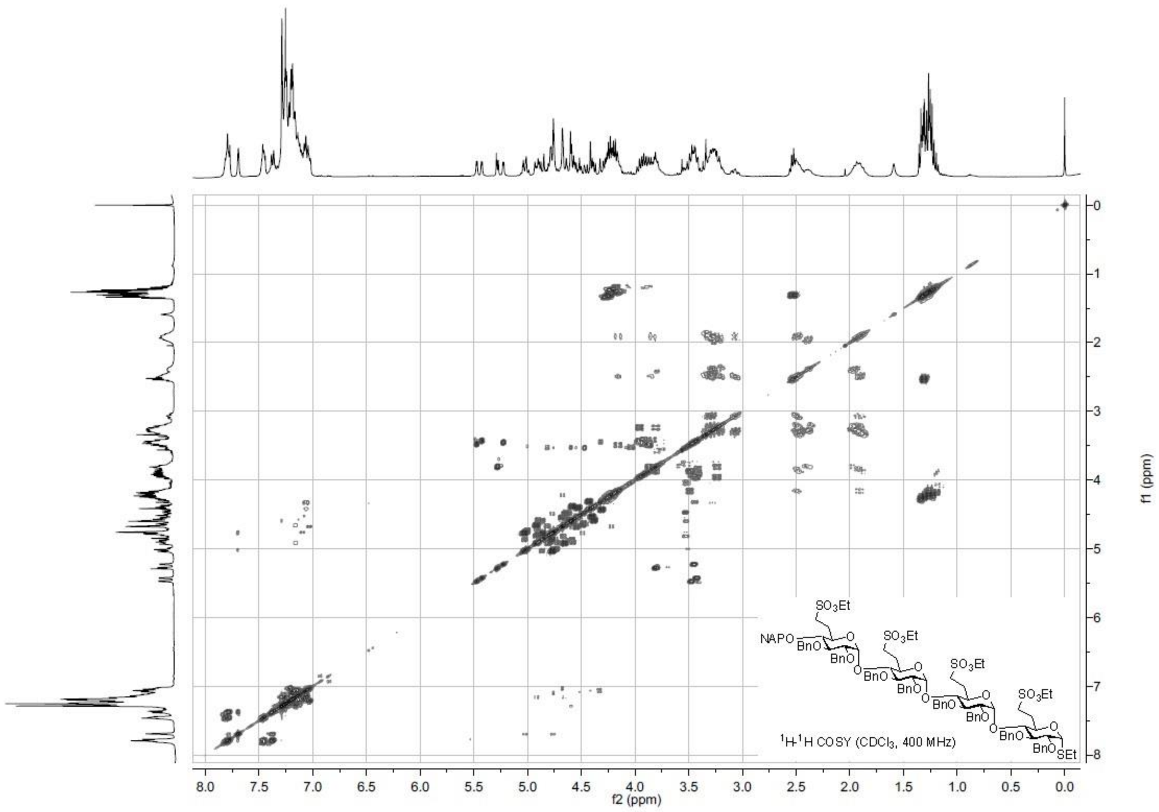

SO3Et
NAPO
BnO
BnO
SEt
1H-1H COSY (CDCl3, 400 MHz)
f2 (ppm)
f1 (ppm)

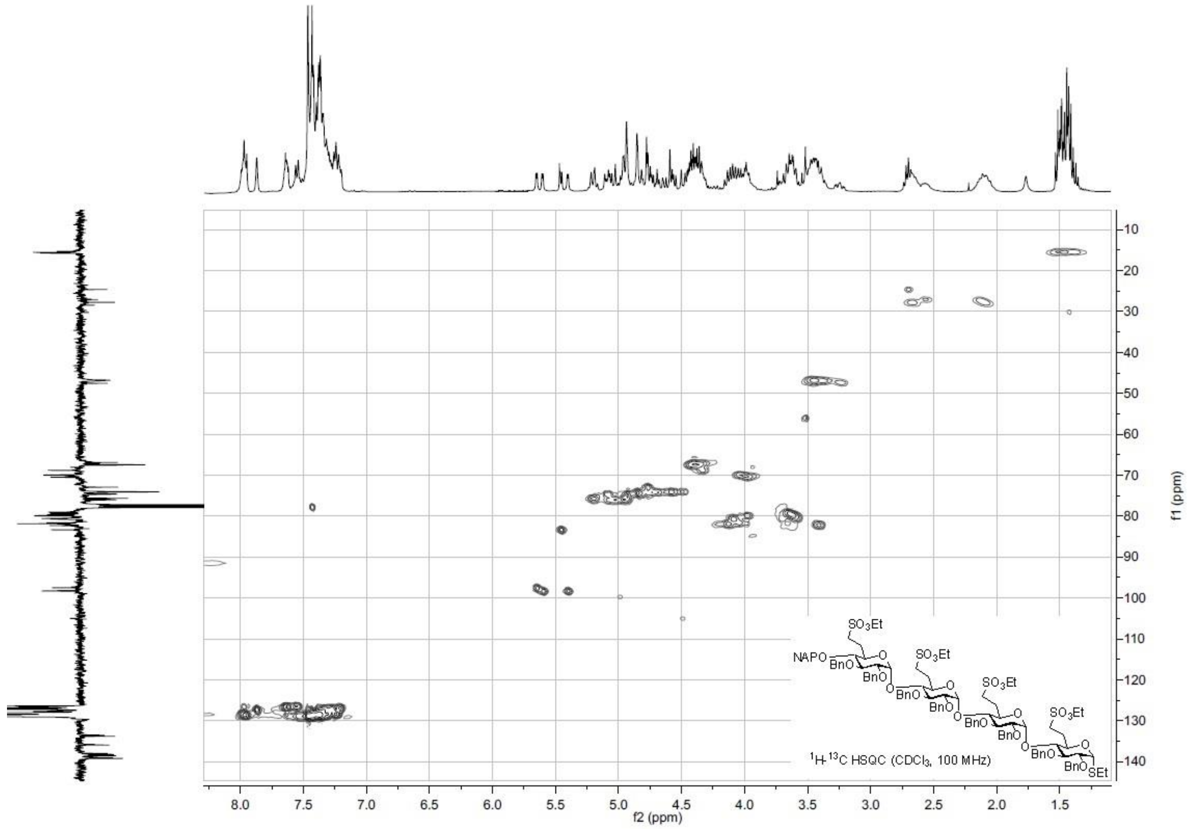

SO3Et
NAPO
BnO
BnO
SEt
1H-13C HSQC (CDCl3, 100 MHz)
f2 (ppm)
f1 (ppm)

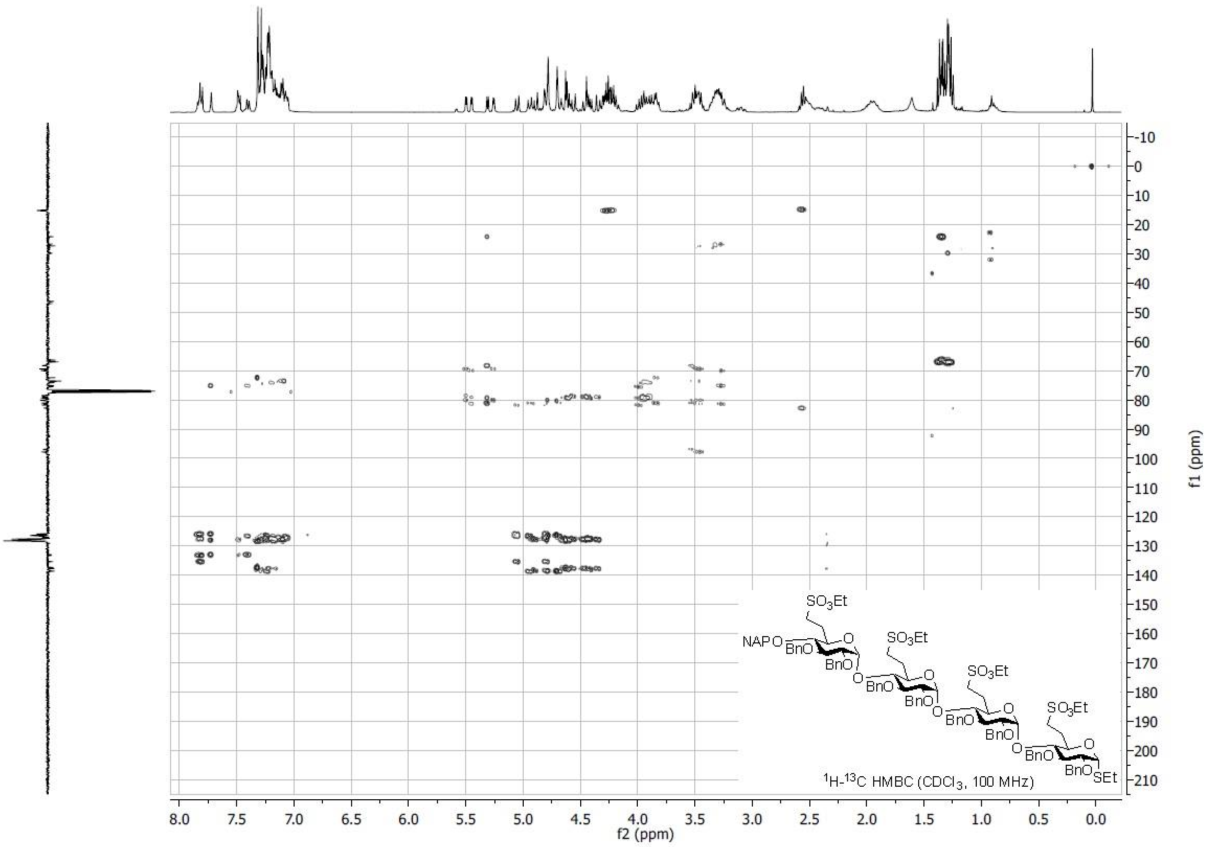

SO3Et
NAPO
BnO
BnO
O
SO3Et
SO3Et
SO3Et
SEt
1H-13C HMBC (CDCl3, 100 MHz)
f2 (ppm)
f1 (ppm)

^{1}H and ^{13}C NMR spectra of compound 31

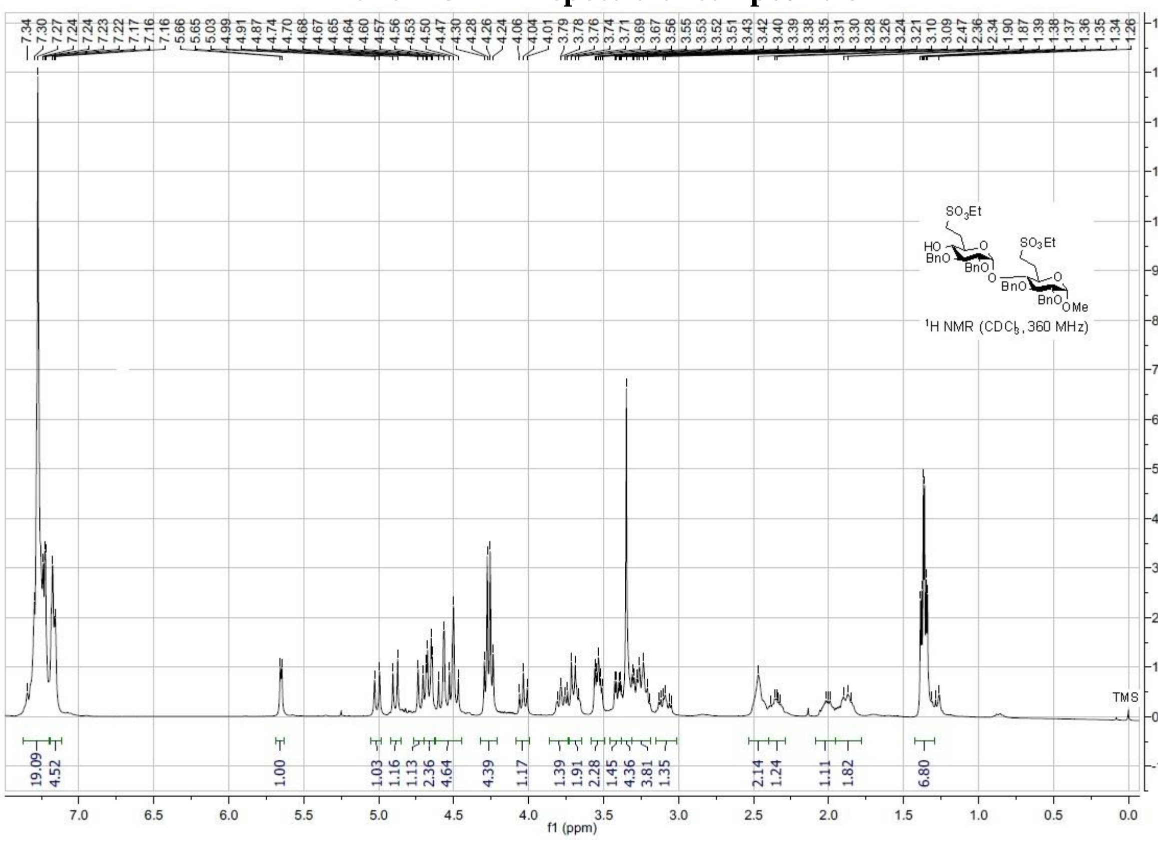

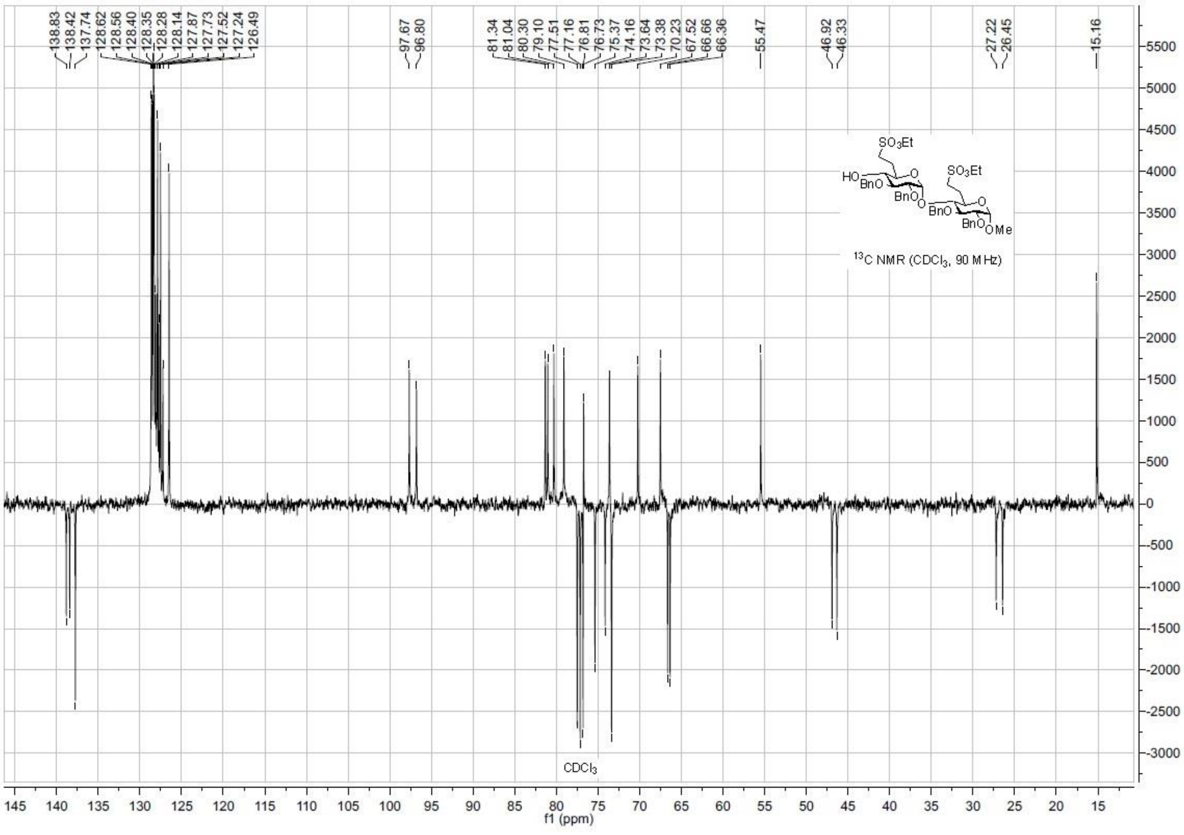

^{1}H and ^{13}C NMR spectra of compound 32

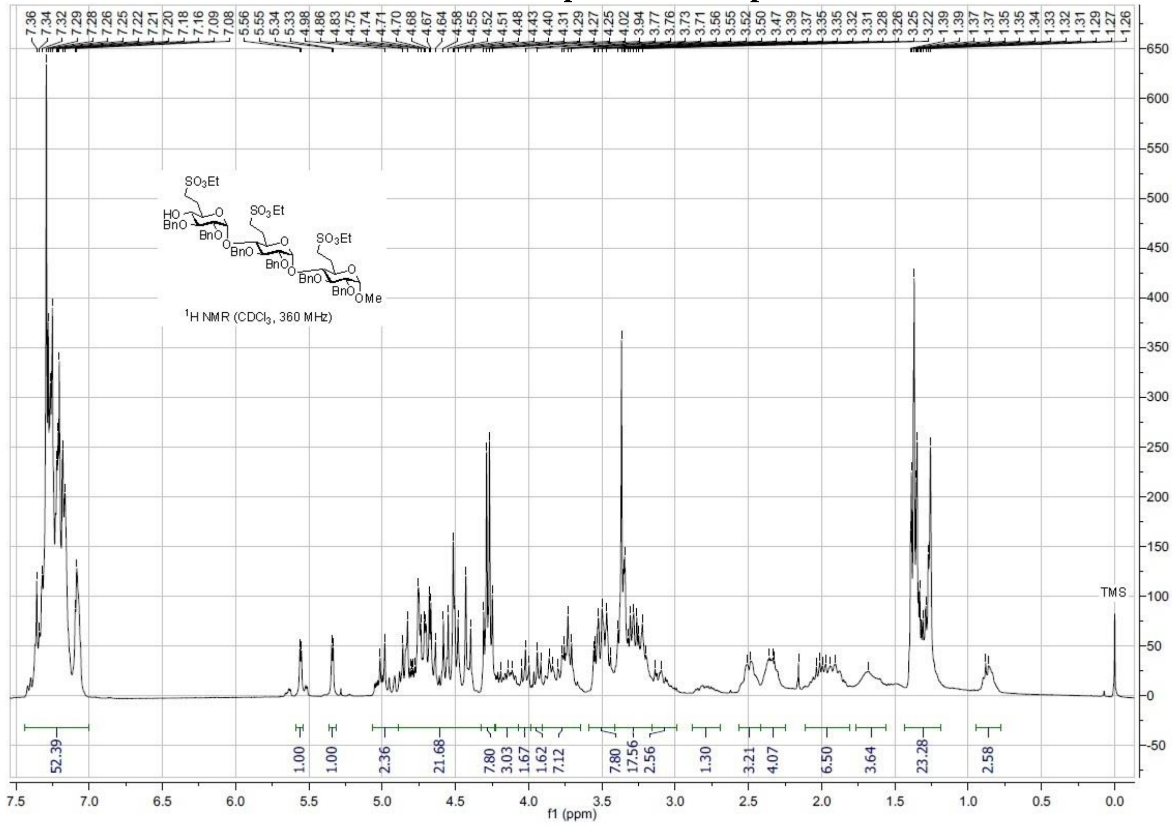

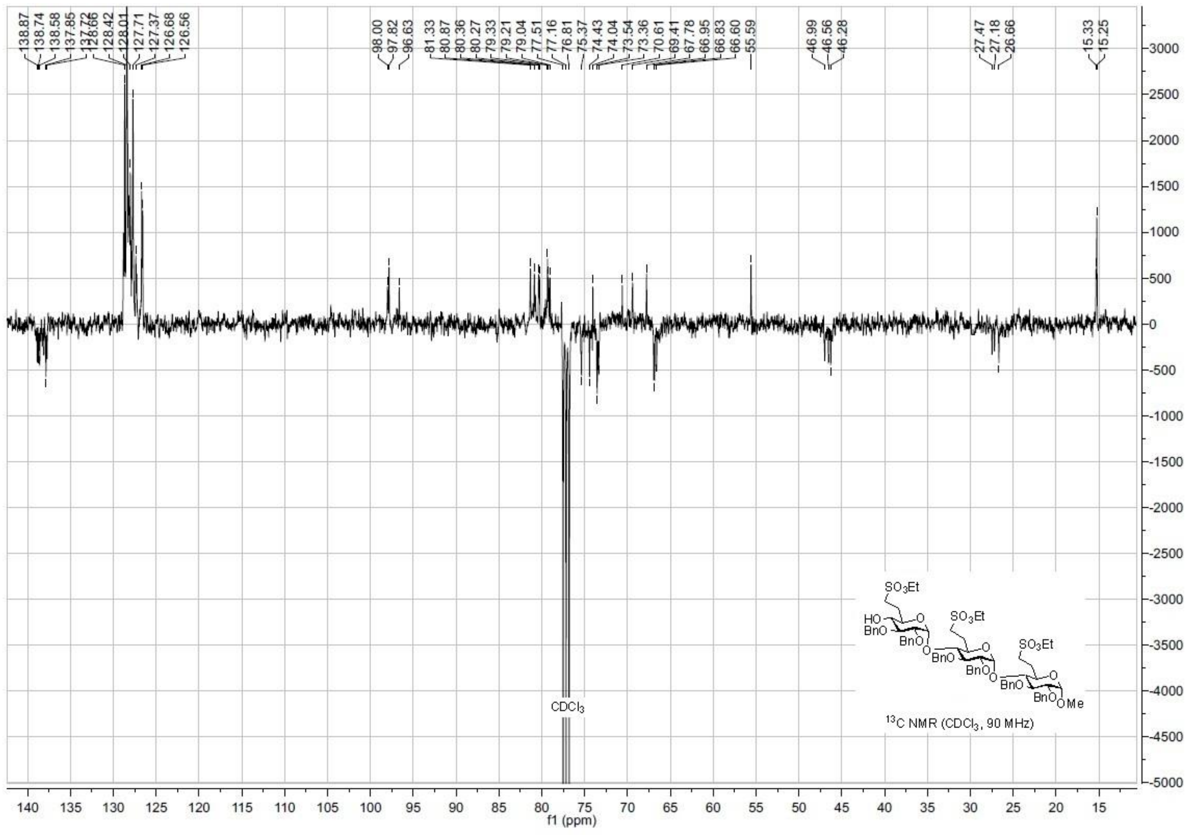

^{1}H and ^{13}C NMR spectra of compound 51

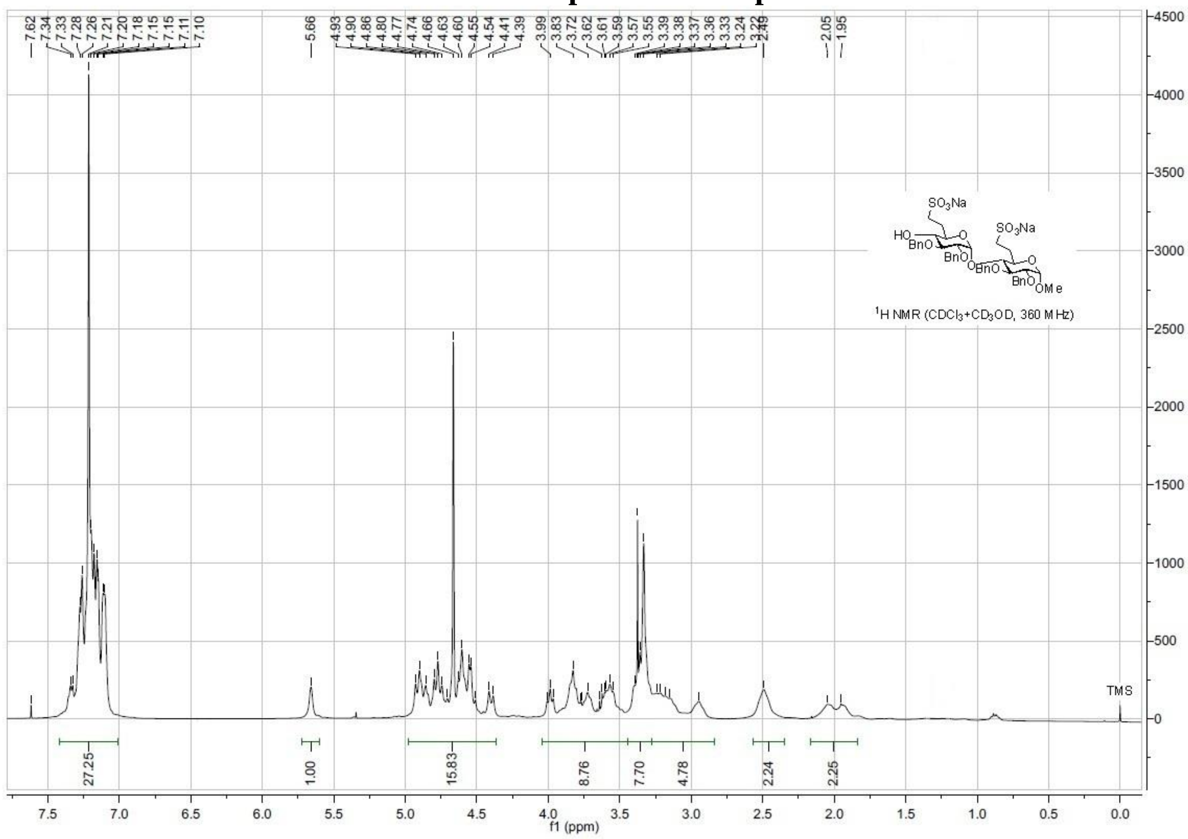

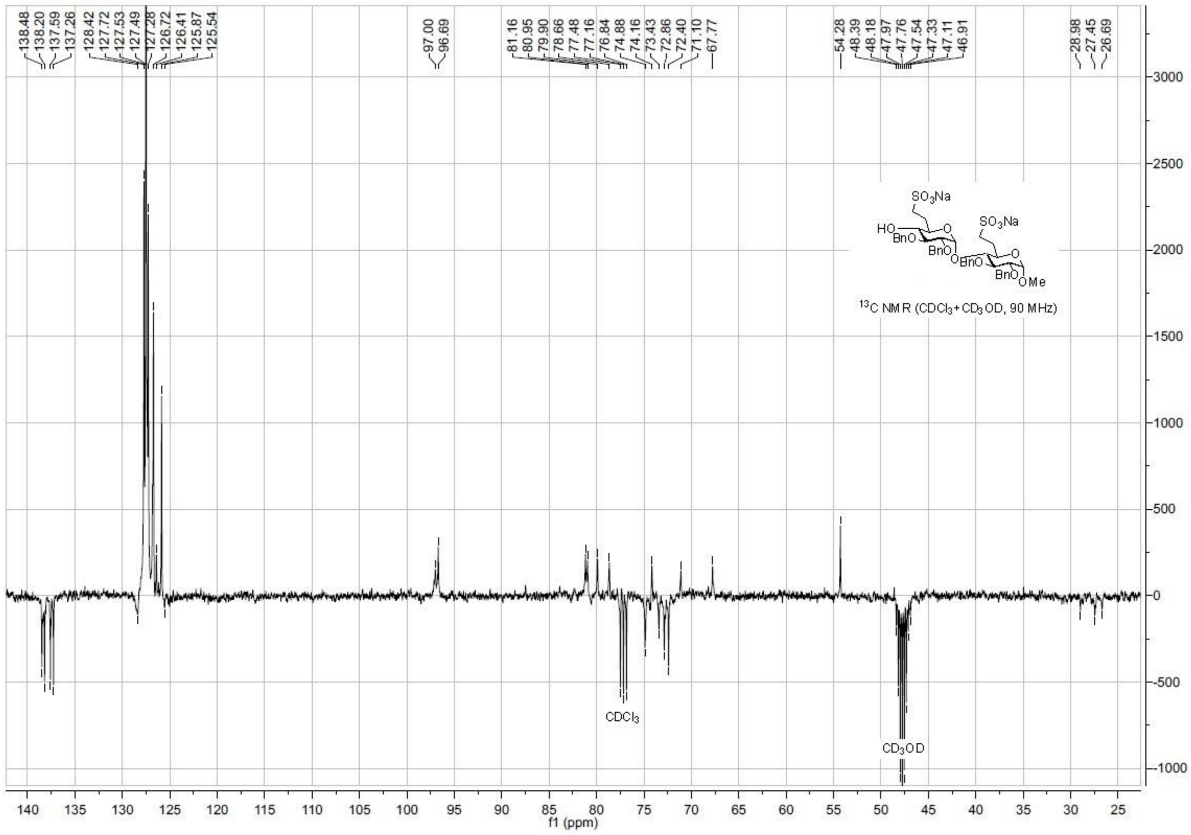

^{1}H and ^{13}C NMR spectra of compound 53

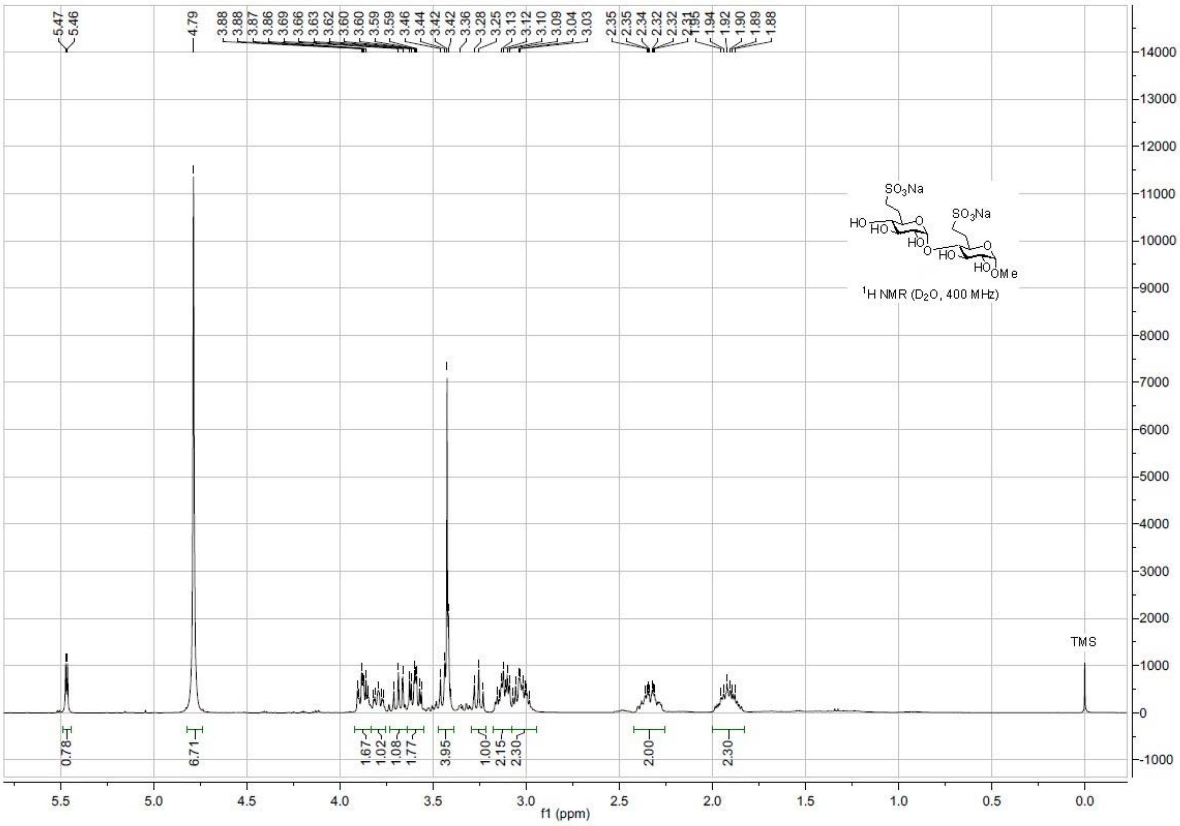

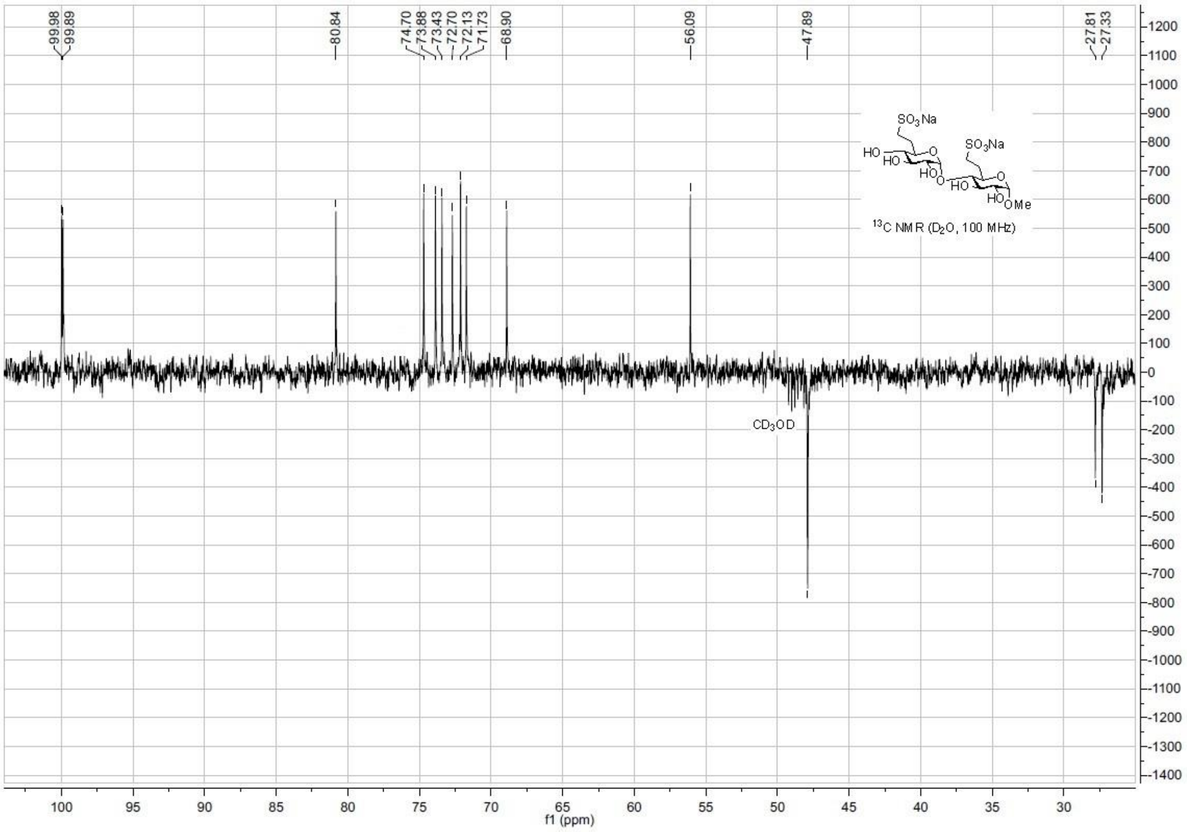

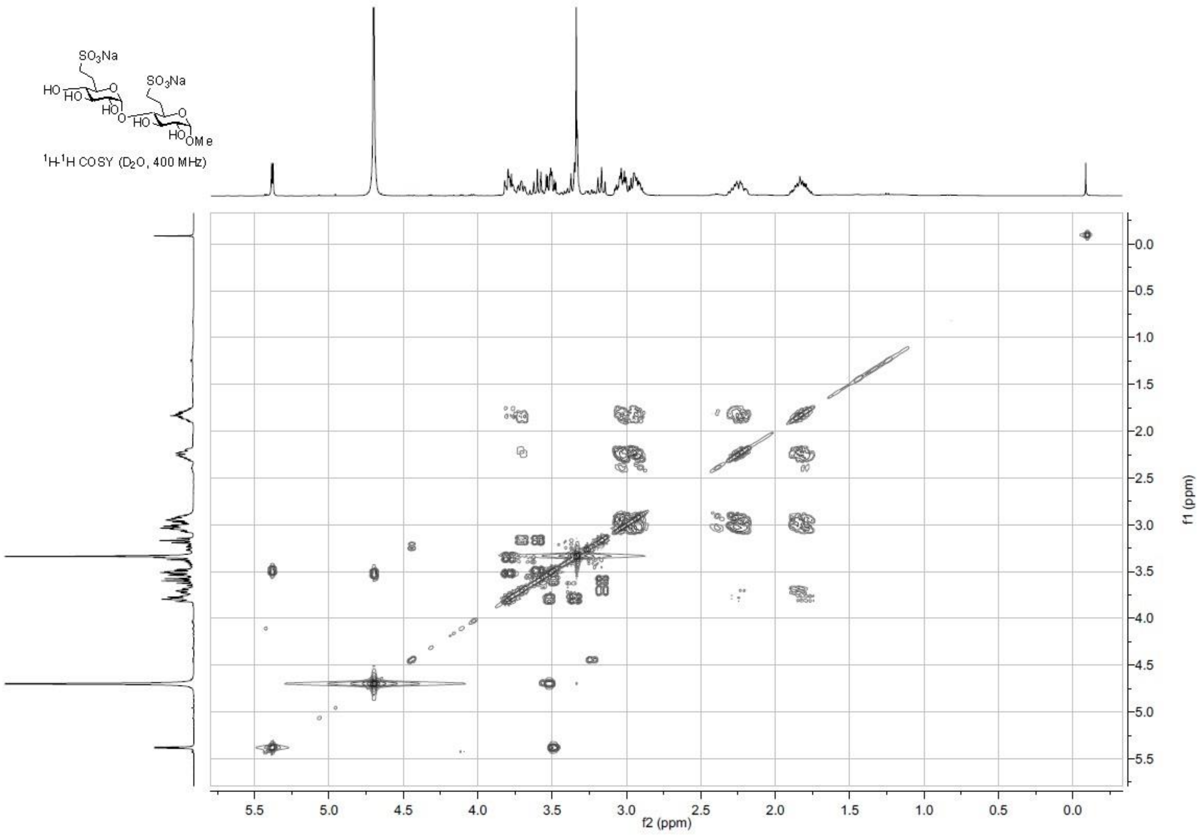
SO3Na
HO
HO
HO
O
SO3Na
HO
HO
OMe
1H-1H COSY (D2O, 400 MHz)
f2 (ppm)
f1 (ppm)

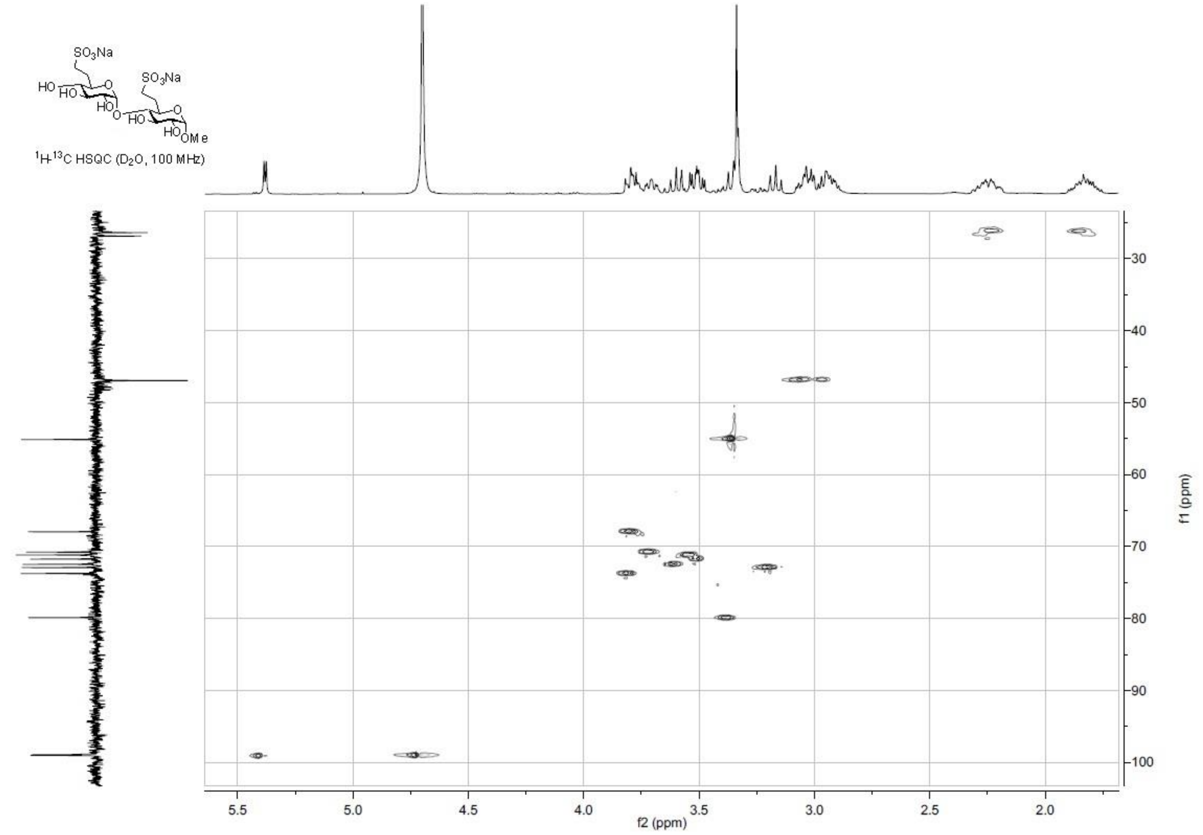
SO3Na
HO
HO
HO
O
SO3Na
HO
HO
OMe
1H-13C HSQC (D2O, 100 MHz)
f2 (ppm)
f1 (ppm)

^{1}H and ^{13}C NMR spectra of heptaol

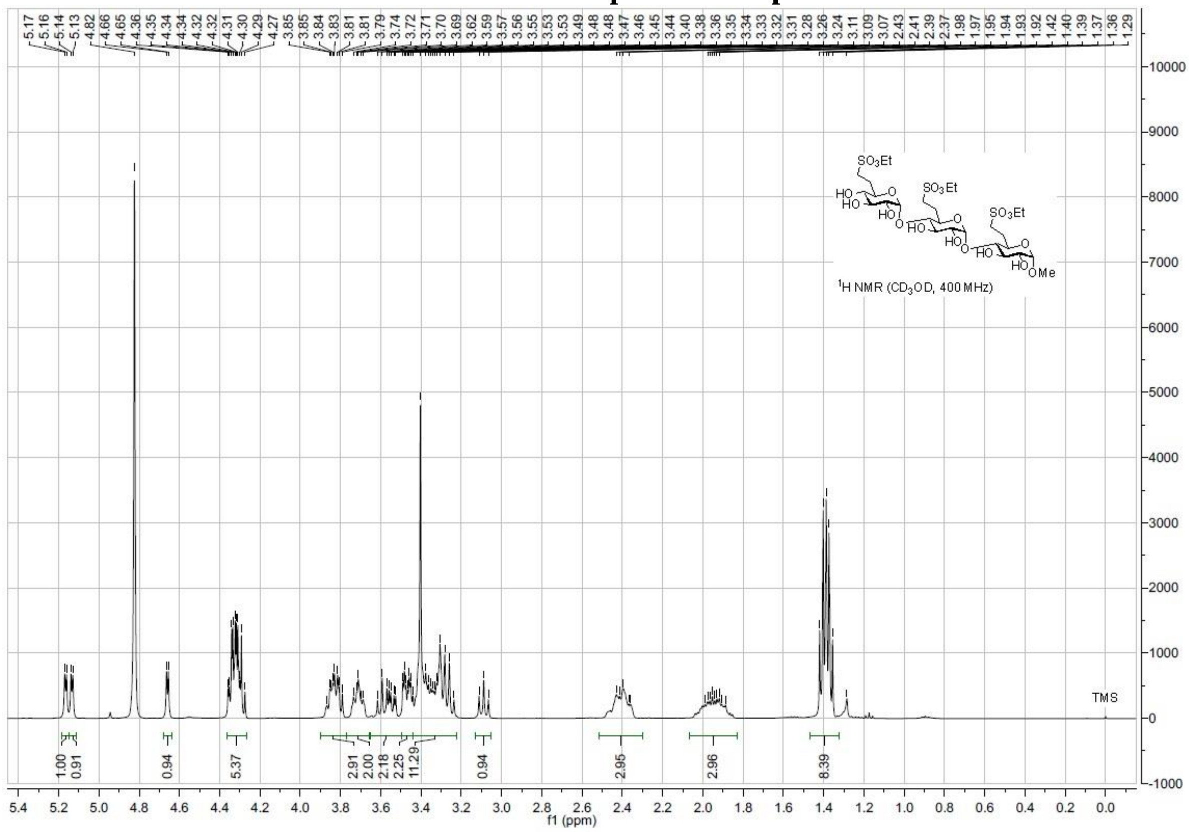

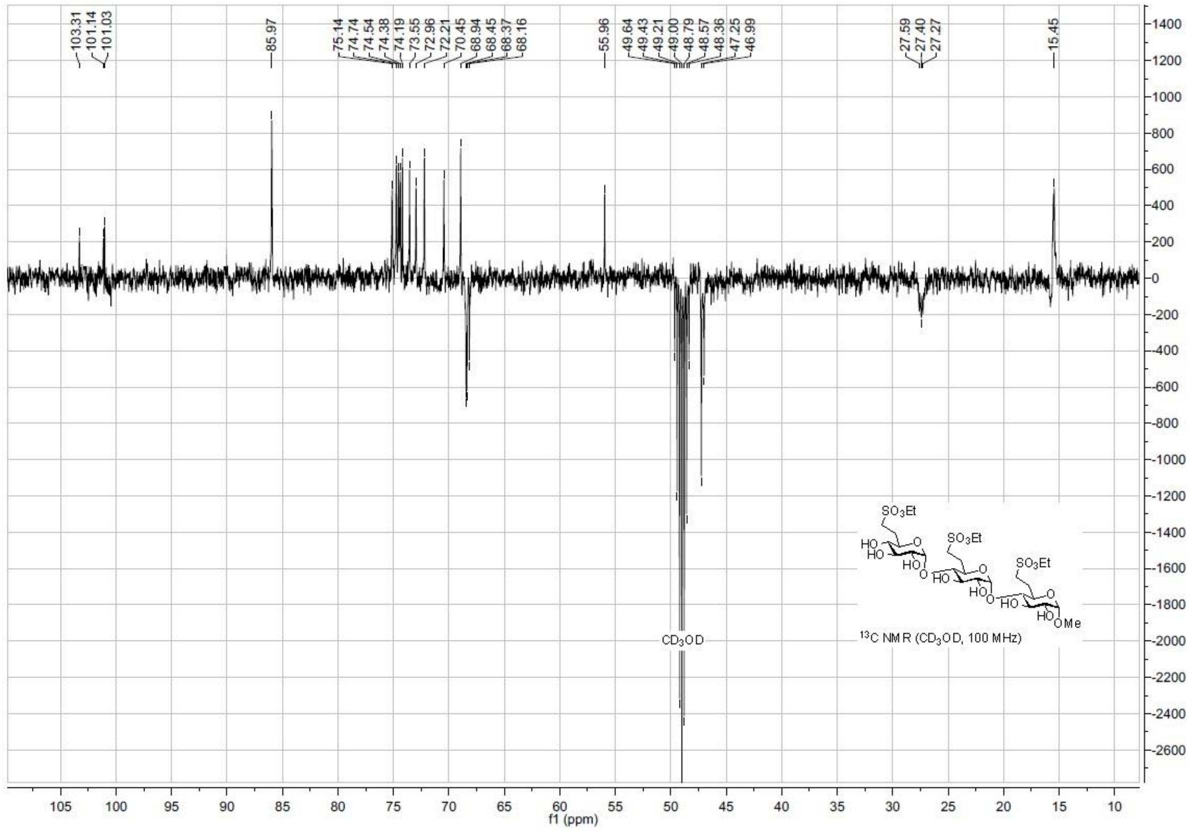

^{1}H and ^{13}C NMR spectra of compound 54

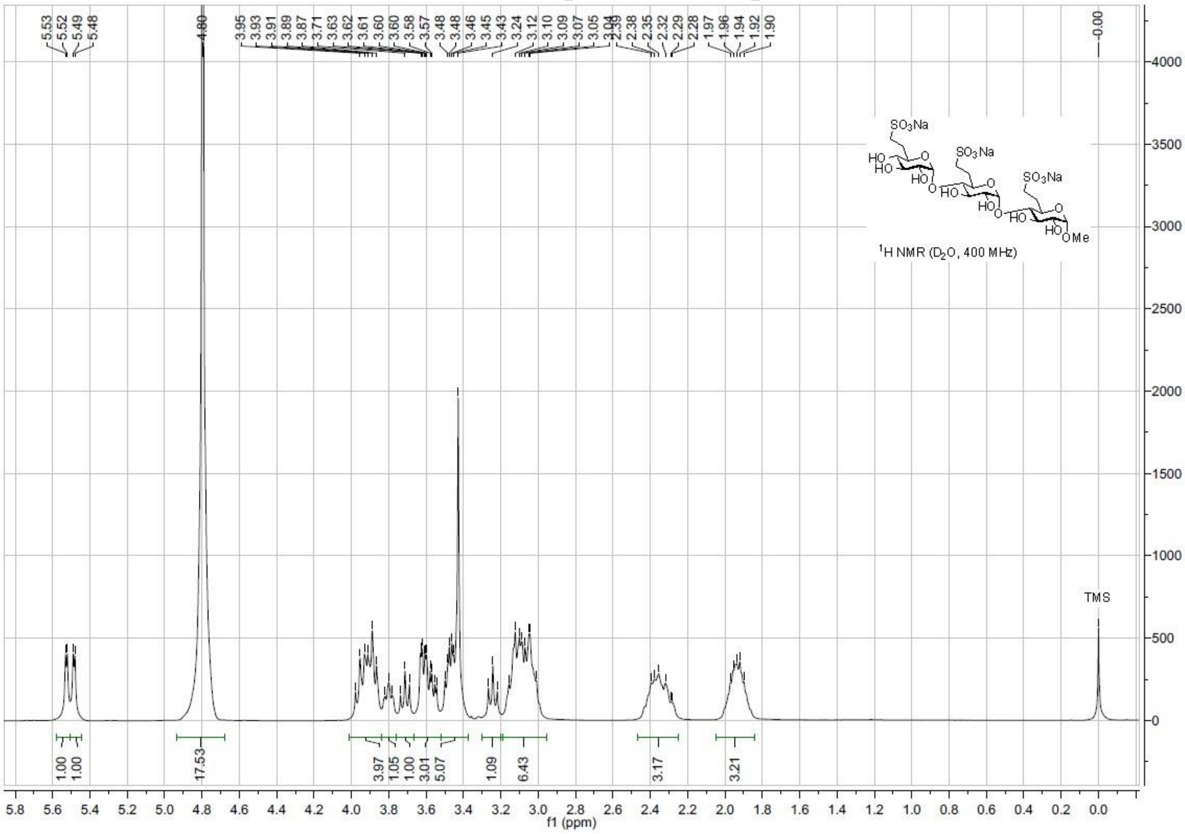

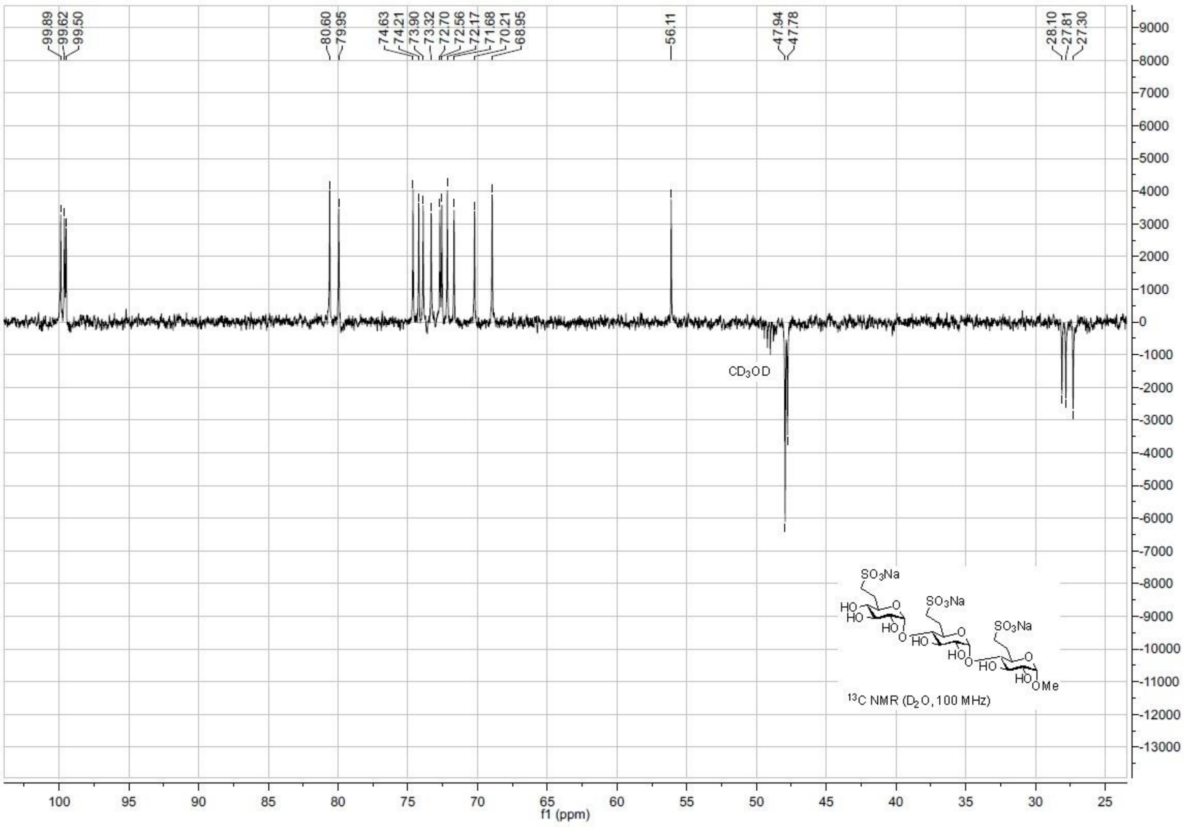

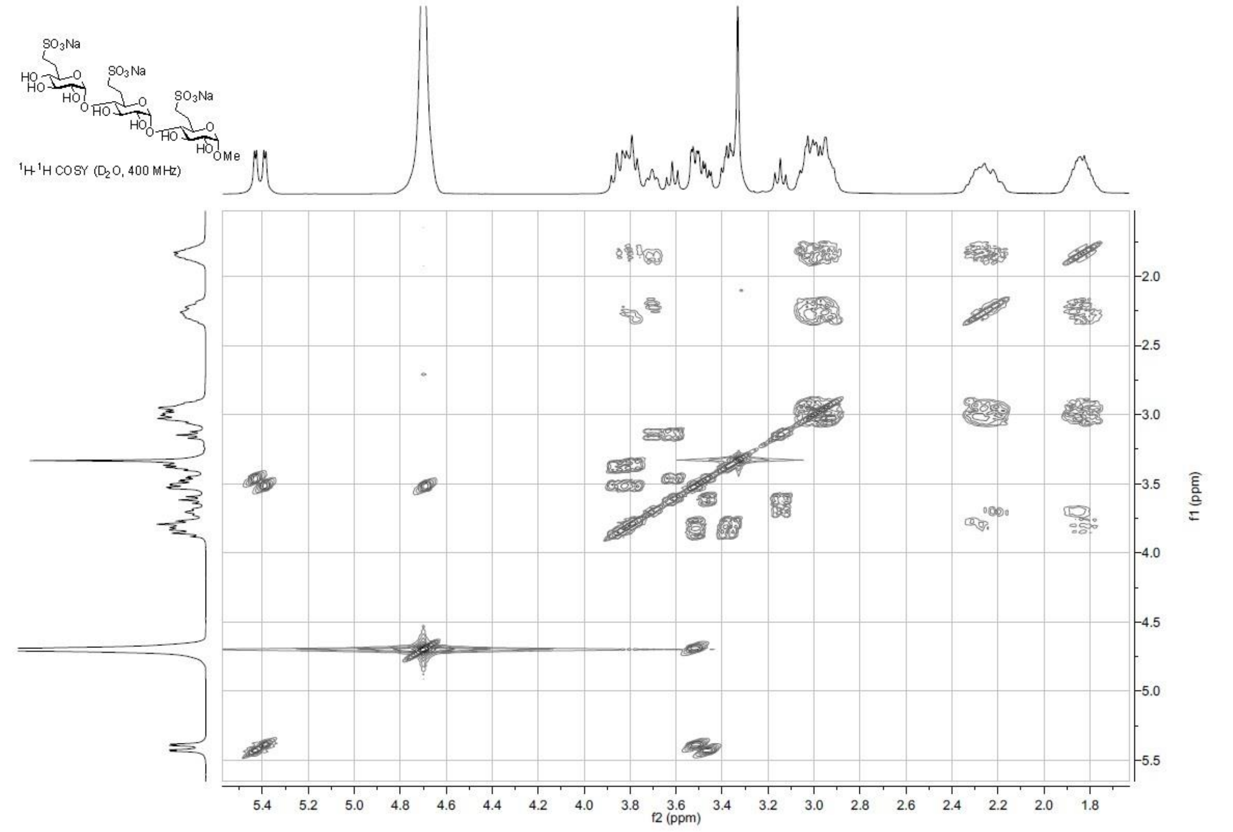

SO3Na
HO
HO
HO
O
SO3Na
HO
O
SO3Na
HO
OMe
1H-1H COSY (D2O, 400 MHz)
f1 (ppm)
f2 (ppm)

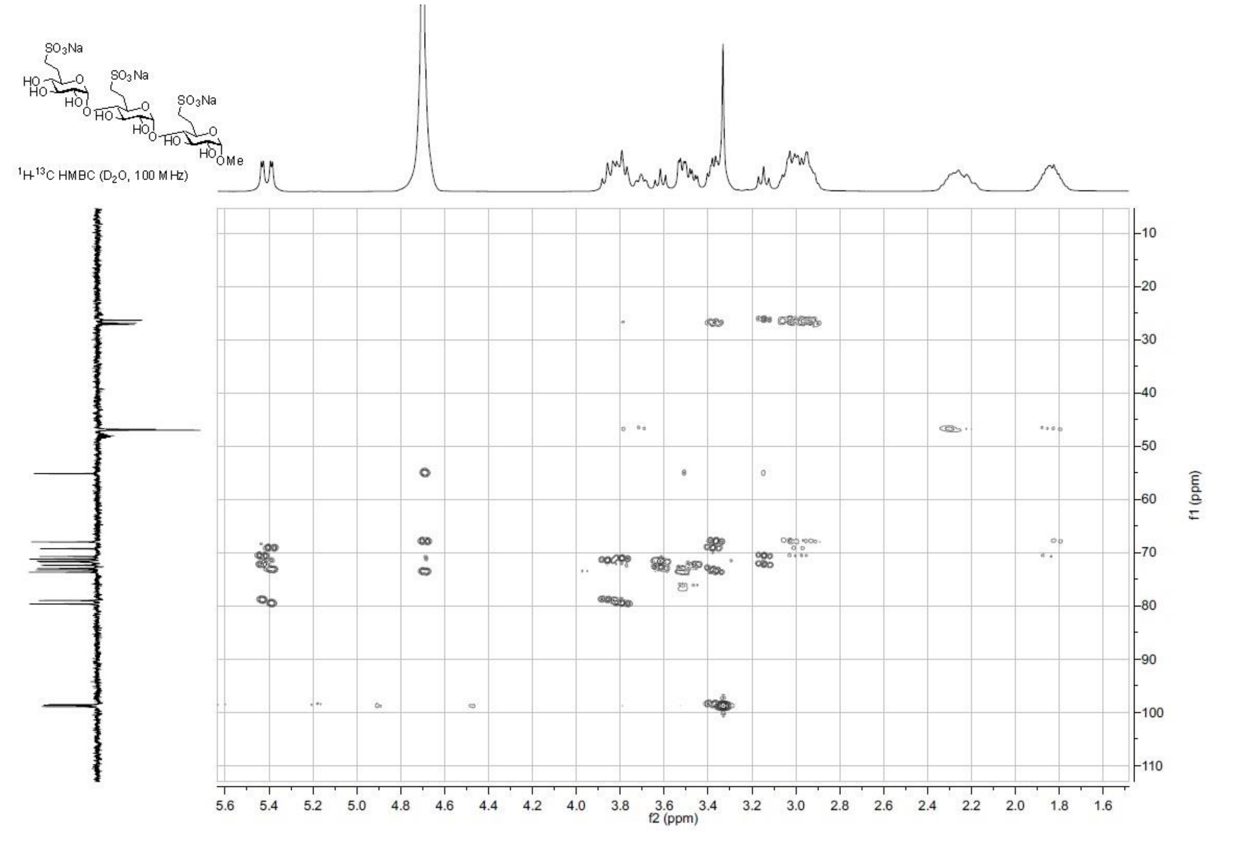

SO3Na
HO
HO
HO
O
SO3Na
HO
O
SO3Na
HO
OMe
1H-13C HMBC (D2O, 100 MHz)
f1 (ppm)
f2 (ppm)

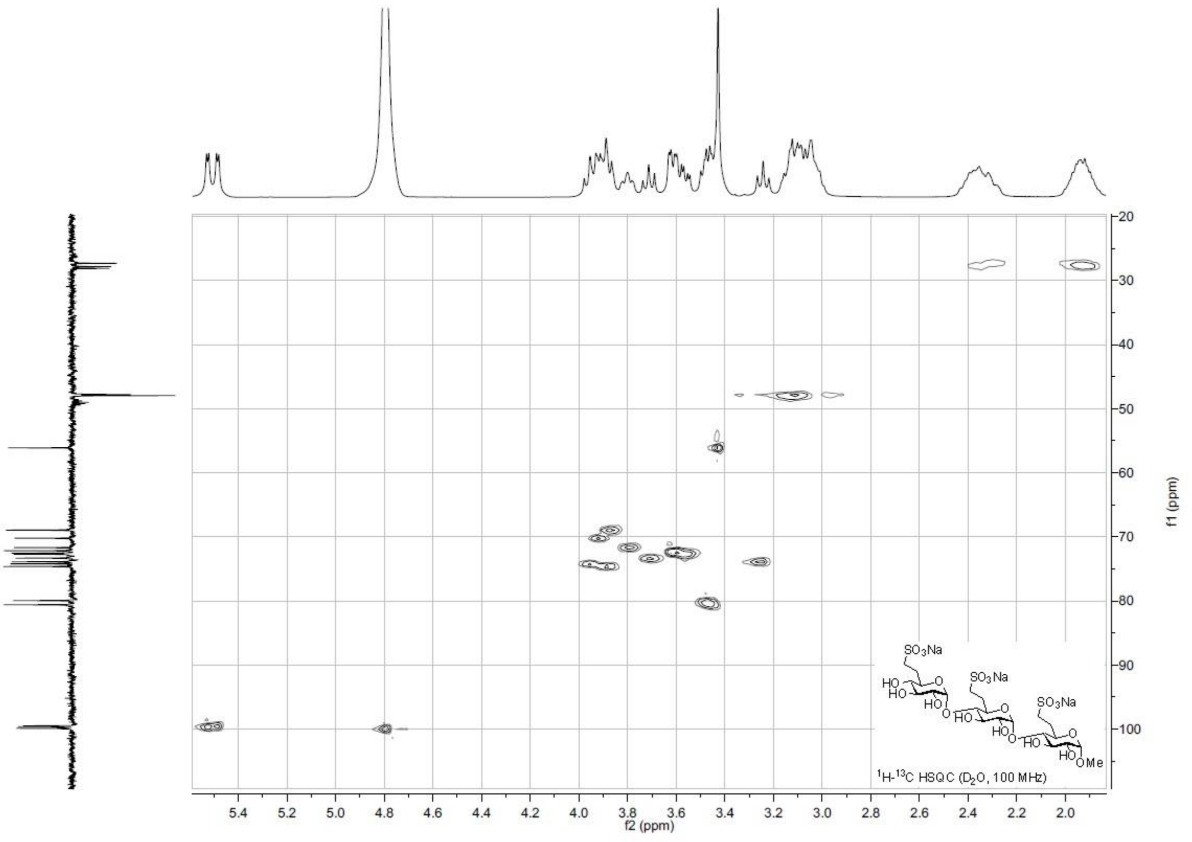
f1 (ppm)
f2 (ppm)
SO3Na
HO
HO
HO
O
OMe
1H-13C HSQC (D2O, 100 MHz)

^{1}H and ^{13}C NMR spectra of compound 56

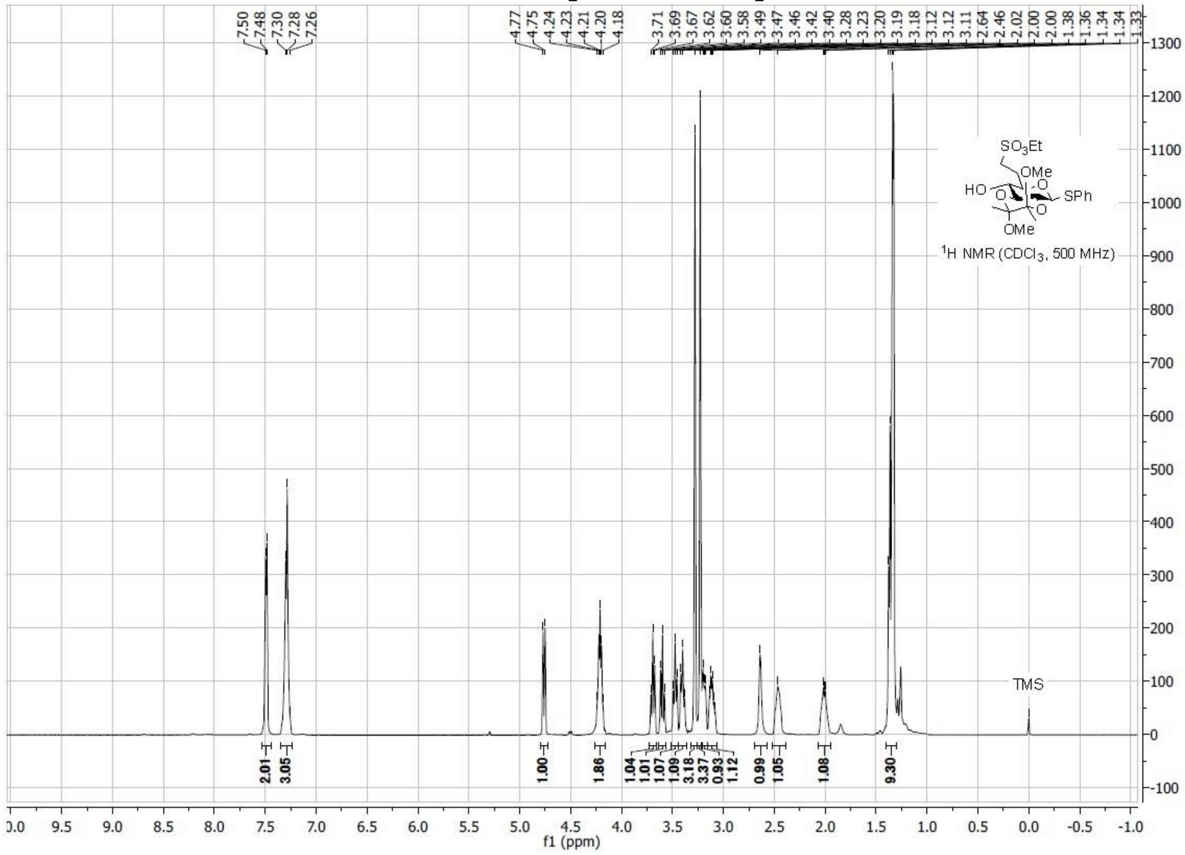

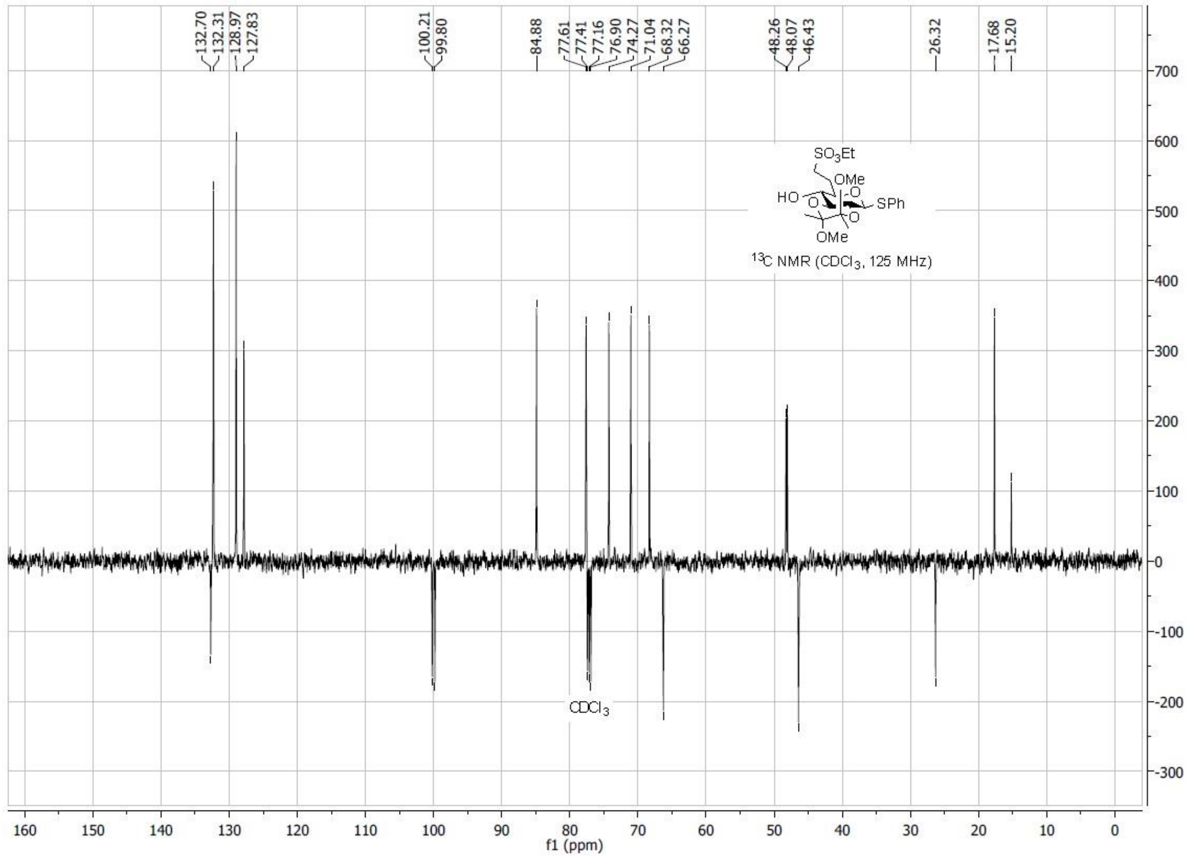

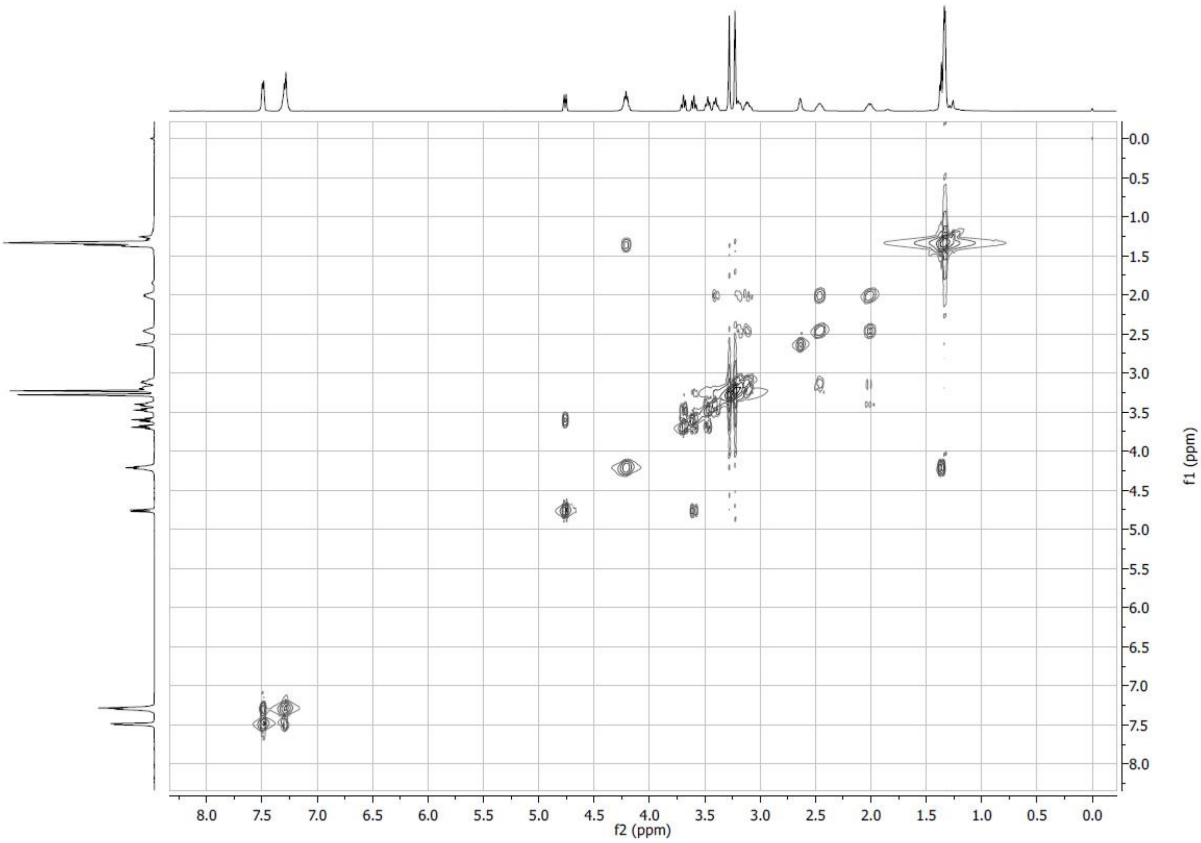
f2 (ppm)
f1 (ppm)

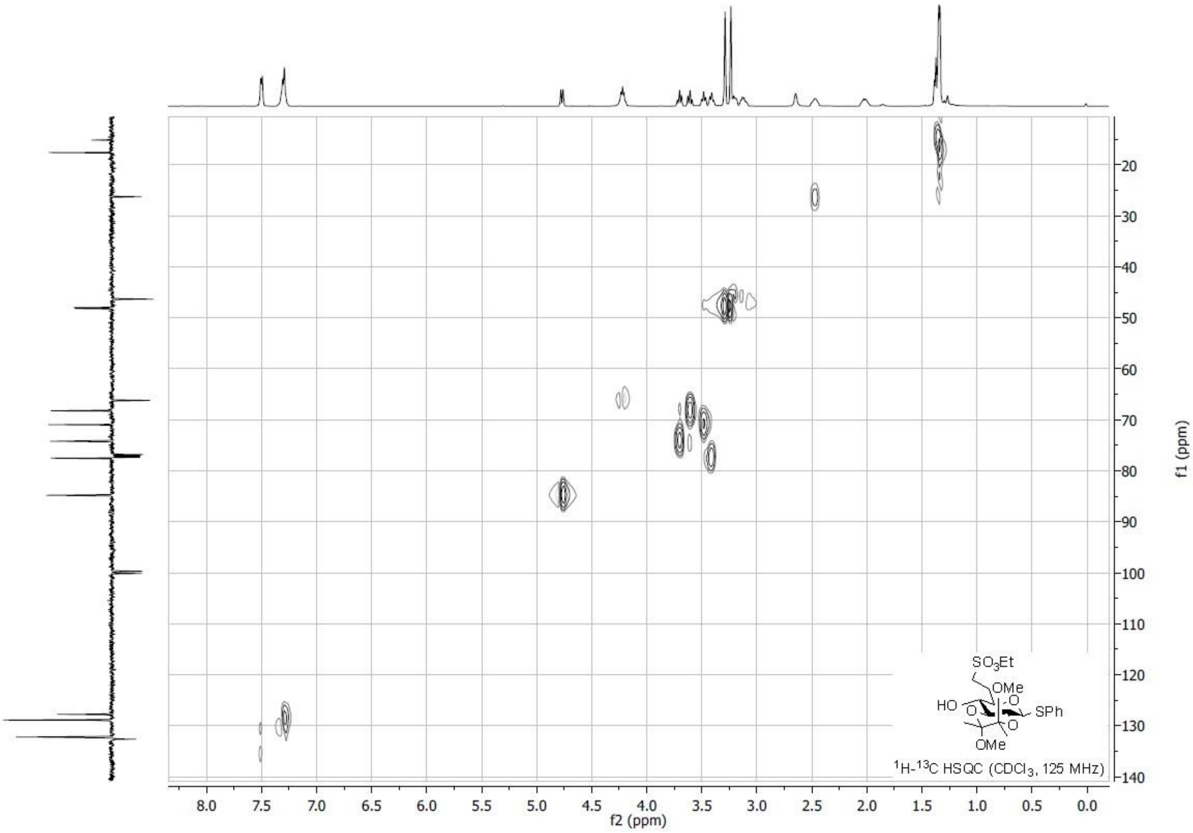
SO3Et
OMe
HO
O
O
SPh
O
OMe
1H-13C HSQC (CDCl3, 125 MHz)
f2 (ppm)
f1 (ppm)

^{1}H and ^{13}C NMR spectra of compound 58

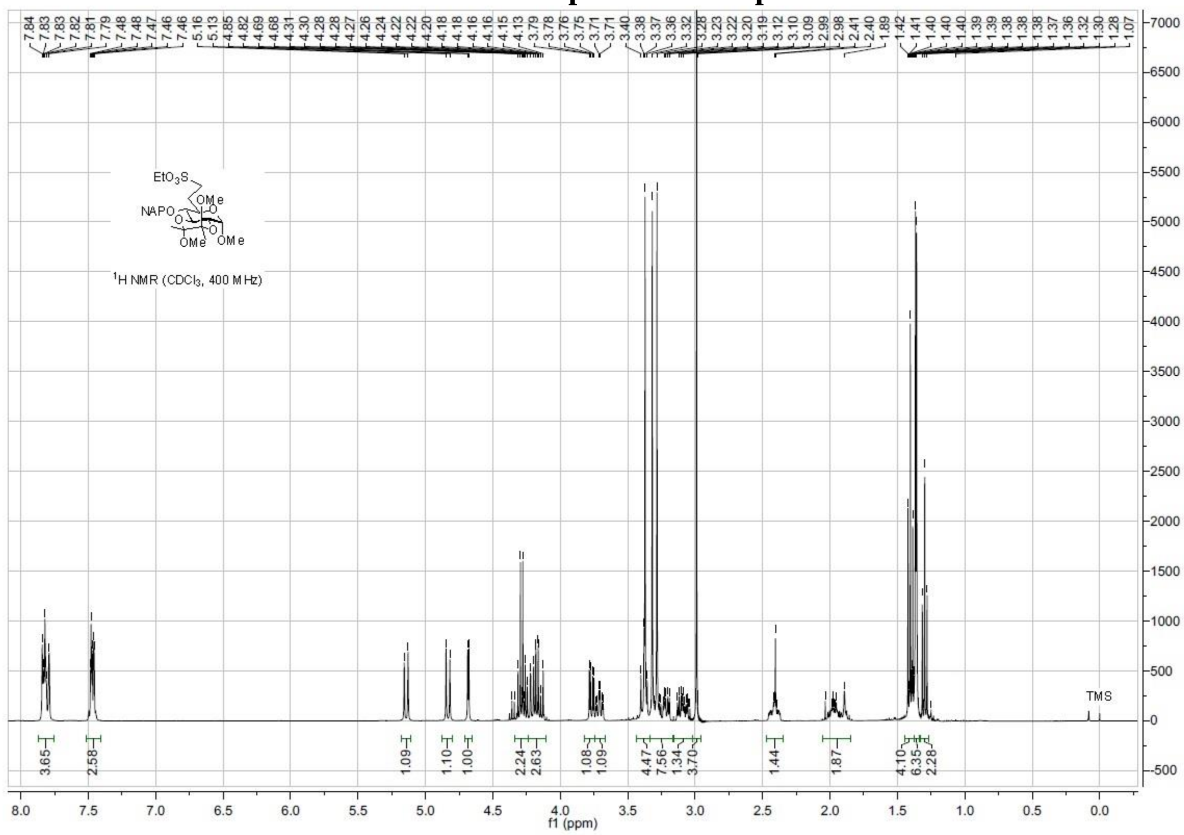

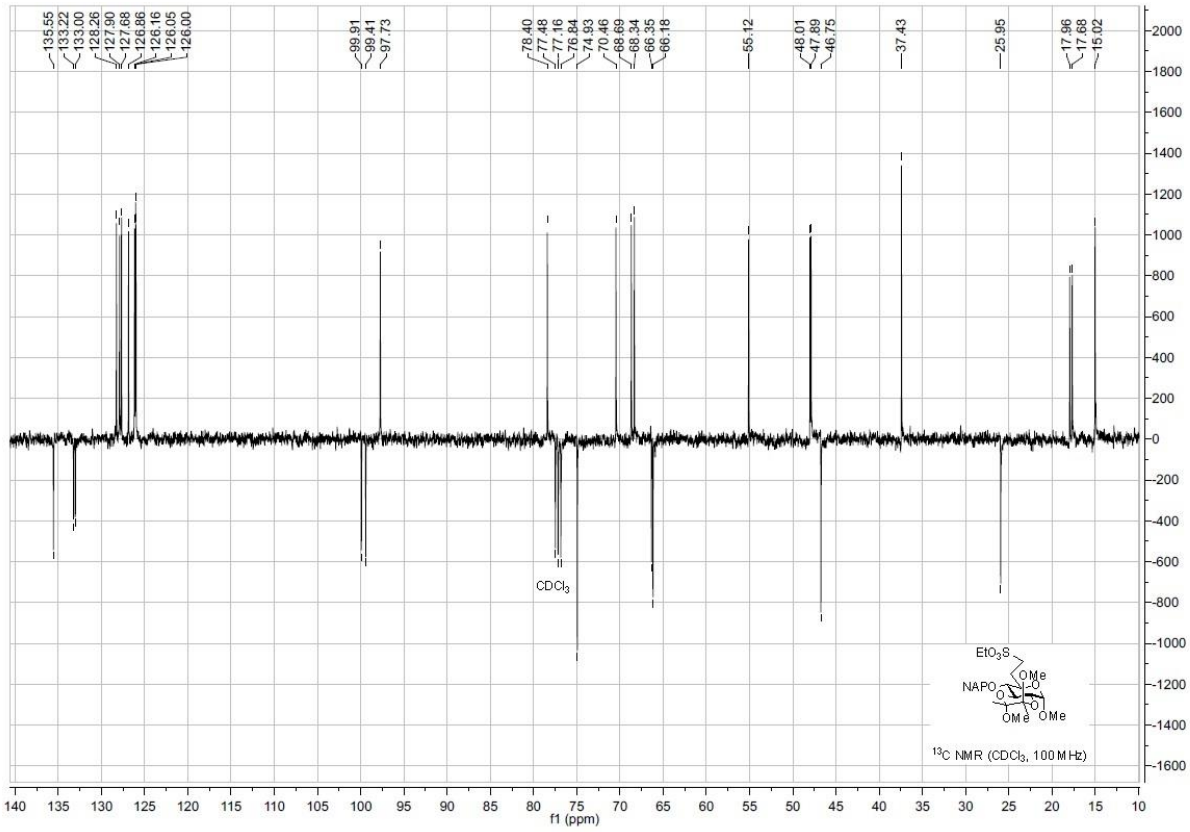

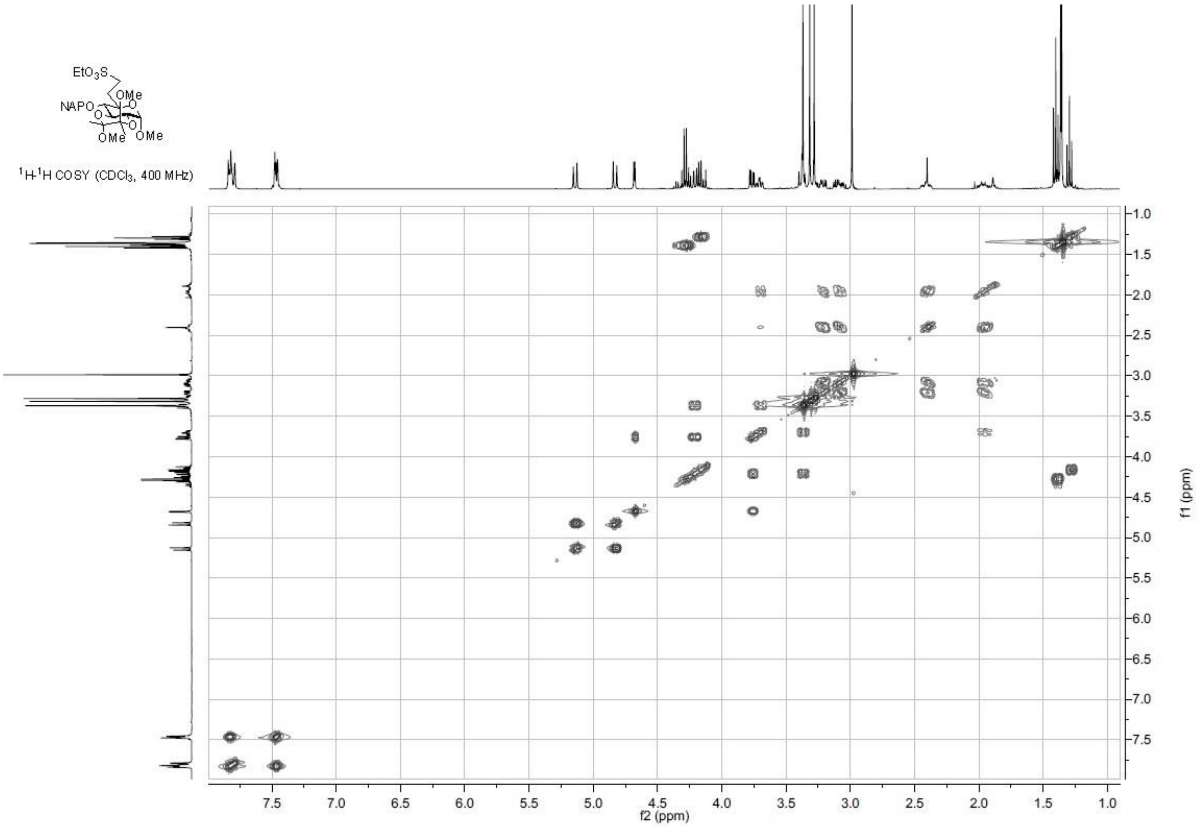
EtO3S
NAPO
OMe
OMe
OMe
1H-1H COSY (CDCl3, 400 MHz)
f2 (ppm)
f1 (ppm)

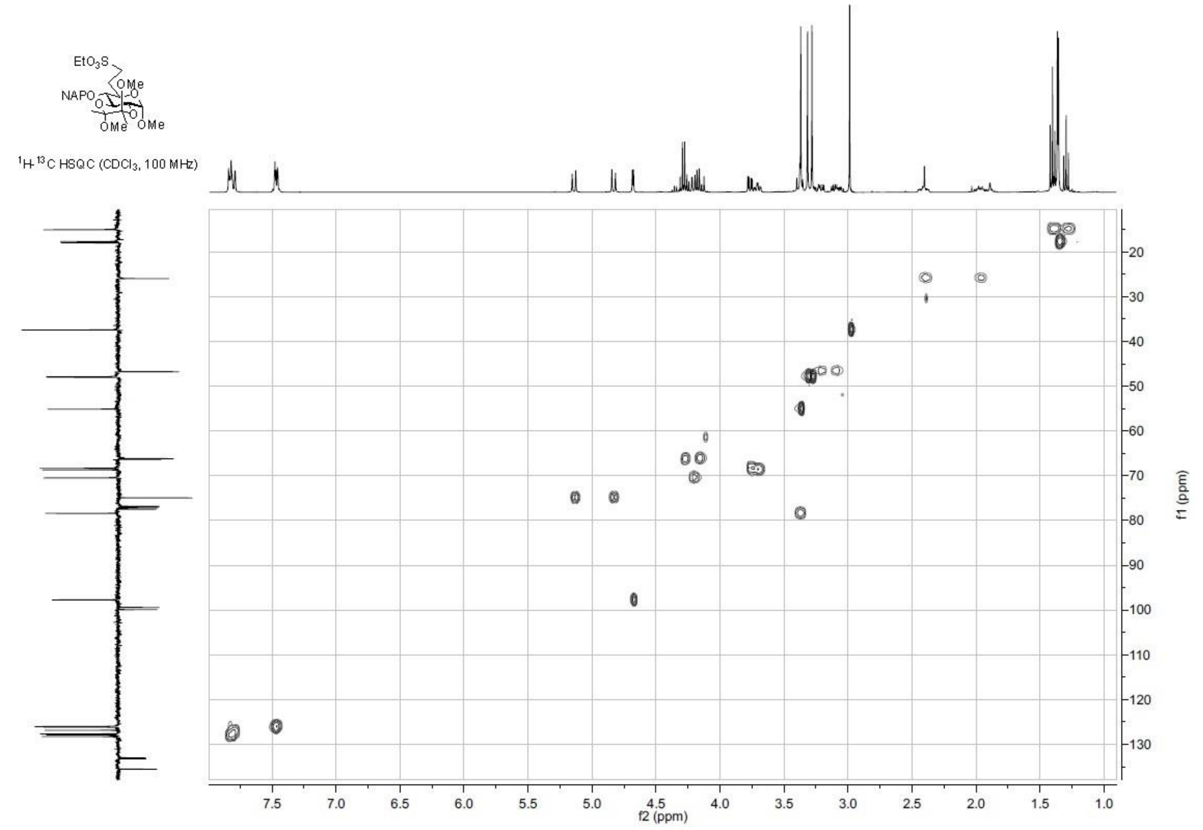
EtO3S
NAPO
OMe
OMe
OMe
1H-13C HSQC (CDCl3, 100 MHz)
f2 (ppm)
f1 (ppm)

^{1}H and ^{13}C NMR spectra of compound 59

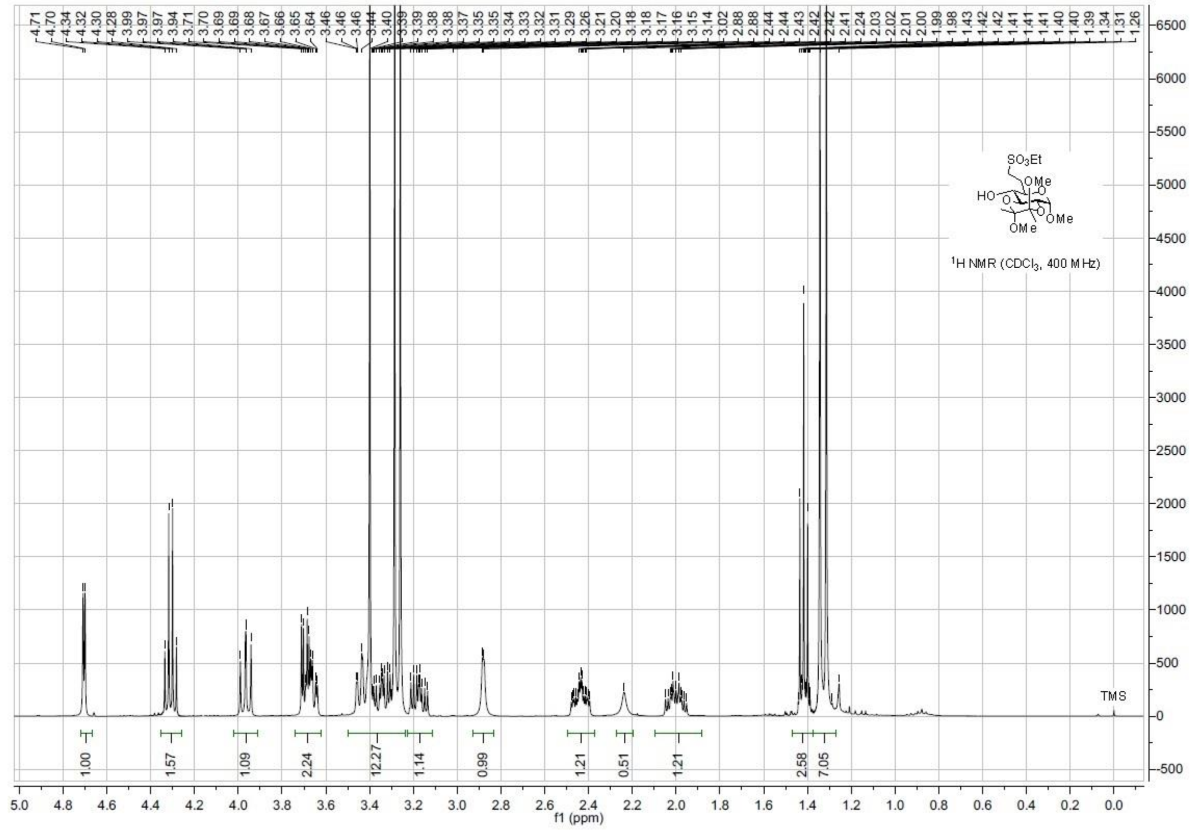

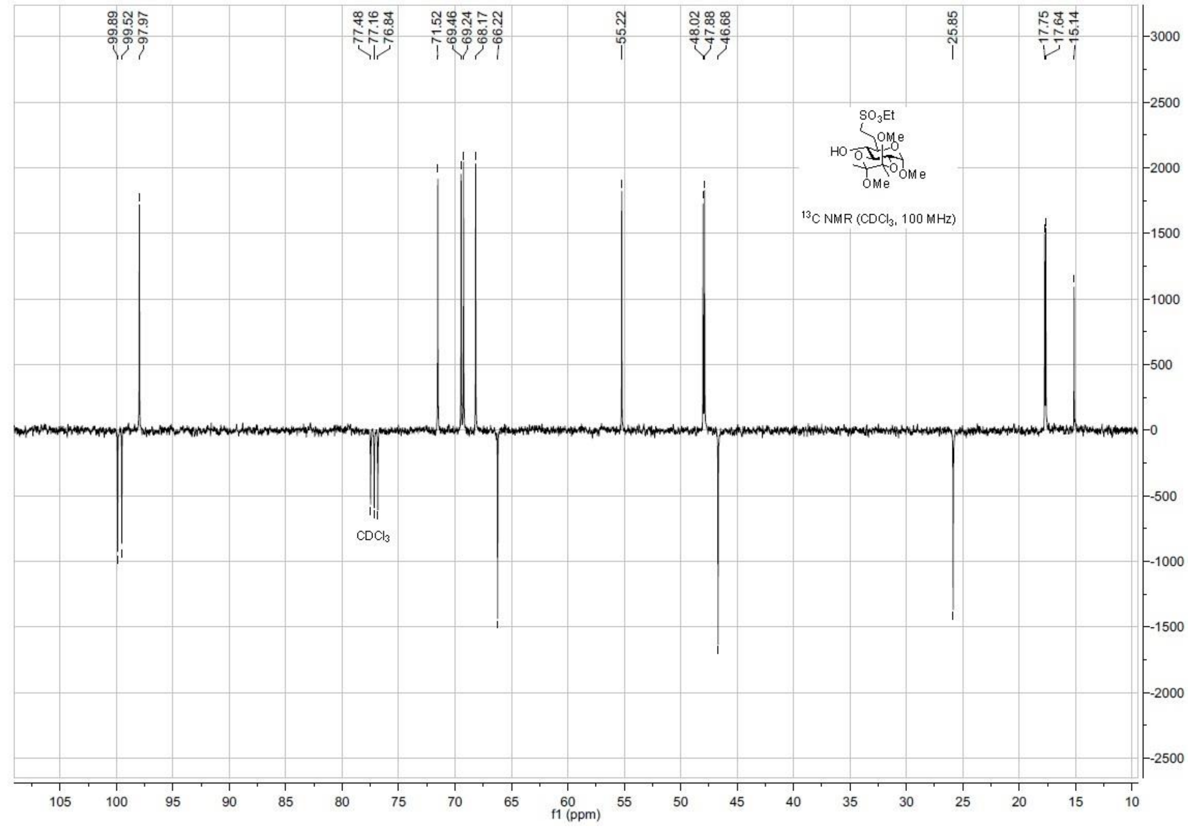

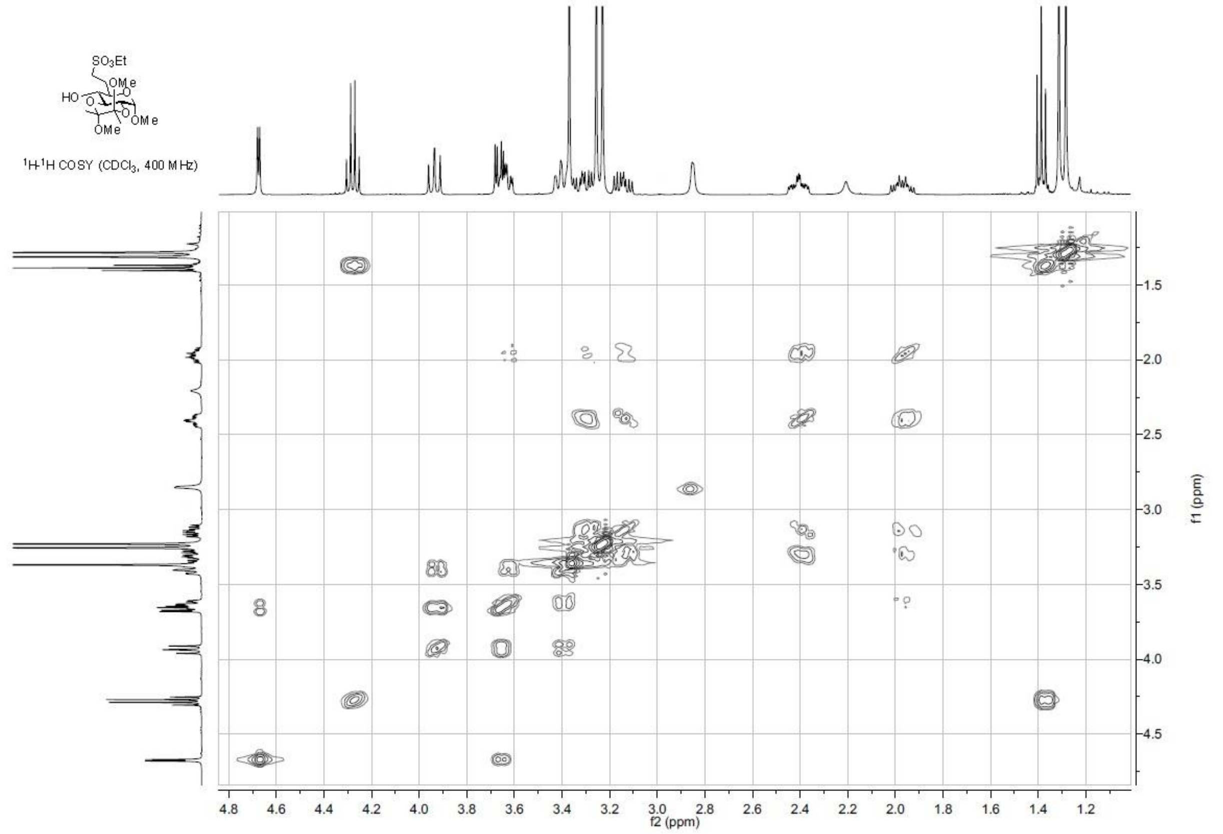
SO3Et
HO
OMe
OMe
OMe
OMe
1H-1H COSY (CDCl3, 400 MHz)
f2 (ppm)
f1 (ppm)

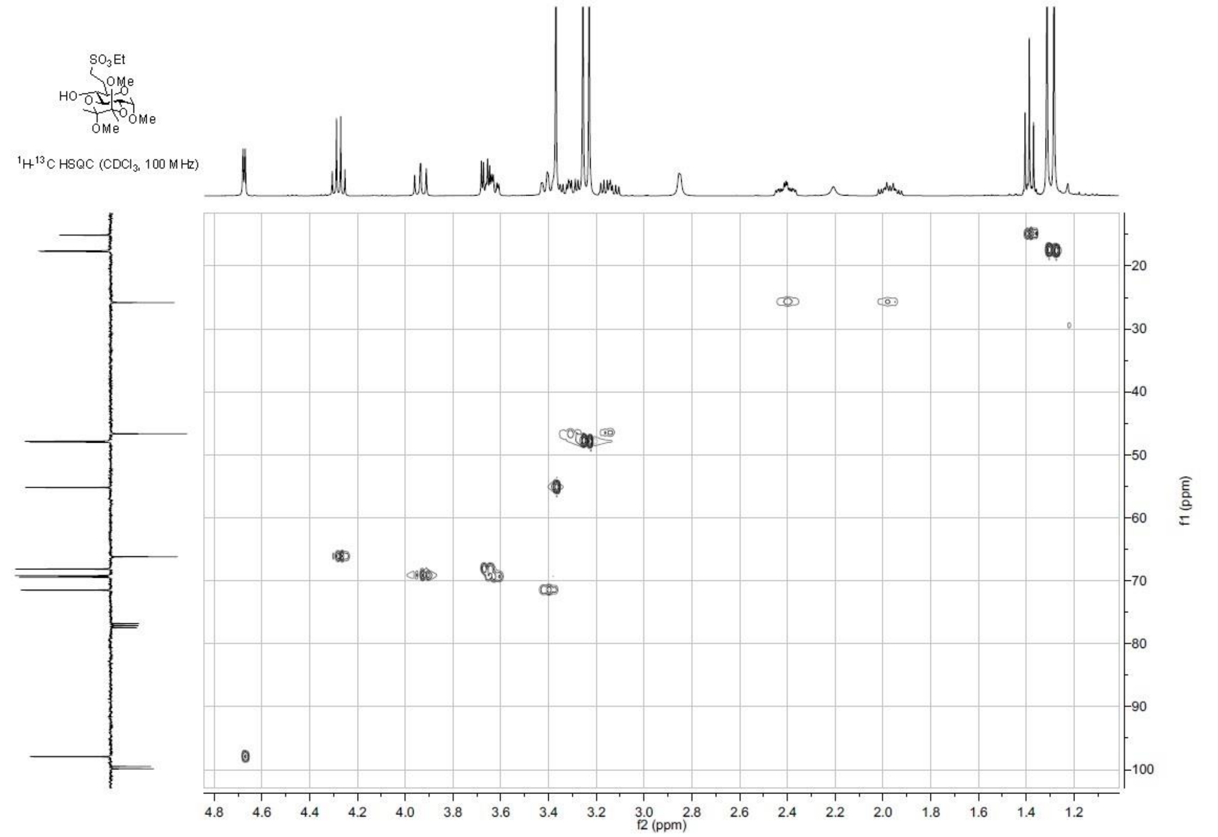
SO3Et
HO
OMe
OMe
OMe
OMe
1H-13C HSQC (CDCl3, 100 MHz)
f2 (ppm)
f1 (ppm)

^{1}H and ^{13}C NMR spectra of compound 60

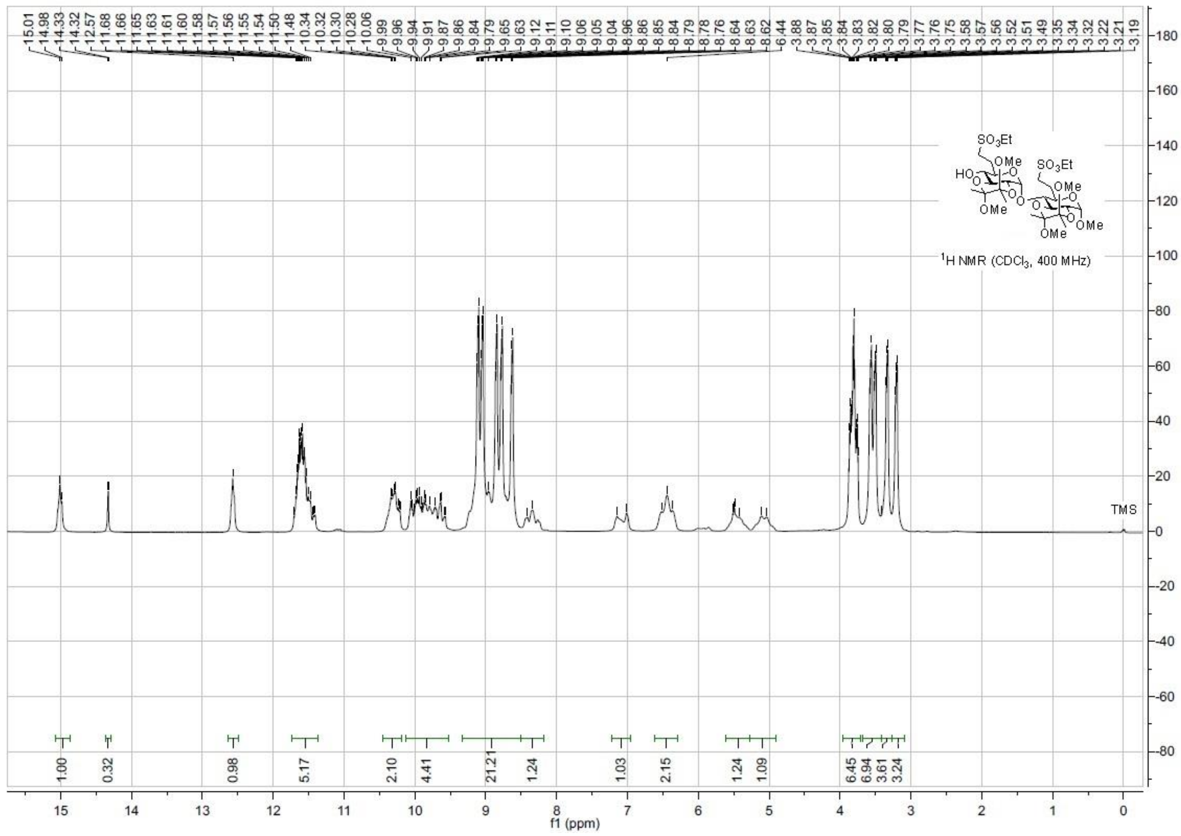

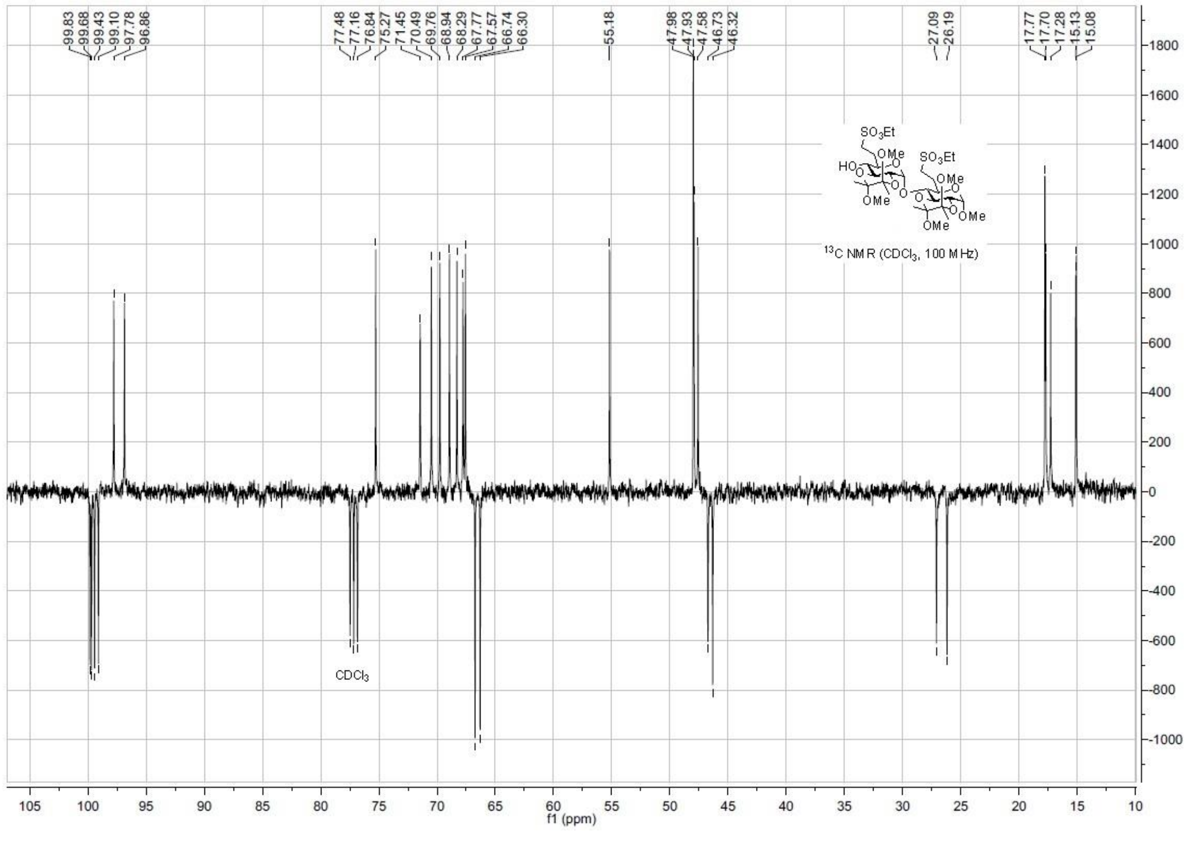

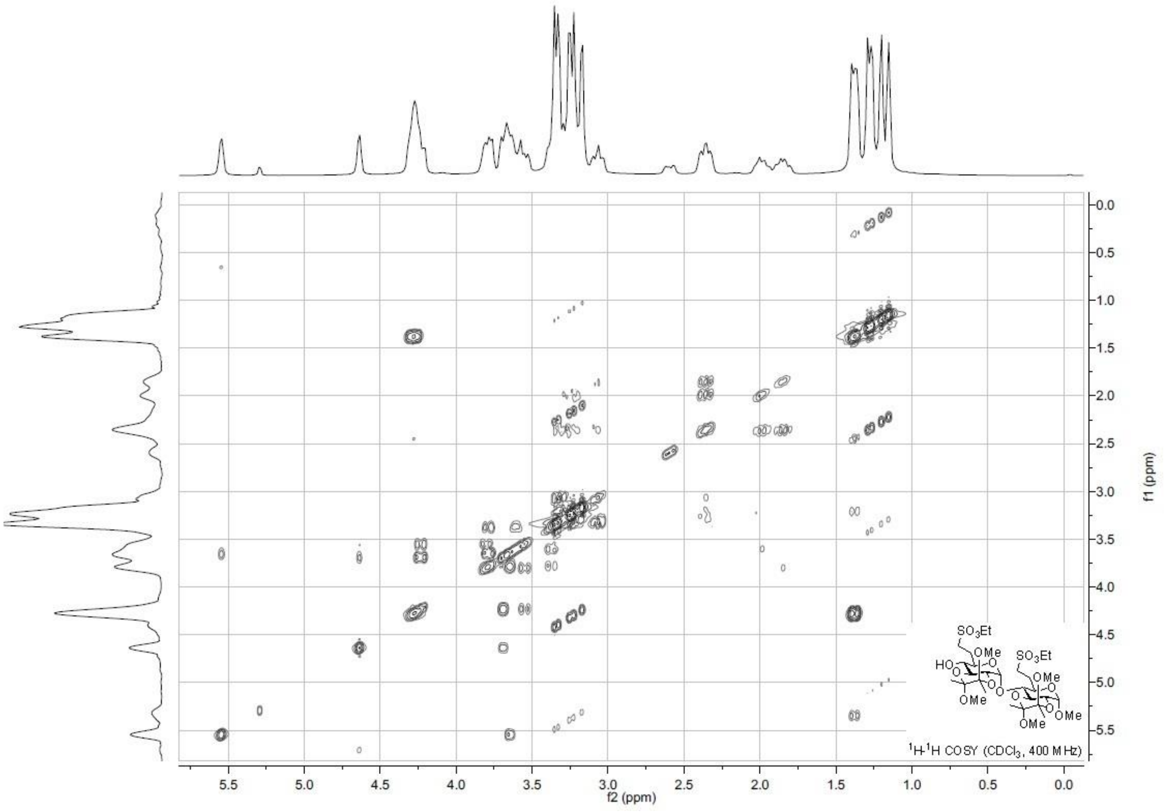

SO3Et
HO
OMe
SO3Et
OMe
OMe
OMe
OMe
1H-1H COSY (CDCl3, 400 MHz)
f1 (ppm)
f2 (ppm)

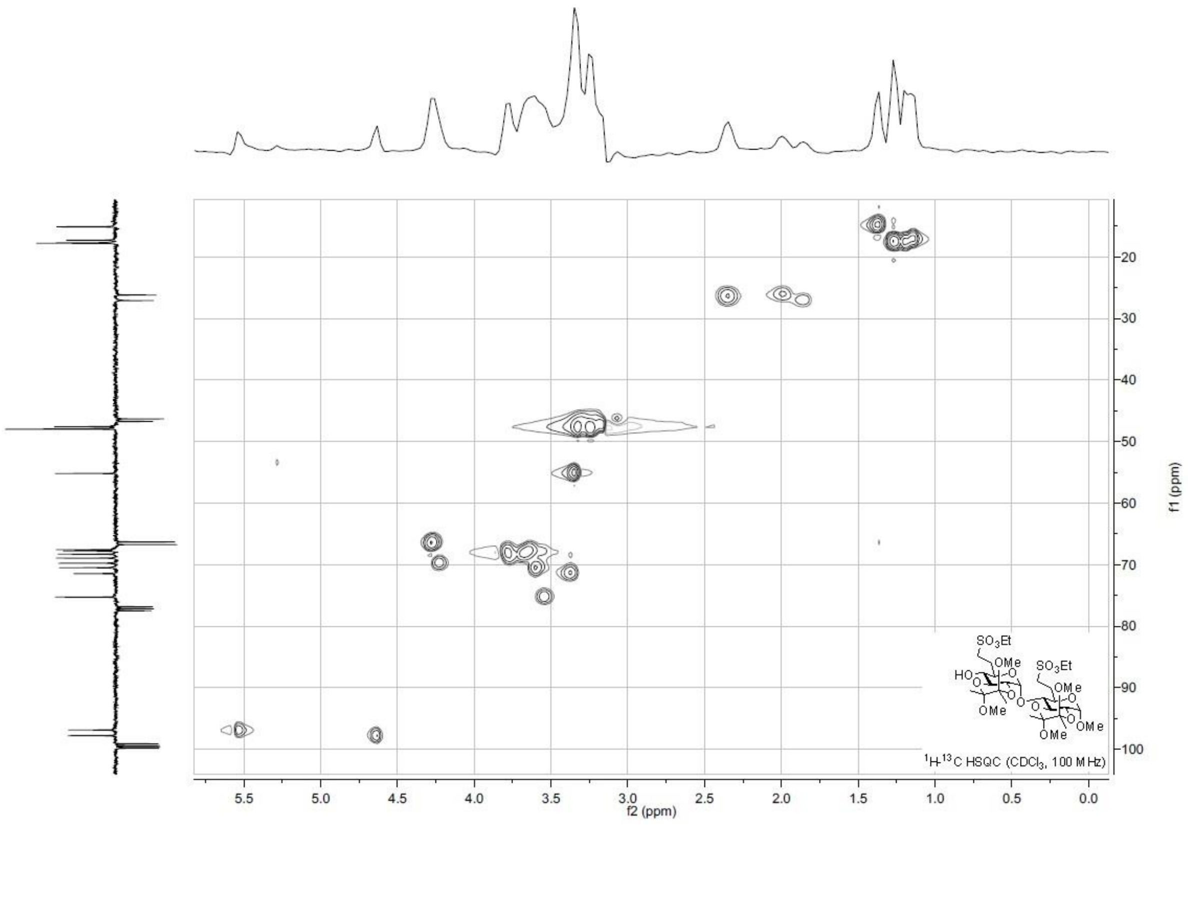

SO3Et
HO
OMe
SO3Et
OMe
OMe
OMe
OMe
1H-13C HSQC (CDCl3, 100 MHz)
f1 (ppm)
f2 (ppm)

^{1}H and ^{13}C NMR spectra of compound 61

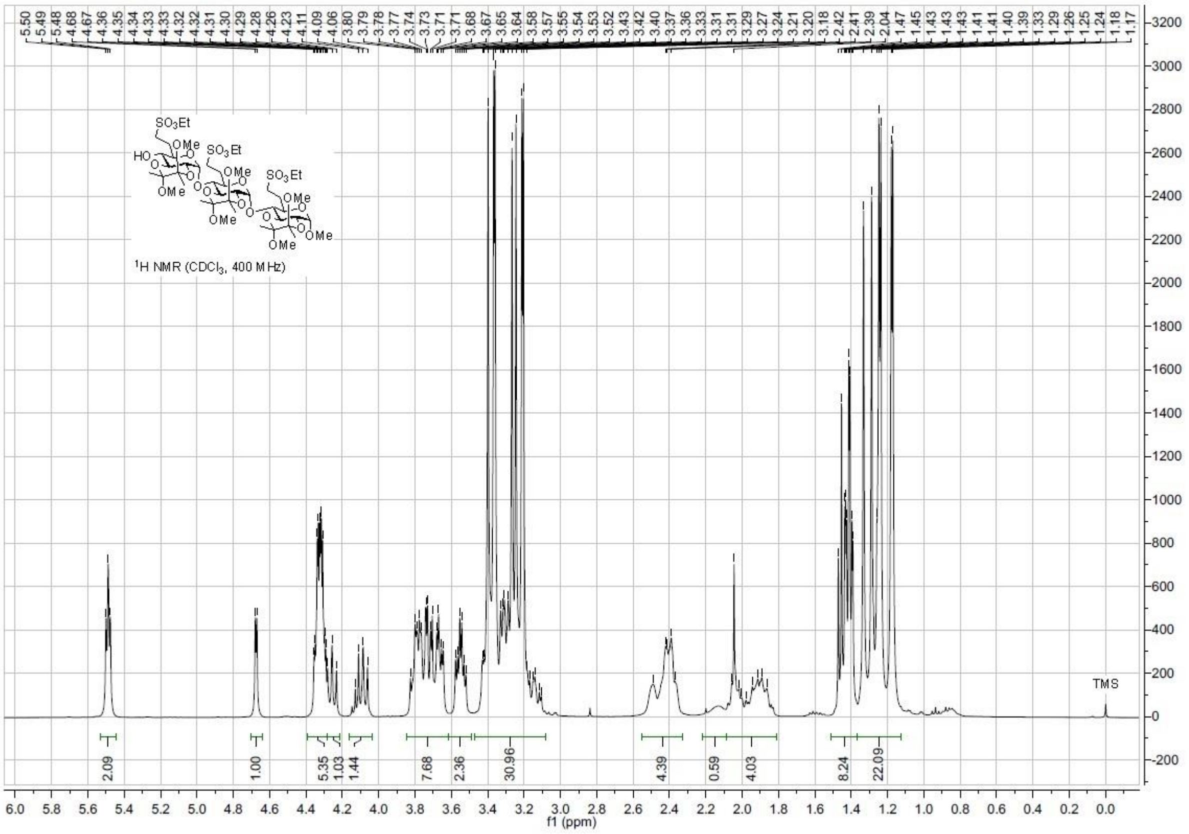

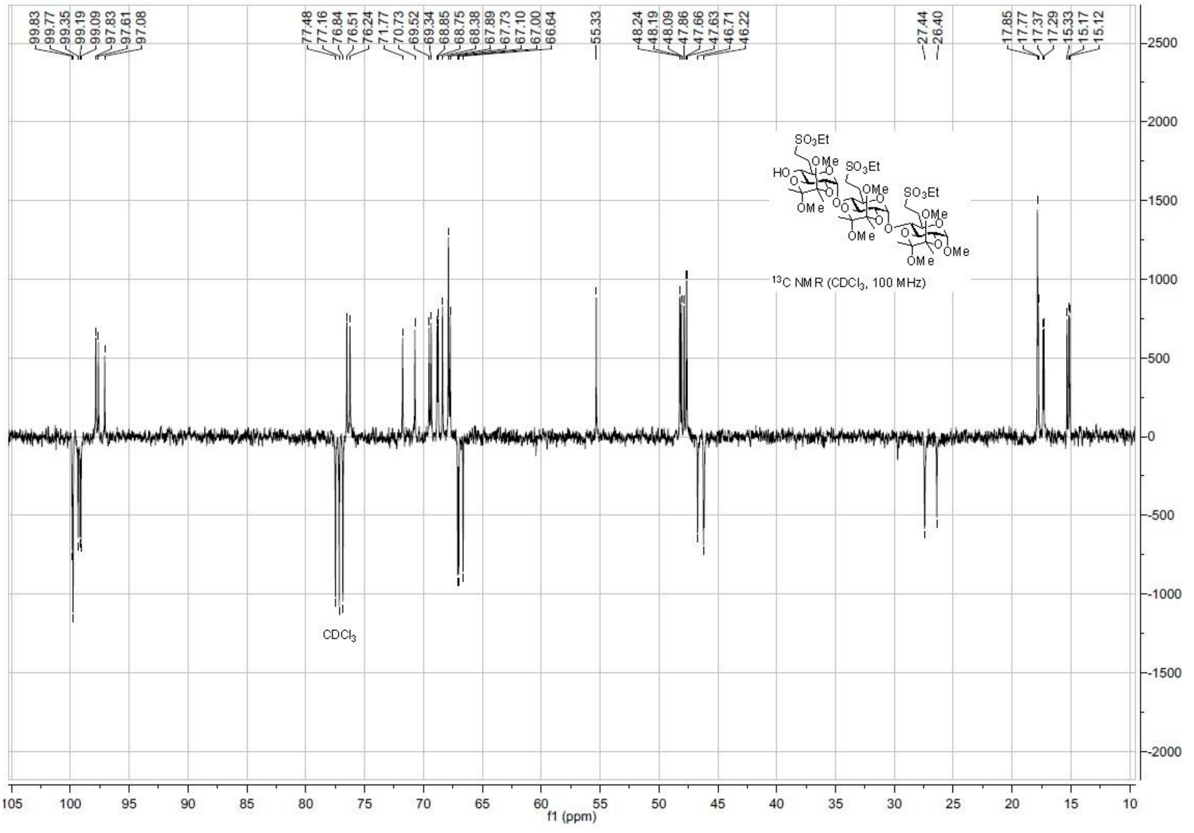

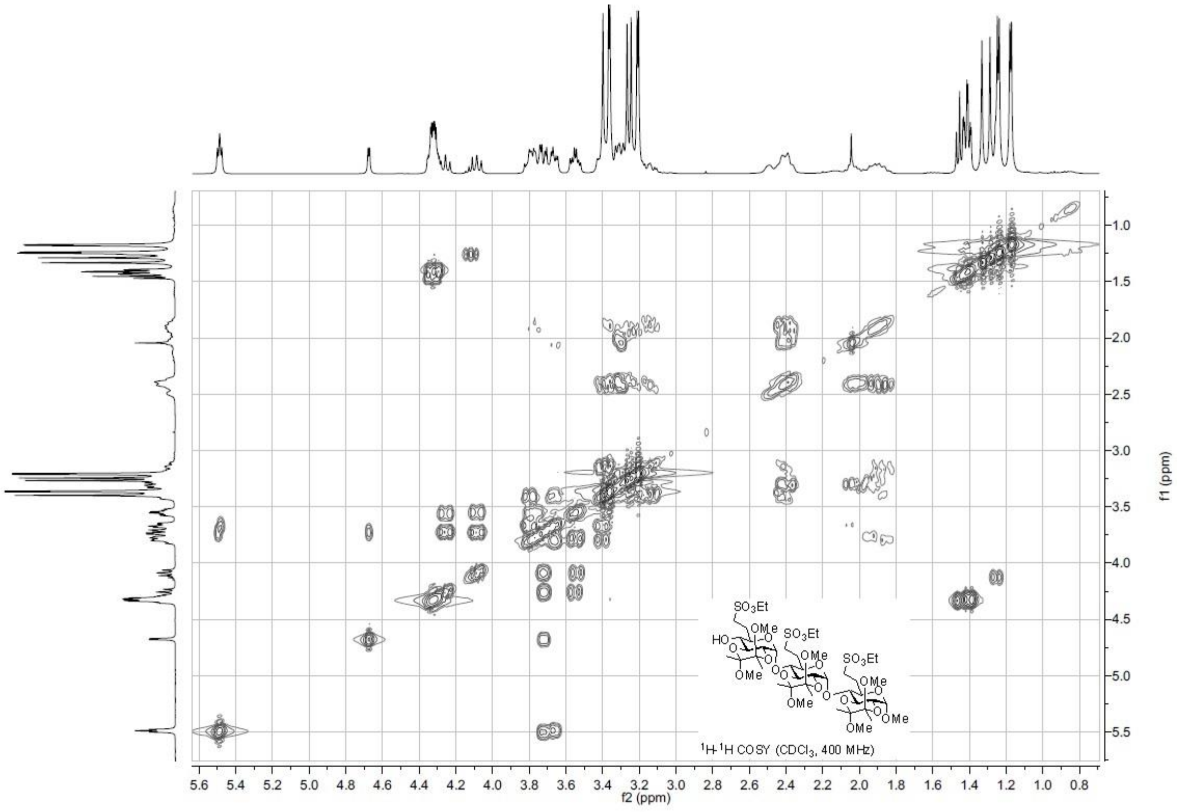
^{1}H-^{1}H COSY (CDCl$_3$, 400 MHz)
f2 (ppm)
f1 (ppm)

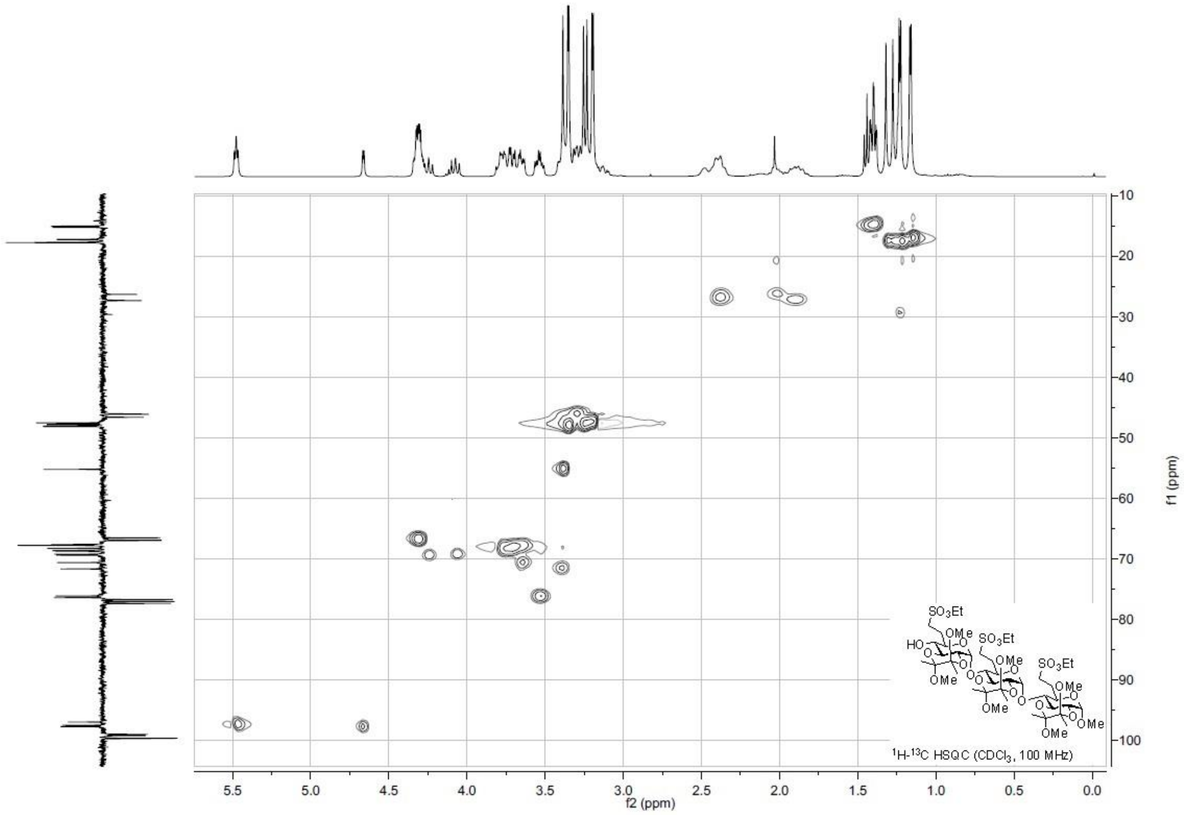
^{1}H-^{13}C HSQC (CDCl$_3$, 100 MHz)
f2 (ppm)
f1 (ppm)

^{1}H and ^{13}C NMR spectra of compound 72

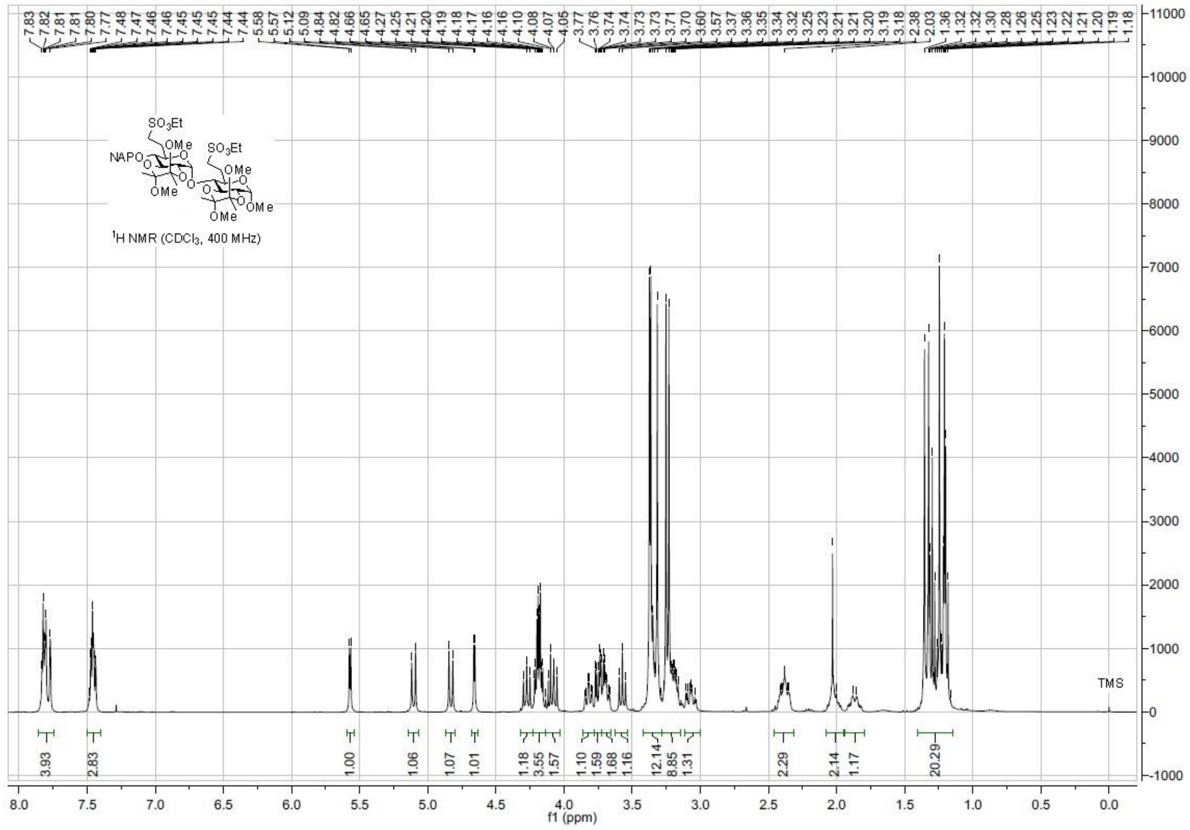

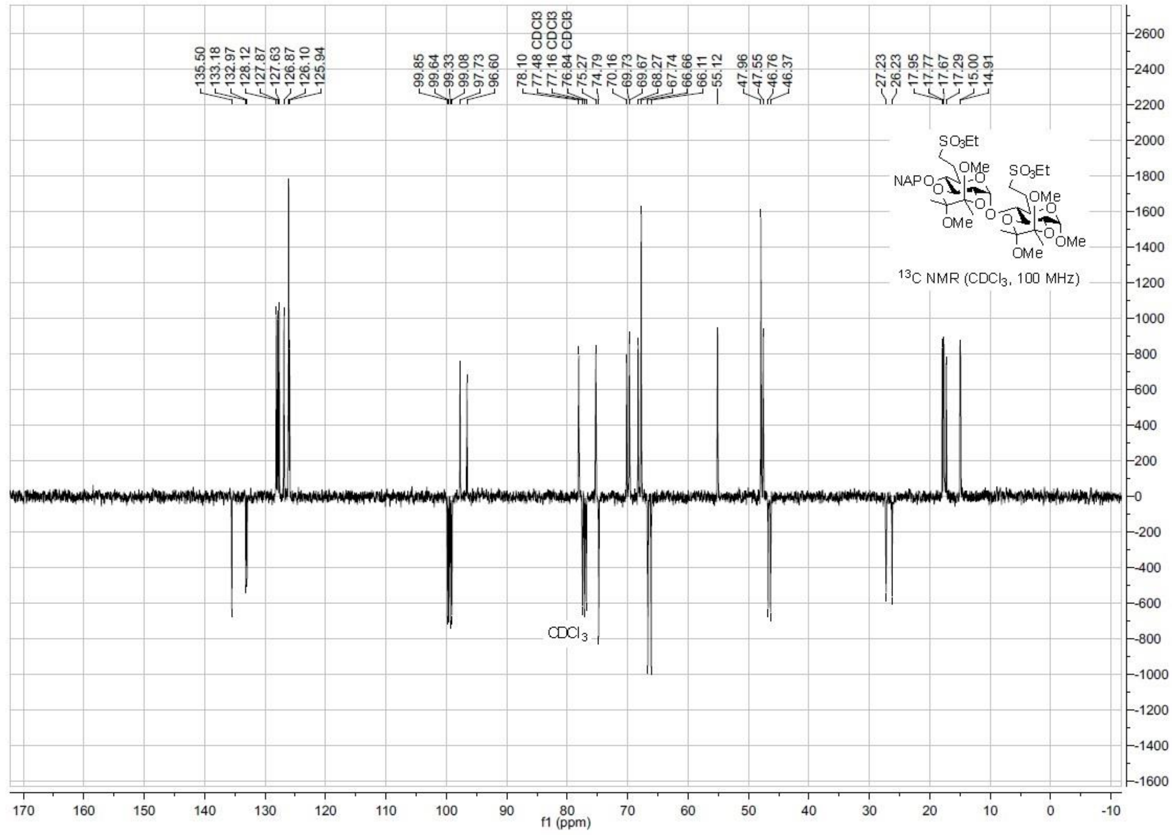

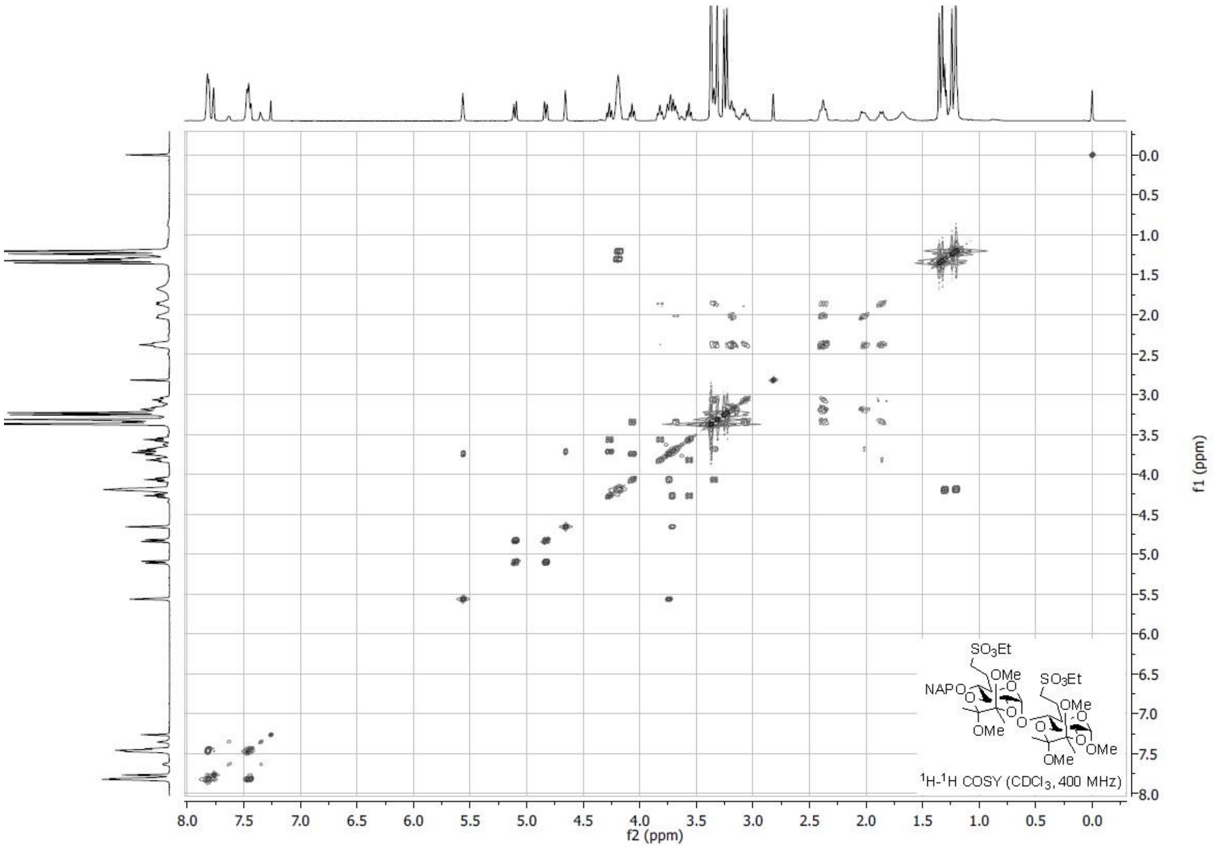

NAPO
SO3Et
OMe
O
O
SO3Et
OMe
O
OMe
OMe
OMe
1H-1H COSY (CDCl3, 400 MHz)
f2 (ppm)
f1 (ppm)

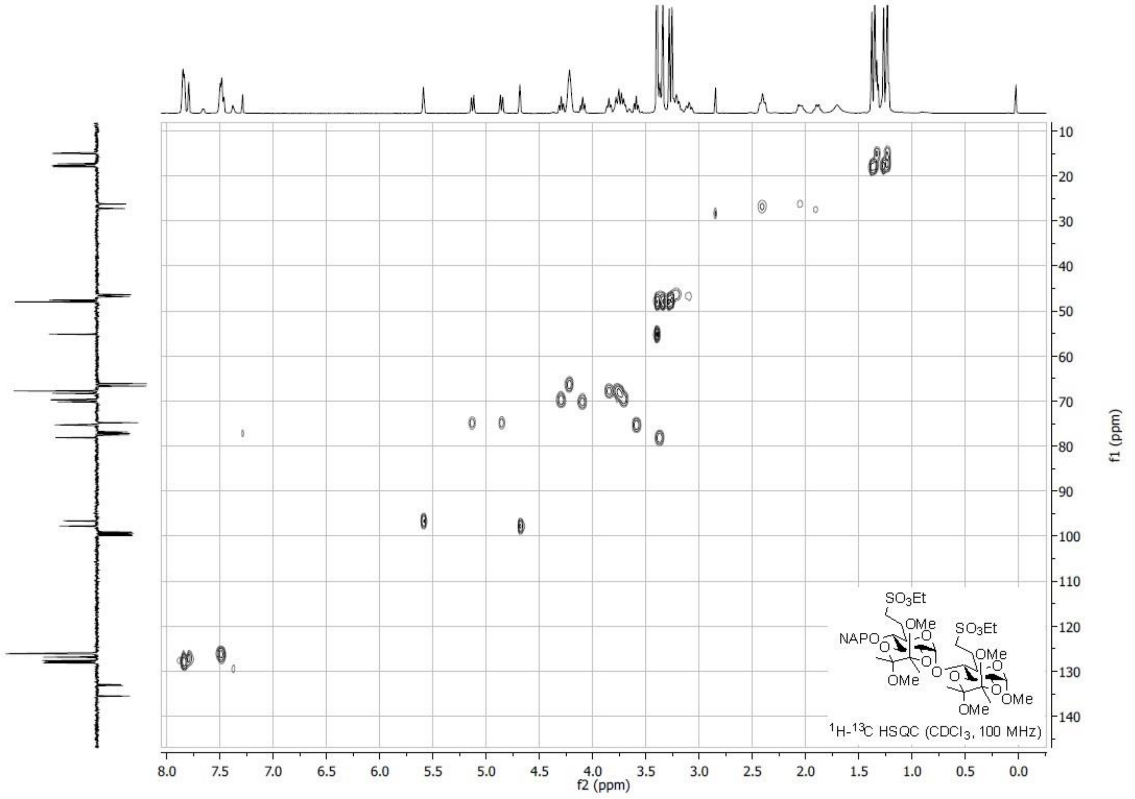

NAPO
SO3Et
OMe
O
O
SO3Et
OMe
O
OMe
OMe
OMe
1H-13C HSQC (CDCl3, 100 MHz)
f2 (ppm)
f1 (ppm)

^{1}H and ^{13}C NMR spectra of compound 73

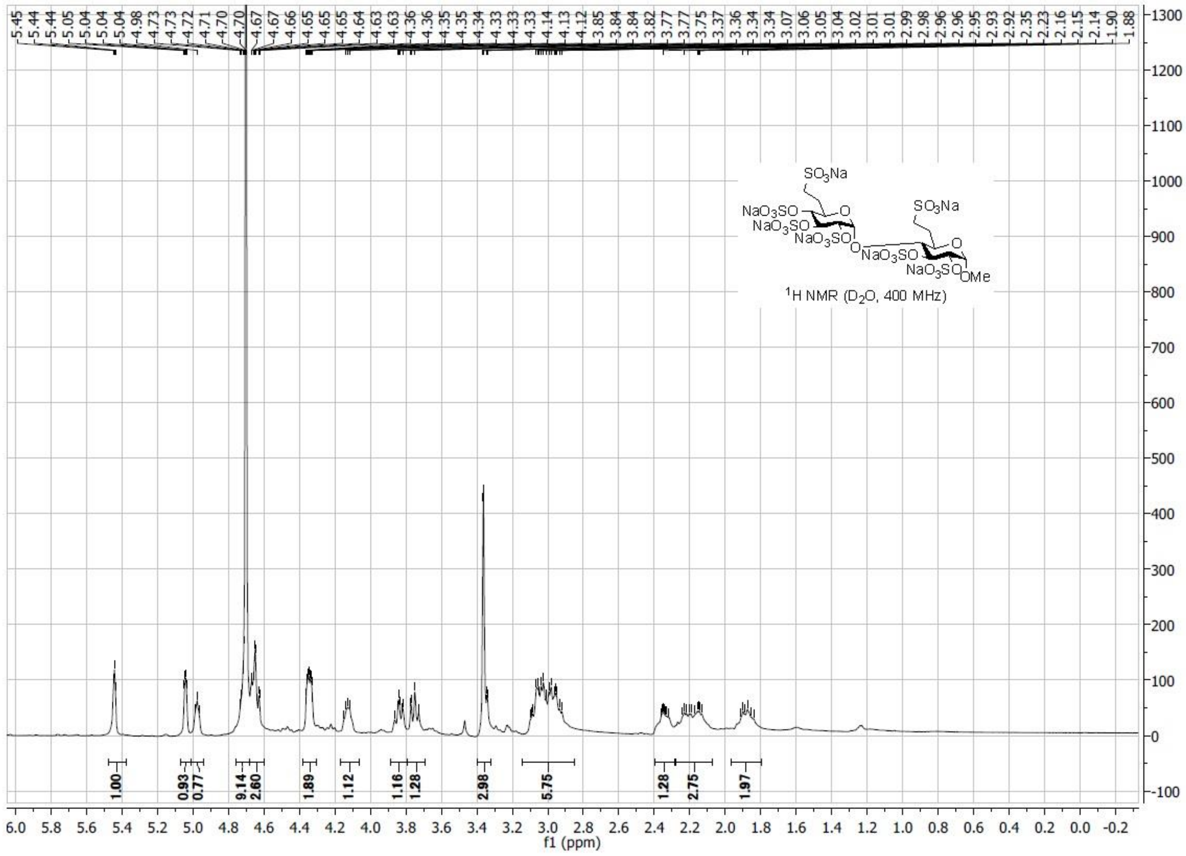

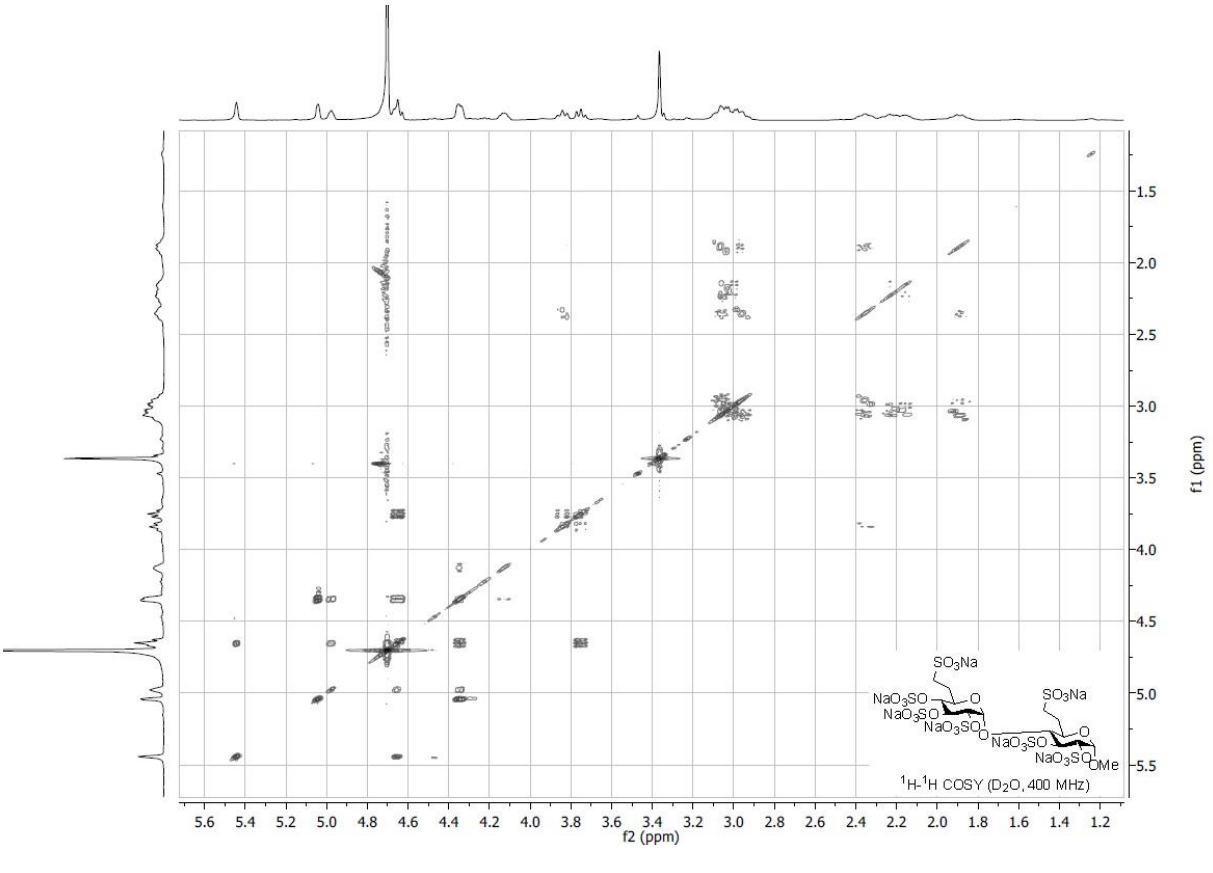

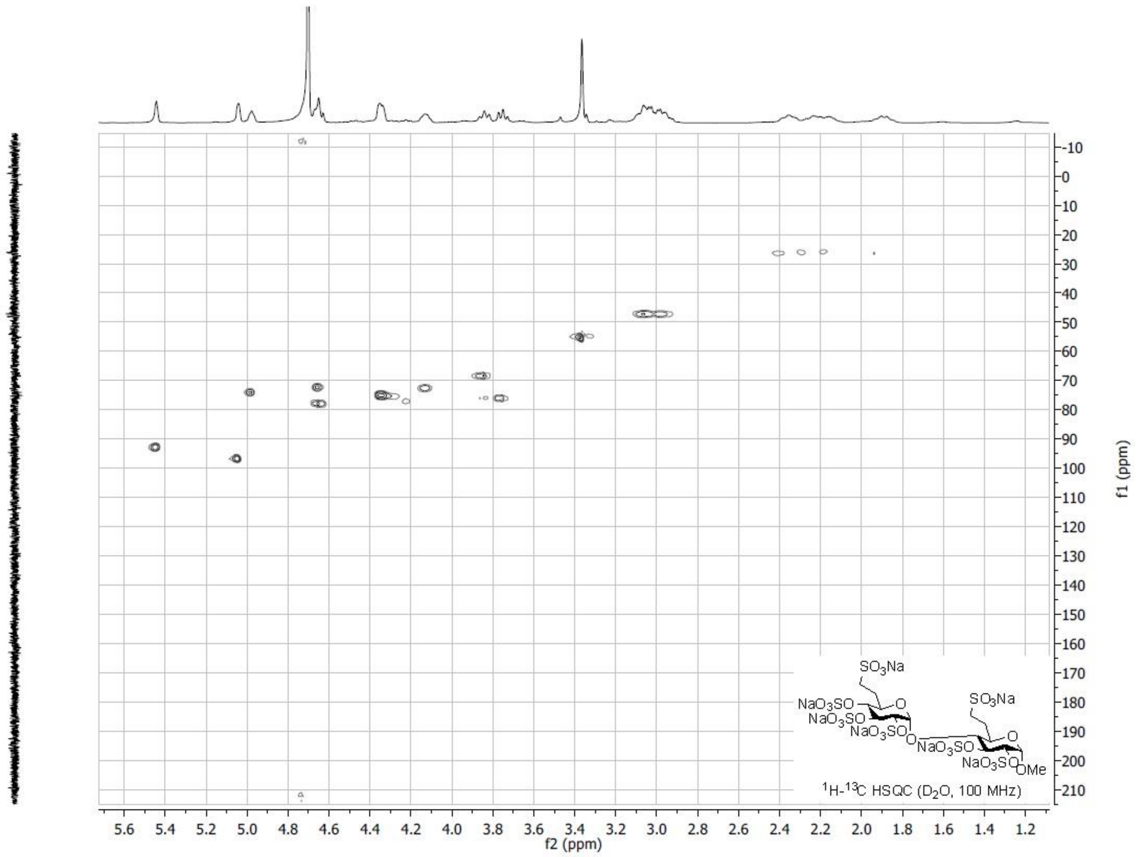
SO3Na
NaO3SO
NaO3SO
NaO3SO
O
SO3Na
NaO3SO
NaO3SO
OMe
1H-13C HSQC (D2O, 100 MHz)
f1 (ppm)
f2 (ppm)

^{1}H and ^{13}C NMR spectra of compound 74

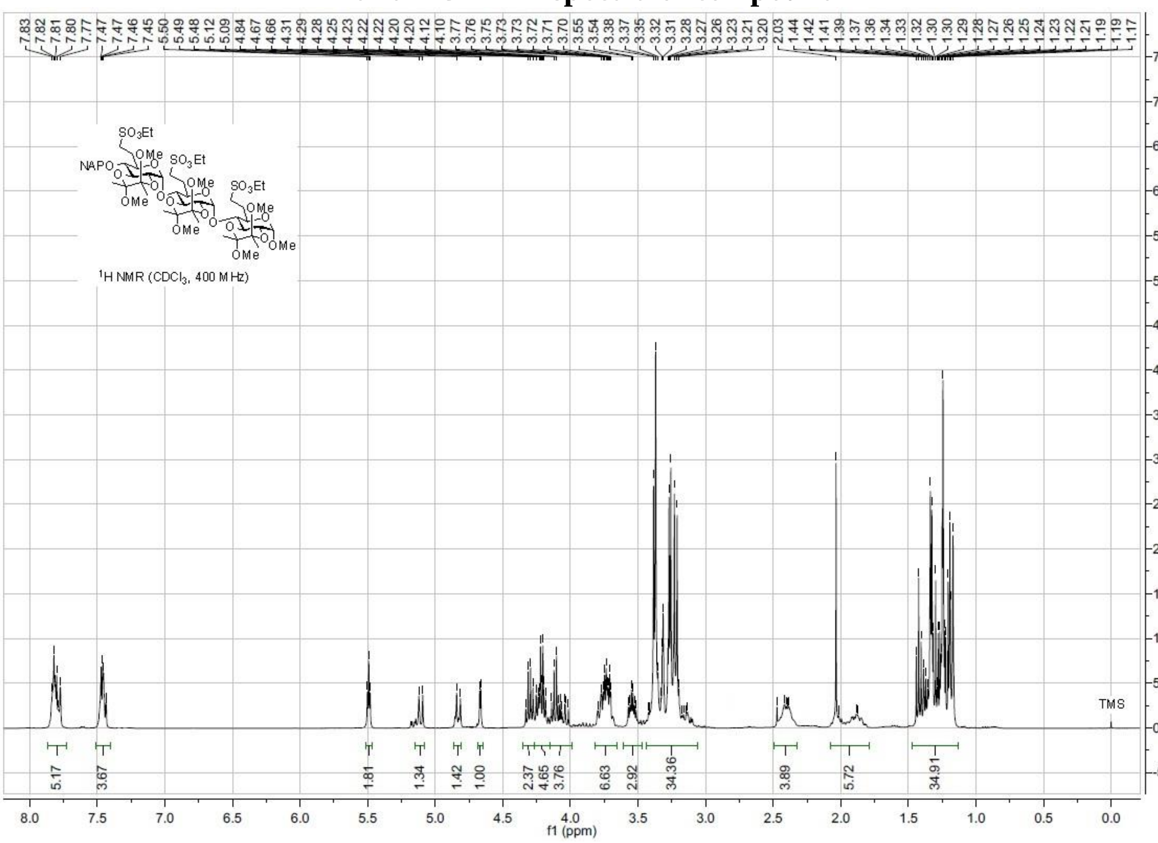

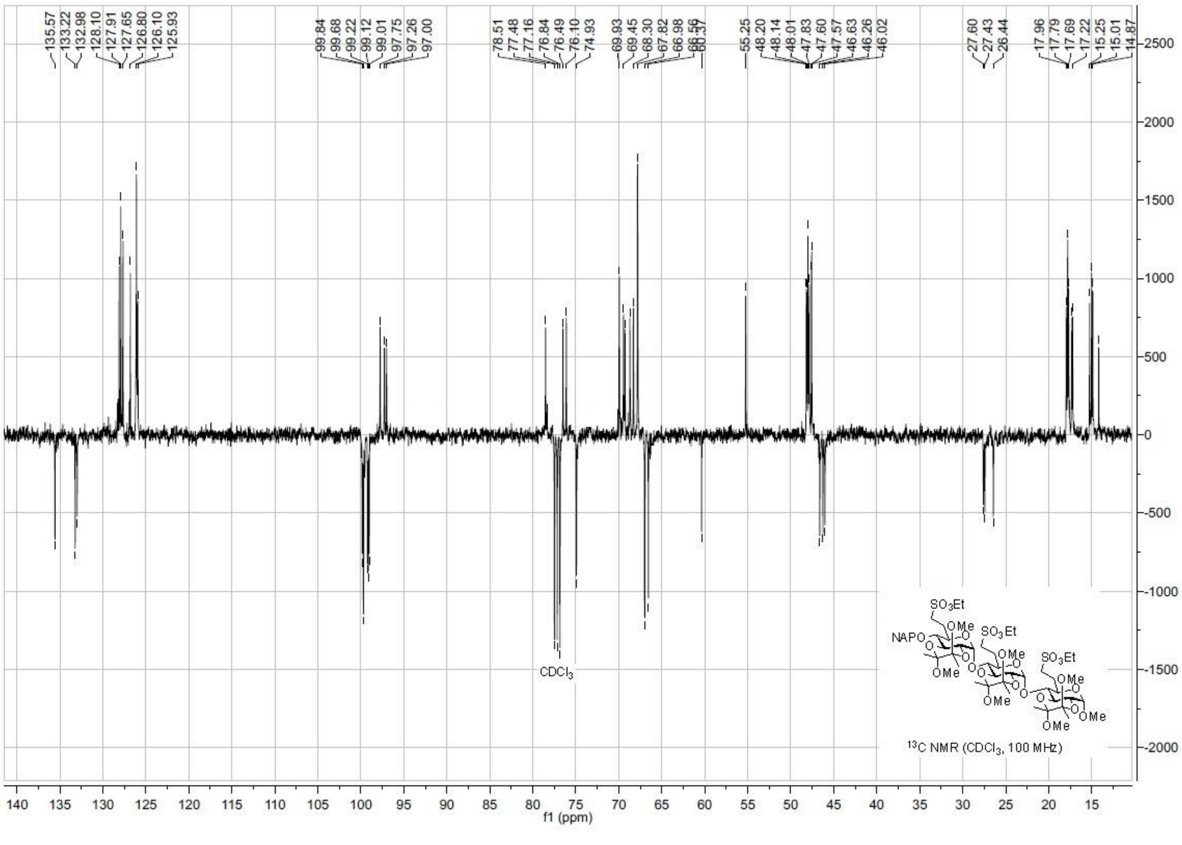

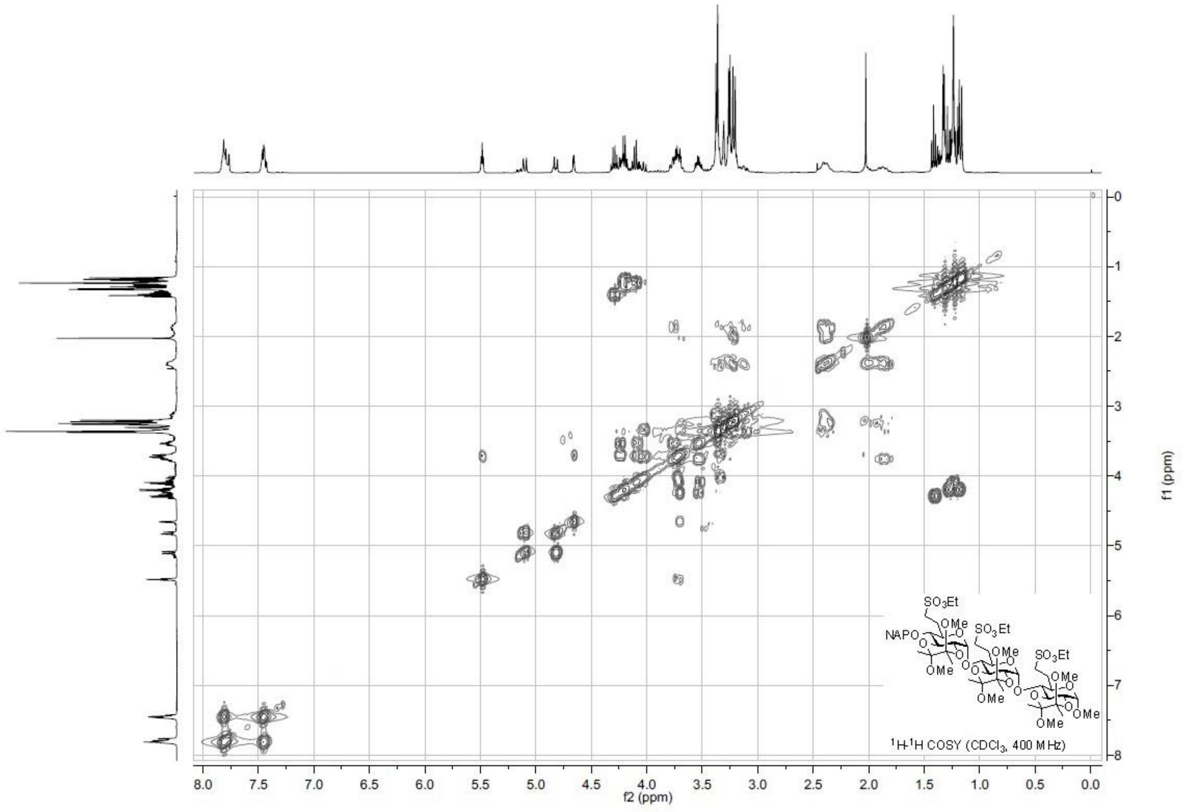

f2 (ppm)
f1 (ppm)
SO3Et
OMe
NAPO
OMe
SO3Et
OMe
OMe
SO3Et
OMe
OMe
OMe
1H-1H COSY (CDCl3, 400 MHz)

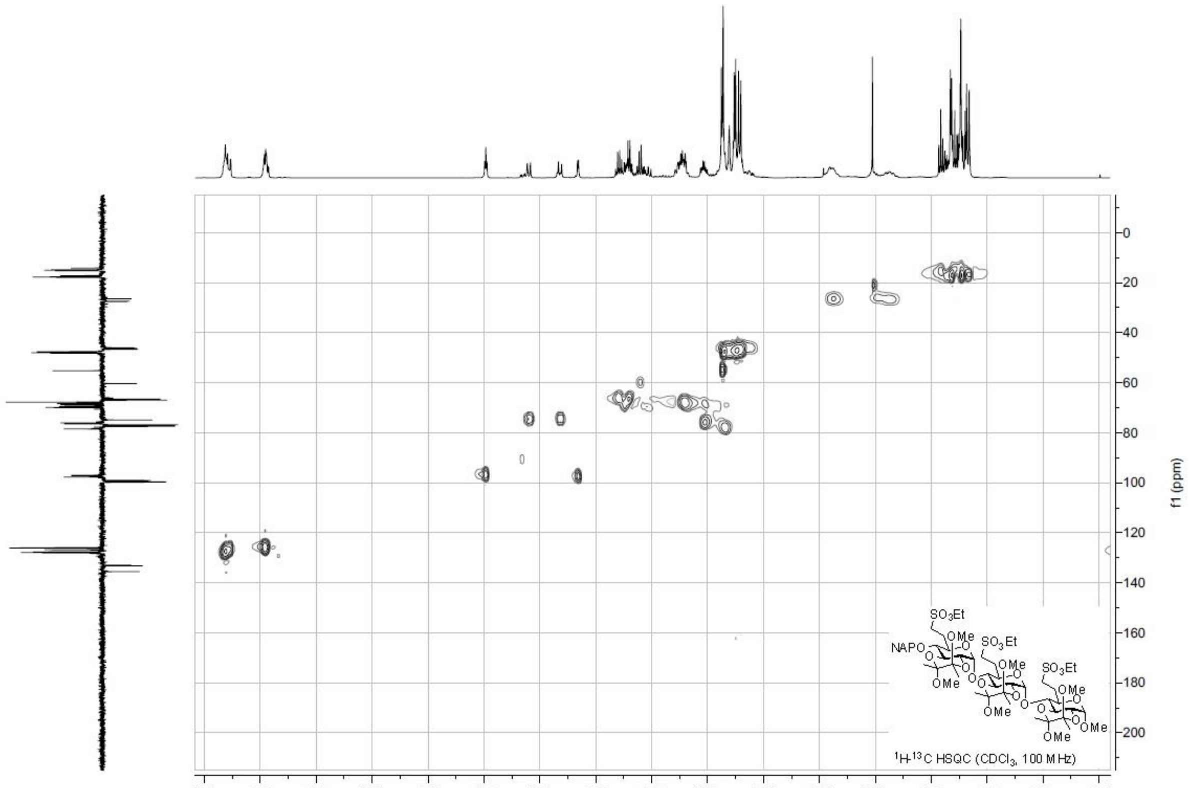

f2 (ppm)
f1 (ppm)
SO3Et
OMe
NAPO
OMe
SO3Et
OMe
OMe
SO3Et
OMe
OMe
OMe
1H-13C HSQC (CDCl3, 100 MHz)

^{1}H and ^{13}C NMR spectra of compound 75

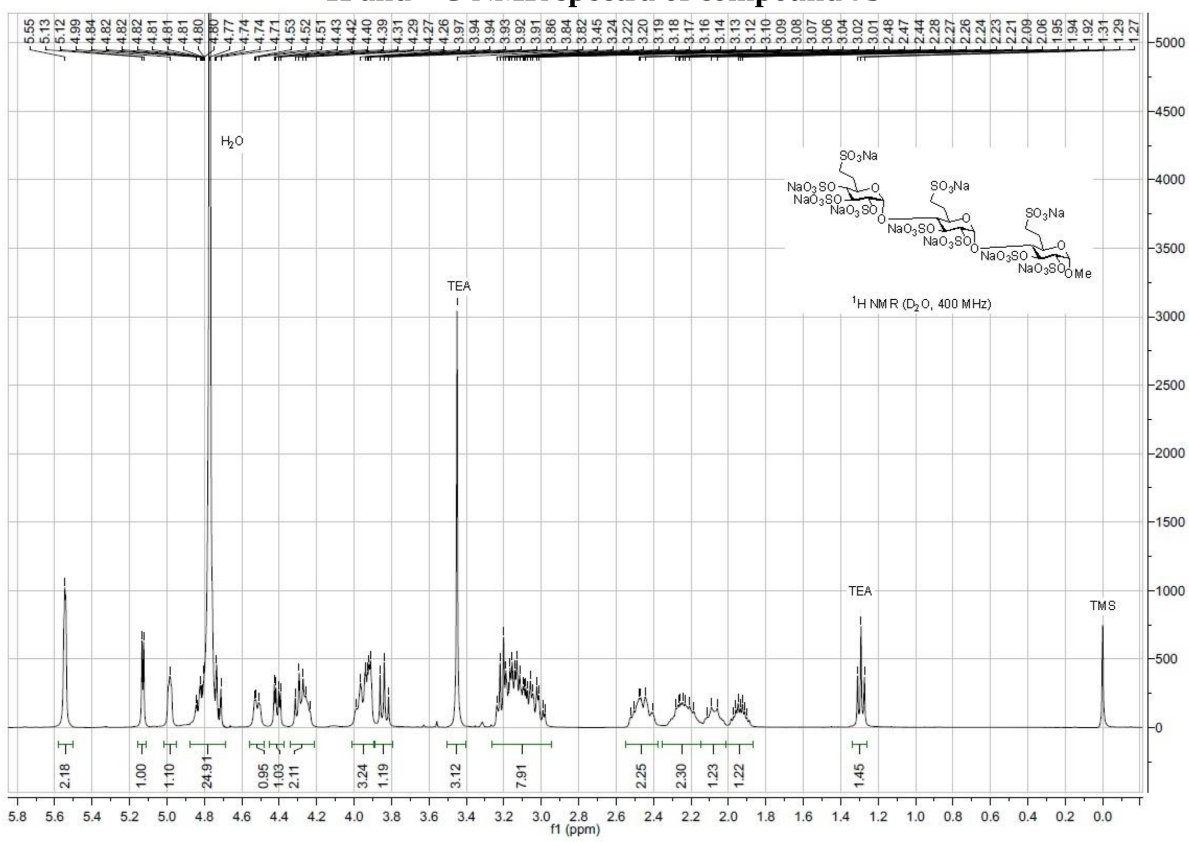

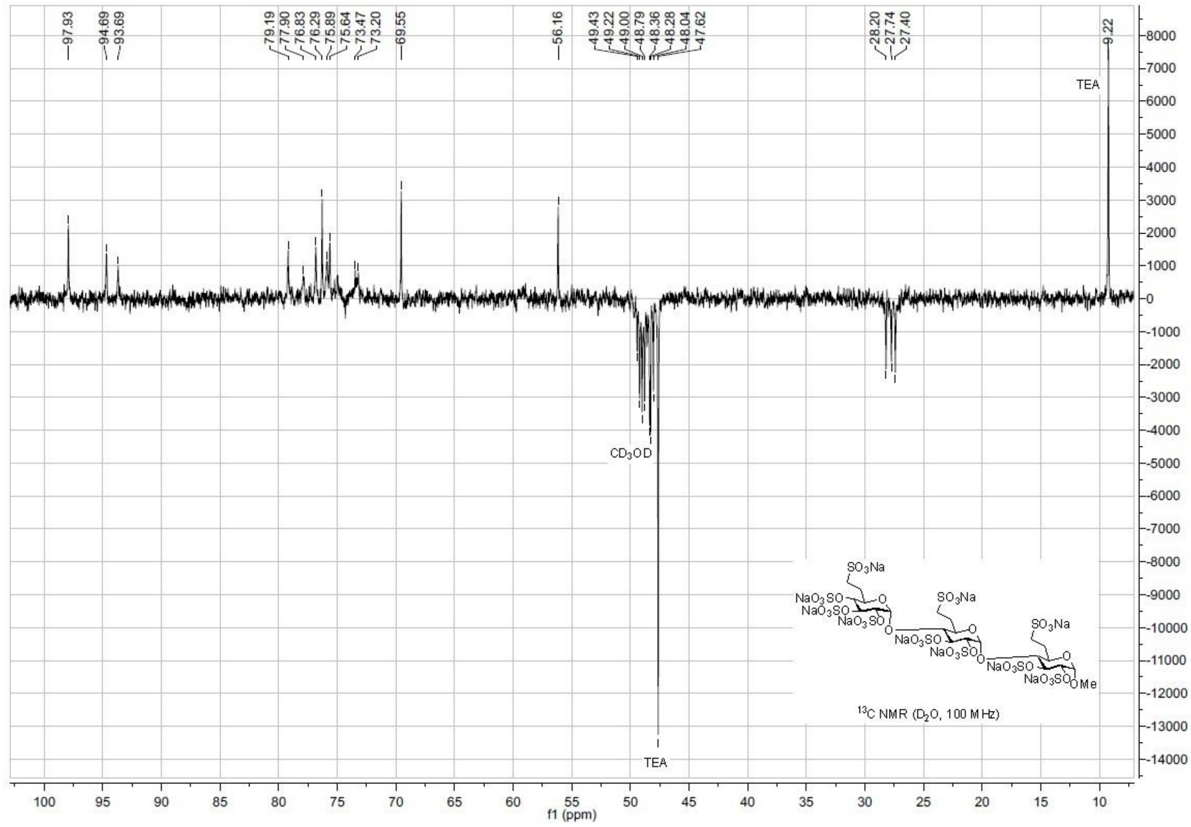

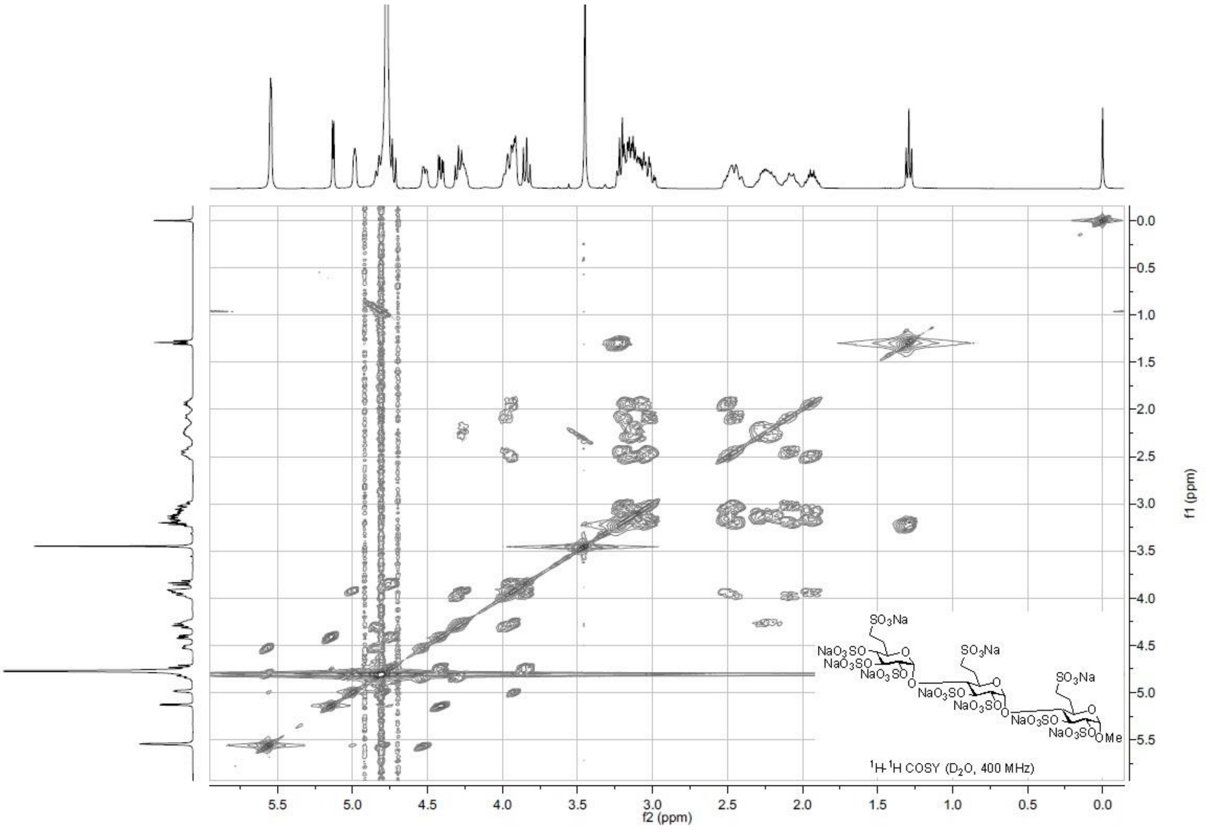

^{1}H-^{1}H COSY (D_2O, 400 MHz)
f2 (ppm)
f1 (ppm)

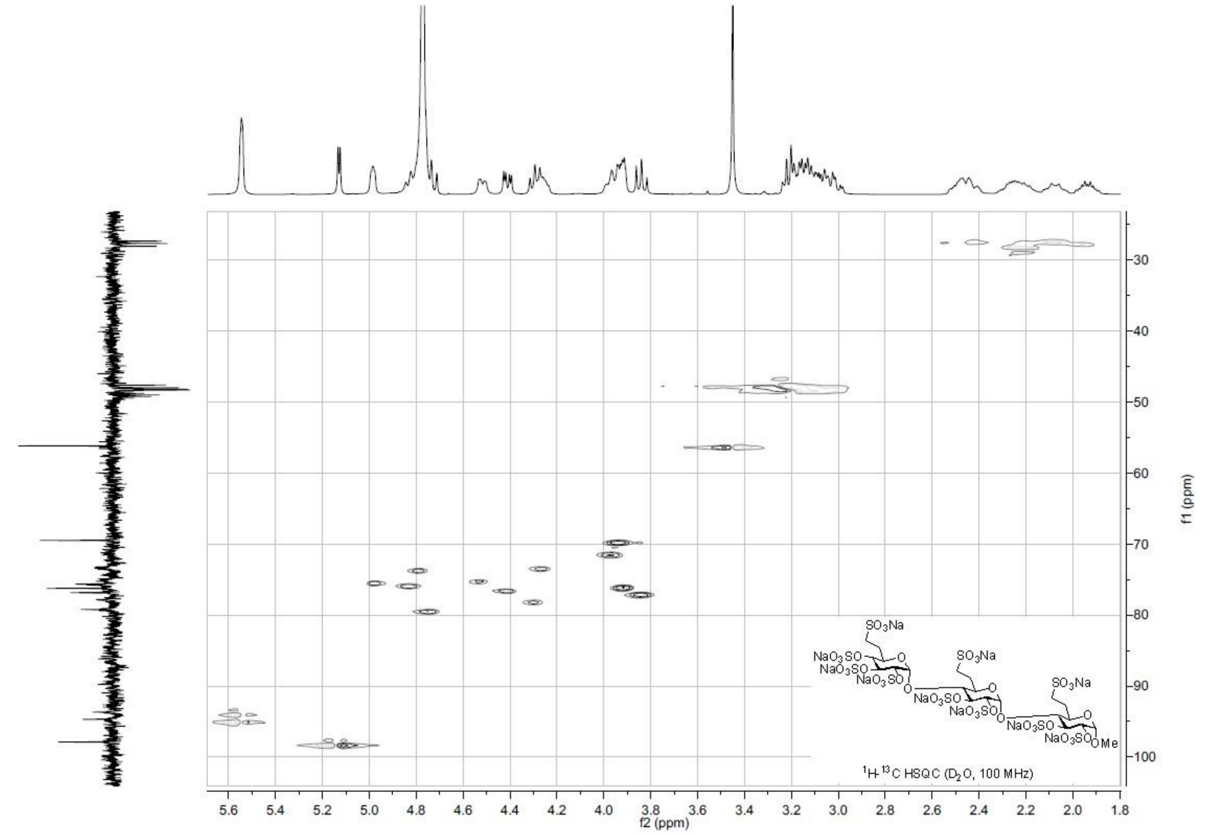

^{1}H-^{13}C HSQC (D_2O, 100 MHz)
f2 (ppm)
f1 (ppm)
